# Discovery of Potent, Orally Bioavailable Sphingosine-1-Phosphate Transporter (Spns2) Inhibitors

**DOI:** 10.1021/acs.jmedchem.4c00879

**Published:** 2024-07-02

**Authors:** Daniel J. Foster, Kyle Dunnavant, Christopher W. Shrader, Marion LoPresti, Sarah Seay, Yugesh Kharel, Anne M. Brown, Tao Huang, Kevin R. Lynch, Webster L. Santos

**Affiliations:** † Department of Chemistry and Virginia Tech Center for Drug Discovery, Virginia Tech, Blacksburg, Virginia 24061, United States; ‡ Department of Biochemistry and Virginia Tech Center for Drug Discovery, Virginia Tech, Blacksburg, Virginia 24061, United States; § Department of Pharmacology, University of Virginia, Charlottesville, Virginia 22908, United States

## Abstract

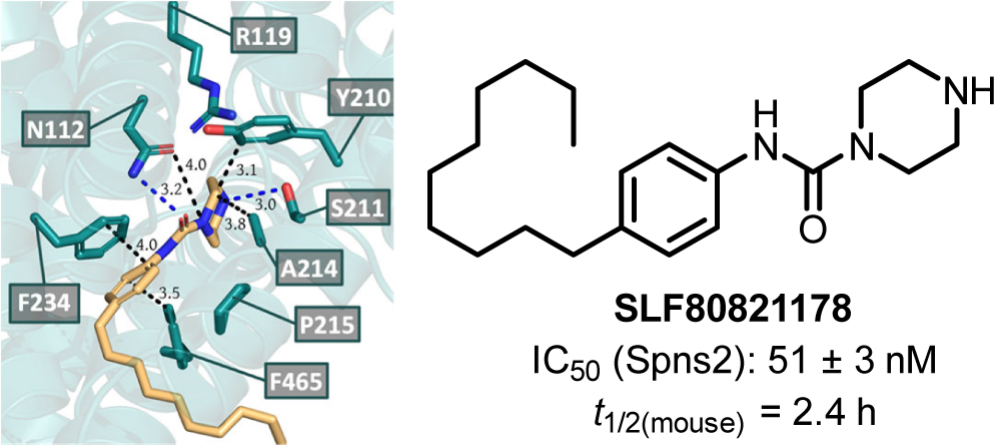

Targeting the S1P pathway has resulted in the development of S1P1 receptor modulators for the treatment of multiple sclerosis and ulcerative colitis. We hypothesize that targeting an upstream node of the S1P pathway may provide an improved adverse event profile. In this report, we performed a structure–activity relationship study focusing on the benzoxazole scaffold in **SLB1122168**, which lead to the discovery of **11i** (**SLF80821178**) as a potent inhibitor of S1P release from HeLa cells (IC_50_: 51 ± 3 nM). Administration of **SLF80821178** to mice induced ∼50% reduction in circulating lymphocyte counts, recapitulating the lymphopenia characteristic of Spns2 null animals. Molecular modeling studies suggest that **SLF80821178** binds Spns2 in its occluded inward-facing state and forms hydrogen bonds with Asn112 and Ser211 and π stacking with Phe234. Taken together, **SLF80821178** can serve as a scaffold for future inhibitor development and represents a chemical tool to study the therapeutic implication of inhibiting Spns2.

## Introduction

**Figure 1:**
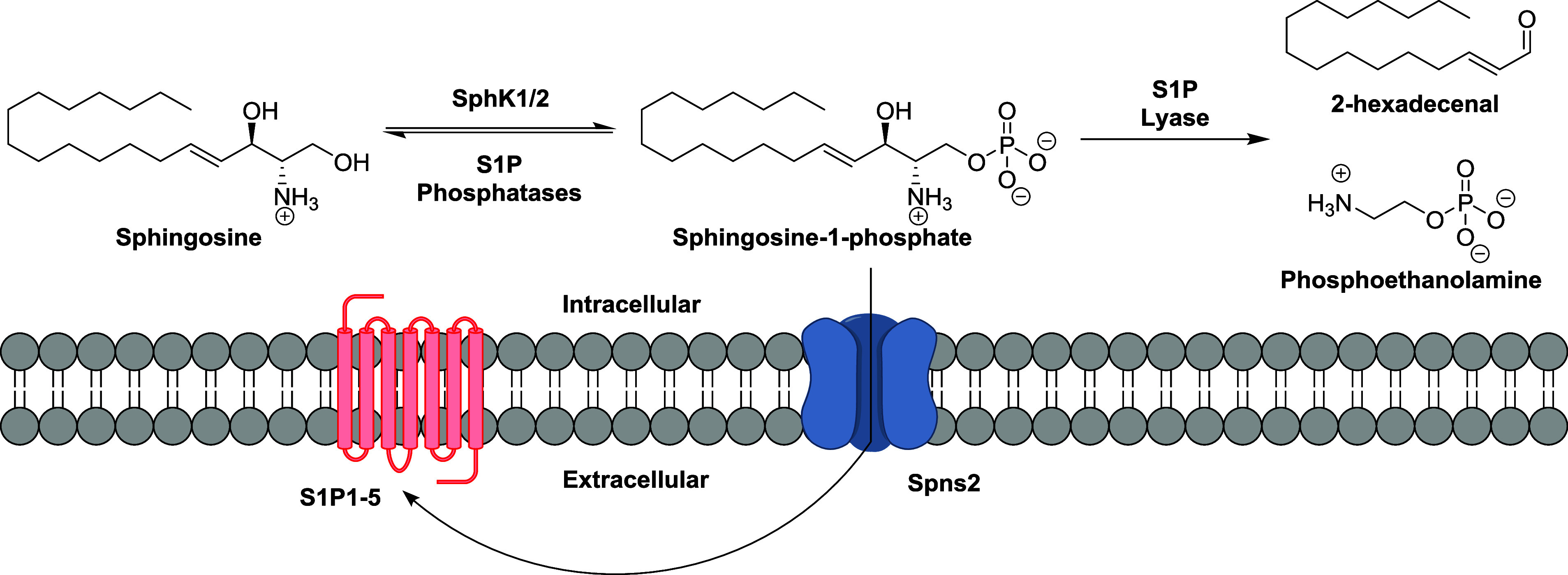
S1P metabolic pathway.

The sphingosine 1-phosphate (S1P) pathway (Figure 1) has been a target by the pharmaceutical industry, which resulted in FDA-approved therapeutics for autoimmune diseases such as multiple sclerosis (MS) and ulcerative colitis (UC).^1−5^ Understanding the mechanism of action of the first orally available MS drug FTY720 (fingolimod, Gilenya) provided the rationale for targeting the S1P receptors (S1P1–S1P5).^6^ Fingolimod is a prodrug. Phosphorylation by sphingosine kinase 2 (SphK2) generates FTY720-phosphate^7,8^ that in turn binds to cell surface S1P receptors on, for example, T-lymphocytes.^9^ The net result of the agonist stimulation is S1P1 receptor desensitization, a blockade of egress of T cells from secondary lymphoid tissues and, ultimately, immunosuppression. To date, four additional S1P receptor modulators (SRMs: siponimod, ozanimod, ponesimod, etrasimod) with the same mechanism of action (S1P1 receptor agonists that drive desensitization) are approved for the MS and/or UC indications.^6,10−13^ While effective, these drugs are characterized by on-target adverse events such as first dose bradycardia and endothelial barrier dysfunction.

Sphingosine is the long chain base precursor of sphingosine 1-phosphate (S1P). Intracellularly, sphingosine is synthesized *de novo* starting with the condensation of the amino acid l-serine and palmitoyl-CoA and ending with the hydrolysis of ceramides.^14^ Two enzymes catalyze the ATP-dependent phosphorylation of the primary alcohol of sphingosine to form S1P: sphingosine kinase 1 (SphK1) and SphK2 (Figure 1).^15,16^ S1P can be dephosphorylated by several nonselective lipid phosphate phosphatases (LPP1–3) and two S1P phosphohydrolases (SPP1–2) to regenerate sphingosine.^17−19^ Irreversible degradation by the endoplasmic reticulum-bound S1P lyase converts S1P to 2-hexadecenal and phosphoethanolamine.^20^ Extracellular S1P acts by binding to a set of G-protein coupled receptors (five in mammals: S1P1–5) on the cell surface.^4,21^ To circumvent adverse events associated with SRMs, other nodes in the S1P pathway can be considered. For example, inhibition of S1P synthesis via SphK inhibitors or S1P degradation via S1P lyase are avenues for pharmacological intervention. Unfortunately, SphK inhibitors have not progressed to the clinic and S1P lyase inhibition results in significant bradycardia and nephrotoxicity in rats.^22,23^

An alternative target upstream of SRMs are the S1P transporters Spns2 and MFSd2b. Spns2 is expressed in endothelial cells and kidney perivascular cells whereas MFSd2b is largely restricted to red blood cells.^24−27^ Studies of Spns2-null mice indicate that this protein supplies lymph S1P,^9,28^ which suggests that the activity of this transporter, like SRMs, controls egress of T cells from lymph nodes,^29^ and thus, this transporter is a potential drug target. Spns2-null mouse strains have been reported to have plasma S1P concentrations ranging from no significant difference^30−32^ to 45% reduction in plasma S1P compared to wild type littermates.^9,24,28,33^ Further, studies of Spns2-null and Spns2 deficient mice demonstrate efficacy in the standard experimental autoimmune encephalomyelitis (EAE) model, collagen-induced arthritis, and (dextran sulfate sodium) DSS-induced colitis further illustrating the potential of this transporter as a therapeutic target.^29,34^

**Figure 2:**
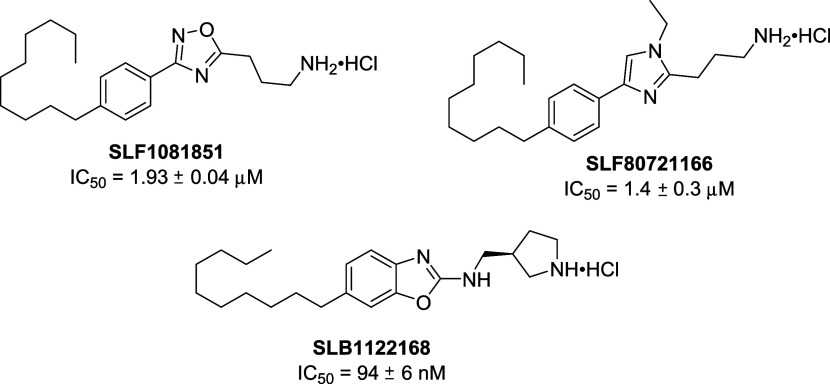
Structures of reported Spns2 inhibitors.^36−38^

Our long-standing interest in the S1P pathway has prompted investigations of Spns2 as a viable therapeutic target.^35^ Recently, we reported a structure–activity relationship (SAR) study that identified prototypes **SLF1081851** (IC_50_ 1.93 ± 0.04 μM)^36^ and **SLF80721166** (IC_50_ 1.4 ± 0.3 μM) (Figure 2).^37^ Additional investigations revealed a second generation Spns2 inhibitor, **SLB1122168** (IC_50_ 94 ± 6 nM), which contained a key benzoxazole scaffold.^37,38^ When administered to rodents, these inhibitors were effective in modulating the immune system as evidenced by a decrease in circulating lymphocytes,^36,38^ which is a hallmark of Spns2 inhibition. In a mouse model of kidney fibrosis (unilateral ischemia-reperfusion injury), **SLF1081851** suppressed inflammatory signaling in perivascular cells and ameliorated kidney fibrosis (Figure 2).^25^

**Figure 3:**
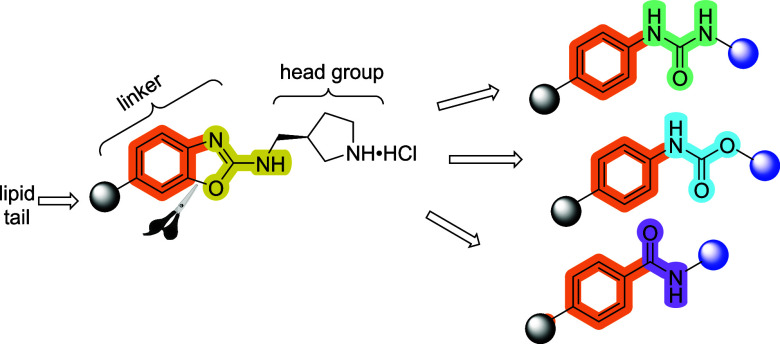
Strategy toward new Spns2 inhibitors.

Although useful as chemical biology tools to study the effects of Spns2 inhibition, previous inhibitors were not optimal. For example, **SLF1081851** was toxic to mice at a dose 30 mg/kg while **SLB1122168** had poor oral bioavailability (Figure 2).^36,38^ In this study, we aimed to develop Spns2 inhibitors with improved toxicity and pharmacokinetic profiles. Starting with **SLB1122168**, we envisioned that breaking the C–O bond of benzoxazole will provide functional groups such as urea,^39^ carbamate,^40^ and benzamide^41^ that will allow us to probe both the structure–activity relationship as well as pharmacokinetic profiles (Figure 3). These functional groups can provide hydrogen bond donor–acceptor interactions, electronic effects, and varying conformations. Our studies revealed that each of the benzamide, phenylcarbamate, and phenylurea series yielded examples of Spns2 inhibitors that are potent *in vitro*. In particular, we discovered **SLF80821178** as the most potent compound with an IC_50_ of 51 ± 3 nM and demonstrated *in vivo* activity in inducing a decrease in peripheral blood lymphocytes in mice after oral administration. In addition, molecular docking studies identified several residues of the transporter responsible for binding.

## Results and Discussion

**Scheme 1:**
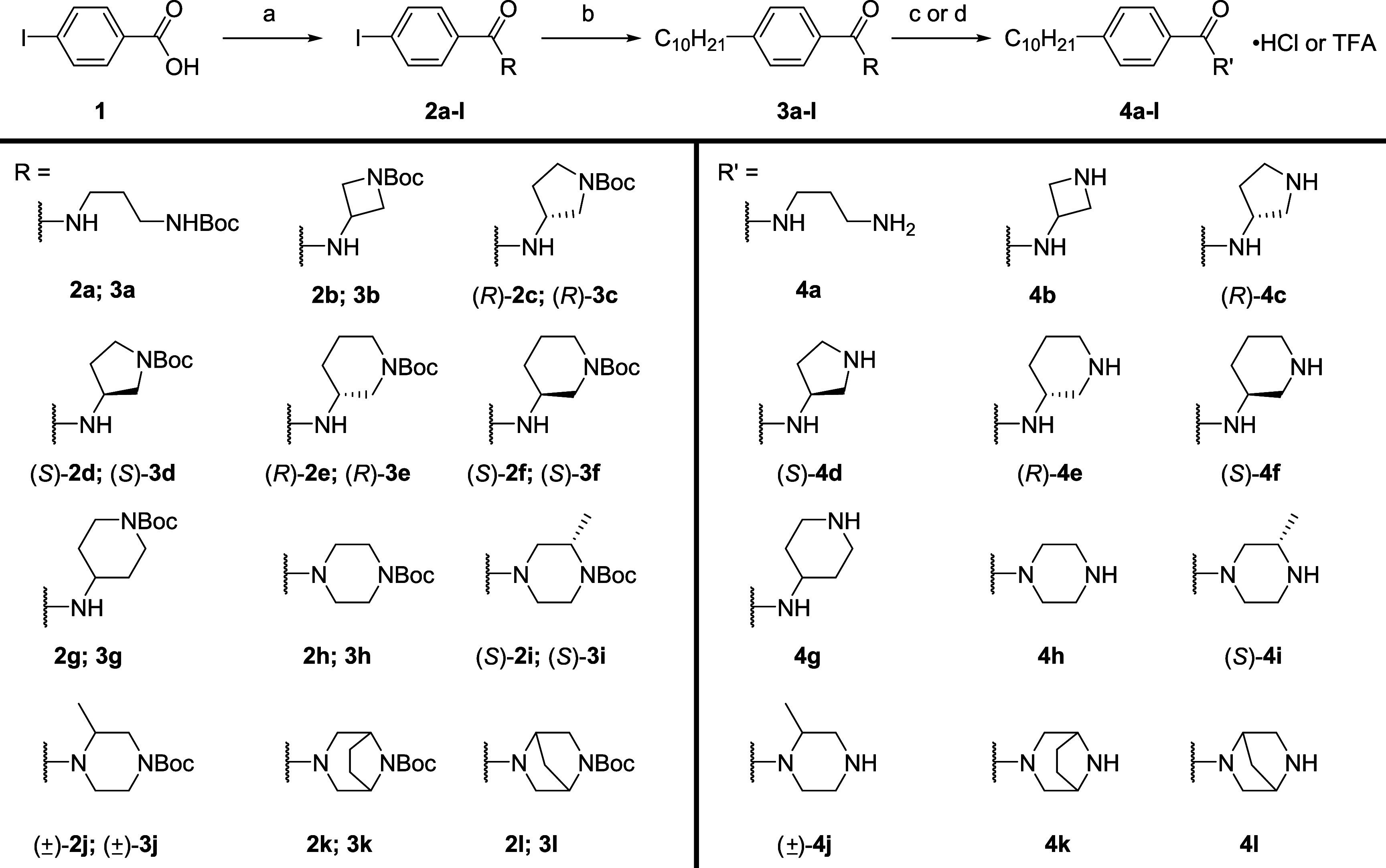
Synthesis of Benzamide Analogs **4a**–**l** (a) Mono-*N*-Boc protected diamine, HCTU, DIEA, CH_2_Cl_2_, rt, 16 h, 92–99%; (b) (i) 1-decene, 9-BBN, THF, 66 °C, 2 h; (ii) aryl iodides **2a**–**l**, Pd(dppf)Cl_2_·CH_2_Cl_2_, KOH-aq, THF, 66 °C, 4 h, 74–98%; (c) 4 M HCl/dioxane, CH_2_Cl_2_, rt, 2 h, 67–93%; (d) TFA, CH_2_Cl_2_, rt, 2 h, 67%.

The synthesis of benzamide derivatives is shown in Scheme 1. Cyclic or linear mono-*N*-Boc protected diamines were formed by condensation with 4-iodobenzoic acid (**1**) in good yields using HCTU.^36^ The corresponding *N*-Boc protected benzamides **2a**–**l** were coupled to 1-decene through a one-pot hydroboration and Suzuki–Miyaura cross-coupling reaction. Following alkyl tail attachment, the protected amines **3a**–**l** were treated with hydrochloric acid or trifluoroacetic acid to yield derivatives **4a**–**l** as ammonium salts.

**Scheme 2:**
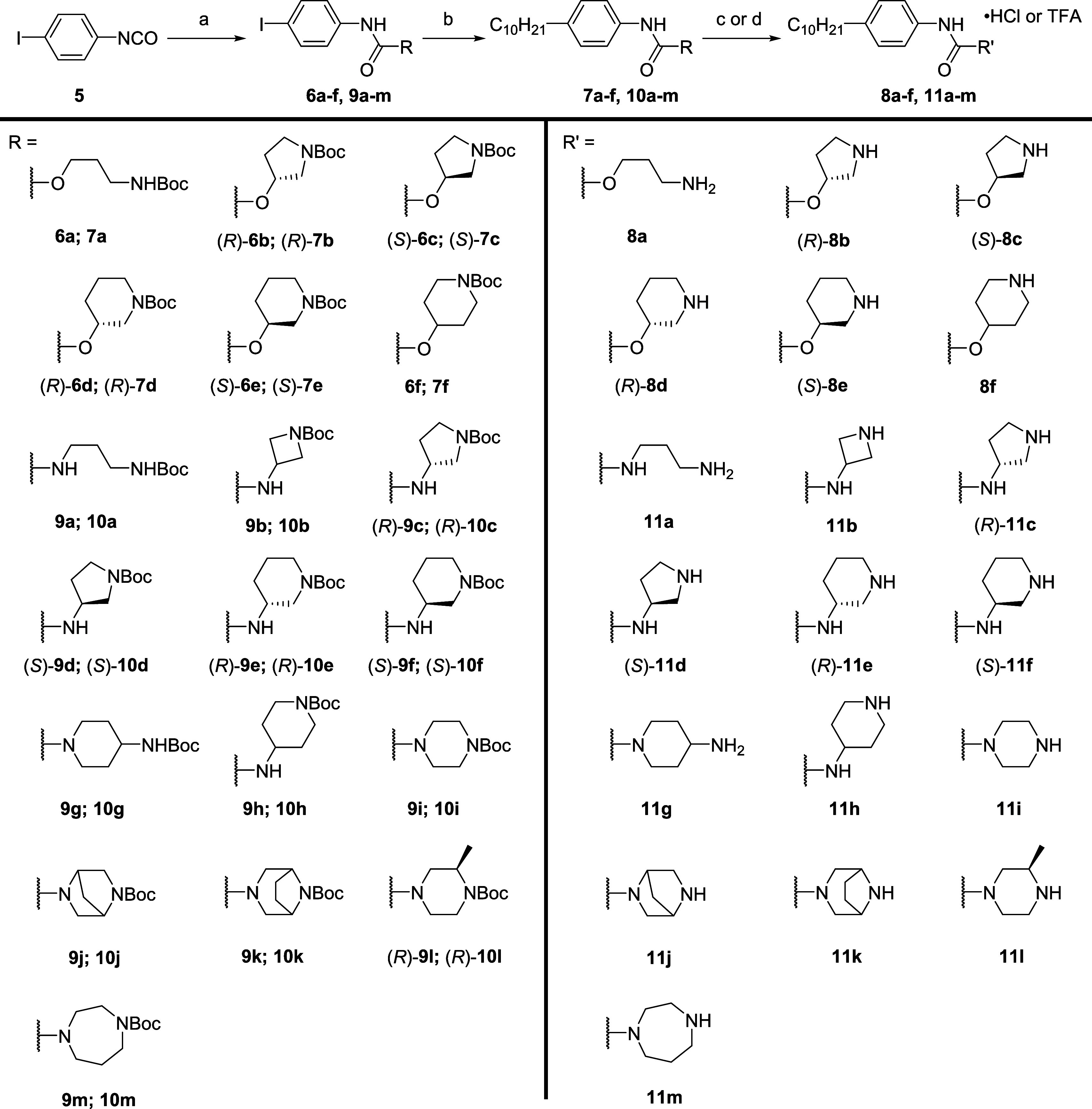
Synthesis of Phenylcarbamate **8a**–**f** and Phenylurea **11a**–**m** (a) *N*-Boc protected amino alcohol or mono-*N*-Boc protected diamine, CH_2_Cl_2_, 0 °C to rt, 16 h, 84–99%; (b) (i) 1-decene, 9-BBN, THF, 66 °C, 2 h; (ii) aryl iodides **9a**–**m**, Pd(dppf)Cl_2_·CH_2_Cl_2_, KOH-aq, THF, 66 °C, 4 h, 46–93%; (c) 4 M HCl/dioxane, CH_2_Cl_2_, rt, 2 h, 57–87%; (d) TFA, CH_2_Cl_2_, rt, 2 h, 50%.

To synthesize phenylcarbamate and urea derivatives, 4-iodophenyl isocyanate **5** was treated with a mono-*N*-Boc protected amino alcohol or diamine in the presence of a base to generate the protected intermediates **6a**–**f** and **9a**–**m** (Scheme 2). Hydroboration of 1-decene with 9-BBN was followed by cross-coupling with **6a**–**f** and **9a**–**m** under Suzuki–Miyaura conditions afforded **7a**–**f** and **10a**–**m**, which were then deprotected with hydrochloric acid to remove the Boc protecting groups and yield structures **8a**–**f** and **11a**–**m** as hydrochloride or TFA salts.

**Scheme 3:**
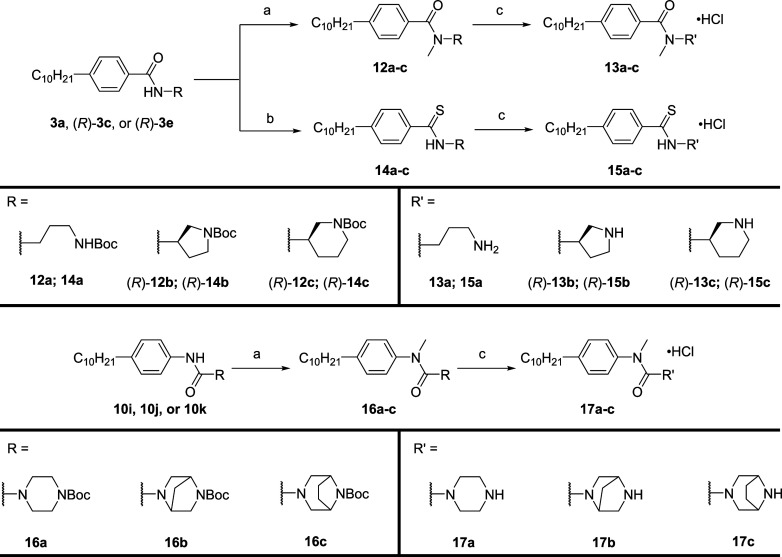
Synthesis of Analogs **13a**–**c**, **15a**–**c**, and **17a**–**c** (a) (i) NaH, THF, 0 °C, 0.5 h; (ii) MeI, THF, 0 °C to rt, 16 h, 51–85%; (b) Lawesson’s reagent, THF, rt, 16 h, 68–80%; (c) 4 M HCl/dioxane, CH_2_Cl_2_, rt, 2 h, 46–94%.

Selected benzamide and urea analogs were functionalized further through *NH* methylation and thionation of selected benzamide derivatives to thioamides (Scheme 3). The *N*-Boc protected compounds **3a**, (*R*)-**3c**, (*R*)-**3e**, **10i**, **10j**, and **10k** were treated with sodium hydride and alkylated with methyl iodide to afford intermediates **12a**–**c** and **16a**–**c**, which were then treated with hydrochloric acid to liberate the desired ammonium salts **13a**–**c** and **17a**–**c**. Thioamides **15a**–**c** were achieved by reacting the *N*-Boc protected compounds **3a**, (*R*)-**3c**, and (*R*)-**3e** with Lawesson’s reagent to afford **14a**–**c** and subsequent deprotection with hydrochloric acid to generate the inhibitors **15a**–**c**.

**Scheme 4:**
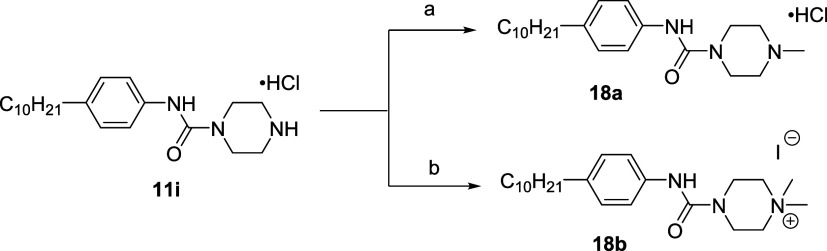
Derivatization of **11i** Through *N*-methylation of the Piperazine Nitrogen (a) (i) Paraformaldehyde, formic acid, MeOH, 65 °C, 6 h; (ii) 4 M HCl/dioxane, CH_2_Cl_2_, rt, 2 h, 50%; (b) MeI, K_2_CO_3_, MeCN, rt, 4 h, 83%.

Monomethylation and hypermethylation of the terminal piperazine nitrogen were obtained from the secondary ammonium chloride **11i** (Scheme 4). The tertiary amine derivative **18a** was prepared using Eschweiler–Clarke reaction conditions.^42^ The resulting intermediate was neutralized and treated with hydrochloric acid to afford **18a** as a hydrochloride salt. Exposure of **11i** to excess methyl iodide and base yielded the quaternary ammonium iodide **18b**.

**Scheme 5:**
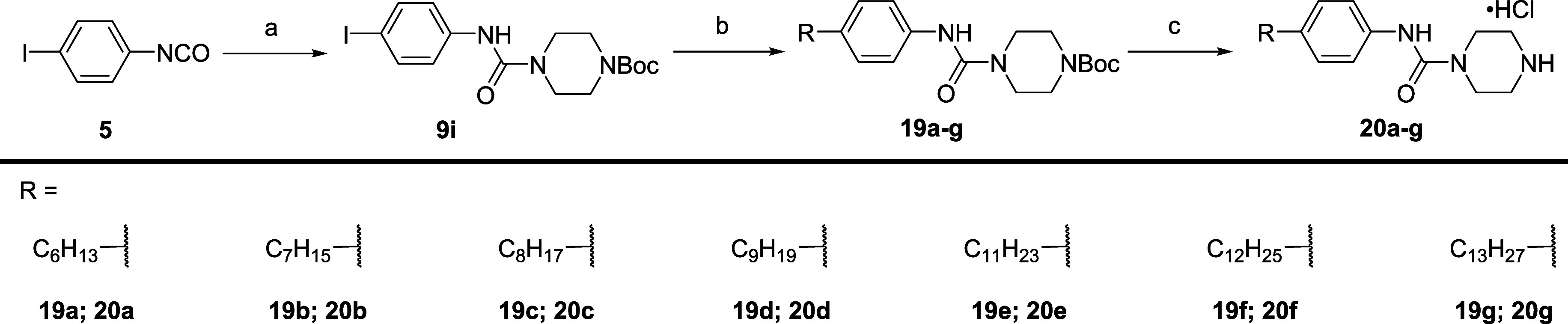
Synthesis of Tail Homologation Series **20a**–**g** (a) 1-Boc-piperazine, CH_2_Cl_2_, 0 °C to rt, 16 h, 96%; (b) (i) alkene, 9-BBN, THF, 66 °C, 2 h; (ii) aryl iodides **9i**, Pd(dppf) Cl_2_·CH_2_Cl_2_, KOH-aq, THF, 66 °C, 4 h, 46–93%; (c) 4 M HCl/dioxane, CH_2_Cl_2_, rt, 2 h, 57–87.

Subsequent focus on the effect of alkyl tail length to activity led to the preparation of several phenyl urea derivatives with varying alkyl tail lengths bearing the piperazine headgroup **20a**–**g** (Scheme 5). Alkyl tail attachment was achieved through a two-step, one-pot hydroboration and Suzuki–Miyaura cross-coupling reaction between requisite terminal alkene and **9i**. Removal of the Boc protecting groups of intermediates **19a**–**g** with hydrochloric acid provided analogs **20a**–**g** as ammonium salts.

### Analysis of Benzamide, Phenylcarbamate, and Phenyl Urea Scaffolds

With a library of analogs of **SLB1122168** containing phenylurea, phenylcarbamate, and benzamide as bioisostere of the benzoxazole, we investigated their potential in inhibiting S1P transport using a HeLa cell assay.^36−38,43^ Briefly, in this assay, Spns2 is overexpressed whereas the S1P-degrading enzymes (lyase or phosphatases) were blocked with inhibitors.^44,45^ Inhibition of S1P transport is determined by measuring the amount of S1P in the buffer using LC-MS. As shown in Table 1, one micromolar benzamide inhibitors were added in the assay performed in triplicate with **SLB1122168** and **SLF1081851** as controls.

**Table 1:**
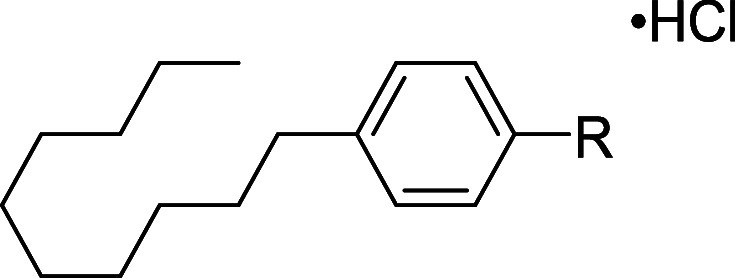

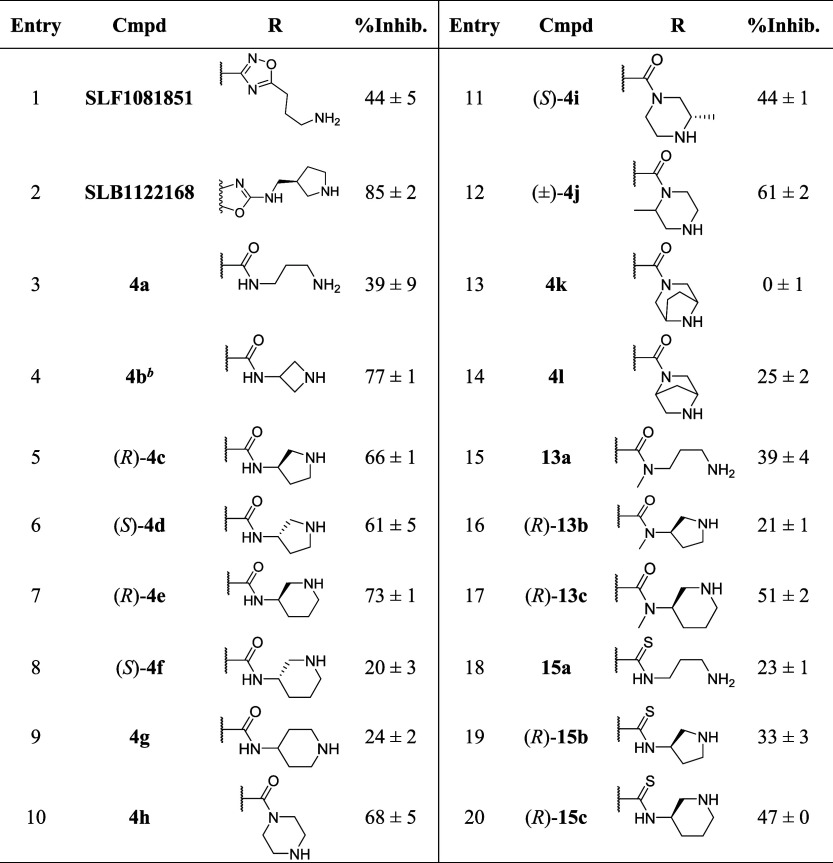
Spns2 Inhibition of Benzamide, Thiobenzamide, and *N*-Methyl Benzamide Containing Compounds^a^^b^

a Spns2 inhibition is reported as the percent decrease in S1P secretion relative to the control. Compounds were assayed with 1 μM inhibitor. Cell media was extracted, and S1P concentrations were measured by LC-MS/MS. Assays were performed in duplicate.

b Compound is a trifluoroacetate salt.

The direct propyl amine analog **4a** had similar inhibitory activity as **SLF1081851**. The azetidine bearing derivative **4b** showed a significant increase in inhibition (77%) whereas the 5-membered ring analogs β-pyrrolidine (*R*)-**4c** and (*S*)-**4d** had similar potency at 61–66% inhibition. Expanding the ring further to a 6-membered piperidine (*R*)-**4e** (73%) showed an improvement in potency and, interestingly, was significantly more potent than its enantiomer (*S*)-**4f** (20%). Moving the nitrogen by one carbon to form the symmetric piperidine **4g** exhibited a drastic reduction in activity at 24% inhibition. To our delight, removing the nitrogen “spacer” from **4g** to generate **4i** restored inhibition to 68% while attempts to induce conformational restriction by addition of methyl groups in **41–4j** or bicyclic structures **4k**–**4l** did not lead to improved inhibitory activity. To investigate the effect of the amide *NH*, we methylated key compounds **13a–c** and found that methylation has a negative impact in the activity potentially because of the loss of a hydrogen bond donor or steric encumbrance. Likewise, thioamides **15a–c** did not show improved activity.

The results of the phenylcarbamate derivatives are shown in Table 2. The primary amine **8a** had moderate inhibition at 44% whereas the (*R*)-pyrrolidine analog **8b** showed increased potency (68%) relative to **8a** as well as enantiomer (*S*)-**8c**. Further increase in ring size as well as investigating stereochemistry and position of the nitrogen did not improve potency (i.e., **8d**–**f**).

**Table 2:**
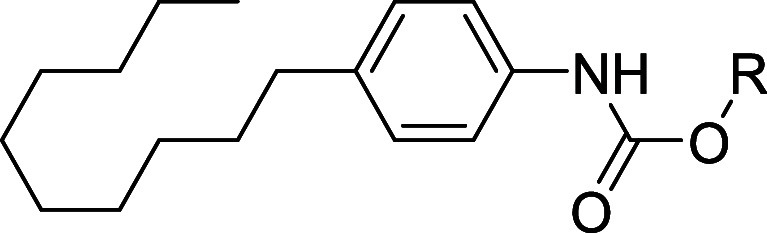

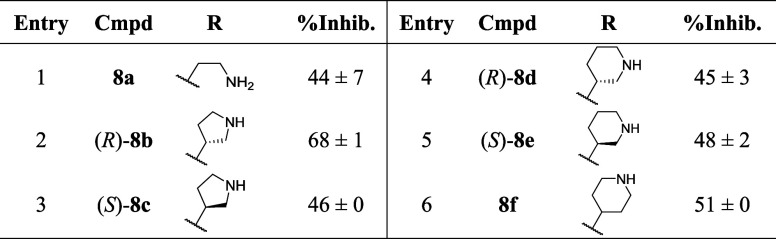
Spns2 Inhibition of Phenylcarbamate Derivatives^a^

a Spns2 inhibition is reported as the percent decrease in S1P secretion relative to the control (no inhibitor). Compounds were assayed with 1 μM inhibitor. Cell media was extracted, and S1P concentrations were measured by LC-MS/MS. Assays were performed in duplicate.

The results of the S1P transport inhibition of the phenyl urea linker are shown in Table 3. The propyl amine **11a** showed a moderate 53% Spns2 inhibition. While the cyclic azetidine headgroup exhibited a slightly increased inhibition at 59%, both enantiomers of the β-pyrrolidine head groups ((*R*)-**11c** and (*S*)-**11d**) had modest Spns2 inhibition. Increasing the ring size further with the β-piperidine analogs (*R*)-**11e** and (*S*)-**11f** profoundly improved Spns2 inhibition at 86% and 78%, respectively. Further investigating the effect of piperidine-bearing head groups, 4-aminopiperidine analog **11g** (59%) and the symmetric piperidine analog **11h** (34%) were synthesized and tested, but a decrease in inhibition was observed. Interestingly, the introduction of the piperazine head in **11i** resulted in a significant decrease (91%) in S1P transport. Further, SAR on this key functional group where conformational restriction via bridged ring systems (**11j**–**k**), alkylation ((*R*)-**11l**), or ring expansion (**11m**) were largely unfruitful. To investigate the effect of urea N–H hydrogen bonding, we methylated **11i**–**k** and discovered that this group likely plays a role in binding as **17a**–**c** had a corresponding decrease in potency.

**Table 3:**
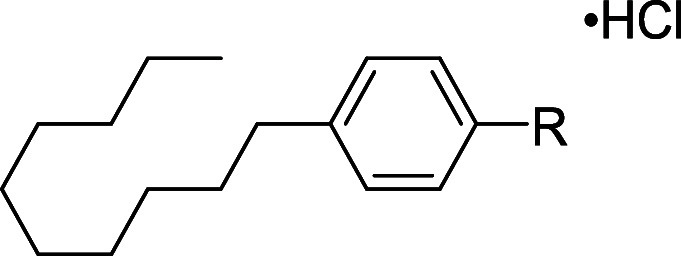

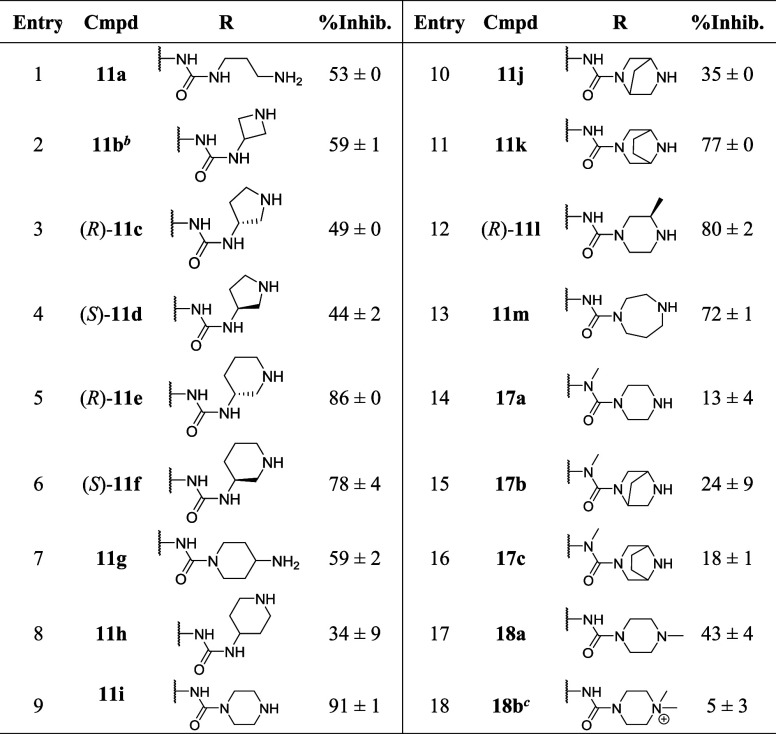
Spns2 Inhibition of Phenylurea and *N*-Methyl Phenylurea Containing Compounds^a^^b^^c^

a Inhibitory data for diversified phenyl urea analogs. Spns2 inhibition is reported as the percent decrease in S1P secretion relative to the control. All compounds were assayed with 1 μM inhibitor. Cell media was extracted, and S1P concentrations were measured by LC-MS/MS. Assays were performed in duplicate.

b Compound is a trifluoroacetate salt.

c Co mpound is an iodide salt.

Similarly, the importance of the hydrogen-bond donor of the piperazine nitrogen was also probed with tertiary (**18a**) and quaternary (**18b**) amine analogs of **11i** by the methylation of the amine moiety. Both analogs showed a drastic loss in activity suggesting that a hydrogen bond donor is important and that steric interaction within the protein binding site is occurring (*vide infra*). In summary, **11i** was revealed to be the most potent analog among the benzamide, phenylcarbamate, and phenyl urea series.

**Figure 4:**
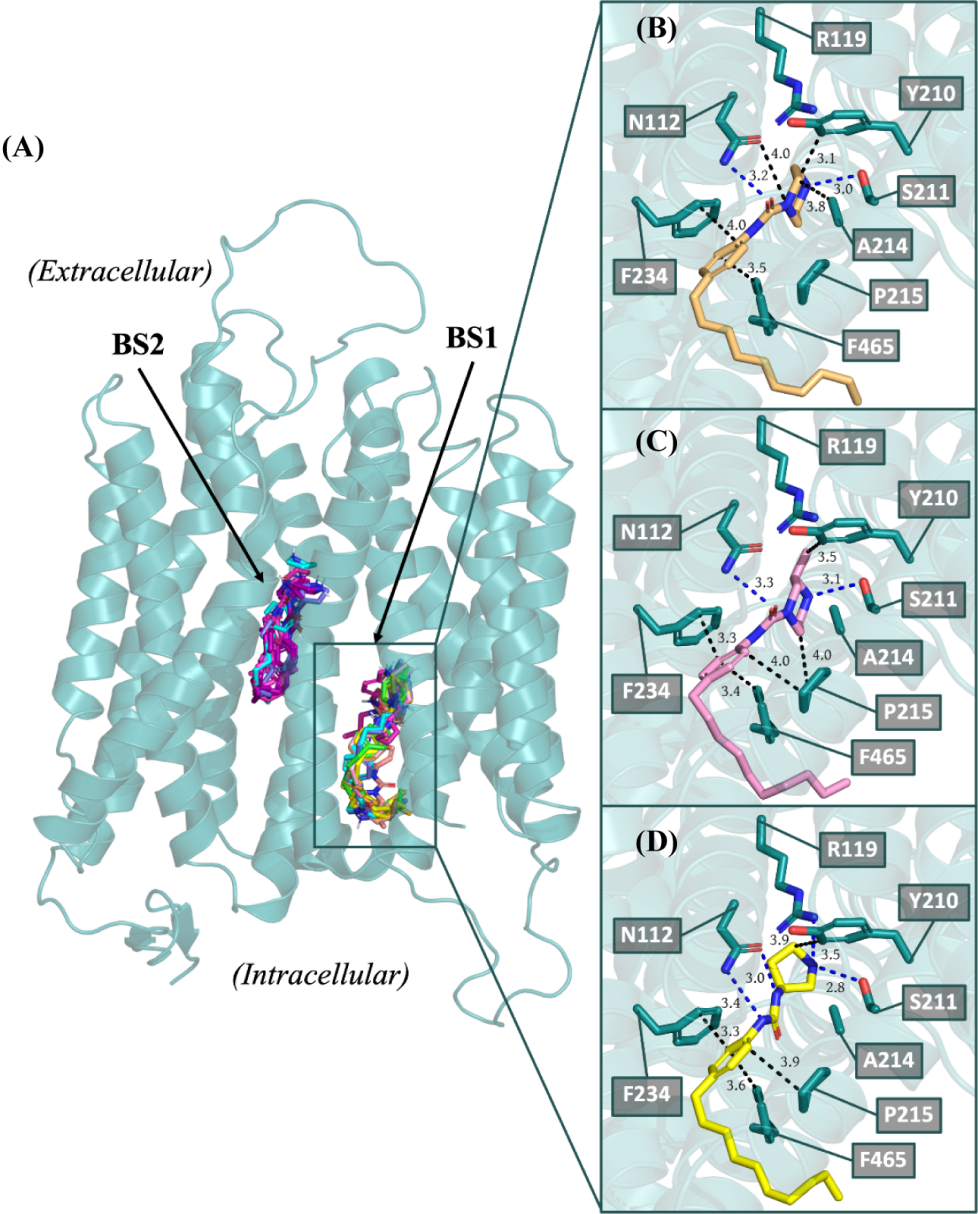
(A) Docked poses of inhibitors binding within the transport channel of Spns2. Comparison of the docked poses of (B) **11i**, (C) (*R*)-**11l**, and (D) (*R*)-**11c** in dominant cluster from MD simulations of Spns2 (Homology model generated from I-TASSER using PDB ID: 6E8J as a primary template. The simulated homology model of SPNS2 used in this work is deposited on OSF (https://osf.io/82n73/)).^51^ Key residues in the transporter channel are represented by teal sticks and labeled. Inhibitors are shown as sticks and colored by element, with the carbon atom indicating an inhibitor (**11i**, orange; (*R*)-**11l**, pink; (*R*)-**11c**, yellow). BS1: binding site 1, BS2: binding site 2.

To understand the intermolecular interactions of **11i** and related analogs, we performed *in silico* docking with cryo-EM structures of Spns2. Recently, three groups independently reported the cryo-EM structure of Spns2 for a total of four apo (PDB IDs: 7YUF, 8EX5, 8EX7, and 8EX8) and three S1P bound states (PDB IDs: 7YUB, 8JHQ, and 8EX4).^46−48^ Three inhibitors ((*R*)-**11c**, **11i**, and (*R*)-**11l**) bearing the phenyl urea linker were docked into the dominant morphology from a molecular dynamics (MD) simulation that involved an AlphaFold2^49,50^ homology model simulated in the presence of an asymmetric model membrane representative of a general plasma membrane and S1P.^51^ These inhibitors include two of the most potent analogs of the phenyl urea series (**11i**, (*R*)-**11l**) along with a less potent inhibitor ((*R*)-**11c**) to determine the correlation of predicted free energies of inhibitor-transporter interactions to the in vitro Spns2 inhibition. The majority of apo crystal structures are resolved in the outward state, with only one structure in the inward-facing state (PDB 7YUF).^46^ Our MD-simulated structure samples an occluded inward-facing state, which is defined by the partial occlusion of the intracellular side caused by TM11 and TM5 moving toward each other.^51^ When docking Spns2 inhibitors to crystal structure PDB 8EX4 (inward-facing S1P bound state), these inhibitors bind to only one area of the transport channel and predicted free energy of binding MM/GBSA calculations rank inhibitors inaccurately compared to experimental values. However, performing molecular docking to the occluded inward-facing state matches experimental data and indicates that inhibitors (*R*)-**11c**, **11i**, and (*R*)-**11l** target two separate binding sites within the transport channel of Spns2 (Figure 4A). **11i** and (*R*)-**11l** both contain a piperazine headgroup whereas (*R*)-**11c** has a 3-aminopyrrolidine. The two observed binding sites include one site proximal to the intracellular space (BS1) and another connected but adjacent site that is further sequestered within the transport channel of Spns2 (BS2). BS2 is identified as the same binding site targeted by S1P, FTY720-P, and previously reported inhibitors as reported with crystal structures and cryo-EM studies of Spns2.^46−48^ Interestingly, predicted free energy of binding calculations from these docking results with our inhibitors binding at BS2 are less favorable (mean MM/GBSA is −30.0 ± 11.2 kcal/mol) compared to binding at BS1 (mean MM/GBSA is −67.8 ± 7.4 kcal/mol). Further investigation into inhibitor-Spns2 binding at the BS1 site reveals common key interactions with Asn112 and Ser211. All three inhibitors show hydrogen bonding with Ser211; however, there are discernible differences in hydrogen bond between Asn112 and the inhibitors. The oxygen of the urea moiety of the top two inhibitors (exhibiting ≥80% reduction of S1P transport), **11i** and (*R*)-**11l**, interact with the nitrogen from the Asn112 side chain. In these hydrogen bonding pairs, the side-chain *NH* acts as the donor while the urea *O* acts as the acceptor. Interestingly, this interaction is absent with the less effective inhibitor, (*R*)-**11c**, suggesting its potential significance. The carbonyl of the urea group in (*R*)-**11c** is rotated away from Asn112 likely due to increased flexibility and change in the orientation of the pyrrolidine nitrogen. Another common feature among the three inhibitors is a prominent interaction of the aryl ring to the phenyl ring of Phe234, which likely results in a π stacking interaction. Differences between the top two inhibitors are far subtler; however, it appears that the methyl on the piperazine warhead of (*R*)-**11l** is 3.5 Å away from Tyr210 whereas the piperazine warhead on **11i** is only 3.2 Å away from Tyr210 suggesting that the methyl group may be causing some steric interaction that positions the urea moiety and piperazine further away from Asn112 and Ser211, respectively, by about 0.1 Å in the top poses. Weaker hydrophobic interactions can be observed between the inhibitors to the residues Ala214 and Pro215. The *in silico* analysis of these inhibitors produced mean MM/GBSA values of −44.8 kcal/mol, −64.8 kcal/mol, and −61.5 kcal/mol for (*R*)-**11c**, **11i**, and (*R*)-**11l**, respectively. This matches the trend observed for the reduction of S1P transport in HeLa cells for these inhibitors ((*R*)-**11c**, 49%, (*R*)-**11l**, 80%, **11i**, 91%). Docking to the occluded inward-facing state of Spns2 resulted in remarkable alignment between predicted inhibitor free-energy MM/GBSA calculations and experimental inhibitory values, leading to the suggestion that these inhibitors are targeting this specific occluded inward-facing state with two binding sites.

**Figure 5:**
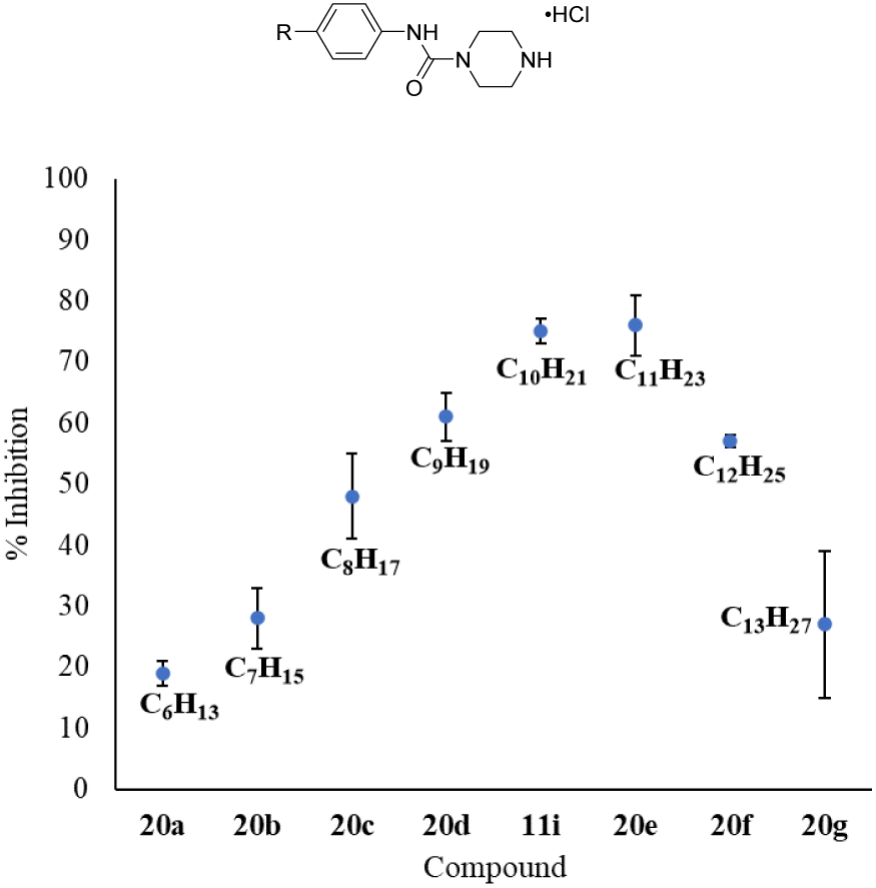
Tail homologation study of 11i. Spns2 inhibition is reported as the percent decrease in S1P secretion relative to the control. All compounds were assayed with 0.3 μM inhibitor and performed in duplicate. Cell media was extracted, and S1P concentrations were measured by LC-MS/MS.

With **11i** in hand, we performed a tail homologation study to determine the optimal alkyl length. As shown in Figure 5, compounds were tested at 0.3 μM to improve the resolution of the assay. Our SAR study suggests that as the alkyl tail increases from a hexyl to tridecyl concomitant potencies resembling a bell curve is observed with a maximum at ten to 11 carbon length. While the undecyl tail in **20e** has comparable activity to decyl chain in **11i**, the additional methylene increases both the number of rotatable bonds and overall lipophilicity of the compound, likely leading to an unfavorable profile for expected oral bioavailability desired for pharmaceuticals.^52,53^ Therefore, **11i** was selected for further investigation.

**Figure 6:**
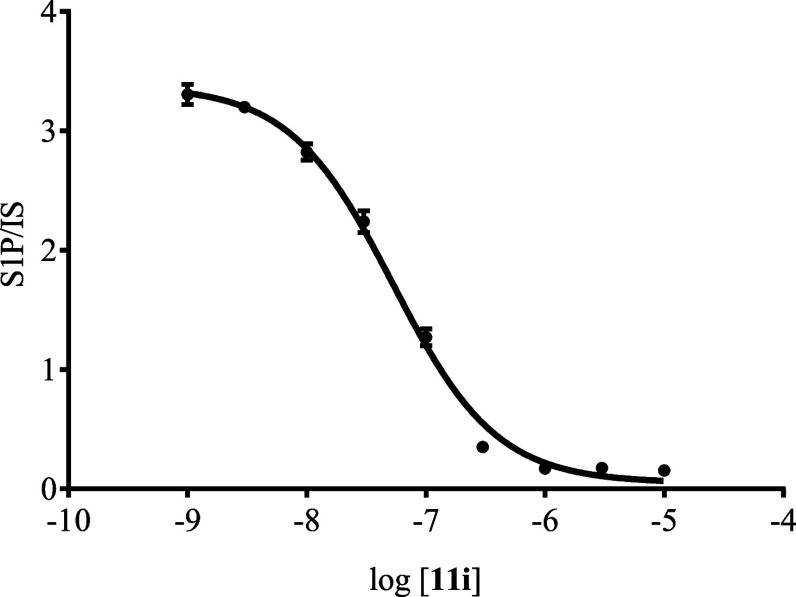
Dose–response assessment of 11i in HeLa cells. The assay was performed in triplicate.

To determine the potency of **11i**, the compound was subjected to a dose–response assay in HeLa cells transfected with Spns2-encoding plasmid DNA (Figure 6). As the concentration of **11i** increased from 1 nM to 10 μM, a dose-dependent decrease in S1P extruded into media was observed. From this assay, it was determined that the **11i** possessed an IC_50_ value of 51 ± 3 nM, an improvement in potency compared to **SLF1081851** (IC_50_: 1.93 ± 0.04 μM)^36^ and **SLB1122168** (94 ± 6 nM).^38^ In fact, **11i** is among the most potent small molecule inhibitors of S1P release by Spns2-expressing that we have discovered to date.

### Pharmacodynamic and Pharmacokinetic Assessment of 11i

**Figure 7:**
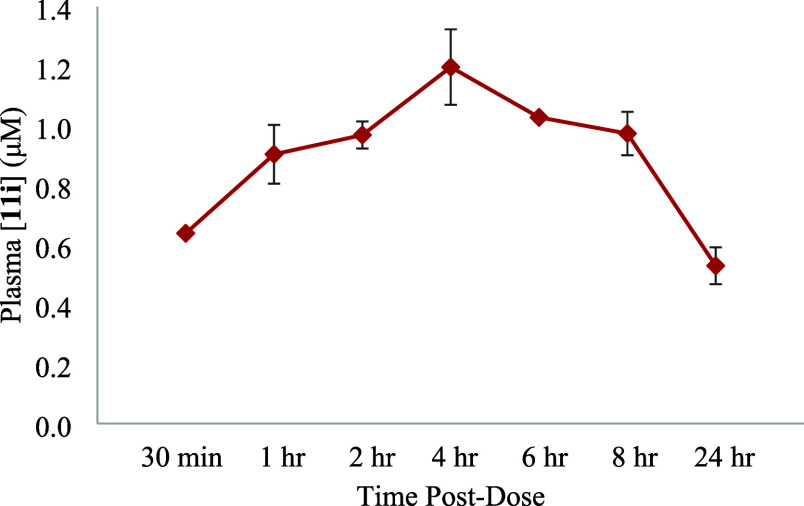
Compound concentrations in plasma of mice treated with **11i**. Three C57BL/6j strain female mice (8–10 weeks old) were treated with a single 10 mg/kg dose of **11i** administered PO. Blood samples were collected, and plasma **11i** levels were quantified via LC-MS/MS.

We next investigated the potential oral bioavailability of **11i** (**SLF80821178**), which will be a significant advantage over **SLB1122168**. Previous work indicated that **SLB1122168** is not orally bioavailable.^38^ Thus, mice were administered a single 10 mg/kg oral dose of **11i**, and compound levels in plasma were quantified. As shown in Figure 7, circulating levels of **11i** reached a maximum at 4 h after treatment and remained elevated above 0.5 μM for more than 8 h. These results suggest that **11i** is orally bioavailable and capable of sustained exposure to assess *in vivo* effects of the compound.

**Figure 8:**
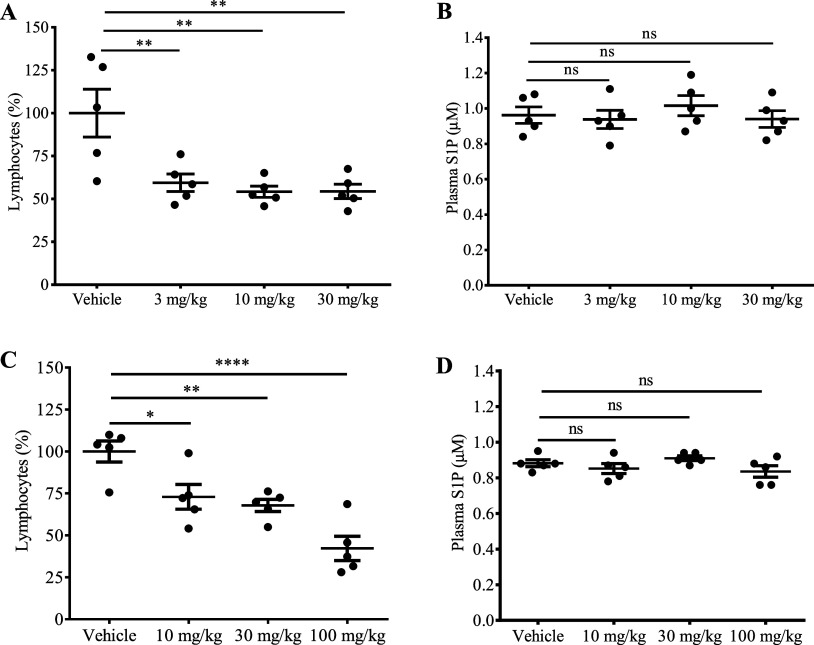
Biological evaluation of **11i** in five C57BL/6j strain female mice (8–10 weeks old). (A) IP administration of inhibitor resulted in a decrease in circulating lymphocytes at 3 mg/kg, 10 mg/kg, and 30 mg/kg compared to the vehicle. Blood was drawn 4 h postdose. (B) Plasma S1P concentrations in mice treated with **11i** are shown relative to vehicle 6 h following IP administration at 3 mg/kg, 10 mg/kg, and 30 mg/kg. (C) PO administration of inhibitor resulted in decrease in circulating lymphocytes at 10 mg/kg, 30 mg/kg, and 100 mg/kg. Blood was drawn 4 h postdose. (D) Plasma S1P concentrations in mice treated with **11i** are shown relative to vehicle 4 h post oral administration at 10 mg/kg, 30 mg/kg, and 100 mg/kg. One-way ANOVA followed by Sidak’s multiple comparison tests (*≤0.05; **≤0.01; ***≤0.001; ****≤0.0001; ns = not statistically significant) were used.

To determine the effect of **11i***in vivo*, mice were treated with 3, 10, and 30 mg/kg intraperitoneally, and the amount of circulating lymphocytes was quantified. A hallmark of Spns2 inhibition is the decrease in lymphocytes counts, which is a pharmacodynamic marker of target engagement.^54,55^ As shown in Figure 8A, a decrease in lymphocyte count was observed that reached to a maximum level of 50%, which is also observed in Spns2-null mice.^3,30^ Encouraged by this result, we tested whether **11i** is efficacious when given orally. Indeed, administration of **11i** via oral gavage resulted in a dose-dependent lymphocytopenia with 100 mg/kg dose achieving maximum effect (Figure 8C). To determine whether blood S1P levels were affected, plasma S1P concentrations were measured via LC-MS. As shown in Figure 8B,D, no change in plasma S1P levels was observed when **11i** was administered IP or PO. Our studies suggest that small molecule inhibition of Spns2 has minimal effect in plasma S1P levels, which is corroborated with our prior work.^38^ The extent by which Spns2 inhibition by knockout or a pharmacological inhibitor affects the S1P level remains unclear. A range of changes from no significant difference^30−32^ to 45% reduction in plasma S1P levels compared to wild type littermates have been reported.^9,24,28,33^ In summary, our SAR study identified **11i** as a potent inhibitor of Spns2 that is orally bioavailable and elicits lymphocytopenia upon target engagement.

## Conclusions

In this report, we performed a structure–activity study of Spns2 inhibitors using **SLB1122168** as a starting point. We envisioned benzamide, phenyl urea, and phenylcarbamate as bioisosteres of the benzoxazole scaffold. For each of these scaffolds, the nature of the headgroup that bears an amine was investigated including both linear and cyclic analogs. The phenylcarbamate series afforded more potent compounds. Overall, cyclic secondary amines were favored with the piperazine moiety being optimal for the phenyl urea and phenylcarbamate isosteres. Increasing the ring size from six to seven or reducing the ring size to five or four reduced the potency of the compounds. Our studies identified **11i** (**SLF80821178**) as the most potent (IC_50_: 51 ± 3 nM) Spns2 reported to date. **11i** contains a 4-decylphenyl urea linked to a piperazine moiety. Analysis of **11i** analogs suggest that (i) a positively charged cyclic secondary amine with hydrogen bond donor character is important for inhibiting S1P transport as methylation completely abrogated activity, (ii) the binding site for these inhibitors is sensitive to ring size as deviation from the 6-membered piperazine ring was detrimental to potency, and (iii) the lipid tail is sensitive to alkyl chain length wherein a decyl group is most optimal. *In silico* docking of **11i** into the recently published occluded inward-facing state cryo-EM structure suggests key hydrogen bonds between the terminal piperazine *N* to Ser211 and urea carbonyl to Asp112 with additional π stacking contribution with Phe234.

An important aspect of inhibitor design is the ability to demonstrate target engagement *in vivo*. When inhibitor **11i** was orally administered to mice at 10 mg/kg, compound exposure was observed as high as 1.2 μM at the 4-h *t*_max_. The orally bioavailable **11i** is substantial because this is the first example of an Spns2 inhibitor that is orally bioavailable. Indeed, when lymphocyte counts were measured at 4 h, a maximum level of lymphopenia (∼50%) was observed, which is similar to Spns2 knock out mice. In addition, no change in the plasma S1P level was observed. These parameters suggest *in vivo* target engagement of Spns2. Taken together, our studies identified **11i** as a potential *in vivo* chemical tool to investigate the physiological function of Spns2 and presents a scaffold for future development. Future work will be aimed at continuing the SAR study and investigating the biological and therapeutic implication of inhibition Spns2.

## Experimental Section

### General Materials and Synthetic Procedures

Reactions were performed using the Schlenk technique under an argon or nitrogen atmosphere, unless otherwise specified. All glassware used was flame-dried or oven-dried overnight. All materials utilized for the completion of this work were purchased from commercial vendors without further purification unless indicated otherwise. Anhydrous solvents were obtained from an Inert PureSolv MD5 system. Purification via column chromatography was performed on a Teledyne Isco CombiFlash Rf 200 machine with a SiliaFlash P60 40–63 μm (60 Å) stationary phase. Thin-layer chromatography (TLC) analyses were performed using Silicycle aluminum-backed silica gel F-254 plates. NMR analysis was conducted in a Bruker Advance III 600 MHz, Bruker Advance II 500 MHz, or Agilent 400-MR 400 MHz spectrometer. Chemical shifts follow standard nomenclature. Data are reported as follows: chemical shift, multiplicity (s = singlet, d = doublet, t = triplet, q = quartet, dd = doublet of doublets, dt = doublet of triplets, m = multiplet), coupling constants (Hz), and integration. Minor rotamer peaks are denoted by an asterisks (*). Determination of molecular mass was conducted using ESI (positive ionization) on an Agilent 6220 TOF mass spectrometer. Purity assessment of compound was performed by Waters UPLC analysis. UPLC conditions: Conditions for LC: Purity assessments were performed by Waters UPLC analysis. UPLC conditions: Solvent A: Water (0.1% TFA); solvent B: acetonitrile (0.1% TFA); column: Acquity BEH C18 1.7 μm 2.1 × 50 mm; method: isocratic 95% A, 5% B from 0 to 3.40 min then linear gradient from 5 to 95% B by 5.10 min, return to 5% B by 5.95 min, then hold for 2 min at 95% A, 5% B; UV wavelength = 254 nm; flow rate: 0.613 mL/min. All final materials synthesized had a purity ≥95% as measured by UPLC unless otherwise noted.

### HeLa Cell S1P Release Assay

This assay was conducted as reported by Kharel et al.^43^ Briefly, compounds were assessed in a HeLa cell assay at a 1 μM test concentration, unless noted otherwise. HeLa cells were transfected with a pcDNA3.1 plasmid encoding mouse Spns2, and pools of cells resistant to G418 were selected. S1P catabolic pathways were suppressed by the addition of 4-deoxypyridoxine (1 mM), NaF (2 mM), and Na_3_VO_4_ (0.2 mM). After growing cells to near confluence on 12-well tissue culture dishes, growth media was removed, inhibitors were introduced in release media (serum free medium containing 0.2% fatty acid free BSA), and the cells were incubated for 16–18 h at 37 °C. The BSA with bound S1P was concentrated from the release media by TFA-driven precipitation after addition of the *d7*-S1P internal standard, the S1P extracted from the protein pellet, and quantified by LC-MS/MS. Lower levels of S1P release into the media indicated greater degrees of Spns2 inhibition. Inhibition is reported as the percent decrease in S1P release relative to the control where no inhibitor was introduced.

### Molecular Docking

Schrödinger Maestro version 12.4 workspace^56^ (1) was used to run docking studies with inhibitors **11i**, (*R*)-**11l**, and (*R*)-**11c**. These inhibitors were docked to the most prominent cluster from simulations involving an AlphaFold2 generated SPNS2 homology model that was simulated in the presence of a membrane for 1 μs using MD simulations.^49,50,56^ The cluster structure from MD simulation (available on our Open Science Framework - https://osf.io/82n73/) was preprocessed using the protein preparation wizard to assign bond orders, add any missing hydrogens, and create disulfide bonds between sulfur atoms in proximity. Energy minimization was not performed in order to maintain the structure from MD. Inhibitors were processed using Ligprep from Epik to generate possible ionization states at pH 7, and the OPLS3e force field was used.^57,58^ The receptor grid generation tool from Glide was used to create the glide grid file containing box information.^59^ The box was centered around the protein at 2, 0, −4, and the box sized large enough to encompass much of the transport channel, only cutting off the intracellular and extracellular loops (40 Å × 40 Å × 40 Å). The box size is determined with two parameters—the size of the center box and the length of ligand allowed to dock outside of that box. The allowed length of the ligand outside the central box was set to 20 Å, and the center box size was set to 20 Å × 20 Å × 20 Å. This ensured that at least part of the inhibitor is within the center box, but parts of the ligand can dock 20 Å outside of that center box. Ligand docking with standard precision was performed in Glide to obtain up to 9 poses for each inhibitor.^59^ Interaction fingerprints were obtained from Discovery Informatics and QSAR.^60^ Prime was used to calculate predicted free energy of binding using molecular mechanics with generalized born and surface area solvation (MM/GBSA). In prime, the docked complexes were minimized utilizing a local optimization feature. The solvation model applied was the variable dielectric surface general born (VSGB) and the force field employed OPLS3e.^58,61^

### In Vivo Biological Evaluation

Compounds that performed well in the HeLa cell assay were subsequently administered to mice (C57BL/6j strain). Inhibitor (10 mg/kg) or an equivalent dose of vehicle (36.1% PEG400/9.1% ethanol/4.6% solutol/50% H_2_O) was administered to animals via an intraperitoneal injection or oral gavage. After 6 to 16 h, blood samples were collected. Lymphocyte counts were obtained from 20 μL of mouse blood using a Heska HT5 Element blood analyzer. Plasma S1P concentrations were determined using LC-MS/MS. All animal protocols were approved prior to experimentation by the University of Virginia School of Medicine’s Animal Care and Use Committee.

### General Procedure 1: HCTU Amide Coupling

To a round-bottom flask containing a stir bar was added 4-iodobenzoic acid (1.0 equiv), DIEA (3.0 equiv), and DCM. The mixture was allowed to stir at room temperature for 15 min, and HCTU (1.2 equiv) and a mono-*N*-Boc-protected diamine (1.0 equiv) were added. The solution was stirred for 16 h at room temperature at which point complete consumption of the starting material was observed. Upon completion, the reaction mixture was concentrated under reduced pressure to afford a viscous oil, which could be subjected to silica gel chromatography with appropriate ethyl acetate in the hexane solvent system to afford the pure product.

### General Procedure 2: One-Pot Hydroboration–Suzuki Miyaura Cross-Coupling

To an oven-dried two-neck round-bottom flask containing 1-decene (1.1 equiv) in THF was added 9-BBN (1.5 equiv, 0.5 M in THF). A condenser was attached, and the reaction mixture was heated to reflux for 2 h. Following hydroboration, the mixture was cooled to room temperature, and the appropriate aryl iodide (1.0 equiv) and Pd(dppf)Cl_2_·CH_2_Cl_2_ (5 mol %) were added. A solution of 3 M KOH-aq (3.0 equiv) was then slowly syringed into the reaction flask. The resulting mixture was again heated to reflux until complete consumption of the aryl iodide was observed as monitored by TLC (approximately 4 h). Once completion, the mixture was cooled to room temperature, filtered over Celite, and concentrated under reduced pressure to afford a crude oil. This oil was subjected to silica gel chromatography with an appropriate ethyl acetate in the hexane solvent system to yield the desired purified material.

### General Procedure 3: HCl-Assisted Boc Deprotection

To a 6-dram vial containing an *N-*Boc-protected amine (1.0 equiv) dissolved in DCM was added HCl (10.0 equiv, 4 M in dioxane). The resulting mixture was allowed to stir until the complete consumption of starting material was observed as monitored by TLC (approximately 2 h). The solvent was removed under reduced pressure, and the residue was rinsed with diethyl ether until a thick white precipitate formed. This precipitate was subjected to trituration with an appropriate solvent system to afford the pure product as a hydrochloride salt.

### General Procedure 4: Carbamate Formation

To a dried round-bottom flask charged with a stir bar was added 4-iodophenyl isocyanate (1.0 equiv) dissolved in anhydrous DCM. The flask was purged with N_2_ and placed in an ice bath for 20 min. Anhydrous DIEA (2.0 equiv) and a 0.2 M solution of an *N*-Boc-protected amino alcohol (1.0 equiv) dissolved in DCM were syringed into the sealed flask. The mixture was allowed to stir for 16 h while slowly warming to room temperature. Following reaction completion as monitored by TLC, the solvent was removed under reduced pressure, and the resulting residue was subjected to silica gel chromatography with an appropriate ethyl acetate in hexanes eluent to afford the desired phenylcarbamate product.

### General Procedure 5: Urea Formation

To an oven-dried round-bottom flask was added 4-iodophenyl isocyanate (1.0 equiv) and anhydrous DCM. The flask was purged with N_2_ and placed in an ice bath for 20 min. A 0.2 M solution of a mono-*N*-Boc-protected diamine (1.0 equiv) in anhydrous DCM was syringed into the flask, and the reaction was stirred for 16 h while slowly warming to room temperature. Following reaction completion as monitored by TLC, the solvent was removed under reduced pressure to afford an off-white solid. The material was loaded onto Celite and subjected to silica gel chromatography with an appropriate ethyl acetate in hexanes eluent to afford the desired product.

### General Procedure 6: Methylations of Amide and Urea Nitrogen

A round-bottom flask containing a stir bar was flame-dried and placed in a desiccator to cool. NaH (1.1 equiv, 60% dispersion in mineral oil) was added, and the flask was purged with N_2_. Anhydrous THF was syringed into the flask, and the solution was placed in an ice bath. A 0.2 M solution of either phenylurea (1.0 equiv) or benzamide (1.0 equiv) in THF was prepared and added dropwise to the reaction flask. The reaction stirred for 30 min before MeI (1.2 equiv) was added. The mixture was allowed to slowly warm to room temperature overnight. The solvent was then removed under reduced pressure to afford a crude oil, which could be subjected to silica gel chromatography with an appropriate ethyl acetate in hexanes eluent to afford the desired methylated material.

### General Procedure 7: Thionation of Benzamides

To a round-bottom flask containing a solution of benzamide (1.0 equiv) in THF was added Lawesson’s reagent (1.05 equiv). The mixture was allowed to stir at room temperature for 16 h or until TLC indicated reaction completion. The resulting dispersion was concentrated under reduced pressure and subjected to column chromatography in an appropriate ethyl acetate/hexanes solvent system to afford the desired purified product.

### Characterizations

#### *tert*-Butyl (3-(4-Iodobenzamido)propyl)carbamate (**2a**)

Synthesized according to General Procedure 1. Purified via column chromatography (55% ethyl acetate/hexanes). Off-white solid (97%, 394 mg). ^1^H NMR (400 MHz, CDCl_3_) δ 7.78 (d, *J* = 8.3 Hz, 2H), 7.58 (d, *J* = 8.2 Hz, 2H), 7.45 (brs, 1H), 4.91 (t, *J* = 7.1 Hz, 1H), 3.48 (q, *J* = 6.2 Hz, 2H), 3.23 (q, *J* = 6.4 Hz, 2H), 1.69 (p, *J* = 6.0 Hz, 2H), 1.44 (s, 9H). ^13^C NMR (101 MHz, CDCl_3_) δ 166.7, 157.3, 137.8, 134.1, 128.8, 98.4, 79.8, 37.1, 36.1, 30.3, 28.5. HRMS: (ESI) [M + H]^+^ calcd for C_15_H_22_IN_2_O_3_, 405.0670, observed, 405.0673.

#### *tert*-Butyl 3-(4-Iodobenzamido)azetidine-1-carboxylate (**2b**)

Synthesized according to General Procedure 1. Purified via column chromatography (30–40% ethyl acetate/hexanes). White solid (96%, 624 mg). ^1^H NMR (400 MHz, CDCl_3_) δ 7.94 (d, *J* = 7.1 Hz, 1H), 7.66 (d, *J* = 8.0 Hz, 2H), 7.50 (d, *J* = 8.1 Hz, 2H), 4.69 (h, *J* = 6.7 Hz, 1H), 4.19 (t, *J* = 8.5 Hz, 2H), 3.87–3.79 (m, 2H), 1.36 (s, 9H). ^13^C NMR (101 MHz, CDCl_3_) δ 166.9, 156.1, 137.6, 133.1, 128.9, 98.8, 79.9, 56.2, 39.9, 28.4. HRMS: (ESI) [M + H]^+^ calcd for C_15_H_20_IN_2_O_3_, 403.0513, observed, 403.0516.

#### *tert*-Butyl (R)-3-(4-Iodobenzamido)pyrrolidine-1-carboxylate (**2c**)

Synthesized according to General Procedure 1. Purified via column chromatography (50% ethyl acetate/hexanes). Clear oil (95%, 480 mg). ^1^H NMR (400 MHz, CDCl_3_) δ 7.67 (d, *J* = 8.5 Hz, 2H), 7.46 (d, *J* = 8.0 Hz, 2H), 7.04 (d, *J* = 57.1 Hz, 1H), 4.54 (h, *J* = 6.3 Hz, 1H), 3.69–3.53 (m, 1H), 3.49–3.17 (m, 3H), 2.20–2.08 (m, 1H), 2.00–1.83 (m, 1H), 1.40 (s, 9H). ^13^C NMR (101 MHz, CDCl_3_) δ 166.9, 154.5, 137.6, 133.6, 128.8, 98.6, 79.7, 51.7, 50.8*, 50.1, 49.5*, 44.3*, 43.9, 32.0, 30.9*, 28.5. Material isolated as an approximately 1:1 ratio of rotamers. HRMS: (ESI) [M + Na]^+^ calcd for C_16_H_21_IN_2_NaO_3_, 439.0489, observed, 439.0498.

#### *tert*-Butyl (S)-3-(4-Iodobenzamido)pyrrolidine-1-carboxylate (**2d**)

Synthesized according to General Procedure 1. Purified via column chromatography (50% ethyl acetate/hexanes). White solid (99%, 838 mg). ^1^H NMR (400 MHz, CDCl_3_) δ 7.62 (d, *J* = 8.4 Hz, 2H), 7.43 (d, *J* = 8.0 Hz, 2H), 7.27 (d, *J* = 44.6 Hz, 1H), 4.49 (h, *J* = 6.1 Hz, 1H), 3.65–3.48 (m, 1H), 3.45–3.14 (m, 3H), 2.17–2.04 (m, 1H), 1.95–1.81 (m, 1H), 1.36 (s, 9H). HRMS: (ESI) [M + Na]+ calcd for C_16_H_21_IN_2_NaO_3_, 439.0489, observed, 439.0499.

#### *tert*-Butyl (R)-3-(4-Iodobenzamido)piperidine-1-carboxylate (**2e**)

Synthesized according to General Procedure 1. Purified via column chromatography (40% ethyl acetate/hexanes). White solid (94%, 408 mg). ^1^H NMR (400 MHz, CDCl_3_) δ 7.71 (d, *J* = 8.5 Hz, 2H), 7.44 (d, *J* = 8.6 Hz, 2H), 6.55 (d, *J* = 60.8 Hz, 1H), 4.12–4.01 (m, 1H), 3.63–3.22 (m, 4H), 1.88–1.52 (m, 4H), 1.42 (s, 9H). ^13^C NMR (101 MHz, CDCl_3_) δ 166.2, 155.4, 137.7, 134.0, 128.6, 98.5, 80.2, 48.5, 46.2, 43.9, 29.4, 28.5, 22.4. HRMS: (ESI) [M + H]^+^ calcd for C_17_H_23_IN_2_NaO_3_, 453.0646, observed, 453.0647.

#### *tert*-Butyl (S)-3-(4-Iodobenzamido)piperidine-1-carboxylate (**2f**)

Synthesized according to General Procedure 1. Purified via column chromatography (40% ethyl acetate/hexanes). White solid (95%, 512 mg). ^1^H NMR (400 MHz, CDCl_3_) δ 7.64 (d, *J* = 8.5 Hz, 2H), 7.39 (d, *J* = 8.5 Hz, 2H), 6.69 (brs, 1H), 4.05–3.93 (m, 1H), 3.43 (d, *J* = 88.9 Hz, 4H), 1.88–1.54 (m, 4H), 1.37 (s, 9H). ^13^C NMR (101 MHz, CDCl_3_) δ 166.2, 155.4, 137.6, 133.9, 128.6, 98.4, 80.0, 48.3, 46.2, 44.4, 29.4, 28.4, 22.5. HRMS: (ESI) [M + Na]^+^ calcd for C_17_H_23_IN_2_NaO_3_, 453.0646, observed, 453.0649.

#### *tert*-Butyl 4-(4-Iodobenzamido)piperidine-1-carboxylate (**2g**)

Synthesized according to General Procedure 1. Purified via column chromatography (40% ethyl acetate/hexanes). White solid (92%, 799 mg). ^1^H NMR (400 MHz, CD_3_OD) δ 7.82 (d, *J* = 8.6 Hz, 2H), 7.56 (d, *J* = 8.6 Hz, 2H), 4.16–3.95 (m, 3H), 2.98–2.83 (m, 2H), 2.01–1.84 (m, 2H), 1.56–1.41 (m, 11H). ^13^C NMR (101 MHz, CD_3_OD) δ 168.8, 156.4, 138.8, 135.3, 130.1, 99.1, 81.1, 48.8, 44.0, 32.5, 28.7. HRMS: (ESI) [M + Na]^+^ calcd for C_17_H_23_IN_2_NaO_3_, 453.0646, observed, 453.0645.

#### *tert*-Butyl 4-(4-Iodobenzoyl)piperazine-1-carboxylate (**2h**)

Synthesized according to General Procedure 1. Purified via column chromatography (40–50% ethyl acetate/hexanes). White solid (93%, 780 mg). ^1^H NMR (400 MHz, CD_3_OD) δ 7.84 (d, *J* = 8.4 Hz, 2H), 7.21 (d, *J* = 8.4 Hz, 2H), 3.79–3.35 (m, 8H), 1.47 (s, 9H). ^13^C NMR (101 MHz, CD_3_OD) δ 171.7, 156.2, 139.0, 136.0, 130.0, 97.1, 81.7, 44.7, 43.3, 28.6. HRMS: (ESI) [M + H]^+^ calcd for C_16_H_22_IN_2_O_3_, 417.0670, observed, 417.0672.

#### *tert*-Butyl (S)-4-(4-Iodobenzoyl)-2-methylpiperazine-1-carboxylate (**2i**)

Synthesized according to General Procedure 1. Purified via column chromatography (40% ethyl acetate/hexanes). White solid (95%, 412 mg). ^1^H NMR (400 MHz, CDCl_3_) δ 7.72 (d, *J* = 8.4 Hz, 2H), 7.10 (d, *J* = 8.3 Hz, 2H), 4.59–4.06 (m, 2H), 3.95–2.79 (m, 5H), 1.42 (s, 9H), 1.22–0.99 (m, 3H). ^13^C NMR (101 MHz, CDCl_3_) δ 170.3, 154.4, 137.8, 134.9, 128.8, 96.1, 80.2, 51.5, 46.9, 42.1, 38.1, 28.4, 15.1. HRMS: (ESI) [M + H]^+^ calcd for C_17_H_24_IN_2_O_3_, 431.0826, observed, 431.0840.

#### *tert*-Butyl 4-(4-Iodobenzoyl)-3-methylpiperazine-1-carboxylate (**2j**)

Synthesized according to General Procedure 1. Purified via column chromatography (40% ethyl acetate/hexanes). White solid (99%, 431 mg). ^1^H NMR (400 MHz, CDCl_3_) δ 7.73 (d, *J* = 8.3 Hz, 2H), 7.08 (d, *J* = 8.3 Hz, 2H), 4.91–3.63 (m, 4H), 3.28–2.69 (m, 3H), 1.43 (s, 9H), 1.21 (d, *J* = 6.8 Hz, 3H). ^13^C NMR (126 MHz, CDCl_3_) δ 169.7, 155.0, 137.8, 135.4, 128.4, 95.9, 80.3, 50.5, 48.6, 47.3*, 44.2*, 43.2, 36.9, 28.4, 15.6. Material isolated as an approximately 5:4 ratio of rotamers. HRMS: (ESI) [M + H]^+^ calcd for C_17_H_24_IN_2_O_3_, 431.0826, observed, 431.3267.

#### *tert*-Butyl 3-(4-Iodobenzoyl)-3,8-diazabicyclo[3.2.1]octane-8-carboxylate (**2k**)

Synthesized according to General Procedure 1. Purified via column chromatography (35% ethyl acetate/hexanes). White solid (90%, 482 mg). ^1^H NMR (400 MHz, CDCl_3_) δ 7.76 (d, *J* = 8.4 Hz, 2H), 7.21 (d, *J* = 8.4 Hz, 2H), 4.90–4.67 (m, 1H), 4.14–3.68 (m, 3H), 3.30–2.85 (m, 2H), 2.02–1.71 (m, 4H), 1.45 (s, 9H). ^13^C NMR (101 MHz, CDCl_3_) δ 167.6, 155.8, 137.8, 135.2, 129.1, 96.8, 80.4, 56.6, 51.4*, 50.0, 48.9*, 28.5, 27.7, 26.2*. Material isolated as an approximately 1:1 ratio of rotamers. HRMS: (ESI) [M + H]^+^ calcd for C_18_H_24_IN_2_O_3_, 443.0826, observed, 443.0832.

#### *tert*-Butyl (3-(4-Decylbenzamido)propyl)carbamate (**3a**)

Synthesized according to General Procedure 2. Purified via column chromatography (40% ethyl acetate/hexanes). Off-white solid (87%, 354 mg). ^1^H NMR (400 MHz, CDCl_3_) δ 7.75 (d, *J* = 8.2 Hz, 2H), 7.28–7.18 (m, 3H), 5.07 (t, *J* = 6.9 Hz, 1H), 3.48 (q, *J* = 6.2 Hz, 2H), 3.21 (q, *J* = 6.5 Hz, 2H), 2.62 (t, *J* = 7.7 Hz, 2H), 1.68 (p, *J* = 6.0 Hz, 2H), 1.60 (p, *J* = 7.6 Hz, 2H), 1.44 (s, 9H), 1.37–1.15 (m, 14H), 0.87 (t, *J* = 7.0 Hz, 3H). ^13^C NMR (101 MHz, CDCl_3_) δ 167.7, 157.0, 146.8, 132.0, 128.6, 127.1, 79.5, 37.2, 36.2, 35.9, 32.0, 31.3, 30.4, 29.7, 29.7, 29.6, 29.4, 29.3, 28.5, 22.8, 14.2. HRMS: (ESI) [M + Na]^+^ calcd for C_25_H_42_N_2_NaO_3_, 441.3088, observed, 441.3080.

#### *tert*-Butyl 3-(4-Decylbenzamido)azetidine-1-carboxylate (**3b**)

Synthesized according to General Procedure 2. Purified via column chromatography (30–40% ethyl acetate/hexanes). White solid (91%, 589 mg). ^1^H NMR (400 MHz, CDCl_3_) δ 7.72 (d, *J* = 8.3 Hz, 2H), 7.57 (d, *J* = 7.1 Hz, 1H), 7.15 (d, *J* = 8.3 Hz, 2H), 4.81–4.69 (m, 1H), 4.23 (t, *J* = 8.5 Hz, 2H), 3.86 (dd, *J* = 9.3, 5.1 Hz, 2H), 2.59 (t, *J* = 7.7 Hz, 2H), 1.57 (p, *J* = 6.5 Hz, 2H), 1.39 (s, 9H), 1.32–1.16 (m, 14H), 0.85 (t, *J* = 6.5 Hz, 3H). ^13^C NMR (101 MHz, CDCl_3_) δ 167.6, 156.2, 147.1, 131.1, 128.5, 127.3, 79.7, 56.6, 39.8, 35.9, 31.9, 31.2, 29.6, 29.6, 29.5, 29.3, 29.3, 28.4, 22.7, 14.1. HRMS: (ESI) [M + H]^+^ calcd for C_25_H_41_N_2_O_3_, 417.3112, observed, 417.3122.

#### *tert*-Butyl (R)-3-(4-Decylbenzamido)pyrrolidine-1-carboxylate (**3c**)

Synthesized according to General Procedure 2. Purified via column chromatography (50% ethyl acetate/hexanes). White solid (98%, 490 mg). ^1^H NMR (400 MHz, CDCl_3_) δ 7.65 (d, *J* = 8.2 Hz, 2H), 7.16 (d, *J* = 8.1 Hz, 2H), 6.67 (d, *J* = 7.1 Hz, 1H), 4.58 (h, *J* = 5.3 Hz, 1H), 3.71–3.59 (m, 1H), 3.51–3.18 (m, 3H), 2.59 (t, *J* = 7.7 Hz, 2H), 2.22–2.09 (m, 1H), 1.96–1.86 (m, 1H), 1.56 (p, *J* = 7.1 Hz, 2H), 1.42 (s, 9H), 1.33–1.15 (m, 14H), 0.84 (t, *J* = 6.7 Hz, 3H). ^13^C NMR (101 MHz, CDCl_3_) δ 167.6, 154.6, 147.0, 131.5, 128.5, 127.1, 79.6, 51.8, 50.9*, 49.9, 49.3*, 44.2*, 43.9, 35.8, 32.0, 31.9, 31.3, 30.9*, 29.6, 29.6, 29.5, 29.3, 29.3, 28.5, 22.7, 14.1. NMR shows slight residual ethyl acetate impurity peak. Material isolated as an approximately 1:1 ratio of rotamers. HRMS: (ESI) [M + H]^+^ calcd for C_26_H_43_N_2_O_3_, 431.3268, observed, 431.3267.

#### *tert*-Butyl (S)-3-(4-Decylbenzamido)pyrrolidine-1-carboxylate (**3d**)

Synthesized according to General Procedure 2. Purified via column chromatography (40% ethyl acetate/hexanes). White solid (80%, 691 mg). ^1^H NMR (400 MHz, CDCl_3_) δ 7.65 (d, *J* = 8.1 Hz, 2H), 7.15 (d, *J* = 8.0 Hz, 2H), 6.73 (d, *J* = 7.1 Hz, 1H), 4.57 (h, *J* = 6.1 Hz, 1H), 3.72–3.58 (m, 1H), 3.50–3.17 (m, 3H), 2.58 (t, *J* = 7.7 Hz, 2H), 2.21–2.08 (m, 1H), 1.96–1.84 (m, 1H), 1.56 (p, *J* = 7.2 Hz, 2H), 1.41 (s, 9H), 1.30–1.14 (m, 14H), 0.84 (t, *J* = 6.7 Hz, 3H). ^13^C NMR (101 MHz, CDCl_3_) δ 167.6, 154.5, 147.0, 131.5, 128.5, 127.1, 79.5, 51.7, 50.9*, 49.9, 49.3*, 44.3*, 43.9, 35.8, 32.0*, 31.9, 31.2, 30.9, 29.6, 29.6, 29.5, 29.3, 29.2, 28.5, 22.7, 14.1. Material isolated as an approximately 1:1 ratio of rotamers. NMR shows slight residual ethyl acetate impurity peak. HRMS: (ESI) [M + H]^+^ calcd for C_26_H_43_N_2_O_3_, 431.3268, observed, 431.3269.

#### *tert*-Butyl (R)-3-(4-Decylbenzamido)piperidine-1-carboxylate (**3e**)

Synthesized according to General Procedure 2. Purified via column chromatography (30–40% ethyl acetate/hexanes). Off-white solid (90%, 379 mg). ^1^H NMR (400 MHz, CDCl_3_) δ 7.65 (d, *J* = 8.2 Hz, 2H), 7.18 (d, *J* = 8.2 Hz, 2H), 6.40 (brs, 1H), 4.16–4.05 (m, 1H), 3.62–3.21 (m, 4H), 2.60 (t, *J* = 7.7 Hz, 2H), 1.85–1.51 (m, 6H), 1.43 (s, 9H), 1.32–1.17 (m, 14H), 0.85 (t, *J* = 6.9 Hz, 3H). ^13^C NMR (101 MHz, CDCl_3_) δ 167.0, 155.5, 146.9, 132.0, 128.6, 127.0, 80.0, 48.6, 45.8, 43.8, 35.9, 31.9, 31.3, 29.7, 29.6, 29.5, 29.4, 29.3, 28.4, 22.7, 22.4, 14.2. HRMS: (ESI) [M + H]^+^ calcd for C_27_H_45_N_2_O_3_, 445.3425, observed, 445.3427.

#### *tert*-Butyl (S)-3-(4-Decylbenzamido)piperidine-1-carboxylate (**3f**)

Synthesized according to General Procedure 2. Purified via column chromatography (30–40% ethyl acetate/hexanes). Off-white solid (83%, 439 mg). ^1^H NMR (500 MHz, CDCl_3_) δ 7.64 (d, *J* = 8.2 Hz, 2H), 7.16 (d, *J* = 8.2 Hz, 2H), 6.54 (d, *J* = 91.1 Hz, 1H), 4.13–4.04 (m, 1H), 3.66–3.22 (m, 4H), 2.59 (t, *J* = 7.7 Hz, 2H), 1.86–1.48 (m, 6H), 1.41 (s, 9H), 1.29–1.18 (m, 14H), 0.84 (t, *J* = 6.9 Hz, 3H). ^13^C NMR (126 MHz, CDCl_3_) δ 167.0, 155.1, 146.8, 131.9, 128.5, 127.0, 80.0, 48.7, 45.7, 43.6, 35.8, 31.9, 31.2, 29.6, 29.6, 29.5, 29.5, 29.3, 29.2, 28.4, 22.7, 22.4, 14.1. HRMS: (ESI) [M + Na]^+^ calcd for C_27_H_44_N_2_NaO_3_, 467.3244, observed, 467.3247.

#### *tert*-Butyl 4-(4-Decylbenzamido)piperidine-1-carboxylate (**3g**)

Synthesized according to General Procedure 2. Purified via column chromatography (30% ethyl acetate/hexanes). Light brown solid (87%, 721 mg). ^1^H NMR (400 MHz, CDCl_3_) δ 7.65 (d, *J* = 8.2 Hz, 2H), 7.13 (d, *J* = 8.2 Hz, 2H), 6.59 (d, *J* = 7.9 Hz, 1H), 4.11–3.97 (m, 3H), 2.79 (t, *J* = 12.8 Hz, 2H), 2.57 (t, *J* = 7.7 Hz, 2H), 1.94–1.86 (m, 2H), 1.54 (p, *J* = 7.0 Hz, 2H), 1.41–1.37 (m, 11H), 1.29–1.13 (m, 14H), 0.83 (t, *J* = 6.9 Hz, 3H). ^13^C NMR (101 MHz, CDCl_3_) δ 166.9, 154.6, 146.7, 131.9, 128.4, 127.1, 79.5, 47.1, 42.8, 35.8, 32.0, 31.9, 31.2, 29.6, 29.5, 29.4, 29.3, 29.2, 28.4, 22.6, 14.1. HRMS: (ESI) [M + H]^+^ calcd for C_27_H_45_N_2_O_3_, 445.3425, observed, 445.3425.

#### *tert*-Butyl 4-(4-Decylbenzoyl)piperazine-1-carboxylate (**3h**)

Synthesized according to General Procedure 2. Purified via column chromatography (40% ethyl acetate/hexanes). Off-white solid (92%, 740 mg). ^1^H NMR (400 MHz, CDCl_3_) δ 7.30 (d, *J* = 7.8 Hz, 2H), 7.20 (d, *J* = 7.8 Hz, 2H), 3.81–3.29 (m, 8H), 2.61 (t, *J* = 7.7 Hz, 2H), 1.60 (p, *J* = 7.1 Hz, 2H), 1.46 (s, 9H), 1.35–1.20 (m, 14H), 0.87 (t, *J* = 6.7 Hz, 3H). ^13^C NMR (101 MHz, CDCl_3_) δ 171.0, 154.7, 145.3, 132.8, 128.7, 127.3, 80.4, 47.6*, 43.7, 42.3, 35.9, 32.0, 31.4, 29.7, 29.7, 29.6, 29.4, 29.4, 28.5, 22.8, 14.2. Material isolated as an approximately 2:1 ratio of rotamers. HRMS: (ESI) [M + H]^+^ calcd for C_26_H_43_N_2_O_3_, 431.3268, observed, 431.3267.

#### *tert*-Butyl (S)-4-(4-Decylbenzoyl)-2-methylpiperazine-1-carboxylate (**3i**)

Synthesized according to General Procedure 2. Purified via column chromatography (30–40% ethyl acetate/hexanes). Brown oil (80%, 339 mg). ^1^H NMR (400 MHz, CDCl_3_) δ 7.26 (d, *J* = 8.0 Hz, 2H), 7.15 (d, *J* = 8.1 Hz, 2H), 4.58–3.76 (m, 4H), 3.31–2.82 (m, 3H), 2.57 (t, *J* = 7.7 Hz, 2H), 1.58 (p, *J* = 7.2 Hz, 2H), 1.41 (s, 9H), 1.30–1.17 (m, 14H), 0.83 (t, *J* = 6.8 Hz, 3H). ^13^C NMR (101 MHz, CDCl_3_) δ 171.6, 154.5, 145.0, 132.8, 128.5, 127.2, 80.1, 51.6, 47.1, 41.9, 38.3, 35.8, 31.9, 31.3, 29.6, 29.6, 29.5, 29.3, 29.3, 28.4, 22.7, 15.2, 14.1. HRMS: (ESI) [M + H]^+^ calcd for C_27_H_45_N_2_O_3_, 445.3425, observed, 445.3428.

#### *tert*-Butyl 4-(4-Decylbenzoyl)-3-methylpiperazine-1-carboxylate (**3j**)

Synthesized according to General Procedure 2. Purified via column chromatography (35–60% ethyl acetate/hexanes). Off-white solid (78%, 348 mg). ^1^H NMR (400 MHz, CDCl_3_) δ 7.28 (d, *J* = 8.2 Hz, 2H), 7.21 (d, *J* = 8.1 Hz, 2H), 4.93–3.62 (m, 4H), 3.29–2.73 (m, 3H), 2.62 (t, *J* = 7.7 Hz, 2H), 1.61 (p, *J* = 7.5 Hz, 2H), 1.47 (s, 9H), 1.36–1.20 (m, 17H), 0.88 (t, *J* = 6.9 Hz, 3H). ^13^C NMR (126 MHz, CDCl_3_) δ 170.9, 155.1, 145.0, 133.3, 128.7, 126.8, 80.2, 50.0, 48.7, 47.4*, 44.8, 44.4*, 43.3, 35.9, 32.0, 31.4, 29.7, 29.6, 29.5, 29.4, 29.3, 28.4, 22.7, 15.5, 14.2. Material isolated as an approximately 1:1 ratio of rotamers. HRMS: (ESI) [M + H]^+^ calcd for C_27_H_44_N_2_NaO_3_, 467.3244, observed, 467.3250.

#### *tert*-Butyl 3-(4-Decylbenzoyl)-3,8-diazabicyclo[3.2.1]octane-8-carboxylate (**3k**)

Synthesized according to General Procedure 2. Purified via column chromatography (30–40% ethyl acetate/hexanes). Clear oil (74%, 367 mg). ^1^H NMR (400 MHz, CDCl_3_) δ 7.38 (d, *J* = 8.1 Hz, 2H), 7.19 (d, *J* = 8.0 Hz, 2H), 4.91–4.65 (m, 1H), 4.20–3.66 (m, 3H), 3.29–2.93 (m, 2H), 2.60 (t, *J* = 7.7 Hz, 2H), 1.99–1.68 (m, 4H), 1.59 (p, *J* = 7.3 Hz, 2H), 1.44 (s, 9H), 1.34–1.18 (m, 14H), 0.86 (t, *J* = 6.8 Hz, 3H). ^13^C NMR (101 MHz, CDCl_3_) δ 168.7, 155.9, 145.6, 133.0, 128.6, 127.5, 80.2, 56.5, 51.2*, 50.1, 49.0*, 35.9, 32.0, 31.3, 29.7, 29.6, 29.5, 29.4, 29.3, 28.4, 27.7, 26.1*, 22.7, 14.2. Material isolated as an approximately 1:1 ratio of rotamers. HRMS: (ESI) [2M+H]^+^ calcd for C_56_H_89_N_4_O_6_, 913.6777, observed, 913.6774.

#### *N*-(3-Aminopropyl)-4-decylbenzamide Hydrochloride (**4a**)

Synthesized according to General Procedure 3. Purified via trituration with ethyl acetate and diethyl ether. White solid (78%, 66 mg). ^1^H NMR (400 MHz, CD_3_OD) δ 7.77 (d, *J* = 8.2 Hz, 2H), 7.29 (d, *J* = 8.3 Hz, 2H), 3.50 (t, *J* = 6.6 Hz, 2H), 3.00 (t, *J* = 7.3 Hz, 2H), 2.67 (t, *J* = 7.6 Hz, 2H), 1.97 (p, *J* = 6.9 Hz, 2H), 1.63 (p, *J* = 7.6 Hz, 2H), 1.38–1.23 (m, 14H), 0.89 (t, *J* = 6.9 Hz, 3H). ^13^C NMR (101 MHz, CD_3_OD) δ 170.9, 148.6, 132.4, 129.6, 128.4, 38.3, 37.3, 36.7, 33.1, 32.4, 30.7, 30.7, 30.5, 30.4, 30.3, 28.9, 23.7, 14.4. HRMS: (ESI) [M + H]^+^ calcd for C_20_H_35_N_2_O, 319.2744, observed, 319.2752.

#### *N*-(Azetidin-3-yl)-4-decylbenzamide 2,2,2-Trifluoroacetate (**4b**)

*tert*-Butyl 3-(4-decylbenzamido)azetidine-1-carboxylate (**3b**) was dissolved in DCM and added to a 6-dram vial containing a stir bar. Trifluoroacetic acid (20.0 equiv) was added to the flask, and the mixture was allowed to stir at room temperature for 2 h. Following completion as monitored by TLC, the material was loaded onto Celite and subjected to silica gel chromatography. Purified via column chromatography (10–15% MeOH/DCM). White solid (67%, 69 mg). ^1^H NMR (400 MHz, CD_3_OD) δ 7.80 (d, *J* = 8.3 Hz, 2H), 7.46 (d, *J* = 8.4 Hz, 2H), 4.48 (tt, *J* = 7.6, 3.8 Hz, 1H), 4.14 (t, *J* = 11.5 Hz, 1H), 3.93 (dd, *J* = 11.4, 7.2 Hz, 1H), 3.80 (dd, *J* = 11.8, 3.7 Hz, 1H), 3.69 (dd, *J* = 11.8, 4.4 Hz, 1H), 2.74 (t, *J* = 7.7 Hz, 2H), 1.66 (p, *J* = 7.2 Hz, 2H), 1.38–1.20 (m, 14H), 0.89 (t, *J* = 6.9 Hz, 3H). ^13^C NMR (101 MHz, CD_3_OD) δ 167.3, 152.3, 130.7, 129.5, 121.1, 63.5, 60.5, 47.9, 36.9, 33.1, 32.2, 30.7, 30.7, 30.5, 30.4, 30.2, 23.7, 14.4. HRMS: (ESI) [M + H]^+^ calcd for C_20_H_33_N_2_O, 317.2587, observed, 317.2592.

#### (R)-4-Decyl-*N*-(pyrrolidin-3-yl)benzamide Hydrochloride (**4c**)

Synthesized according to General Procedure 3. Purified via trituration with ethyl acetate and diethyl ether. White solid (79%, 101 mg). ^1^H NMR (400 MHz, CD_3_OD) δ 7.80 (d, *J* = 8.3 Hz, 2H), 7.28 (d, *J* = 8.2 Hz, 2H), 4.63 (tt, *J* = 7.1, 5.0 Hz, 1H), 3.66–3.52 (m, 2H), 3.46–3.34 (m, 2H), 2.66 (t, *J* = 7.7 Hz, 2H), 2.47–2.34 (m, 1H), 2.27–2.15 (m, 1H), 1.63 (p, *J* = 7.5 Hz, 2H), 1.36–1.20 (m, 14H), 0.89 (t, *J* = 6.8 Hz, 3H). ^13^C NMR (101 MHz, CD_3_OD) δ 170.4, 148.7, 132.3, 129.6, 128.6, 51.1, 50.8, 45.8, 36.7, 33.1, 32.4, 30.9, 30.7, 30.7, 30.5, 30.4, 30.3, 23.7, 14.4. HRMS: (ESI) [M + H]^+^ calcd for C_21_H_35_N_2_O, 331.2744, observed, 331.2731.

#### (S)-4-Decyl-*N*-(pyrrolidin-3-yl)benzamide Hydrochloride (**4d**)

Synthesized according to General Procedure 3. Purified via trituration with ethyl acetate and diethyl ether. White solid (77%, 99 mg). ^1^H NMR (400 MHz, CD_3_OD) δ 7.80 (d, *J* = 8.3 Hz, 2H), 7.28 (d, *J* = 8.2 Hz, 2H), 4.63 (tt, *J* = 7.1, 5.0 Hz, 1H), 3.66–3.52 (m, 2H), 3.45–3.34 (m, 2H), 2.66 (t, *J* = 7.7 Hz, 2H), 2.48–2.34 (m, 1H), 2.29–2.14 (m, 1H), 1.63 (p, *J* = 7.5 Hz, 2H), 1.37–1.20 (m, 14H), 0.89 (t, *J* = 6.8 Hz, 3H). ^13^C NMR (101 MHz, CD_3_OD) δ 170.5, 148.7, 132.3, 129.6, 128.6, 51.2, 50.8, 45.8, 36.7, 33.0, 32.4, 30.9, 30.7, 30.7, 30.5, 30.4, 30.3, 23.7, 14.4. HRMS: (ESI) [M + H]^+^ calcd for C_21_H_35_N_2_O, 331.2744, observed, 331.2731.

#### (R)-4-Decyl-*N*-(piperidin-3-yl)benzamide Hydrochloride (**4e**)

Synthesized according to General Procedure 3. Purified via trituration with ethyl acetate and diethyl ether. White solid (67%, 69 mg). ^1^H NMR (400 MHz, CD_3_OD) δ 7.79 (d, *J* = 8.3 Hz, 2H), 7.28 (d, *J* = 8.3 Hz, 2H), 4.29 (tt, *J* = 10.5, 4.0 Hz, 1H), 3.50 (dd, *J* = 12.2, 4.3 Hz, 1H), 3.35 (dt, *J* = 12.9, 4.0 Hz, 1H), 3.07–2.95 (m, 2H), 2.66 (t, *J* = 7.7 Hz, 2H), 2.13–2.02 (m, 2H), 1.95–1.70 (m, 2H), 1.62 (p, *J* = 7.2 Hz, 2H), 1.38–1.21 (m, 14H), 0.88 (t, *J* = 6.8 Hz, 3H). ^13^C NMR (101 MHz, CD_3_OD) δ 169.9, 148.6, 132.4, 129.6, 128.6, 47.9, 45.5, 44.9, 36.7, 33.0, 32.4, 30.7, 30.7, 30.5, 30.4, 30.3, 29.1, 23.7, 22.3, 14.5. HRMS: (ESI) [M + H]^+^ calcd for C_22_H_37_N_2_O, 345.2900, observed, 345.2894.

#### (S)-4-Decyl-*N*-(piperidin-3-yl)benzamide Hydrochloride (**4f**)

Synthesized according to General Procedure 3. Purified via trituration with ethyl acetate and diethyl ether. White solid (67%, 69 mg). ^1^H NMR (400 MHz, CD_3_OD) δ 7.79 (d, *J* = 8.3 Hz, 2H), 7.27 (d, *J* = 8.3 Hz, 2H), 4.30 (tt, *J* = 10.4, 4.0 Hz, 1H), 3.50 (dd, *J* = 12.3, 4.4 Hz, 1H), 3.35 (dt, *J* = 12.9, 4.1 Hz, 1H), 3.07–2.96 (m, 2H), 2.65 (t, *J* = 7.6 Hz, 2H), 2.13–2.02 (m, 2H), 1.96–1.72 (m, 2H), 1.62 (p, *J* = 7.1 Hz, 2H), 1.36–1.21 (m, 14H), 0.89 (t, *J* = 6.9 Hz, 3H). ^13^C NMR (101 MHz, CD_3_OD) δ 169.9, 148.6, 132.4, 129.6, 128.6, 47.9, 45.5, 44.8, 36.7, 33.0, 32.4, 30.7, 30.7, 30.5, 30.4, 30.3, 29.1, 23.7, 22.3, 14.5. HRMS: (ESI) [M + H]^+^ calcd for C_22_H_37_N_2_O, 345.2900, observed, 345.2900.

#### 4-Decyl-*N*-(piperidin-4-yl)benzamide Hydrochloride (**4g**)

Synthesized according to General Procedure 3. Purified via trituration with ethyl acetate and diethyl ether. White solid (84%, 72 mg). ^1^H NMR (400 MHz, CD_3_OD) δ 8.44 (d, *J* = 7.4 Hz, 1H), 7.77 (d, *J* = 8.1 Hz, 2H), 7.27 (d, *J* = 8.4 Hz, 2H), 4.24–4.11 (m, 1H), 3.48 (dt, *J* = 14.1, 4.2 Hz, 2H), 3.15 (td, *J* = 12.8, 3.1 Hz, 2H), 2.65 (t, *J* = 7.7 Hz, 2H), 2.23–2.12 (m, 2H), 2.00–1.85 (m, 2H), 1.61 (p, *J* = 7.4 Hz, 2H), 1.37–1.20 (m, 14H), 0.89 (t, *J* = 6.8 Hz, 3H). ^13^C NMR (101 MHz, CD_3_OD) δ 169.9*, 169.8, 148.4, 132.8*, 132.8, 129.5, 128.5, 46.3*, 46.2, 44.4, 36.7, 33.0, 32.4, 30.7, 30.7, 30.5, 30.4, 30.3, 29.5*, 29.5, 23.7, 14.5. HRMS: (ESI) [M + H]^+^ calcd for C_22_H_37_N_2_O, 345.2900, observed, 345.2904.

#### (4-Decylphenyl)(piperazin-1-yl)methanone Hydrochloride (**4h**)

Synthesized according to General Procedure 3. Purified via trituration with ethyl acetate and diethyl ether. White solid (74%, 95 mg). ^1^H NMR (400 MHz, CD_3_OD) δ 7.43 (d, *J* = 8.1 Hz, 2H), 7.33 (d, *J* = 8.0 Hz, 2H), 3.88 (brs, 4H), 3.32 (brs, 4H), 2.68 (t, *J* = 7.7 Hz, 2H), 1.65 (p, *J* = 7.5 Hz, 2H), 1.40–1.23 (m, 14H), 0.91 (t, *J* = 6.8 Hz, 3H). ^13^C NMR (101 MHz, CD_3_OD) δ 172.9, 147.3, 132.7, 129.9, 128.5, 44.4, 36.7, 33.1, 32.5, 30.7, 30.7, 30.6, 30.5, 30.3, 23.7, 14.4. One piperazine carbon signal overlaps with CD_3_OD solvent peak. HRMS: (ESI) [M + H]^+^ calcd for C_21_H_35_N_2_O, 331.2744, observed, 331.2731.

#### (S)-(4-Decylphenyl)(3-methylpiperazin-1-yl)methanone Hydrochloride (**4i**)

Synthesized according to General Procedure 3. Purified via trituration with ethyl acetate and diethyl ether. White solid (91%, 78 mg). ^1^H NMR (500 MHz, CD_3_OD) δ 7.41 (d, *J* = 8.2 Hz, 2H), 7.31 (d, *J* = 8.2 Hz, 2H), 4.57 (brs, 1H), 3.98 (brs, 1H), 3.51–3.36 (m, 3H), 3.25–3.15 (m, 2H), 2.67 (t, *J* = 7.7 Hz, 2H), 1.63 (p, *J* = 7.3 Hz, 2H), 1.43–1.22 (m, 17H), 0.90 (t, *J* = 7.0 Hz, 3H). ^13^C NMR (126 MHz, CD_3_OD) δ 172.8, 147.3, 132.8, 129.9, 128.5, 52.4, 44.1, 36.8, 33.1, 32.5, 30.7, 30.7, 30.6, 30.5, 30.3, 23.7, 16.0, 14.5. HRMS: (ESI) [M + H]^+^ calcd for C_22_H_37_N_2_O, 345.2900, observed, 345.2907.

#### (4-Decylphenyl)(2-methylpiperazin-1-yl)methanone Hydrochloride (**4j**)

Synthesized according to General Procedure 3. Purified via trituration with ethyl acetate and diethyl ether. White solid (90%, 77 mg). ^1^H NMR (400 MHz, CD_3_OD) δ 7.41 (d, *J* = 8.2 Hz, 2H), 7.33 (d, *J* = 8.2 Hz, 2H), 4.79–4.65 (m, 1H), 4.22 (d, *J* = 12.2 Hz, 1H), 3.56–3.43 (m, 1H), 3.42–3.36 (m, 3H), 3.18 (td, *J* = 12.9, 3.9 Hz, 1H), 2.69 (t, *J* = 7.7 Hz, 2H), 1.65 (p, *J* = 7.2 Hz, 2H), 1.45 (d, *J* = 7.2 Hz, 3H), 1.40–1.26 (m, 14H), 0.92 (t, *J* = 6.9 Hz, 3H). ^13^C NMR (126 MHz, CD_3_OD) δ 173.1, 147.1, 133.2, 129.9, 128.1, 47.9, 46.6, 44.3, 37.9, 36.7, 33.1, 32.5, 30.7, 30.7, 30.6, 30.5, 30.3, 23.7, 15.4, 14.4. HRMS: (ESI) [M + H]^+^ calcd for C_22_H_37_N_2_O, 345.2900, observed, 345.2900.

#### (3,8-Diazabicyclo[3.2.1]octan-3-yl)(4-decylphenyl)methanone Hydrochloride (**4k**)

Synthesized according to General Procedure 3. Purified via trituration with ethyl acetate and diethyl ether. White solid (93%, 80 mg). ^1^H NMR (400 MHz, CD_3_OD) δ 7.50 (d, *J* = 8.1 Hz, 2H), 7.33 (d, *J* = 8.1 Hz, 2H), 4.80–4.19 (m, 2H), 3.43–3.26 (m, 4H), 2.67 (t, *J* = 7.7 Hz, 2H), 2.27–2.18 (m, 2H), 2.11–1.99 (m, 2H), 1.64 (p, *J* = 7.3 Hz, 2H), 1.42–1.22 (m, 14H), 0.90 (t, *J* = 6.9 Hz, 3H). ^13^C NMR (126 MHz, CD_3_OD) δ 171.0, 147.9, 132.9, 129.9, 128.7, 56.5, 51.4*, 49.9, 36.8, 33.1, 32.5, 30.7, 30.7, 30.6, 30.5, 30.3, 27.6, 26.3*, 23.7, 14.4. Material isolated as an approximately 1:1 ratio of rotamers. HRMS: (ESI) [M + H]^+^ calcd for C_23_H_37_N_2_O, 357.2900, observed, 357.2911.

#### (2,5-Diazabicyclo[2.2.1]heptan-2-yl)(4-decylphenyl)methanone Hydrochloride (**4l**)

Synthesized according to General Procedure 3. Purified via trituration with ethyl acetate and diethyl ether. White solid (65%, 56 mg). ^1^H NMR (400 MHz, CD_3_OD) δ 7.57–7.42 (m, 2H), 7.36–7.23 (m, 2H), 5.01–4.42 (m, 2H), 3.86–3.79 (m, 1H), 3.74–3.52 (m, 2H), 3.46–3.38 (m, 1H), 2.67 (t, *J* = 7.8 Hz, 2H), 2.25 (dd, *J* = 45.0, 11.8 Hz, 1H), 2.05 (dd, *J* = 34.3, 11.4 Hz, 1H)*, 1.63 (p, *J* = 6.7 Hz, 2H), 1.40–1.21 (m, 14H), 0.90 (t, *J* = 6.9 Hz, 3H). ^13^C NMR (101 MHz, CD_3_OD) δ 172.6, 171.3*, 147.9*, 147.6, 133.5, 133.3*, 129.9, 129.7*, 128.8*, 128.5, 59.9, 59.7*, 58.9, 56.3*, 54.1, 53.8*, 52.9, 50.4*, 37.4, 36.8, 35.8*, 33.0, 32.5, 30.7, 30.7, 30.6, 30.4, 30.3, 23.7, 14.5. Material isolated as an approximately 1:1 ratio of rotamers. HRMS: (ESI) [M + H]^+^ calcd for C_22_H_35_N_2_O, 343.2744, observed, 343.2738.

#### 2-((*tert*-Butoxycarbonyl)amino)ethyl (4-Iodophenyl)carbamate (**6a**)

Synthesized according to General Procedure 4. Purified via column chromatography (20–30% ethyl acetate/hexanes). White solid (67%, 223 mg). ^1^H NMR (400 MHz, CD_3_OD) δ 7.60 (d, *J* = 8.8 Hz, 2H), 7.27 (d, *J* = 8.3 Hz, 2H), 4.17 (t, *J* = 5.6 Hz, 2H), 3.36 (t, *J* = 5.6 Hz, 2H), 1.45 (s, 9H). ^13^C NMR (101 MHz, CD_3_OD) δ 158.4, 155.4, 140.2, 138.8, 121.7, 86.1, 80.2, 64.8, 40.7, 28.7. HRMS: (ESI) [M + Na]^+^ calcd for C_14_H_19_IN_2_NaO_4_, 429.0282, observed, 429.0293.

#### *tert*-Butyl (R)-3-(((4-iodophenyl)carbamoyl)oxy)pyrrolidine-1-carboxylate (**6b**)

Synthesized according to General Procedure 4. Purified via column chromatography (40–50% ethyl acetate/hexanes). White solid (40%, 210 mg). ^1^H NMR (400 MHz, CDCl_3_) δ 7.91–7.49 (m, 3H), 7.24 (d, *J* = 8.3 Hz, 2H), 5.33–5.26 (m, 1H), 3.61–3.31 (m, 4H), 2.13–2.00 (m, 2H), 1.46 (d, *J* = 5.9 Hz, 9H). ^13^C NMR (101 MHz, CDCl_3_) δ 154.7*, 154.6, 152.9, 138.2*, 138.0, 137.9*, 137.9, 120.6, 86.3*, 86.2, 79.9, 74.9, 73.8*, 52.2, 51.5*, 44.1*, 43.8, 32.0*, 31.0, 28.6. Material isolated as an approximately 1:1 ratio of rotamers. HRMS: (ESI) [M + H]^+^ calcd for C_16_H_22_IN_2_O_4_, 433.0619, observed, 433.0617.

#### *tert*-Butyl (S)-3-(((4-iodophenyl)carbamoyl)oxy)pyrrolidine-1-carboxylate (**6c**)

Synthesized according to General Procedure 4. Purified via column chromatography (40–50% ethyl acetate/hexanes). White solid (47%, 247 mg). ^1^H NMR (400 MHz, CDCl_3_) δ 8.19 (d, *J* = 130.7 Hz, 1H), 7.56 (d, *J* = 8.4 Hz, 2H), 7.29 (d, *J* = 8.2 Hz, 1H), 5.36–5.29 (m, 1H), 3.63–3.45 (m, 3H), 3.44–3.30 (m, 1H), 2.12–2.02 (m, 2H), 1.48 (d, *J* = 7.9 Hz, 9H). ^13^C NMR (101 MHz, CDCl_3_) δ 154.7*, 154.5, 152.9*, 152.9, 138.3, 138.2*, 137.8*, 137.7, 120.6, 86.1*, 86.0, 79.8, 74.6*, 73.6, 52.1*, 51.4, 44.0, 43.7*, 31.9, 30.9*, 28.5, 28.5*. Material isolated as an approximately 1:1 ratio of rotamers. HRMS: (ESI) [M + H]^+^ calcd for C_16_H_22_IN_2_O_4_, 433.0619, observed, 433.0619.

#### *tert*-Butyl (R)-3-(((4-iodophenyl)carbamoyl)oxy)piperidine-1-carboxylate (**6d**)

Synthesized according to General Procedure 4. Purified via column chromatography (20–40% ethyl acetate/hexanes). White solid (21%, 151 mg). ^1^H NMR (500 MHz, CD_3_OD) δ 7.60 (d, *J* = 8.9 Hz, 2H), 7.28 (brs, 2H), 4.79–4.65 (m, 1H), 4.14–2.91 (m, 4H), 1.98–1.77 (m, 3H), 1.61–1.26 (m, 10H). ^13^C NMR (126 MHz, CD_3_OD) δ 156.8, 154.9, 140.3, 138.8, 121.5, 86.1, 81.2, 70.0, 47.9 45.4*, 44.6, 30.1*, 29.9, 28.6, 22.9*, 22.1. One piperidine carbon signal overlaps with CD_3_OD solvent signal. Material isolated as an approximately 7:1 ratio of rotamers. HRMS: (ESI) [M + H]^+^ calcd for C_17_H_23_IN_2_NaO_4_, 469.0595, observed, 469.0600.

#### *tert*-Butyl (S)-3-(((4-iodophenyl)carbamoyl)oxy)piperidine-1-carboxylate (**6e**)

Synthesized according to General Procedure 4. Purified via column chromatography (15–40% ethyl acetate/hexanes). White solid (9%, 60 mg). ^1^H NMR (400 MHz, CD_3_OD) δ 7.58 (d, *J* = 8.9 Hz, 2H), 7.27 (d, *J* = 6.8 Hz, 2H), 4.77–4.68 (m, 1H), 4.08–3.00 (m, 4H), 1.94–1.76 (m, 3H), 1.57–1.28 (m, 10H). ^13^C NMR (126 MHz, CD_3_OD) δ 156.8, 154.9, 140.4, 138.8, 121.5, 86.1, 81.2, 70.0, 47.9, 45.4*, 44.6, 30.1*, 29.9, 28.6, 22.9*, 22.1. One piperidine carbon signal overlaps with CD_3_OD solvent signal. Material isolated as an approximately 7:1 ratio of rotamers. HRMS: (ESI) [M + Na]^+^ calcd for C_17_H_23_IN_2_NaO_4_, 469.0595, observed, 469.0602.

#### *tert*-Butyl 4-(((4-Iodophenyl)carbamoyl)oxy)piperidine-1-carboxylate (**6f**)

Synthesized according to General Procedure 4. Purified via column chromatography (20–40% ethyl acetate/hexanes). White solid (30%, 162 mg). ^1^H NMR (400 MHz, CDCl_3_) δ 7.57 (d, *J* = 8.8 Hz, 2H), 7.19 (d, *J* = 8.7 Hz, 2H), 7.09 (brs, 1H), 4.96–4.85 (m, 1H), 3.77–3.66 (m, 2H), 3.22 (ddd, *J* = 13.4, 8.8, 3.7 Hz, 2H), 1.95–1.84 (m, 2H), 1.68–1.53 (m, 2H), 1.45 (s, 9H). ^13^C NMR (101 MHz, CDCl_3_) δ 154.9, 152.7, 138.0, 138.0, 120.6, 86.3, 80.0, 70.9, 41.0, 30.9, 28.5. HRMS: (ESI) [M + Na]^+^ calcd for C_17_H_23_IN_2_NaO_4_, 469.0595, observed, 469.0596.

#### 2-((*tert*-Butoxycarbonyl)amino)ethyl (4-Decylphenyl)carbamate (**7a**)

Synthesized according to General Procedure 2. Purified via column chromatography (30–50% ethyl acetate/hexanes). Yellow solid (97%, 225 mg). ^1^H NMR (400 MHz, CDCl_3_) δ 7.28 (d, *J* = 7.4 Hz, 2H), 7.09 (d, *J* = 8.2 Hz, 2H), 7.04 (brs, 1H), 5.04–4.93 (m, 1H), 4.20 (t, *J* = 5.3 Hz, 2H), 3.41 (q, *J* = 5.6 Hz, 2H), 2.56–2.51 (m, 2H), 1.55 (p, *J* = 7.1 Hz, 2H), 1.44 (s, 9H), 1.32–1.21 (m, 14H), 0.87 (t, *J* = 6.8 Hz, 3H). ^13^C NMR (101 MHz, CDCl_3_) δ 156.1, 153.7, 138.3, 135.4, 129.0, 118.9, 79.7, 64.2, 40.2, 35.4, 32.0, 31.6, 29.7, 29.7, 29.6, 29.4, 29.3, 28.5, 22.8, 14.2. HRMS: (ESI) [M + Na]^+^ calcd for C_24_H_40_N_2_NaO_4_, 443.2880, observed, 443.2868.

#### *tert*-Butyl (R)-3-(((4-decylphenyl)carbamoyl)oxy)pyrrolidine-1-carboxylate (**7b**)

Synthesized according to General Procedure 2. Purified via column chromatography (20–30% ethyl acetate/hexanes). Off-white solid (92%, 200 mg). ^1^H NMR (400 MHz, CDCl_3_) δ 7.29 (d, *J* = 7.9 Hz, 2H), 7.10 (d, *J* = 8.1 Hz, 2H), 7.06–6.85 (m, 1H), 5.35–5.26 (m, 1H), 3.60–3.35 (m, 4H), 2.54 (t, *J* = 7.7 Hz, 2H), 2.12–2.06 (m, 2H), 1.55 (p, *J* = 7.3 Hz, 2H), 1.46 (s, 9H), 1.34–1.22 (m, 14H), 0.87 (t, *J* = 6.8 Hz, 3H). ^13^C NMR (101 MHz, CDCl_3_) δ 154.6, 153.1, 138.4, 135.5, 129.0, 118.8, 79.7, 74.7, 73.8*, 52.2, 51.7*, 44.1*, 43.8, 35.4, 32.1, 32.0*, 31.7, 31.1, 29.7, 29.7, 29.6, 29.4, 29.3, 28.6, 22.8, 14.2. NMR shows residual dichloromethane and ethyl acetate solvent peaks. Material isolated as an approximately 1:1 ratio of rotamers. HRMS: (ESI) [M + Na]^+^ calcd for C_26_H_42_N_2_NaO_4_, 469.3037, observed, 469.3036.

#### *tert*-Butyl (S)-3-(((4-decylphenyl)carbamoyl)oxy)pyrrolidine-1-carboxylate (**7c**)

Synthesized according to General Procedure 2. Purified via column chromatography (20–30% ethyl acetate/hexanes). Off-white solid (79%, 201 mg). ^1^H NMR (400 MHz, CDCl_3_) δ 7.30 (d, *J* = 7.9 Hz, 2H), 7.17–6.95 (m, 3H), 5.29 (s, 1H), 3.61–3.35 (m, 4H), 2.54 (t, *J* = 7.7 Hz, 2H), 2.12–2.06 (m, 2H), 1.55 (p, *J* = 7.3 Hz, 2H), 1.46 (s, 9H), 1.33–1.20 (m, 14H), 0.87 (t, *J* = 6.7 Hz, 3H). ^13^C NMR (101 MHz, CDCl_3_) δ 154.6, 153.1, 138.4, 135.4, 129.0, 118.8, 79.7, 74.6, 73.7*, 52.2, 51.6*, 44.1*, 43.8, 35.4, 32.0, 31.9*, 31.6, 31.0, 29.7, 29.7, 29.6, 29.4, 29.3, 28.6, 22.8, 14.2. NMR shows residual ethyl acetate impurity. Material isolated as an approximately 1:1 ratio of rotamers. HRMS: (ESI) [M + Na]^+^ calcd for C_26_H_42_N_2_NaO_4_, 469.3037, observed, 469.3040.

#### *tert*-Butyl (R)-3-(((4-decylphenyl)carbamoyl)oxy)piperidine-1-carboxylate (**7d**)

Synthesized according to General Procedure 2. Purified via column chromatography (10–30% ethyl acetate/hexanes). Amber solid (76%, 118 mg). ^1^H NMR (500 MHz, CDCl_3_) δ 7.28 (d, *J* = 6.6 Hz, 2H), 7.10 (d, *J* = 8.4 Hz, 2H), 6.70 (s, 1H), 4.78 (s, 1H), 3.81–3.18 (m, 4H), 2.54 (t, *J* = 7.7 Hz, 2H), 1.97–1.70 (m, 3H), 1.61–1.48 (m, 3H), 1.42 (s, 9H), 1.34–1.18 (m, 14H), 0.87 (t, *J* = 6.9 Hz, 3H). ^13^C NMR (126 MHz, CDCl_3_) δ 155.1, 152.9, 138.2, 135.5, 129.0, 118.7, 79.8, 68.8, 48.0, 47.3*, 44.3*, 43.5, 35.4, 32.0, 31.6, 29.7, 29.7, 29.6, 29.5, 29.4, 29.3, 28.5, 22.8, 22.0*, 21.7, 14.2. Material isolated as an approximately 5:1 ratio of rotamers. HRMS: (ESI) [M + H]^+^ calcd for C_27_H_45_N_2_O_4_, 461.3374, observed, 461.3366.

#### *tert*-Butyl (S)-3-(((4-decylphenyl)carbamoyl)oxy)piperidine-1-carboxylate (**7e**)

Synthesized according to General Procedure 2. Purified via column chromatography (10–30% ethyl acetate/hexanes). White solid (87%, 54 mg). ^1^H NMR (500 MHz, CDCl_3_) δ 7.28 (d, *J* = 7.3 Hz, 2H), 7.10 (d, *J* = 8.4 Hz, 2H), 6.64 (brs, 1H), 4.84–4.72 (m, 1H), 3.78–3.18 (m, 4H), 2.54 (t, *J* = 7.8 Hz, 2H), 1.99–1.72 (m, 3H), 1.62–1.48 (m, 3H), 1.42 (s, 9H), 1.34–1.18 (m, 14H), 0.87 (t, *J* = 7.0 Hz, 3H). ^13^C NMR (126 MHz, CDCl_3_) δ 155.1, 152.9, 138.2, 135.5, 129.0, 118.7, 79.8, 68.9, 48.0, 47.3*, 44.3*, 43.5, 35.4, 32.0, 31.6, 29.7, 29.7, 29.6, 29.5, 29.4, 29.4, 28.5, 22.8, 22.1*, 21.7, 14.2. Material isolated as an approximately 5:1 ratio of rotamers. HRMS: (ESI) [M + H]^+^ calcd for C_27_H_45_N_2_O_4_, 461.3374, observed, 461.3364.

#### *tert*-Butyl 4-(((4-decylphenyl)carbamoyl)oxy)piperidine-1-carboxylate (**7f**)

Synthesized according to General Procedure 2. Purified via column chromatography (30% ethyl acetate/hexanes). Yellow oil (87%, 145 mg). ^1^H NMR (500 MHz, CDCl_3_) δ 7.29 (d, *J* = 6.9 Hz, 2H), 7.10 (d, *J* = 8.5 Hz, 2H), 6.89 (brs, 1H), 4.95–4.87 (m, 1H), 3.76–3.69 (m, 2H), 3.22 (ddd, *J* = 13.7, 9.1, 4.4 Hz, 2H), 2.54 (t, *J* = 7.7 Hz, 2H), 1.94–1.85 (m, 2H), 1.70–1.53 (m, 4H), 1.46 (s, 9H), 1.33–1.21 (m, 14H), 0.87 (t, *J* = 7.0 Hz, 3H). ^13^C NMR (126 MHz, CDCl_3_) δ 154.9, 153.1, 138.2, 135.6, 129.0, 118.8, 79.8, 70.5, 41.2, 35.3, 32.0, 31.6, 31.0, 29.7, 29.7, 29.6, 29.4, 29.3, 28.5, 22.8, 14.2. HRMS: (ESI) [M + Na]^+^ calcd for C_27_H_44_N_2_NaO_4_, 483.3193, observed, 483.3195.

#### 2-Aminoethyl (4-Decylphenyl)carbamate Hydrochloride (**8a**)·

Synthesized according to General Procedure 3. Purified via trituration with ethyl acetate and diethyl ether. White solid (78%, 33 mg). ^1^H NMR (400 MHz, CD_3_OD) δ 7.35 (d, *J* = 8.1 Hz, 2H), 7.10 (d, *J* = 8.6 Hz, 2H), 4.37 (t, *J* = 5.1 Hz, 2H), 3.27 (t, *J* = 5.1 Hz, 2H), 2.56 (t, *J* = 7.6 Hz, 2H), 1.58 (p, *J* = 6.7 Hz, 2H), 1.38–1.26 (m, 14H), 0.89 (t, *J* = 6.7 Hz, 3H). ^13^C NMR (101 MHz, CD_3_OD) δ 155.2, 139.2, 137.3, 129.8, 120.1, 62.2, 40.4, 36.2, 33.1, 32.8, 30.7, 30.7, 30.6, 30.4, 30.3, 23.7, 14.4. HRMS: (ESI) [M + H]^+^ calcd for C_19_H_33_N_2_O_2_, 321.2537, observed, 321.2537.

#### (R)-Pyrrolidin-3-yl (4-Decylphenyl)carbamate Hydrochloride (**8b**)

Synthesized according to General Procedure 3. Purified via trituration with ethyl acetate and diethyl ether. White solid (82%, 53 mg). ^1^H NMR (400 MHz, CD_3_OD) δ 7.33 (d, *J* = 8.0 Hz, 2H), 7.10 (d, *J* = 8.5 Hz, 2H), 5.43–5.35 (m, 1H), 3.57–3.40 (m, 4H), 2.55 (t, *J* = 7.7 Hz, 2H), 2.33–2.26 (m, 2H), 1.58 (p, *J* = 6.7 Hz, 2H), 1.36–1.23 (m, 14H), 0.89 (t, *J* = 6.8 Hz, 3H). ^13^C NMR (101 MHz, CD_3_OD) δ 154.6, 139.2, 137.3, 129.8, 120.0, 74.5, 52.2, 45.4, 36.2, 33.1, 32.8, 31.9, 30.7, 30.7, 30.6, 30.4, 30.3, 23.7, 14.4. HRMS: (ESI) [M + H]^+^ calcd for C_21_H_35_N_2_O_2_, 347.2693, observed, 347.2693.

#### (S)-Pyrrolidin-3-yl (4-Decylphenyl)carbamate Hydrochloride (**8c**)

Synthesized according to General Procedure 3. Purified via trituration with ethyl acetate and diethyl ether. White solid (81%, 52 mg). ^1^H NMR (400 MHz, CD_3_OD) δ 7.33 (d, *J* = 7.7 Hz, 2H), 7.09 (d, *J* = 8.5 Hz, 2H), 5.43–5.35 (m, 1H), 3.58–3.40 (m, 4H), 2.55 (t, *J* = 7.6 Hz, 2H), 2.35–2.25 (m, 2H), 1.58 (p, *J* = 7.5 Hz, 2H), 1.39–1.20 (m, 14H), 0.89 (t, *J* = 6.6 Hz, 3H). ^13^C NMR (101 MHz, CD_3_OD) δ 154.6, 139.2, 137.3, 129.8, 120.0, 74.5, 52.1, 45.4, 36.2, 33.1, 32.8, 31.9, 30.7, 30.7, 30.6, 30.4, 30.3, 23.7, 14.5. HRMS: (ESI) [M + H]^+^ calcd for C_21_H_35_N_2_O_2_, 347.2693, observed, 347.2699.

#### (S)-Piperidin-3-yl (4-Decylphenyl)carbamate Hydrochloride (**8e**)

Synthesized according to General Procedure 3. Purified via trituration with ethyl acetate and diethyl ether. White solid (82%, 38 mg). ^1^H NMR (400 MHz, CD_3_OD) δ 7.36 (d, *J* = 8.3 Hz, 2H), 7.12 (d, *J* = 8.5 Hz, 2H), 5.06 (p, *J* = 4.3 Hz, 1H), 3.37–3.12 (m, 4H), 2.57 (t, *J* = 7.6 Hz, 2H), 2.19–1.80 (m, 4H), 1.60 (p, *J* = 7.1 Hz, 2H), 1.39–1.21 (m, 14H), 0.91 (t, *J* = 6.9 Hz, 3H). ^13^C NMR (101 MHz, CD_3_OD) δ 154.3, 139.2, 137.3, 129.8, 120.1, 67.1, 47.6, 44.9, 36.2, 33.1, 32.8, 30.7, 30.7, 30.6, 30.4, 30.3, 27.9, 23.7, 19.6, 14.4. HRMS: (ESI) [M + H]^+^ calcd for C_22_H_37_N_2_O_2_, 361.2850, observed, 361.2851.

#### (R)-Piperidin-3-yl (4-Decylphenyl)carbamate Hydrochloride (**8d**)

Synthesized according to General Procedure 3. Purified via trituration with ethyl acetate and diethyl ether. White solid (87%, 88 mg). ^1^H NMR (400 MHz, CD_3_OD) δ 7.36 (d, *J* = 8.5 Hz, 2H), 7.10 (d, *J* = 8.5 Hz, 2H), 5.05 (p, *J* = 4.2 Hz, 1H), 3.39–3.34 (m, 2H), 3.29–3.23 (m, 1H), 3.19–3.08 (m, 1H), 2.55 (t, *J* = 7.6 Hz, 2H), 2.16–2.03 (m, 1H), 2.02–1.89 (m, 2H), 1.82 (dp, *J* = 13.6, 4.8 Hz, 1H), 1.57 (p, *J* = 7.0 Hz, 2H), 1.37–1.20 (m, 14H), 0.88 (t, *J* = 7.0 Hz, 3H). ^13^C NMR (101 MHz, CD_3_OD) δ 154.3, 139.1, 137.3, 129.7, 120.1, 67.0, 47.6, 44.9, 36.2, 33.1, 32.8, 30.7, 30.7, 30.6, 30.4, 30.3, 27.8, 23.7, 19.5, 14.5. HRMS: (ESI) [M + H]^+^ calcd for C_22_H_37_N_2_O_2_, 361.2850, observed, 361.2851.

#### Piperidin-4-yl (4-Decylphenyl)carbamate Hydrochloride (**8f**)

Synthesized according to General Procedure 3. Purified via trituration with ethyl acetate and diethyl ether. White solid (80%, 100 mg). ^1^H NMR (400 MHz, CD_3_OD) δ 7.33 (d, *J* = 8.4 Hz, 2H), 7.09 (d, *J* = 8.6 Hz, 2H), 5.04–4.95 (m, 1H), 3.42–3.31 (m, 2H), 3.28–3.18 (m, 2H), 2.55 (t, *J* = 7.6 Hz, 2H), 2.22–2.10 (m, 2H), 2.06–1.93 (m, 2H), 1.58 (p, *J* = 7.1 Hz, 2H), 1.37–1.20 (m, 14H), 0.89 (t, *J* = 7.1 Hz, 3H). ^13^C NMR (101 MHz, CD_3_OD) δ 154.7, 139.0, 137.5, 129.7, 120.0, 67.4, 42.1, 36.2, 33.1, 32.8, 30.7, 30.7, 30.6, 30.4, 30.3, 28.7, 23.7, 14.4. HRMS: (ESI) [M + H]^+^ calcd for C_22_H_37_N_2_O_2_, 361.2850, observed, 361.2851.

#### *tert*-Butyl (3-(3-(4-Iodophenyl)ureido)propyl)carbamate (**9a**)

Synthesized according to General Procedure 5. Purified via column chromatography (40–60% ethyl acetate/hexanes). White solid (92%, 316 mg). ^1^H NMR (400 MHz, CD_3_OD) δ 7.53 (d, *J* = 8.8 Hz, 2H), 7.18 (d, *J* = 8.9 Hz, 2H), 3.21 (t, *J* = 6.7 Hz, 2H), 3.14–3.06 (m, 2H), 1.65 (p, *J* = 6.7 Hz, 2H), 1.43 (s, 9H). ^13^C NMR (101 MHz, CD_3_OD) δ 158.6, 157.9, 141.0, 138.7, 121.9, 85.4, 79.9, 38.6, 38.0, 31.5, 28.8. HRMS: (ESI) [M + H]^+^ calcd for C_15_H_23_IN_3_O_3_, 420.0779, observed, 420.0776.

#### *tert*-Butyl 3-(3-(4-Iodophenyl)ureido)azetidine-1-carboxylate (**9b**)

Synthesized according to General Procedure 5. Purified via column chromatography (70% ethyl acetate/hexanes). White solid (96%, 326 mg). ^1^H NMR (400 MHz, CDCl_3_) δ 7.67 (brs, 1H), 7.51 (d, *J* = 8.8 Hz, 2H), 7.06 (d, *J* = 8.9 Hz, 2H), 6.19 (d, *J* = 7.1 Hz, 1H), 4.53–4.44 (m, 1H), 4.22 (t, *J* = 8.4 Hz, 2H), 3.65 (dd, *J* = 9.4, 5.0 Hz, 2H), 1.44 (s, 9H). ^13^C NMR (101 MHz, CDCl_3_) δ 156.8, 155.1, 138.6, 138.0, 121.3, 86.0, 80.7, 56.8, 40.0, 28.5. HRMS: (ESI) [M + Na]^+^ calcd for C_15_H_20_IN_3_NaO_3_, 440.0442, observed, 440.0434.

#### *tert*-Butyl (R)-3-(3-(4-Iodophenyl)ureido)pyrrolidine-1-carboxylate (**9c**)

Synthesized according to General Procedure 5. Purified via column chromatography (60–70% ethyl acetate/hexanes). White solid (84%, 445 mg). ^1^H NMR (400 MHz, CDCl_3_) δ 7.87 (d, *J* = 35.2 Hz, 1H), 7.48 (d, *J* = 8.8 Hz, 2H), 7.09 (d, *J* = 8.8 Hz, 2H), 6.07 (dd, *J* = 37.2, 7.1 Hz, 1H), 4.38–4.27 (m, 1H), 3.54–3.27 (m, 3H), 3.20–3.13 (m, 1H), 2.10–1.76 (m, 2H), 1.47 (s, 4.5H), 1.44 (s, 4.5H)*. ^13^C NMR (101 MHz, CDCl_3_) δ 155.3, 155.3, 139.2, 137.8, 120.8, 85.0, 80.5*, 80.4, 52.5*, 51.8, 49.9*, 49.2, 44.1, 43.9*, 32.4, 31.3*, 28.6. Material isolated as an approximately 1:1 mixture of rotamers. HRMS: (ESI) [M + H]^+^ calcd for C_16_H_23_IN_3_O_3_, 432.0779, observed, 432.0783.

#### *tert*-Butyl (S)-3-(3-(4-Iodophenyl)ureido)pyrrolidine-1-carboxylate (**9d**)

Synthesized according to General Procedure 5. Purified via column chromatography (50–70% ethyl acetate/hexanes). White solid (95%, 670 mg). ^1^H NMR (400 MHz, CDCl_3_) δ 7.88 (d, *J* = 35.1 Hz, 1H), 7.47 (d, *J* = 8.9 Hz, 2H), 7.08 (d, *J* = 8.7 Hz, 2H), 6.07 (dd, *J* = 38.0, 7.0 Hz, 1H), 4.38–4.27 (m, 1H), 3.50 (dd, *J* = 11.5, 5.6 Hz, 1H), 3.46–3.26 (m, 2H), 3.15 (dd, *J* = 11.5, 2.2 Hz, 1H), 2.12–1.95 (m, 1H), 1.94–1.73 (m, 1H), 1.45 (d, *J* = 12.5 Hz, 9H). ^13^C NMR (101 MHz, CDCl_3_) δ 155.3, 155.2, 139.2, 137.7, 120.8, 85.0, 80.4, 80.3*, 52.4, 51.7*, 49.8*, 49.1, 44.1, 43.9*, 32.3, 31.3*, 28.6. HRMS: (ESI) [M + H]^+^ calcd for C_16_H_23_IN_3_O_3_, 432.0779, observed, 432.0784.

#### *tert*-Butyl (R)-3-(3-(4-Iodophenyl)ureido)piperidine-1-carboxylate (**9e**)

Synthesized according to General Procedure 5. Purified via column chromatography (50–70% ethyl acetate/hexanes). White solid (92%, 418 mg). ^1^H NMR (400 MHz, CDCl_3_) δ 7.95 (brs, 1H), 7.46 (d, *J* = 8.8 Hz, 2H), 7.03 (d, *J* = 8.8 Hz, 2H), 5.82 (d, *J* = 7.7 Hz, 1H), 3.80–3.68 (m, 1H), 3.58 (dd, *J* = 12.9, 3.8 Hz, 1H), 3.43–3.33 (m, 1H), 3.24–3.13 (m, 1H), 3.07 (dd, *J* = 13.0, 7.1 Hz, 1H), 1.81–1.70 (m, 1H), 1.64–1.53 (m, 1H), 1.48–1.36 (m, 11H). ^13^C NMR (101 MHz, CDCl_3_) δ 171.4, 155.3, 139.2, 137.8, 121.3, 85.3, 80.4, 48.6, 45.5, 44.4, 30.2, 28.5, 22.4. HRMS: (ESI) [2M+H]^+^ calcd for C_34_H_49_I_2_N_6_O_6_, 891.1797, observed, 891.1777.

#### *tert*-Butyl (S)-3-(3-(4-Iodophenyl)ureido)piperidine-1-carboxylate (**9f**)

Synthesized according to General Procedure 5. Purified via column chromatography (30–50% ethyl acetate/hexanes). Off-white solid (73%, 333 mg). ^1^H NMR (400 MHz, CDCl_3_) δ 7.88 (brs, 1H), 7.48 (d, *J* = 8.6 Hz, 2H), 7.06 (d, *J* = 8.7 Hz, 2H), 5.76 (d, *J* = 7.6 Hz, 1H), 3.84–3.72 (m, 1H), 3.52 (dd, *J* = 13.1, 3.6 Hz, 1H), 3.36–3.24 (m, 2H), 3.18 (dd, *J* = 13.0, 6.6 Hz, 1H), 1.82–1.68 (m, 1H), 1.64–1.35 (m, 12H). ^13^C NMR (101 MHz, CDCl_3_) δ 171.4, 155.2, 139.2, 137.9, 121.3, 85.3, 80.5, 48.7, 45.4, 44.5, 30.1, 28.5, 22.3. HRMS: (ESI) [M + H]^+^ calcd for C_17_H_25_IN_3_O_3_, 446.0935, observed, 446.0941.

#### *tert*-Butyl (1-((4-Iodophenyl)carbamoyl)piperidin-4-yl)carbamate (**9g**)

Synthesized according to General Procedure 5. During the course of the reaction a thick white precipitated formed. This precipitate was filtered over a Hirsch funnel and rinsed with hexanes. The resulting white solid was carried forward crude with no additional purification.

#### *tert*-Butyl 4-(3-(4-Iodophenyl)ureido)piperidine-1-carboxylate (**9h**)

Synthesized according to General Procedure 5. Purified via column chromatography (40–50% ethyl acetate/hexanes). White solid (96%, 347 mg). ^1^H NMR (400 MHz, CDCl_3_) δ 7.67 (brs, 1H), 7.49 (d, *J* = 8.7 Hz, 2H), 7.04 (d, *J* = 8.8 Hz, 2H), 5.60 (d, *J* = 7.6 Hz, 1H), 3.98–3.87 (m, 2H), 3.81–3.69 (m, 1H), 2.83 (t, *J* = 12.2 Hz, 1H), 1.85 (dd, *J* = 13.3, 4.3 Hz, 1H), 1.45 (s, 9H), 1.21–1.09 (m, 2H). ^13^C NMR (101 MHz, CDCl_3_) δ 155.2, 155.1, 139.0, 137.9, 121.3, 85.6, 80.3, 46.9, 42.4, 32.6, 28.6. HRMS: (ESI) [M + H]^+^ calcd for C_17_H_25_IN_3_O_3_, 446.0935, observed, 446.0945.

#### *tert*-Butyl 4-((4-Iodophenyl)carbamoyl)piperazine-1-carboxylate (**9i**)

Synthesized according to General Procedure 5. Purified via column chromatography (40–50% ethyl acetate/hexanes). White solid (97%, 512 mg). ^1^H NMR (400 MHz, CDCl_3_) δ 7.47 (d, *J* = 8.7 Hz, 2H), 7.17 (brs, 1H), 7.07 (d, *J* = 8.8 Hz, 2H), 3.43–3.32 (m, 8H), 1.44 (s, 9H). ^13^C NMR (101 MHz, CDCl_3_) δ 155.0, 154.6, 139.0, 137.6, 122.3, 86.2, 80.4, 43.8, 42.9, 28.4. HRMS: (ESI) [M + H]^+^ calcd for C_16_H_23_IN_3_O_3_, 432.0779, observed, 432.0771.

#### *tert*-Butyl 5-((4-Iodophenyl)carbamoyl)-2,5-diazabicyclo[2.2.1]heptane-2-carboxylate (**9j**)

Synthesized according to General Procedure 5. Purified via column chromatography (60–70% ethyl acetate/hexanes). White solid (95%, 518 mg). ^1^H NMR (400 MHz, CDCl_3_) δ 7.53 (d, *J* = 8.8 Hz, 2H), 7.22–7.13 (m, 2H), 6.55–6.39 (m, 1H), 4.69–4.46 (m, 2H), 3.51–3.29 (m, 4H), 1.90–1.76 (m, 2H), 1.47–1.41 (m, 9H). ^13^C NMR (101 MHz, CDCl_3_) δ 154.3, 154.2*, 153.5, 138.8, 137.7, 121.7*, 121.7, 85.9, 80.2*, 80.2, 57.5*, 56.8, 56.8, 56.5*, 54.0, 53.7, 53.6*, 37.4*, 36.8, 28.6*, 28.5. Material isolated as an approximately 2:1 ratio of rotamers. HRMS: (ESI) [M + H]^+^ calcd for C_17_H_23_IN_3_O_3_, 444.0779, observed, 444.0770.

#### *tert*-Butyl 3-((4-Iodophenyl)carbamoyl)-3,8-diazabicyclo[3.2.1]octane-8-carboxylate (**9k**)

Synthesized according to General Procedure 5. Purified via column chromatography (30–40% ethyl acetate/hexanes). White solid (99%, 745 mg). ^1^H NMR (400 MHz, CDCl_3_) δ 7.53 (d, *J* = 8.5 Hz, 2H), 7.16 (d, *J* = 8.5 Hz, 2H), 6.80 (s, 1H), 4.31–4.23 (m, 2H), 3.78 (dd, *J* = 55.8, 13.2 Hz, 2H), 3.07 (dd, *J* = 45.4, 13.2 Hz, 2H), 2.00–1.66 (m, 4H), 1.45 (s, 9H). ^13^C NMR (101 MHz, CDCl_3_) δ 156.0, 153.3, 138.9, 137.8, 121.8, 86.1, 80.3, 54.2*, 53.5, 49.8, 48.6*, 28.5, 27.3*, 27.0. HRMS: (ESI) [M + H]^+^ calcd for C_18_H_25_IN_3_O_3_, 458.0935, observed, 458.0945.

#### *tert*-Butyl (R)-4-((4-Iodophenyl)carbamoyl)-2-methylpiperazine-1-carboxylate (**9l**)

Synthesized according to General Procedure 5. Purified via column chromatography (30–50% ethyl acetate/hexanes). White solid (98%, 434 mg) ^1^H NMR (400 MHz, CDCl_3_) δ 7.57–7.53 (m, 2H), 7.16–7.12 (m, 2H), 6.67 (s, 1H), 4.25 (s, 1H), 3.92–3.82 (m, 2H), 3.77–3.68 (m, 1H), 3.27–3.11 (m, 2H), 3.07–2.98 (m, 1H), 1.48 (s, 9H), 1.17 (d, *J* = 6.7 Hz, 3H). ^13^C NMR (126 MHz, CDCl_3_) δ 155.1, 154.7, 138.9, 137.8, 122.0, 86.2, 80.4, 47.6, 47.4, 44.0, 38.3, 28.5, 16.0. HRMS: (ESI) [M + H]^+^ calcd for C_17_H_25_IN_3_O_3_^+^ 446.0935, observed, 446.0935.

#### *tert*-Butyl 4-((4-Iodophenyl)carbamoyl)-1,4-diazepane-1-carboxylate (**9m**)

Synthesized according to General Procedure 5. Purified via column chromatography (40–50% ethyl acetate/hexanes). White solid (83%, 303 mg). ^1^H NMR (500 MHz, CDCl_3_) δ 7.58–7.54 (m, 2H), 7.18–7.14 (m, 2H), 6.44 (s, 0.65H), 6.27 (s, 0.35H)*, 3.61–3.44 (m, 7H), 3.40 (t, *J* = 6.0 Hz, 1H), 2.00–1.94 (m, 0.7H)*, 1.87–1.82 (m, 1.3H), 1.61–1.56 (m, 2H), 1.45 (s, 3H)*, 1.43 (s, 6H). ^13^C NMR (126 MHz, CDCl_3_) δ 155.8, 154.8, 139.2, 137.9*, 137.8, 121.9*, 121.7, 80.3, 48.7, 48.3, 47.6, 46.3, 28.5, 27.0. Material isolated as an approximately 2:1 mixture of rotamers.

#### *tert*-Butyl (3-(3-(4-Decylphenyl)ureido)propyl)carbamate (**10a**)

Synthesized according to General Procedure 2. Purified via column chromatography (35–55% ethyl acetate/hexanes). White solid (68%, 224 mg). ^1^H NMR (400 MHz, CDCl_3_) δ 7.36 (brs, 1H), 7.18 (d, *J* = 8.0 Hz, 2H), 7.04 (d, *J* = 8.2 Hz, 2H), 5.80 (t, *J* = 7.6 Hz, 1H), 5.13 (t, *J* = 6.3 Hz, 1H), 3.21 (q, *J* = 6.2 Hz, 2H), 3.12 (q, *J* = 6.5 Hz, 2H), 2.51 (t, *J* = 7.8 Hz, 2H), 1.62–1.49 (m, 4H), 1.41 (s, 9H), 1.34–1.20 (m, 14H), 0.87 (t, *J* = 6.7 Hz, 3H). ^13^C NMR (101 MHz, CDCl_3_) δ 156.9, 156.7, 138.0, 136.6, 129.0, 120.7, 79.4, 37.4, 36.8, 35.4, 32.0, 31.7, 30.8, 29.7, 29.7, 29.6, 29.5, 29.4, 28.5, 22.8, 14.2. HRMS: (ESI) [M + H]^+^ calcd for C_25_H_44_N_3_O_3_, 434.3377, observed, 434.3372.

#### *tert*-Butyl 3-(3-(4-Decylphenyl)ureido)azetidine-1-carboxylate (**10b**)

Synthesized according to General Procedure 2. Purified via column chromatography (30–50% ethyl acetate/hexanes). Light brown solid (78%, 264 mg). ^1^H NMR (400 MHz, CDCl_3_) δ 7.53 (brs, 1H), 7.17 (d, *J* = 8.5 Hz, 2H), 7.05 (d, *J* = 8.5 Hz, 2H), 6.18 (d, *J* = 7.2 Hz, 1H), 4.57–4.44 (m, 1H), 4.18 (t, *J* = 8.5 Hz, 2H), 3.65 (dd, *J* = 9.3, 5.2 Hz, 2H), 2.50 (t, *J* = 7.8 Hz, 2H), 1.54 (p, *J* = 7.1 Hz, 2H), 1.43 (s, 9H), 1.34–1.19 (m, 14H), 0.87 (t, *J* = 6.8 Hz, 3H). ^13^C NMR (101 MHz, CDCl_3_) δ 156.5, 155.8, 138.2, 136.2, 129.0, 120.3, 80.2, 57.0, 40.0, 35.4, 32.0, 31.7, 29.7, 29.7, 29.6, 29.4, 29.4, 28.5, 22.8, 14.2. HRMS: (ESI) [M + H]^+^ calcd for C_25_H_42_N_3_O_3_, 432.3221, observed, 432.3222.

#### *tert*-Butyl (R)-3-(3-(4-Decylphenyl)ureido)pyrrolidine-1-carboxylate (**10c**)

Synthesized according to General Procedure 2. Purified via column chromatography (30–40% ethyl acetate/hexanes). Light brown solid (88%, 402 mg). ^1^H NMR (400 MHz, CDCl_3_) δ 7.40 (d, *J* = 6.3 Hz, 1H), 7.21 (d, *J* = 8.4 Hz, 2H), 7.05 (d, *J* = 8.4 Hz, 2H), 5.78 (dd, *J* = 55.6, 7.1 Hz, 1H), 4.43–4.32 (m, *J* = 3.9 Hz, 1H), 3.57–3.30 (m, 3H), 3.23–3.16 (m, 1H), 2.52 (t, *J* = 7.7 Hz, 2H), 2.13–1.76 (m, 2H), 1.55 (p, *J* = 7.5 Hz, 2H), 1.48 (s, 4.5H), 1.46 (s, 4.5H)*, 1.34–1.22 (m, 14H), 0.87 (t, *J* = 6.9 Hz, 3H). ^13^C NMR (101 MHz, CDCl_3_) δ 155.8, 155.2, 137.9, 137.7*, 136.7, 129.1, 120.0, 119.8*, 80.1, 52.6, 51.7*, 50.1, 49.4*, 44.2*, 44.0, 35.4, 32.6, 32.0, 31.7, 31.4*, 29.8, 29.7, 29.6, 29.5, 29.4, 28.7, 22.8, 14.2. Material isolated as an approximately 1:1 mixture of rotamers. HRMS: (ESI) [M + H]^+^ calcd for C_26_H_44_N_3_O_3_, 446.3377, observed, 446.3371.

#### *tert*-Butyl (S)-3-(3-(4-Decylphenyl)ureido)pyrrolidine-1-carboxylate (**10d**)

Synthesized according to General Procedure 2. Purified via column chromatography (30–40% ethyl acetate/hexanes). Off-white solid (73%, 505 mg). ^1^H NMR (400 MHz, CDCl_3_) δ 7.63 (d, *J* = 24.8 Hz, 1H), 7.20 (d, *J* = 6.3 Hz, 2H), 7.03 (d, *J* = 8.5 Hz, 2H), 5.98 (dd, *J* = 38.9, 7.1 Hz, 1H), 4.41–4.29 (m, 1H), 3.53 (dd, *J* = 11.4, 5.8 Hz, 1H), 3.46–3.29 (m, 2H), 3.16 (dd, *J* = 11.2, 3.6 Hz, 1H), 2.50 (t, *J* = 7.7 Hz, 2H), 2.12–1.95 (m, 1H), 1.93–1.70 (m, 1H), 1.60–1.43 (m, 11H), 1.35–1.17 (m, 14H), 0.87 (t, *J* = 6.9 Hz, 3H). ^13^C NMR (101 MHz, CDCl_3_) δ 156.0, 155.2, 137.5, 136.8, 128.9, 119.6, 80.1*, 80.0, 52.5, 51.7*, 50.0*, 49.3, 44.2*, 44.0, 35.4, 32.5*, 32.0, 31.7, 31.3, 29.7, 29.7, 29.6, 29.4, 29.4, 28.6, 22.8, 14.2. HRMS: (ESI) [M + H]^+^ calcd for C_26_H_44_N_3_O_3_, 446.3377, observed, 446.3371.

#### *tert*-Butyl (R)-3-(3-(4-Decylphenyl)ureido)piperidine-1-carboxylate (**10e**)

Synthesized according to General Procedure 2. Purified via column chromatography (30–40% ethyl acetate/hexanes). Light brown solid (93%, 400 mg). ^1^H NMR (400 MHz, CDCl_3_) δ 7.65 (brs, 1H), 7.17 (d, *J* = 8.6 Hz, 2H), 7.02 (d, *J* = 8.6 Hz, 2H), 5.75 (d, *J* = 7.7 Hz, 1H), 3.84–3.72 (m, 1H), 3.61 (dd, *J* = 12.9, 3.7 Hz, 1H), 3.44–3.35 (m, 1H), 3.22–3.04 (m, 2H), 2.49 (t, *J* = 7.7 Hz, 2H), 1.84–1.75 (m, 1H), 1.65–1.47 (m, 3H), 1.48–1.36 (m, 11H), 1.31–1.23 (m, 14H), 0.88 (t, *J* = 6.7 Hz, 3H). ^13^C NMR (101 MHz, CDCl_3_) δ 155.8, 155.4, 137.6, 136.7, 129.0, 120.2, 80.1, 49.0, 45.6, 43.9, 35.3, 32.0, 31.6, 30.4, 29.7, 29.7, 29.6, 29.4, 29.4, 28.5, 22.7, 22.6, 14.2. HRMS: (ESI) [M + H]^+^ calcd for C_27_H_45_N_3_NaO_3_, 482.3353, observed, 482.3344.

#### *tert*-Butyl (S)-3-(3-(4-Decylphenyl)ureido)piperidine-1-carboxylate (**10f**)

Synthesized according to General Procedure 2. Purified via column chromatography (20–50% ethyl acetate/hexanes). Light brown oil (79%, 270 mg). ^1^H NMR (400 MHz, CDCl_3_) δ 7.51 (brs, 1H), 7.18 (d, *J* = 8.4 Hz, 2H), 7.04 (d, *J* = 8.4 Hz, 2H), 5.66 (d, *J* = 7.7 Hz, 1H), 3.87–3.76 (m, 1H), 3.56 (dd, *J* = 13.0, 3.5 Hz, 1H), 3.39–3.15 (m, 3H), 2.51 (t, *J* = 7.6 Hz, 2H), 1.83–1.74 (m, 1H), 1.64–1.36 (m, 14H), 1.35–1.18 (m, 14H), 0.87 (t, *J* = 6.8 Hz, 3H). ^13^C NMR (101 MHz, CDCl_3_) δ 155.8, 155.5, 137.9, 136.7, 129.1, 120.5, 80.1, 49.0, 45.6, 44.1, 35.4, 32.0, 31.7, 30.3, 29.7, 29.7, 29.6, 29.4, 29.4, 28.5, 22.8, 22.5, 14.2. HRMS: (ESI) [M + H]^+^ calcd for C_27_H_46_N_3_O_3_, 460.3534, observed, 460.3533.

#### *tert*-Butyl (1-((4-Decylphenyl)carbamoyl)piperidin-4-yl)carbamate (**10g**)

Synthesized according to General Procedure 2. Purified via column chromatography (35–50% ethyl acetate/hexanes). White solid (53%, 190 mg). ^1^H NMR (400 MHz, CD_3_OD) δ 7.23 (d, *J* = 8.4 Hz, 1H), 7.09 (d, *J* = 8.4 Hz, 1H), 4.14–4.06 (m, 2H), 3.62–3.53 (m, 1H), 3.06–2.96 (m, 2H), 2.57 (t, *J* = 7.6 Hz, 1H), 1.96–1.87 (m, 2H), 1.60 (p, *J* = 7.5 Hz, 2H), 1.47 (s, 9H), 1.38–1.25 (m, 17H), 0.91 (d, *J* = 6.8 Hz, 3H). ^13^C NMR (101 MHz, CD_3_OD) δ 160.7, 138.9, 138.4, 129.5, 122.5, 54.7, 49.6, 44.3, 36.3, 33.2, 33.1, 32.8, 30.7, 30.7, 30.6, 30.5, 30.3, 28.8, 23.7, 14.4. HRMS: (ESI) [M + H]^+^ calcd for C_27_H_46_N_3_O_3_, 460.3534, observed, 482.3394.

#### *tert*-Butyl 4-(3-(4-Decylphenyl)ureido)piperidine-1-carboxylate (**10h**)

Synthesized according to General Procedure 2. Purified via column chromatography (30–40% ethyl acetate/hexanes). Off-white solid (82%, 295 mg). ^1^H NMR (400 MHz, CDCl_3_) δ 7.48 (s, 1H), 7.15 (d, *J* = 8.2 Hz, 2H), 7.03 (d, *J* = 8.2 Hz, 2H), 5.58 (d, *J* = 7.8 Hz, 1H), 3.99–3.83 (m, 2H), 3.82–3.70 (m, 1H), 2.79 (t, *J* = 11.1 Hz, 1H), 2.50 (t, *J* = 7.5 Hz, 2H), 1.84 (dd, *J* = 13.2, 4.3 Hz, 2H), 1.53 (p, *J* = 7.5 Hz, 2H), 1.44 (s, 9H), 1.33–1.13 (m, 16H), 0.87 (t, *J* = 6.7 Hz, 3H). ^13^C NMR (101 MHz, CDCl_3_) δ 155.9, 154.9, 137.9, 136.5, 129.0, 120.3, 79.9, 47.0, 42.6, 35.4, 32.6, 32.0, 31.7, 29.7, 29.7, 29.6, 29.4, 29.4, 28.5, 22.7, 14.2. HRMS: (ESI) [M + H]^+^ calcd for C_27_H_46_N_3_O_3_, 460.3534, observed, 460.3523.

#### *tert*-Butyl 4-((4-Decylphenyl)carbamoyl)piperazine-1-carboxylate (**10i**)

Synthesized according to General Procedure 2. Purified via column chromatography (20–40% ethyl acetate/hexanes). White solid (22%, 122 mg). ^1^H NMR (400 MHz, CDCl_3_) δ 7.22 (d, *J* = 8.5 Hz, 2H), 7.08 (d, *J* = 8.5 Hz, 2H), 6.48 (brs, 1H), 3.49–3.41 (m, 8H), 2.53 (t, *J* = 7.7 Hz, 2H), 1.56 (p, *J* = 7.6 Hz, 2H), 1.47 (s, 9H), 1.33–1.22 (m, 14H), 0.87 (t, *J* = 6.8 Hz, 3H). ^13^C NMR (101 MHz, CDCl_3_) δ 155.4, 154.8, 138.2, 136.4, 128.9, 120.5, 80.4, 44.0, 43.0, 35.4, 32.0, 31.7, 29.7, 29.7, 29.6, 29.4, 29.4, 28.5, 22.8, 14.2. HRMS: (ESI) [M + H]^+^ calcd for C_26_H_44_N_3_O_3_, 446.3377, observed, 446.3382.

#### *tert*-Butyl 5-((4-Decylphenyl)carbamoyl)-2,5-diazabicyclo[2.2.1]heptane-2-carboxylate (**10j**)

Synthesized according to General Procedure 2. Purified via column chromatography (35–60% ethyl acetate/hexanes). Off-white solid (77%, 401 mg). ^1^H NMR (500 MHz, CDCl_3_) δ 7.31–7.24 (m, 2H), 7.08 (d, *J* = 8.4 Hz, 2H), 6.22–6.12 (m, 1H), 4.73–4.47 (m, 2H), 3.55–3.31 (m, 4H), 2.53 (t, *J* = 7.5 Hz, 2H), 1.90–1.80 (m, 2H), 1.56 (p, *J* = 7.2 Hz, 2H), 1.49–1.42 (m, 9H), 1.33–1.20 (m, 14H), 0.87 (t, *J* = 6.9 Hz, 3H). ^13^C NMR (126 MHz, CDCl_3_) δ 154.4, 154.2*, 154.0, 153.9*, 138.0, 136.4, 128.9, 120.1*, 119.9, 80.1*, 80.1, 57.7*, 56.9, 56.8, 56.4*, 54.1, 53.9*, 53.7, 53.6*, 37.4*, 36.8, 35.4, 32.0, 31.7, 29.7, 29.7, 29.6, 29.4, 29.4, 28.6*, 28.5, 22.8, 14.2. Material isolated as an approximately 2:1 ratio of rotamers. HRMS: (ESI) [M + H]^+^ calcd for C_27_H_44_N_3_O_3_, 458.3377, observed, 458.3376.

#### *tert*-Butyl 3-((4-Decylphenyl)carbamoyl)-3,8-diazabicyclo[3.2.1]octane-8-carboxylate (**10k**)

Synthesized according to General Procedure 2. Purified via column chromatography (30–40% ethyl acetate/hexanes). White solid (60%, 483 mg). ^1^H NMR (400 MHz, CDCl_3_) δ 7.28 (d, *J* = 8.4 Hz, 2H), 7.22 (s, 1H), 7.04 (d, *J* = 8.4 Hz, 2H), 4.33–4.25 (m, 2H), 3.72 (dd, *J* = 57.3, 13.2 Hz, 2H), 3.06 (dd, *J* = 43.5, 12.7 Hz, 2H), 2.52 (t, *J* = 7.7 Hz, 2H), 1.89–1.80 (m, 2H), 1.71–1.51 (m, 4H), 1.45 (s, 9H), 1.34–1.23 (m, 14H), 0.88 (t, *J* = 6.8 Hz, 3H). ^13^C NMR (101 MHz, CDCl_3_) δ 155.9, 154.1, 137.7, 136.6, 128.6, 120.3, 80.0, 53.8*, 53.2, 49.7*, 48.5, 35.3, 31.9, 31.6, 29.6, 29.6, 29.5, 29.3, 29.3, 28.4, 27.2, 26.9*, 22.7, 14.1. HRMS: (ESI) [M + H]^+^ calcd for C_28_H_46_N_3_O_3_, 472.3534, observed, 472.3530.

#### *tert*-Butyl (R)-4-((4-Decylphenyl)carbamoyl)-2-methylpiperazine-1-carboxylate (**10l**)

Synthesized according to General Procedure 2. Purified via column chromatography (30–40% ethyl acetate/hexanes). Clear oil (54%, 56 mg). ^1^H NMR (400 MHz, CDCl_3_) δ 7.26–7.22 (m, 2H), 7.11–7.07 (m, 2H), 6.36 (brs, 1H), 4.26 (s, 1H), 3.88 (t, *J* = 13.3 Hz, 2H), 3.74–3.67 (m, 1H), 3.31–3.23 (m, 1H), 3.23–3.14 (m, 1H), 3.09–2.99 (m, 1H), 2.58–2.50 (m, 2H), 1.60–1.52 (m, 2H), 1.48 (s, 9H), 1.33–1.22 (m, 14H), 1.20 (d, *J* = 6.7 Hz, 3H), 0.90–0.84 (m, 3H). ^13^C NMR (126 MHz, CDCl_3_) δ 155.5, 154.6, 138.1, 136.3, 128.8, 120.3, 80.1, 47.6, 43.9, 38.3, 35.3, 31.9, 31.6, 29.6, 29.6, 29.6, 29.5, 29.3, 29.3, 28.4, 22.7, 15.9, 14.1. HRMS: (ESI) [M + H]^+^ calcd for C27H46N3O3^+^ 460.3534, observed, 460.3538

#### *tert*-Butyl 4-((4-Decylphenyl)carbamoyl)-1,4-diazepane-1-carboxylate (**10m**)

Synthesized according to General Procedure 2. Purified via column chromatography (30–40% ethyl acetate/hexanes). Clear oil (54%, 56 mg). Brown oil (15%, 30 mg). ^1^H NMR (500 MHz, CDCl_3_) δ 7.25 (d, *J* = 7.7 Hz, 2H), 7.13–7.04 (m, 2H), 6.41–6.24 (m, 1H), 3.62–3.45 (m, 6H), 3.40 (t, *J* = 6.2 Hz, 1H), 2.54 (t, *J* = 7.7 Hz, 2H), 2.02–1.93 (m, 1H), 1.94–1.82 (m, 2H), 1.76–1.64 (m, 1H), 1.49–1.40 (m, 9H), 1.33–1.20 (m, 15H), 0.88 (t, *J* = 6.8 Hz, 3H). ^13^C NMR (126 MHz, CDCl_3_) δ 155.5, 155.0*, 155.0, 154.9*, 138.0, 137.7, 136.5*, 136.4*, 128.8, 128.7, 120.2, 120.1, 80.0, 48.8, 48.3, 47.9, 47.1, 46.1, 46.0, 45.9, 35.3, 31.9, 31.6, 29.6, 29.6, 29.5, 29.3, 29.3, 28.4, 22.7, 14.1. Material isolated as an approximately 1:1 ratio of rotamers. HRMS: (ESI) [M + H]^+^ calcd for C_27_H_46_N_3_O_3_^+^ 460.3534, observed, 460.3521.

#### 1-(3-Aminopropyl)-3-(4-decylphenyl)urea Hydrochloride (**11a**)

Synthesized according to General Procedure 3. Purified via trituration with ethyl acetate and diethyl ether. White solid (84%, 160 mg). ^1^H NMR (400 MHz, CD_3_OD) δ 7.28 (d, *J* = 8.4 Hz, 2H), 7.09 (d, *J* = 8.5 Hz, 2H), 3.33 (t, *J* = 6.4 Hz, 2H), 3.02 (t, *J* = 7.1 Hz, 2H), 2.56 (t, *J* = 7.6 Hz, 2H), 1.88 (p, *J* = 6.9 Hz, 2H), 1.60 (p, *J* = 7.4 Hz, 2H), 1.37–1.28 (m, 14H), 0.92 (t, *J* = 6.8 Hz, 3H). ^13^C NMR (101 MHz, CD_3_OD) δ 159.1, 138.5, 138.2, 129.7, 120.8, 38.1, 37.1, 36.2, 33.1, 32.8, 30.7, 30.7, 30.6, 30.4, 30.3, 29.5, 23.7, 14.4. HRMS: (ESI) [M + H]^+^ calcd for C_20_H_36_N_3_O, 334.2853, observed, 334.2843.

#### 1-(Azetidin-3-yl)-3-(4-decylphenyl)urea 2,2,2-Trifluoroacetate (**11b**)

*tert*-butyl 3-(3-(4-iodophenyl)ureido)azetidine-1-carboxylate (**10b**) was dissolved in DCM and added to a 6-dram vial containing a stir bar. Trifluoroacetic acid (20.0 equiv) was added to the flask and the mixture was allowed to stir at room temperature for 2 h. Following completion as monitored by TLC, the material was loaded onto Celite and subjected to silica gel chromatography. Purified via column chromatography (10–15% methanol/dichloromethane). White solid (50%, 52 mg). ^1^H NMR (400 MHz, CD_3_OD) δ 7.26 (d, *J* = 8.5 Hz, 2H), 7.08 (d, *J* = 8.5 Hz, 2H), 4.65 (p, *J* = 7.8 Hz, 1H), 4.32–4.17 (m, 4H), 2.55 (t, *J* = 7.6 Hz, 2H), 1.57 (p, *J* = 7.3 Hz, 2H), 1.38–1.21 (m, 14H), 0.89 (t, *J* = 6.9 Hz, 3H). ^13^C NMR (101 MHz, CD_3_OD) δ 157.4, 138.8, 137.8, 129.7, 120.9, 54.5, 44.0, 36.2, 33.1, 32.8, 30.7, 30.7, 30.6, 30.4, 30.3, 23.7, 14.4. HRMS: (ESI) [M + H]^+^ calcd for C_20_H_34_N_3_O, 332.2696, observed, 332.2701.

#### (R)-1-(4-Decylphenyl)-3-(pyrrolidin-3-yl)urea Hydrochloride (**11c**)

Synthesized according to General Procedure 3. Purified via trituration with ethyl acetate and diethyl ether. White solid (76%, 65 mg). ^1^H NMR (400 MHz, CD_3_OD) δ 7.27 (d, *J* = 8.5 Hz, 2H), 7.08 (d, *J* = 8.5 Hz, 2H), 4.38 (tt, *J* = 6.8, 3.5 Hz, 1H), 3.57–3.45 (m, 2H), 3.40–3.29 (m, 2H), 2.55 (t, *J* = 7.6 Hz, 2H), 2.43–2.29 (m, 1H), 2.12–2.00 (m, 1H), 1.59 (p, *J* = 7.6 Hz, 2H), 1.37–1.25 (m, 14H), 0.91 (t, *J* = 6.9 Hz, 3H). ^13^C NMR (101 MHz, CD_3_OD) δ 158.0, 138.6, 138.0, 129.7, 120.6, 51.8, 50.8, 45.6, 36.2, 33.1, 32.8, 31.4, 30.7, 30.7, 30.6, 30.4, 30.3, 23.7, 14.4. HRMS: (ESI) [M + H]^+^ calcd for C_21_H_36_N_3_O, 346.2853, observed, 346.2854.

#### (S)-1-(4-Decylphenyl)-3-(pyrrolidin-3-yl)urea Hydrochloride (**11d**)

Synthesized according to General Procedure 3. Purified via trituration with ethyl acetate and diethyl ether. White solid (77%, 66 mg). ^1^H NMR (400 MHz, CD_3_OD) δ 7.27 (d, *J* = 8.5 Hz, 2H), 7.08 (d, *J* = 8.6 Hz, 2H), 4.44–4.33 (m, 1H), 3.57–3.45 (m, 2H), 3.40–3.28 (m, 2H), 2.55 (t, *J* = 7.6 Hz, 2H), 2.43–2.29 (m, 1H), 2.14–1.99 (m, 1H), 1.59 (p, *J* = 7.5 Hz, 2H), 1.36–1.26 (m, 14H), 0.91 (t, *J* = 6.8 Hz, 3H). ^13^C NMR (101 MHz, CD_3_OD) δ 157.9, 138.6, 138.0, 129.7, 120.7, 51.8, 50.8, 45.6, 36.2, 33.1, 32.8, 31.4, 30.7, 30.7, 30.6, 30.4, 30.3, 23.7, 14.4. HRMS: (ESI) [M + H]^+^ calcd for C_21_H_36_N_3_O, 346.2853, observed, 346.2867.

#### (R)-1-(4-Decylphenyl)-3-(piperidin-3-yl)urea Hydrochloride (**11e**)

Synthesized according to General Procedure 3. Purified via trituration with ethyl acetate and diethyl ether. White solid (57%, 49 mg). ^1^H NMR (400 MHz, CD_3_OD) δ 7.26 (d, *J* = 8.5 Hz, 2H), 7.06 (d, *J* = 8.5 Hz, 2H), 3.95 (tt, *J* = 9.9, 3.9 Hz, 1H), 3.42 (dd, *J* = 12.2, 4.1 Hz, 1H), 3.25 (dt, *J* = 12.9, 4.5 Hz, 1H), 3.04–2.87 (m, 2H), 2.53 (t, *J* = 7.6 Hz, 2H), 2.09–1.95 (m, 2H), 1.90–1.76 (m, 1H), 1.68–1.51 (m, 3H), 1.36–1.21 (m, 14H), 0.89 (t, *J* = 6.8 Hz, 3H). ^13^C NMR (101 MHz, CD_3_OD) δ 157.5, 138.5, 138.0, 129.7, 120.6, 48.7, 45.3, 44.8, 36.2, 33.0, 32.8, 30.7, 30.7, 30.6, 30.4, 30.3, 29.4, 23.7, 22.0, 14.5. HRMS: (ESI) [M + H]^+^ calcd for C_22_H_38_N_3_O, 360.3009, observed, 360.2998.

#### (S)-1-(4-Decylphenyl)-3-(piperidin-3-yl)urea Hydrochloride (**11f**)

Synthesized according to General Procedure 3. Purified via trituration with hexane. Off-white solid (73%, 63 mg). ^1^H NMR (400 MHz, CD_3_OD) δ 7.25 (d, *J* = 8.4 Hz, 2H), 7.07 (d, *J* = 8.5 Hz, 2H), 3.94 (tt, *J* = 9.9, 3.8 Hz, 1H), 3.43 (dd, *J* = 12.2, 3.9 Hz, 1H), 3.26 (dt, *J* = 12.9, 4.3 Hz, 1H), 3.04–2.87 (m, 2H), 2.53 (t, *J* = 7.6 Hz, 2H), 2.07–1.96 (m, 2H), 1.90–1.74 (m, 1H), 1.68–1.51 (m, 3H), 1.37–1.22 (m, 14H), 0.89 (t, *J* = 6.8 Hz, 3H). ^13^C NMR (101 MHz, CD_3_OD) δ 157.5, 138.6, 138.0, 129.7, 120.7, 48.7, 45.3, 44.8, 36.2, 33.0, 32.8, 30.7, 30.7, 30.6, 30.4, 30.3, 29.4, 23.7, 22.0, 14.4. HRMS: (ESI) [M + H]^+^ calcd for C_22_H_38_N_3_O, 360.3009, observed, 360.3005.

#### 4-Amino-*N*-(4-decylphenyl)piperidine-1-carboxamide Hydrochloride (**11g**)

Synthesized according to General Procedure 3. Purified via trituration with ethyl acetate and diethyl ether. White solid (86%, 140 mg). ^1^H NMR (400 MHz, CD_3_OD) δ 7.23 (d, *J* = 8.5 Hz, 2H), 7.09 (d, *J* = 8.4 Hz, 2H), 4.26 (dp, *J* = 14.2, 2.2 Hz, 2H), 3.36 (tt, *J* = 11.3, 4.1 Hz, 1H), 2.98 (ddd, *J* = 14.4, 12.3, 2.5 Hz, 2H), 2.56 (t, *J* = 7.6 Hz, 2H), 2.10–1.99 (m, 2H), 1.65–1.50 (m, 4H), 1.38–1.23 (m, 14H), 0.90 (t, *J* = 6.8 Hz, 3H). ^13^C NMR (101 MHz, CD_3_OD) δ 157.9, 139.2, 138.2, 129.5, 122.5, 49.8, 43.6, 36.3, 33.1, 32.8, 31.1, 30.7, 30.7, 30.6, 30.5, 30.3, 23.7, 14.4. HRMS: (ESI) [M + Na]^+^ calcd for C_22_H_38_N_3_NaO, 382.2829, observed, 382.2827.

#### 1-(4-Decylphenyl)-3-(piperidin-4-yl)urea Hydrochloride (**11h**)

Synthesized according to General Procedure 3. Purified via trituration with ethyl acetate and diethyl ether. White solid (82%, 71 mg). ^1^H NMR (400 MHz, CD_3_OD) δ 7.25 (d, *J* = 8.4 Hz, 2H), 7.06 (d, *J* = 8.4 Hz, 2H), 3.85 (tt, *J* = 10.2, 3.9 Hz, 1H), 3.41 (dt, *J* = 13.3, 4.1 Hz, 2H), 3.11 (td, *J* = 12.2, 3.2 Hz, 2H), 2.53 (t, *J* = 7.6 Hz, 2H), 2.15 (dq, *J* = 14.3, 3.9 Hz, 2H), 1.82–1.67 (m, 2H), 1.56 (p, *J* = 6.9 Hz, 2H), 1.37–1.17 (m, 14H), 0.89 (t, *J* = 6.7 Hz, 3H). ^13^C NMR (101 MHz, CD_3_OD) δ 157.6, 138.3, 138.2, 129.7, 120.5, 45.6, 44.0, 36.2, 33.1, 32.8, 30.7, 30.7, 30.6, 30.4, 30.3, 30.1, 23.7, 14.5. HRMS: (ESI) [M + H]^+^ calcd for C_22_H_38_N_3_O, 360.3009, observed, 360.3013.

#### *N*-(4-Decylphenyl)piperazine-1-carboxamide Hydrochloride (**11i**)

Synthesized according to General Procedure 3. Purified via trituration with ethyl acetate and diethyl ether. White solid (63%, 27 mg). ^1^H NMR (400 MHz, CD_3_OD) δ 7.26 (d, *J* = 8.4 Hz, 2H), 7.10 (d, *J* = 8.5 Hz, 2H), 3.81–3.74 (m, 4H), 3.29–3.24 (m, 4H), 2.56 (t, *J* = 7.6 Hz, 2H), 1.59 (p, *J* = 7.1 Hz, 2H), 1.37–1.22 (m, 14H), 0.90 (t, *J* = 6.9 Hz, 3H). ^13^C NMR (101 MHz, CD_3_OD) δ 157.6, 139.5, 137.9, 129.6, 122.4, 44.5, 42.4, 36.3, 33.1, 32.8, 30.7, 30.7, 30.6, 30.5, 30.3, 23.7, 14.4. HRMS: (ESI) [M + H]^+^ calcd for C_21_H_36_N_3_O, 346.2853, observed, 346.2852.

#### *N*-(4-Decylphenyl)-2,5-diazabicyclo[2.2.1]heptane-2-carboxamide Hydrochloride (**11j**)

Synthesized according to General Procedure 3. Purified via trituration with ethyl acetate and diethyl ether. White solid (81%, 70 mg). ^1^H NMR (400 MHz, CD_3_OD) δ 7.30 (d, *J* = 8.0 Hz, 2H), 7.07 (d, *J* = 8.1 Hz, 2H), 4.76 (brs, 1H), 4.39 (brs, 1H), 3.64 (brs, 2H), 3.43–3.27 (m, 2H), 2.53 (t, *J* = 7.6 Hz, 2H), 2.16–1.92 (m, 2H), 1.56 (p, *J* = 7.4 Hz, 2H), 1.37–1.16 (m, 14H), 0.88 (t, *J* = 6.7 Hz, 3H). ^13^C NMR (101 MHz, CD_3_OD) δ 156.5, 139.3, 137.8, 129.5, 122.4, 59.4, 56.5, 53.5, 51.7, 36.8, 36.3, 33.0, 32.8, 30.7, 30.7, 30.6, 30.4, 30.3, 23.7, 14.5. HRMS: (ESI) [M + H]^+^ calcd for C_22_H_36_N_3_O, 358.2853, observed, 358.2861.

#### *N*-(4-Decylphenyl)-3,8-diazabicyclo[3.2.1]octane-3-carboxamide Hydrochloride (**11k**)

Synthesized according to General Procedure 3. Purified via trituration with ethyl acetate and diethyl ether. White solid (87%, 75 mg). ^1^H NMR (400 MHz, CD_3_OD) δ 7.34 (d, *J* = 8.5 Hz, 2H), 7.12 (d, *J* = 8.5 Hz, 2H), 4.67–4.59 (m, 2H), 3.38–3.22 (m, 4H), 2.57 (t, *J* = 7.6 Hz, 2H), 2.26–2.20 (m, 2H), 2.12–2.03 (m, 2H), 1.60 (p, *J* = 7.1 Hz, 2H), 1.39–1.22 (m, 14H), 0.91 (t, *J* = 6.7 Hz, 3H). ^13^C NMR (101 MHz, CD_3_OD) δ 156.2, 139.4, 137.8, 129.6, 122.1, 53.4, 36.3, 33.1, 32.8, 30.7, 30.7, 30.6, 30.5, 30.3, 27.1, 23.7, 14.5. HRMS: (ESI) [M + H]^+^ calcd for C_23_H_38_N_3_O, 372.3009, observed, 372.3010.

#### (R)-*N*-(4-Decylphenyl)-3-methylpiperazine-1-carboxamide Hydrochloride (**11l**)

Synthesized according to General Procedure 3. Purified via trituration with ethyl acetate and diethyl ether. Light brown solid, 117 mg, 59% yield. ^1^H NMR (400 MHz, CD_3_OD) δ 7.28–7.24 (m, 2H), 7.12–7.08 (m, 2H), 4.30–4.23 (m, 2H), 3.46–3.40 (m, 1H), 3.40–3.34 (m, 1H), 3.29–3.23 (m, 1H), 3.20–3.11 (m, 1H), 3.04 (dd, *J* = 14.5, 10.6 Hz, 1H), 2.60–2.52 (m, 2H), 1.63–1.54 (m, 2H), 1.37 (d, *J* = 6.6 Hz, 3H), 1.34–1.26 (m, 14H), 0.94–0.85 (m, 3H). ^13^C NMR (151 MHz, CD_3_OD) δ 157.4, 139.4, 137.9, 129.6, 122.4, 52.5, 48.4, 44.3, 41.9, 36.3, 33.1, 32.8, 30.7, 30.7, 30.6, 30.4, 30.3, 23.7, 16.0, 14.4. HRMS: (ESI) [M + H]^+^ calcd for C_22_H_38_N_3_O^+^ 360.3009, observed, 360.3006.

#### *N*-(4-Decylphenyl)-1,4-diazepane-1-carboxamide Hydrochloride (**11m**)

Synthesized according to General Procedure 3. Purified via trituration with ethyl acetate and diethyl ether. Light brown solid, 19 mg, 74% yield. ^1^H NMR (600 MHz, CD_3_OD) δ 7.29–7.27 (m, 2H), 7.12–7.09 (m, 2H), 3.86–3.81 (m, 2H), 3.69 (t, *J* = 6.1 Hz, 2H), 3.37–3.33 (m, 4H), 2.59–2.54 (m, 2H), 2.17 (p, *J* = 6.0 Hz, 2H), 1.63–1.55 (m, 2H), 1.32–1.28 (m, 14H), 0.93–0.87 (m, 3H). ^13^C NMR (151 MHz, CD_3_OD) δ 156.6, 138.1, 136.6, 128.1, 121.7, 46.6, 45.3, 44.8, 42.1, 34.9, 31.7, 31.4, 29.4, 29.3, 29.2, 29.1, 28.9, 25.6, 22.3, 13.1. HRMS: (ESI) [M + H]^+^ calcd for C_22_H_38_N_3_O^+^ 360.3009, observed, 360.3018.

#### *tert*-Butyl (3-(4-Decyl-*N*-methylbenzamido)propyl)carbamate (**12a**)

Synthesized according to General Procedure 6. Purified via column chromatography (30–40% ethyl acetate/hexanes). Clear oil (72%, 74 mg). ^1^H NMR (400 MHz, CDCl_3_) δ 7.75 (d, *J* = 8.2 Hz, 2H), 7.28–7.18 (m, 3H), 5.07 (t, *J* = 6.9 Hz, 1H), 3.48 (q, *J* = 6.2 Hz, 2H), 3.21 (q, *J* = 6.5 Hz, 2H), 2.62 (t, *J* = 7.7 Hz, 2H), 1.68 (p, *J* = 6.0 Hz, 2H), 1.60 (p, *J* = 7.6 Hz, 2H), 1.44 (s, 9H), 1.37–1.15 (m, 14H), 0.87 (t, *J* = 7.0 Hz, 3H). ^13^C NMR (101 MHz, CDCl_3_) δ 167.7, 157.0, 146.8, 132.0, 128.6, 127.1, 79.5, 37.2, 36.2, 35.9, 32.0, 31.3, 30.4, 29.7, 29.7, 29.6, 29.4, 29.3, 28.5, 22.8, 14.2. HRMS: (ESI) [M + Na]^+^ calcd for C_25_H_42_N_2_NaO_3_, 441.3088, observed, 441.3080.

#### *tert*-Butyl (R)-3-(4-Decyl-*N*-methylbenzamido)pyrrolidine-1-carboxylate (**12b**)

Synthesized according to General Procedure 6. Purified via column chromatography (40–60% ethyl acetate/hexanes). Yellow (67%, 103 mg). ^1^H NMR (400 MHz, CDCl_3_) δ 7.29 (d, *J* = 8.1 Hz, 2H), 7.20 (d, *J* = 8.2 Hz, 2H), 5.30–4.33 (m, 1H), 3.68–3.46 (m, 2H), 3.42–3.17 (m, 2H), 2.94 (s, 3H), 2.62 (t, *J* = 7.5 Hz, 2H), 2.12–1.98 (m, 2H), 1.60 (p, *J* = 7.6 Hz, 2H), 1.44 (s, 9H), 1.37–1.19 (m, 14H), 0.87 (t, *J* = 6.8 Hz, 3H). ^13^C NMR (101 MHz, CDCl_3_) δ 172.5, 154.5, 145.0, 133.7, 128.7, 126.9, 79.8, 47.2, 46.8*, 44.6*, 44.2, 35.9, 32.0, 31.4, 29.7, 29.7, 29.6, 29.4, 29.4, 28.6, 28.4, 27.9*, 22.8, 14.2. Some carbon signals not observed due to peak broadening from adjacent heteroatoms. HRMS: (ESI) [M + H]^+^ calcd for C_27_H_45_N_2_O_3_, 445.3425, observed, 445.3419.

#### *tert*-Butyl (R)-3-(4-Decyl-*N*-methylbenzamido)piperidine-1-carboxylate (**12c**)

Synthesized according to General Procedure 6. Purified via column chromatography (30–50% ethyl acetate/hexanes). Clear oil (85%, 88 mg). ^1^H NMR (500 MHz, CDCl_3_) δ 7.28 (d, *J* = 8.1 Hz, 2H), 7.18 (d, *J* = 8.1 Hz, 2H), 4.54–3.40 (m, 3H), 3.02–2.76 (m, 4H), 2.63–2.42 (m, 3H), 1.97–1.54 (m, 6H), 1.40 (s, 9H), 1.32–1.21 (m, 14H), 0.86 (t, *J* = 7.0 Hz, 3H). ^13^C NMR (126 MHz, CDCl_3_) δ 172.4, 154.7, 144.7, 133.9, 128.6, 126.5, 79.9, 55.5, 50.82*, 47.7, 46.3*, 44.1*, 43.2, 35.9, 33.3, 32.0, 31.4, 29.7, 29.7, 29.6, 29.4, 29.4, 28.9, 28.4, 24.6, 22.8, 14.2. HRMS: (ESI) [M + Na]^+^ calcd for C_28_H_46_N_2_NaO_3_, 481.3401, observed, 481.3394.

#### *N*-(3-Aminopropyl)-4-decyl-*N*-methylbenzamide Hydrochloride (**13a**)

Synthesized according to General Procedure 3. Purified via trituration with ethyl acetate and diethyl ether. White solid (78%, 66 mg). ^1^H NMR (400 MHz, CD_3_OD) δ 7.41–7.37 (m, 2H), 7.31–7.27 (m, 2H), 3.68–3.62 (m, 2H), 3.08–3.02 (m, 4H), 2.66 (t, *J* = 7.7 Hz, 2H), 2.09–2.04 (m, 2H), 1.68–1.58 (m, 2H), 1.39–1.22 (m, 14H), 0.89 (t, *J* = 6.8 Hz, 3H). ^13^C NMR (101 MHz, CD_3_OD) δ 174.8, 146.7, 134.0, 129.6, 128.3, 47.8, 45.4, 38.2, 36.7, 33.6, 33.0, 32.5, 30.7, 30.6, 30.4, 30.3, 24.7, 23.7, 14.4. HRMS: (ESI) [M + H]^+^ calcd for C_21_H_37_N_2_O, 333.2900, observed, 333.2897.

#### (R)-4-Decyl-*N*-methyl-*N*-(pyrrolidin-3-yl)benzamide Hydrochloride (**13b**)

Synthesized according to General Procedure 3. Purified via trituration with ethyl acetate and diethyl ether. White solid (46%, 41 mg). ^1^H NMR (400 MHz, CD_3_OD) δ 7.38 (d, *J* = 8.1 Hz, 2H), 7.30 (d, *J* = 8.2 Hz, 2H), 4.68 (p, *J* = 7.5 Hz, 1H), 3.73–3.45 (m, 3H), 3.30–3.20 (m, 1H), 3.04 (s, 3H), 2.66 (t, *J* = 7.7 Hz, 2H), 2.52–2.23 (m, 2H), 1.63 (p, *J* = 7.6 Hz, 2H), 1.38–1.21 (m, 14H), 0.90 (t, *J* = 6.9 Hz, 3H). ^13^C NMR (101 MHz, CD_3_OD) δ 174.7, 146.9, 134.4, 129.7, 128.2, 58.3, 46.7, 36.7, 33.0, 32.5, 30.7, 30.7, 30.6, 30.4, 30.3, 28.5, 23.7, 14.4. Some carbon signals not observed due to peak broadening from adjacent heteroatoms. HRMS: (ESI) [M + H]^+^ calcd for C_22_H_37_N_2_O, 345.2900, observed, 345.2911.

#### (R)-4-Decyl-*N*-Methyl-*N*-(piperidin-3-yl)benzamide Hydrochloride (**13c**)

Synthesized according to General Procedure 3. Purified via trituration with ethyl acetate and diethyl ether. White solid (80%, 61 mg). ^1^H NMR (400 MHz, CD_3_OD) δ 7.37 (d, *J* = 8.1 Hz, 2H), 7.30 (d, *J* = 8.2 Hz, 2H), 4.74–3.91 (m, 1H), 3.49–3.19 (m, 3H), 3.03–2.88 (m, 4H), 2.66 (t, *J* = 7.7 Hz, 2H), 2.20–1.80 (m, 4H), 1.64 (p, *J* = 7.3 Hz, 2H), 1.42–1.22 (m, 14H), 0.90 (t, *J* = 6.6 Hz, 3H). ^13^C NMR (126 MHz, CD_3_OD) δ 174.5, 146.8, 134.3, 129.7, 128.2, 50.7, 45.5, 44.7, 36.7, 34.0, 33.1, 32.5, 30.7, 30.7, 30.6, 30.5, 30.3, 26.5, 23.7, 23.0, 14.5. HRMS: (ESI) [M + H]^+^ calcd for C_23_H_39_N_2_O, 359.3057, observed, 359.3057.

#### *tert*-Butyl (3-(4-Decylphenylthioamido)propyl)carbamate (**14a**)

Synthesized according to General Procedure 7. Purified via column chromatography (15–35% ethyl acetate/hexanes). Yellow oil (72%, 75 mg). ^1^H NMR (500 MHz, CDCl_3_) δ 8.95 (brs, 1H), 7.81 (d, *J* = 8.2 Hz, 2H), 7.20 (d, *J* = 8.5 Hz, 2H), 4.84 (t, *J* = 6.7 Hz, 1H), 3.95 (q, *J* = 6.0 Hz, 2H), 3.24 (q, *J* = 6.6 Hz, 2H), 2.62 (t, *J* = 7.7 Hz, 2H), 1.86 (p, *J* = 6.0 Hz, 2H), 1.60 (p, *J* = 7.6 Hz, 2H), 1.45 (s, 9H), 1.33–1.22 (m, 14H), 0.88 (t, *J* = 6.9 Hz, 3H). ^13^C NMR (126 MHz, CDCl_3_) δ 198.5, 157.5, 146.7, 138.9, 128.6, 127.1, 80.1, 42.8, 37.4, 35.9, 32.0, 31.4, 29.8, 29.7, 29.6, 29.5, 29.4, 28.8, 28.5, 22.8, 14.3. HRMS: (ESI) [M + H]^+^ calcd for C_25_H_43_N_2_O_2_S, 435.3040, observed, 435.3048.

#### *tert*-Butyl (R)-3-(4-Decylphenylthioamido)pyrrolidine-1-carboxylate (**14b**)

Synthesized according to General Procedure 7. Purified via column chromatography (30% ethyl acetate/hexanes). Yellow oil (68%, 151 mg). ^1^H NMR (400 MHz, CDCl_3_) δ 7.86 (d, *J* = 38.3 Hz, 1H), 7.63 (d, *J* = 8.3 Hz, 2H), 7.15 (d, *J* = 8.2 Hz, 2H), 5.17–5.03 (m, 1H), 3.80–3.63 (m, 1H), 3.53–3.30 (m, 3H), 2.60 (t, *J* = 7.7 Hz, 2H), 2.35–2.22 (m, 1H), 2.19–2.00 (m, 1H), 1.58 (p, *J* = 7.2 Hz, 2H), 1.43 (s, 9H), 1.34–1.20 (m, 14H), 0.86 (t, *J* = 6.8 Hz, 3H). ^13^C NMR (101 MHz, CDCl_3_) δ 199.3, 154.5, 146.9, 139.1, 128.5, 126.9, 79.9, 56.0, 55.4*, 51.0, 50.5*, 44.3*, 44.0, 35.8, 31.9, 31.3, 31.1*, 30.3, 29.7, 29.6, 29.5, 29.4, 29.3, 28.5, 22.7, 14.2. Material isolated as an approximately 1:1 ratio of rotamers. HRMS: (ESI) [M + Na]^+^ calcd for C_26_H_42_N_2_NaO_2_S, 469.2859, observed, 469.2830.

#### *tert*-Butyl (R)-3-(4-Decylphenylthioamido)piperidine-1-carboxylate (**14c**)

Synthesized according to General Procedure 7. Purified via column chromatography (30% ethyl acetate/hexanes). Yellow (80%, 83 mg). ^1^H NMR (500 MHz, CDCl_3_) δ 7.68–7.61 (m, 3H), 7.16 (d, *J* = 8.2 Hz, 2H), 4.71–4.62 (m, 1H), 4.02–3.08 (m, 4H), 2.61 (t, *J* = 7.6 Hz, 2H), 2.39–1.76 (m, 2H), 1.65–1.53 (m, 4H), 1.44 (s, 9H), 1.35–1.18 (m, 14H), 0.87 (t, *J* = 7.0 Hz, 3H). ^13^C NMR (126 MHz, CDCl_3_) δ 198.3, 155.1, 146.8, 139.3, 128.6, 126.7, 80.4, 51.4, 47.8, 45.2*, 43.8, 35.8, 32.0, 31.3, 29.7, 29.7, 29.5, 29.4, 29.3, 28.4, 27.7, 22.8, 22.3, 14.2. Carbon signal peak broadening observed due to the presence of rotamers. HRMS: (ESI) [M + H]^+^ calcd for C_27_H_45_N_2_O_2_S, 461.3196, observed, 461.3192.

#### *N*-(3-Aminopropyl)-4-decylbenzothioamide Hydrochloride (**15a**)

Synthesized according to General Procedure 3. Purified via trituration with hexanes. Yellow solid (90%, 58 mg). ^1^H NMR (400 MHz, CD_3_OD) δ 7.73 (d, *J* = 8.2 Hz, 2H), 7.22 (d, *J* = 8.2 Hz, 2H), 3.95 (t, *J* = 6.7 Hz, 2H), 3.04 (t, *J* = 7.5 Hz, 2H), 2.65 (t, *J* = 7.6 Hz, 2H), 2.13 (p, *J* = 6.9 Hz, 2H), 1.63 (p, *J* = 7.1 Hz, 2H), 1.37–1.21 (m, 14H), 0.89 (t, *J* = 6.9 Hz, 3H). ^13^C NMR (101 MHz, CD_3_OD) δ 201.0, 147.7, 140.3, 129.2, 128.3, 43.8, 38.5, 36.6, 33.0, 32.4, 30.7, 30.7, 30.6, 30.4, 30.3, 27.5, 23.7, 14.4. HRMS: (ESI) [M + H]^+^ calcd for C_20_H_35_N_2_S, 335.2515, observed, 335.2514

#### (R)-4-Decyl-*N*-(pyrrolidin-3-yl)benzothioamide Hydrochloride (**15b**)

Synthesized according to General Procedure 3. Purified via trituration with hexanes. Yellow solid (94%, 122 mg). ^1^H NMR (400 MHz, CD_3_OD) δ 7.77 (d, *J* = 8.2 Hz, 2H), 7.23 (d, *J* = 8.3 Hz, 2H), 5.22 (tt, *J* = 7.2, 4.9 Hz, 1H), 3.77 (dd, *J* = 12.5, 7.1 Hz, 1H), 3.63–3.39 (m, 3H), 2.66 (t, *J* = 7.6 Hz, 2H), 2.50 (dq, *J* = 13.7, 7.5 Hz, 1H), 2.40–2.27 (m, 1H), 1.64 (p, *J* = 7.1 Hz, 2H), 1.39–1.22 (m, 14H), 0.91 (t, *J* = 6.9 Hz, 3H). ^13^C NMR (101 MHz, CD_3_OD) δ 201.8, 147.8, 140.1, 129.2, 128.6, 56.2, 50.6, 45.9, 36.6, 33.0, 32.4, 30.7, 30.7, 30.6, 30.4, 30.7, 30.7, 23.7, 14.5. HRMS: (ESI) [M + H]^+^ calcd for C_21_H_35_N_2_S, 347.2515, observed, 347.2526.

#### (R)-4-Decyl-*N*-(piperidin-3-yl)benzothioamide Hydrochloride (**15c**)

Synthesized according to General Procedure 3. Purified via trituration with diethyl ether. White solid (88%, 60 mg). ^1^H NMR (400 MHz, CD_3_OD) δ 7.72 (d, *J* = 6.7 Hz, 2H), 7.22 (d, *J* = 8.1 Hz, 2H), 4.98 (dt, *J* = 10.8, 5.5 Hz, 1H), 3.75 (dd, *J* = 12.1, 4.4 Hz, 1H), 3.39 (d, *J* = 12.8 Hz, 1H), 3.01 (q, *J* = 12.4 Hz, 2H), 2.65 (t, *J* = 7.6 Hz, 2H), 2.29–2.03 (m, 2H), 1.94–1.79 (m, 2H), 1.64 (p, *J* = 7.1 Hz, 2H), 1.38–1.26 (m, 14H), 0.91 (t, *J* = 6.9 Hz, 3H). ^13^C NMR (101 MHz, CD_3_OD) δ 201.4, 147.8, 140.4, 129.2, 128.6, 51.1, 46.4, 44.9, 36.6, 33.0, 32.4, 30.7, 30.7, 30.6, 30.4, 30.3, 28.1, 23.7, 22.4, 14.4. HRMS: (ESI) [M + H]^+^ calcd for C_22_H_37_N_2_S, 361.2672, observed, 361.2678.

#### *tert*-Butyl 4-((4-Decylphenyl)(methyl)carbamoyl)piperazine-1-carboxylate (**16a**)

Synthesized according to General Procedure 6. Purified via column chromatography (30–40% ethyl acetate/hexanes). Clear oil (51%, 53 mg). ^1^H NMR (400 MHz, CDCl_3_) δ 7.12 (d, *J* = 8.4 Hz, 2H), 6.99 (d, *J* = 8.4 Hz, 2H), 3.23–3.10 (m, 11H), 2.55 (t, *J* = 7.8 Hz, 2H), 1.58 (p, *J* = 7.6 Hz, 2H), 1.40 (s, 9H), 1.34–1.22 (m, 14H), 0.86 (t, *J* = 6.5 Hz, 3H). ^13^C NMR (101 MHz, CDCl_3_) δ 161.4, 154.7, 144.3, 140.0, 129.5, 124.2, 80.0, 45.6, 43.1, 39.9, 35.4, 32.0, 31.5, 29.7, 29.7, 29.6, 29.4, 29.4, 28.5, 22.8, 14.2. HRMS: (ESI) [M + Na]^+^ calcd for C_27_H_45_N_3_NaO_3_, 482.3353, observed, 482.3361.

#### *tert*-Butyl 5-((4-Decylphenyl)(methyl)carbamoyl)-2,5-diazabicyclo[2.2.1]heptane-2-carboxylate (**16b**)

Synthesized according to General Procedure 6. Purified via column chromatography (30–40% ethyl acetate/hexanes). White solid (67%, 69 mg). ^1^H NMR (400 MHz, CDCl_3_) δ 7.13–7.04 (m, 2H), 7.04–6.97 (m, 2H), 4.47–4.12 (m, 2H), 3.48–3.35 (m, 1H), 3.23–3.11 (m, 4H), 2.88–2.39 (m, 4H), 1.68–1.49 (m, 4H), 1.39 (s, 9H), 1.32–1.17 (m, 17H), 0.84 (t, *J* = 6.7 Hz, 3H). ^13^C NMR (101 MHz, CDCl_3_) δ 159.8, 159.5*, 154.2, 143.2*, 142.9, 140.6, 140.4*, 129.4, 126.0, 125.7*, 79.6, 58.4, 57.9*, 57.4*, 56.5, 56.3, 56.0*, 53.0, 52.6*, 39.8*, 39.7, 36.6*, 36.0, 35.4, 31.9, 31.4, 29.6, 29.6, 29.5, 29.4, 29.3, 28.5, 22.7, 14.2. Material isolated as an approximately 2:1 ratio of rotamers. HRMS: (ESI) [M + H]+ calcd for C_28_H_46_N_3_O_3_, 472.3525.

#### *tert*-Butyl 3-((4-Decylphenyl)(methyl)carbamoyl)-3,8-diazabicyclo[3.2.1]octane-8-carboxylate (**16c**)

Synthesized according to General Procedure 6. Purified via column chromatography (30–50% ethyl acetate/hexanes). Clear oil (82%, 84 mg). ^1^H NMR (400 MHz, CDCl_3_) δ 7.17–7.05 (m, 4H), 3.96–3.88 (m, 2H), 3.52 (dd, *J* = 37.7, 11.6 Hz, 2H), 3.21 (s, 3H), 2.79 (dd, *J* = 57.1, 12.4 Hz, 2H), 2.56 (t, *J* = 7.8 Hz, 2H), 1.76–1.65 (m, 2H), 1.64–1.49 (m, 4H), 1.40 (s, 9H), 1.34–1.17 (m, 14H), 0.87 (t, *J* = 6.8 Hz, 3H). ^13^C NMR (101 MHz, CDCl_3_) δ 159.8, 156.0, 144.1, 140.5, 129.5, 125.2, 79.7, 54.9, 54.4*, 49.6*, 48.4, 39.7, 35.5, 32.0, 31.5, 29.7, 29.7, 29.6, 29.4, 29.3, 28.5, 26.8, 22.8, 14.2. HRMS: (ESI) [M + H]+ calcd for C_29_H_48_N_3_O_3_, 486.3690, observed, 486.3696.

#### *N*-(4-Decylphenyl)-*N*-methylpiperazine-1-carboxamide Hydrochloride (**17a**)

Synthesized according to General Procedure 3. Purified via trituration with diethyl ether and ethyl acetate. White solid (85%, 39 mg). ^1^H NMR (400 MHz, CD_3_OD) δ 7.24 (d, *J* = 8.5 Hz, 2H), 7.13 (d, *J* = 8.5 Hz, 2H), 3.45–3.38 (m, 4H), 3.20 (s, 3H), 3.05–2.98 (m, 4H), 2.61 (t, *J* = 7.7 Hz, 2H), 1.61 (p, *J* = 7.1 Hz, 2H), 1.38–1.23 (m, 14H), 0.90 (t, *J* = 6.9 Hz, 3H). ^13^C NMR (101 MHz, CD_3_OD) δ 162.42, 144.64, 142.06, 130.90, 125.63, 44.00, 43.79, 40.18, 36.28, 33.05, 32.66, 30.72, 30.71, 30.58, 30.44, 30.33, 23.72, 14.45. HRMS: (ESI) [M + H]^+^ calcd for C_22_H_38_N_3_O, 360.3009, observed, 360.3014.

#### *N*-(4-Decylphenyl)-*N*-methyl-2,5-diazabicyclo[2.2.1]heptane-2-carboxamide Hydrochloride (**17b**)

Synthesized according to General Procedure 3. Purified via trituration with diethyl ether and ethyl acetate. White solid (50%, 43 mg). ^1^H NMR (400 MHz, CD_3_OD) δ 7.24 (d, *J* = 8.4 Hz, 2H), 7.14 (d, *J* = 8.4 Hz, 2H), 4.46 (s, 1H), 4.09 (t, *J* = 2.3 Hz, 1H), 3.35 (dd, *J* = 11.1, 1.9 Hz, 1H), 3.20 (s, 3H), 3.14 (dd, *J* = 11.0, 2.1 Hz, 1H), 3.07 (dd, *J* = 11.1, 1.6 Hz, 1H), 2.72 (dd, *J* = 11.2, 2.5 Hz, 1H), 2.61 (t, *J* = 7.7 Hz, 2H), 1.93–1.75 (m, 2H), 1.61 (p, *J* = 7.5 Hz, 2H), 1.38–1.23 (m, 14H), 0.90 (t, *J* = 6.7 Hz, 3H). ^13^C NMR (126 MHz, CD_3_OD) δ 161.1, 143.4, 142.7, 130.9, 127.2, 59.3, 58.1, 53.6, 52.4, 40.0, 36.3, 35.9, 33.1, 32.7, 30.7, 30.7, 30.6, 30.5, 30.3, 23.7, 14.5. HRMS: (ESI) [M + H]^+^ calcd for C_23_H_36_N_3_O, 372.3009, observed, 372.3002.

#### *N*-(4-Decylphenyl)-*N*-methyl-3,8-diazabicyclo[3.2.1]octane-3-carboxamide Hydrochloride (**17c**)

Synthesized according to General Procedure 3. Purified via trituration with ethyl acetate and diethyl ether. White solid (92%, 67 mg). ^1^H NMR (400 MHz, CD_3_OD) δ 7.30–7.18 (m, 4H), 4.10–4.03 (m, 2H), 3.23 (s, 3H), 3.06–2.98 (m, 4H), 2.62 (t, *J* = 7.7 Hz, 2H), 2.03–1.81 (m, 4H), 1.61 (p, *J* = 7.1 Hz, 2H), 1.38–1.24 (m, 14H), 0.90 (t, *J* = 6.9 Hz, 3H). ^13^C NMR (101 MHz, CD_3_OD) δ 160.6, 144.3, 142.7, 130.9, 126.5, 54.5, 48.6, 39.9, 36.3, 33.1, 32.6, 30.7, 30.7, 30.6, 30.4, 30.3, 26.8, 23.7, 14.4. HRMS: (ESI) [M + H]+ calcd for C_24_H_40_N_3_O, 386.3166, observed, 386.3164.

#### *N*-(4-Decylphenyl)-4-methylpiperazine-1-carboxamide Hydrochloride (**18a**)

A solution of **11i** in MeOH was added to a pressure vial charged with a stir bar. Paraformaldehyde (10.0 equiv) and formic acid (10.0 equiv) were added. The vial was sealed, purged with N_2_, and heated to 65 °C for 6 h. Upon complete consumption of starting material as monitored by TLC, the mixture was cooled to room temperature. The reaction was concentrated under reduced pressure and the residue was dissolved in ethyl acetate. The solution was partitioned with saturated sodium carbonate and the aqueous layer was rinsed three times with ethyl acetate. The combined organic layers were further rinsed twice with saturated sodium carbonate and thrice with brine. The organic solvent was removed under reduced pressure to yield the free amine base, which was subjected to column chromatography (5–15% methanol/dichloromethane). The resulting white solid free amine base was treated with hydrochloric acid to yield the tertiary ammonium chloride salt. The material was purified via trituration with ethyl acetate and diethyl ether. White solid (50%, 33 mg). ^1^H NMR (400 MHz, CD_3_OD) δ 7.27 (d, *J* = 8.5 Hz, 2H), 7.10 (d, *J* = 8.6 Hz, 2H), 4.44–4.25 (m, 2H), 3.61–3.47 (m, 2H), 3.38–3.25 (m, 2H), 3.20–3.06 (m, 2H), 2.94 (s, 3H), 2.56 (t, *J* = 7.6 Hz, 2H), 1.58 (p, *J* = 7.6 Hz, 2H), 1.38–1.21 (m, 14H), 0.90 (t, *J* = 6.8 Hz, 3H). ^13^C NMR (101 MHz, CD_3_OD) δ 157.4, 139.5, 137.9, 129.6, 122.4, 54.3, 43.6, 42.7, 36.3, 33.1, 32.8, 30.7, 30.7, 30.6, 30.4, 30.3, 23.7, 14.4. HRMS: (ESI) [M + H]^+^ calcd for C_22_H_38_N_3_O, 360.3009, observed, 360.3017.

#### 4-((4-Decylphenyl)carbamoyl)-1,1-dimethylpiperazin-1-ium Iodide (**18b**)

An oven-dried round-bottom flask containing a stir bar, K_2_CO_3_ (4.0 equiv), and MeI (10.0 equiv) was purged with N_2_. A dispersion of **11i** (1.0 equiv) in anhydrous MeCN was syringed into the flask and the solution was allowed to stir at room temperature for 16 h. The crude material was concentrated under reduced pressure and subjected to silica gel chromatography. Purified via column chromatography (10–15% MeOH/DCM). Purified via column chromatography (10–15% methanol/dichloromethane). White solid (83%, 65 mg). ^1^H NMR (400 MHz, CD_3_OD) δ 7.28 (d, *J* = 8.5 Hz, 2H), 7.10 (d, *J* = 8.6 Hz, 2H), 3.92 (t, *J* = 5.3 Hz, 4H), 3.55 (t, *J* = 4.7 Hz, 4H), 3.29 (s, 6H), 2.56 (t, *J* = 7.5 Hz, 2H), 1.59 (p, *J* = 7.4 Hz, 2H), 1.36–1.23 (m, 14H), 0.90 (t, *J* = 6.7 Hz, 3H). ^13^C NMR (101 MHz, CD_3_OD) δ 157.4, 139.5, 137.9, 129.6, 122.5, 62.3, 52.0, 39.5, 36.2, 33.0, 32.8, 30.7, 30.7, 30.6, 30.4, 30.3, 23.7, 14.4. HRMS: (ESI) [M + H]^+^ calcd for C_23_H_40_N_3_O, 374.3166, observed, 374.3170.

#### *tert*-Butyl 4-((4-Hexylphenyl)carbamoyl)piperazine-1-carboxylate (**19a**)

Synthesized according to General Procedure 2. Purified via column chromatography (20–40% ethyl acetate/hexanes). White solid (84%, 57 mg). ^1^H NMR (500 MHz, CDCl_3_) δ 7.23 (d, *J* = 8.1 Hz, 2H), 7.09 (d, *J* = 8.0 Hz, 2H), 6.39 (brs, 1H), 3.47 (d, *J* = 2.9 Hz, 8H), 2.58–2.50 (m, 2H), 1.63–1.51 (m, 2H), 1.47 (s, 9H), 1.35–1.23 (m, 6H), 0.92–0.82 (m, 3H). ^13^C NMR (126 MHz, CDCl_3_) δ 155.3, 154.8, 138.3, 136.4, 128.9, 120.4, 80.4, 35.4, 31.8, 31.6, 29.0, 28.5, 22.7, 14.2. HRMS: (ESI) [M+NH_4_]^+^ calcd for C_22_H_40_N_4_O_3_, 407.3017, observed, 407.3028.

#### *tert*-Butyl 4-((4-Heptylphenyl)carbamoyl)piperazine-1-carboxylate (**19b**)

Synthesized according to General Procedure 2. Purified via column chromatography (20–40% ethyl acetate/hexanes). White solid (48%, 82 mg). ^1^H NMR (500 MHz, CDCl_3_) δ 7.26–7.19 (m, 2H), 7.14–7.05 (m, 2H), 6.31 (brs, 1H), 3.54–3.37 (m, 8H), 2.54 (dd, *J* = 8.6, 6.8 Hz, 2H), 1.68–1.52 (m, 2H), 1.48 (s, 9H), 1.35–1.18 (m, 8H), 0.93–0.82 (m, 3H). ^13^C NMR (126 MHz, CDCl_3_) δ 155.3, 154.8, 138.3, 136.3, 129.0, 120.4, 80.4, 35.4, 32.0, 31.7, 29.3, 29.3, 28.5, 22.8, 14.2. HRMS: (ESI) [M + H]^+^ calcd for C_23_H_38_N_3_O_3_, 404.2908, observed, 402.2902.

#### *tert*-Butyl 4-((4-Octylphenyl)carbamoyl)piperazine-1-carboxylate (**19c**)

Synthesized according to General Procedure 2. Purified via column chromatography (20–40% ethyl acetate/hexanes). White solid (60%, 87 mg). ^1^H NMR (600 MHz, CDCl_3_) δ 7.25–7.20 (m, 2H), 7.15–7.01 (m, 2H), 6.33 (brs, 1H), 3.55–3.40 (m, 8H), 2.58–2.49 (m, 2H), 1.60–1.52 (m, 2H), 1.48 (s, 9H), 1.34–1.19 (m, 10H), 0.87 (t, *J* = 7.0 Hz, 3H).^13^C NMR (151 MHz, CDCl_3_) δ 155.3, 154.8, 138.3, 136.3, 129.0, 120.4, 80.4, 35.4, 32.0, 31.7, 29.6, 29.4, 29.4, 28.5, 22.8, 14.3. HRMS: (ESI) [M + H]^+^ calcd for C_24_H_40_N_3_O_3_, 418.3064, observed, 418.3073

#### *tert*-Butyl 4-((4-Nonylphenyl)carbamoyl)piperazine-1-carboxylate (**19d**)

Synthesized according to General Procedure 2. Purified via column chromatography (20–40% ethyl acetate/hexanes). White solid (59%, 88 mg). ^1^H NMR (600 MHz, CDCl_3_) δ 7.23 (d, *J* = 8.4 Hz, 2H), 7.09 (d, *J* = 8.4 Hz, 2H), 6.37 (brs, 1H), 3.47 (d, *J* = 5.3 Hz, 8H), 2.58–2.49 (m, 2H), 1.60–1.53 (m, 2H), 1.47 (s, 9H), 1.30–1.25 (m, 12H), 0.87 (t, *J* = 7.0 Hz, 3H). ^13^C NMR (151 MHz, CDCl_3_) δ 155.3, 154.8, 138.3, 136.3, 128.9, 120.4, 80.4, 35.4, 32.0, 31.7, 29.7, 29.6, 29.5, 29.4, 28.5, 22.8, 14.3. HRMS: (ESI) [M + H]^+^ calcd for C_25_H_42_N_3_O_3_, 432.3221, observed, 432.3221.

#### *tert*-Butyl 4-((4-Undecylphenyl)carbamoyl)piperazine-1-carboxylate (**19e**)

Synthesized according to General Procedure 2. Purified via column chromatography (20–40% ethyl acetate/hexanes). White solid (92%, 117 mg). ^1^H NMR (600 MHz, CDCl_3_) δ 7.25–7.19 (m, 2H), 7.15–7.05 (m, 2H), 6.34 (brs, 1H), 3.52–3.40 (m, 8H), 2.54 (dd, *J* = 8.7, 6.8 Hz, 2H), 1.61–1.51 (m, 2H), 1.48 (s, 9H), 1.33–1.18 (m, 16H), 0.87 (t, *J* = 7.0 Hz, 3H). ^13^C NMR (151 MHz, CDCl_3_) δ 155.3, 154.8, 138.3, 136.3, 129.0, 120.4, 80.4, 35.4, 32.1, 31.7, 29.8, 29.8, 29.7, 29.7, 29.5, 29.4, 28.5, 22.8, 14.3. HRMS: (ESI) [M + H]^+^ calcd for C_27_H_46_N_3_O_3_, 460.3534, observed, 460.3529.

#### *tert*-Butyl 4-((4-Dodecylphenyl)carbamoyl)piperazine-1-carboxylate (**19f**)

Synthesized according to General Procedure 2. Purified via column chromatography (20–40% ethyl acetate/hexanes). White solid (67%, 146 mg). ^1^H NMR (600 MHz, CDCl_3_) δ 7.26–7.20 (m, 2H), 7.15–7.05 (m, 2H), 6.30 (brs, 1H), 3.48 (q, *J* = 5.1, 4.6 Hz, 8H), 2.59–2.48 (m, 2H), 1.56 (td, *J* = 9.9, 8.9, 5.5 Hz, 2H), 1.48 (s, 9H), 1.33–1.18 (m, 18H), 0.87 (t, *J* = 7.0 Hz, 3H). ^13^C NMR (151 MHz, CDCl_3_) δ 155.3, 154.8, 138.3, 136.3, 129.0, 120.3, 80.4, 77.4, 77.2, 76.9, 35.4, 32.1, 31.7, 29.8, 29.8, 29.8, 29.8, 29.7, 29.5, 29.4, 28.5, 22.8, 14.3. HRMS: (ESI) [M + H]^+^ calcd for C_28_H_48_N_3_O_3_, 474.3690, observed, 474.3677.

#### *tert*-Butyl 4-((4-Tridecylphenyl)carbamoyl)piperazine-1-carboxylate (**19g**)

Synthesized according to General Procedure 2. Purified via column chromatography (20–40% ethyl acetate/hexanes). White solid (67%, 151 mg). ^1^H NMR (600 MHz, CDCl_3_) δ 7.26–7.19 (m, 2H), 7.14–7.05 (m, 2H), 6.30 (brs, 1H), 3.48 (p, *J* = 6.3, 4.9 Hz, 8H), 2.54 (dd, *J* = 8.6, 6.8 Hz, 2H), 1.56 (dt, *J* = 14.8, 6.7 Hz, 2H), 1.48 (s, 9H), 1.33–1.18 (m, 20H), 0.87 (t, *J* = 7.0 Hz, 3H). ^13^C NMR (151 MHz, CDCl_3_) δ 155.2, 154.8, 138.3, 136.3, 129.0, 120.3, 80.4, 35.4, 32.1, 31.7, 29.8, 29.8, 29.8, 29.8, 29.8, 29.7, 29.5, 29.4, 28.5, 22.8, 14.3. HRMS: (ESI) [M + H]^+^ calcd for C_29_H_50_N_3_O_3_, 488.3847, observed, 488.3863.

#### *N*-(4-Hexylphenyl)piperazine-1-carboxamide Hydrogen Chloride (**20a**)

Synthesized according to General Procedure 3. Purified via trituration with ethyl acetate and diethyl ether. White solid (90%, 15 mg). ^1^H NMR (500 MHz, CD_3_OD) δ 7.29–7.21 (m, 2H), 7.16–7.05 (m, 2H), 3.83–3.72 (m, 4H), 3.30–3.24 (m, 4H), 2.60–2.52 (m, 2H), 1.59 (p, *J* = 7.6 Hz, 2H), 1.38–1.26 (m, 6H), 0.89 (t, *J* = 6.9 Hz, 3H). ^13^C NMR (126 MHz, CD_3_OD) δ 157.6, 139.5, 137.9, 129.6, 122.4, 44.5, 42.4, 36.3, 32.9, 32.8, 30.0, 23.7, 14.4. HRMS: (ESI) [M + H]^+^ calcd for C_17_H_28_N_3_O, 290.2227, observed, 290.2214.

#### *N*-(4-Heptylphenyl)piperazine-1-carboxamide Hydrogen Chloride (**20b**)

Synthesized according to General Procedure 3. Purified via trituration with ethyl acetate and diethyl ether. White solid (88%, 59 mg). ^1^H NMR (500 MHz, CD_3_OD) δ 7.31–7.20 (m, 2H), 7.14–7.06 (m, 2H), 3.91–3.55 (m, 4H), 3.30–3.25 (m, 4H), 2.61–2.50 (m, 2H), 1.59 (p, *J* = 7.1 Hz, 2H), 1.37–1.23 (m, 8H), 0.89 (t, *J* = 7.0 Hz, 3H). ^13^C NMR (126 MHz, CD_3_OD) δ 157.6, 139.5, 137.9, 129.6, 122.4, 44.5, 42.4, 36.3, 33.0, 32.8, 30.3, 30.2, 23.7, 14.4. HRMS: (ESI) [M + H]^+^ calcd for C_18_H_30_N_3_O, 304.2383, observed, 304.2390.

#### *N*-(4-Octylphenyl)piperazine-1-carboxamide Hydrogen Chloride (**20c**)

Synthesized according to General Procedure 3. Purified via trituration with ethyl acetate and diethyl ether. White solid (83%, 49 mg). ^1^H NMR (500 MHz, CD_3_OD) δ 7.29–7.23 (m, 2H), 7.16–7.06 (m, 2H), 3.78 (dd, *J* = 6.1, 4.6 Hz, 4H), 3.30–3.24 (m, 4H), 2.60–2.52 (m, 2H), 1.59 (p, *J* = 7.3 Hz, 2H), 1.39–1.19 (m, 10H), 0.89 (t, *J* = 7.0 Hz, 3H). ^13^C NMR (126 MHz, CD_3_OD) δ 157.6, 139.5, 137.9, 129.6, 122.4, 44.5, 42.4, 36.3, 33.0, 32.8, 30.6, 30.4, 30.3, 23.7, 14.4. HRMS: (ESI) [M + H]^+^ calcd for C_19_H_32_N_3_O, 318.2540, observed, 318.2532.

#### *N*-(4-Nonylphenyl)piperazine-1-carboxamide Hydrogen Chloride (**20d**)

Synthesized according to General Procedure 3. Purified via trituration with ethyl acetate and diethyl ether. White solid (86%, 22 mg). ^1^H NMR (500 MHz, CD_3_OD) δ 7.25 (d, *J* = 8.4 Hz, 2H), 7.11 (d, *J* = 8.4 Hz, 2H), 3.84–3.66 (m, 4H), 3.29–3.24 (m, 4H), 2.56 (t, *J* = 7.6 Hz, 2H), 1.63–1.55 (m, 2H), 1.34–1.26 (m, 12H), 0.90 (t, *J* = 6.9 Hz, 3H). ^13^C NMR (126 MHz, CD_3_OD) δ 157.6, 139.5, 137.9, 129.6, 122.4, 44.5, 42.4, 36.3, 33.1, 32.8, 30.7, 30.6, 30.4, 30.3, 23.7, 14.4. HRMS: (ESI) [M + H]^+^ calcd for C_20_H_34_N_3_O, 332.2696, observed, 332.9708.

#### *N*-(4-Undecylphenyl)piperazine-1-carboxamide Hydrogen Chloride (**20e**)

Synthesized according to General Procedure 3. Purified via trituration with ethyl acetate and diethyl ether. White solid (91%, 92 mg). ^1^H NMR (500 MHz, CD_3_OD) δ 7.31–7.20 (m, 2H), 7.16–7.04 (m, 2H), 3.82–3.72 (m, 4H), 3.31–3.23 (m, 4H), 2.63–2.48 (m, 2H), 1.59 (p, *J* = 7.2 Hz, 2H), 1.38–1.21 (m, 16H), 0.90 (t, *J* = 7.0 Hz, 3H). ^13^C NMR (126 MHz, CD_3_OD) δ 157.6, 139.5, 137.9, 129.6, 122.4, 44.5, 42.4, 36.3, 33.1, 32.8, 30.8, 30.7, 30.7, 30.6, 30.5, 30.3, 23.7, 14.4. HRMS: (ESI) [M + H]^+^ calcd for C_22_H_38_N_3_O, 360.3009, observed, 360.3010.

#### *N*-(4-Dodecylphenyl)piperazine-1-carboxamide Hydrogen Chloride (**20f**)

Synthesized according to General Procedure 3. Purified via trituration with ethyl acetate and diethyl ether. White solid (94%, 122 mg). ^1^H NMR (600 MHz, CD_3_OD) δ 7.29–7.21 (m, 2H), 7.10 (d, *J* = 8.4 Hz, 2H), 3.77 (dd, *J* = 6.1, 4.5 Hz, 4H), 3.30–3.25 (m, 4H), 2.60–2.52 (m, 2H), 1.58 (p, *J* = 7.4 Hz, 2H), 1.36–1.22 (m, 18H), 0.90 (t, *J* = 7.0 Hz, 3H). ^13^C NMR (151 MHz, CD_3_OD) δ 157.6, 139.5, 137.9, 129.6, 122.4, 44.5, 42.4, 36.3, 33.1, 32.9, 30.8, 30.8, 30.8, 30.8, 30.6, 30.5, 30.3, 23.8, 14.5. HRMS: (ESI) [M + H]^+^ calcd for C_23_H_40_N_3_O, 374.3166, observed, 374.3173.

#### *N*-(4-Tridecylphenyl)piperazine-1-carboxamide Hydrogen Chloride (**20g**)

Synthesized according to General Procedure 3. Purified via trituration with ethyl acetate and diethyl ether. White solid (91%, 119 mg). ^1^H NMR (600 MHz, CD_3_OD) δ 7.31–7.19 (m, 2H), 7.15–7.06 (m, 2H), 3.82–3.72 (m, 4H), 3.30–3.24 (m, 4H), 2.56 (t, *J* = 7.6 Hz, 2H), 1.59 (p, *J* = 7.3 Hz, 2H), 1.37–1.21 (m, 20H), 0.90 (t, *J* = 7.0 Hz, 3H). ^13^C NMR (151 MHz, CD_3_OD) δ 157.6, 139.5, 137.9, 129.6, 122.4, 44.5, 42.4, 36.3, 33.1, 32.9, 30.8, 30.8, 30.8, 30.8, 30.8, 30.6, 30.5, 30.3, 23.8, 14.5. HRMS: (ESI) [M + H]^+^ calcd for C_24_H_42_N_3_O, 388.3322, observed, 388.3322.

## Supporting Information Available

UPLC trace, ^1^H, and ^13^C NMR spectra for **11i** (**SLF80821178**) (PDF)
Molecular formula strings and associated biological data (CSV)

## Abbreviations

BS1–2: binding site 1–2
dppf: 1,1′-bis(diphenylphosphino)ferrocene
DSS: dextran sulfate sodium
EAE: experimental autoimmune encephalomyelitis
HCTU: 2-(6-chloro-1Hbenzotriazole-1-yl)-1,1,3,3-tetramethylaminium hexafluorophosphate
IP: intraperitoneal
LPP1–3: lipid phosphate phosphatase 1–3
MD: molecular dynamics
MFS: major facilitator superfamily
MM/GBSA: molecular mechanics with generalized born and surface area
MS: multiple sclerosis
PO: *per oral*
S1P: sphingosine-1-phosphate
S1P1–5: sphingosine-1-phosphate receptors 1–5
SAR: structure–activity relationship
SphK1: sphingosine kinase 1
SphK2: sphingosine kinase 2
Spns2: spinster homologue 2
SPP1–2: sphingosine-1-phosphate phosphohydrolases 1–2
SRM: sphingosine-1-phosphate receptor modulator
UC: ulcerative colitis
