# Supplementary material for: Discovery of Potent, Orally Bioavailable Sphingosine-1-Phosphate Transporter (Spns2) Inhibitors
Source: J Med Chem. 2024 Jul 2;67(13):11273–95. doi: 10.1021/acs.jmedchem.4c00879 (PMC11247503; doi:10.1021/acs.jmedchem.4c00879)

Supporting Information

Discovery of Potent, Orally Bioavailable Sphingosine-1-Phosphate Transporter (Spns2) Inhibitors

Daniel J. Foster,[1] Kyle Dunnavant,[1] Christopher W. Shrader,[1] Marion LoPresti,[1] Sarah Seay,[1] Yugesh Kharel,[2] Anne M. Brown,[1] Tao Huang,[2] Kevin R. Lynch,[2] Webster L. Santos[1,*]

[1]Department of Chemistry and Virginia Tech Center for Drug Discovery, Virginia Tech, Blacksburg, VA 24061
[2]Department of Pharmacology, University of Virginia, Charlottesville, VA, 22908

Corresponding Author Email: santosw@vt.edu

Table of Contents:

UPLC trace for compound **11i**……………………………………………………………...S2

^{1}H and ^{13}C spectra………………………………………………………………………..S3

UPLC trace for compound **11i**

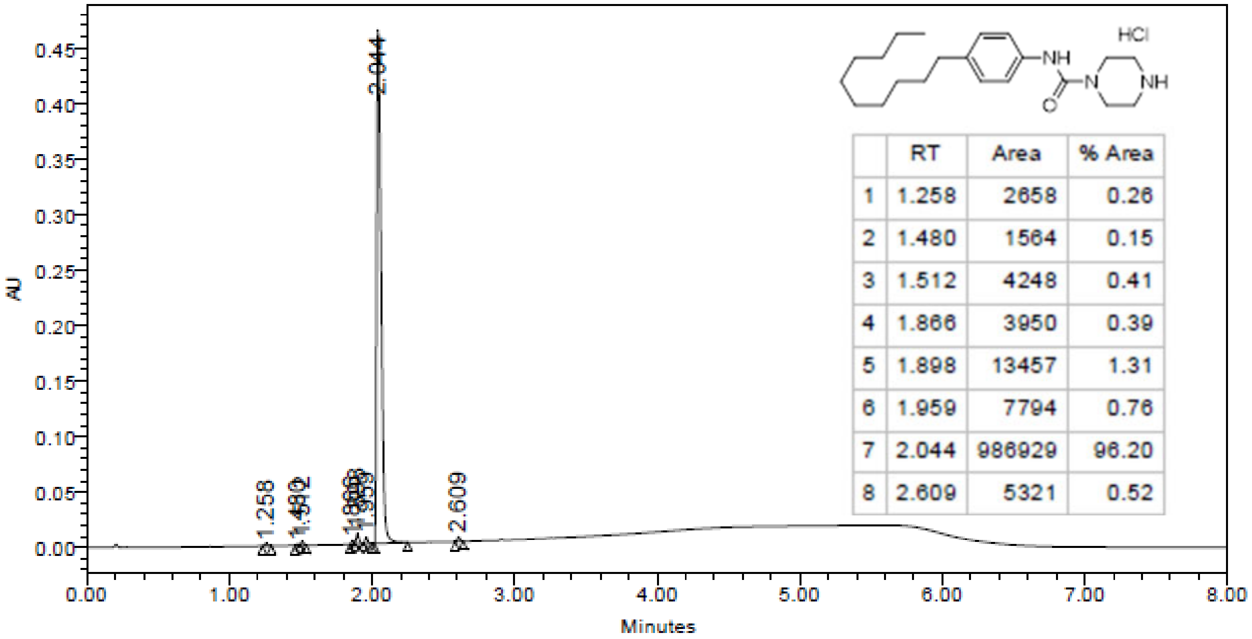

	RT	Area	% Area
1	1.258	2658	0.26
2	1.480	1564	0.15
3	1.512	4248	0.41
4	1.866	3950	0.39
5	1.898	13457	1.31
6	1.959	7794	0.76
7	2.044	986929	96.20
8	2.609	5321	0.52

^{1}H NMR (400 MHz, CD_3OD) **4a**

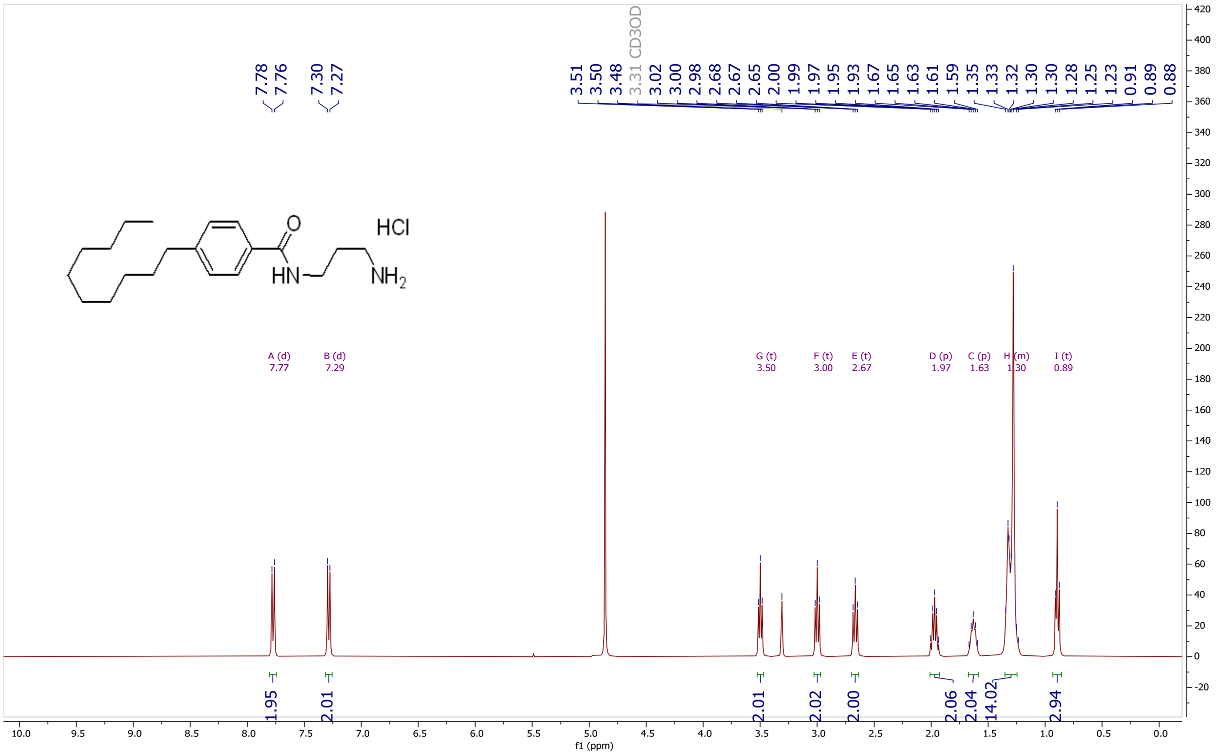

^{13}C NMR (101 MHz, CD_3OD) **4a**

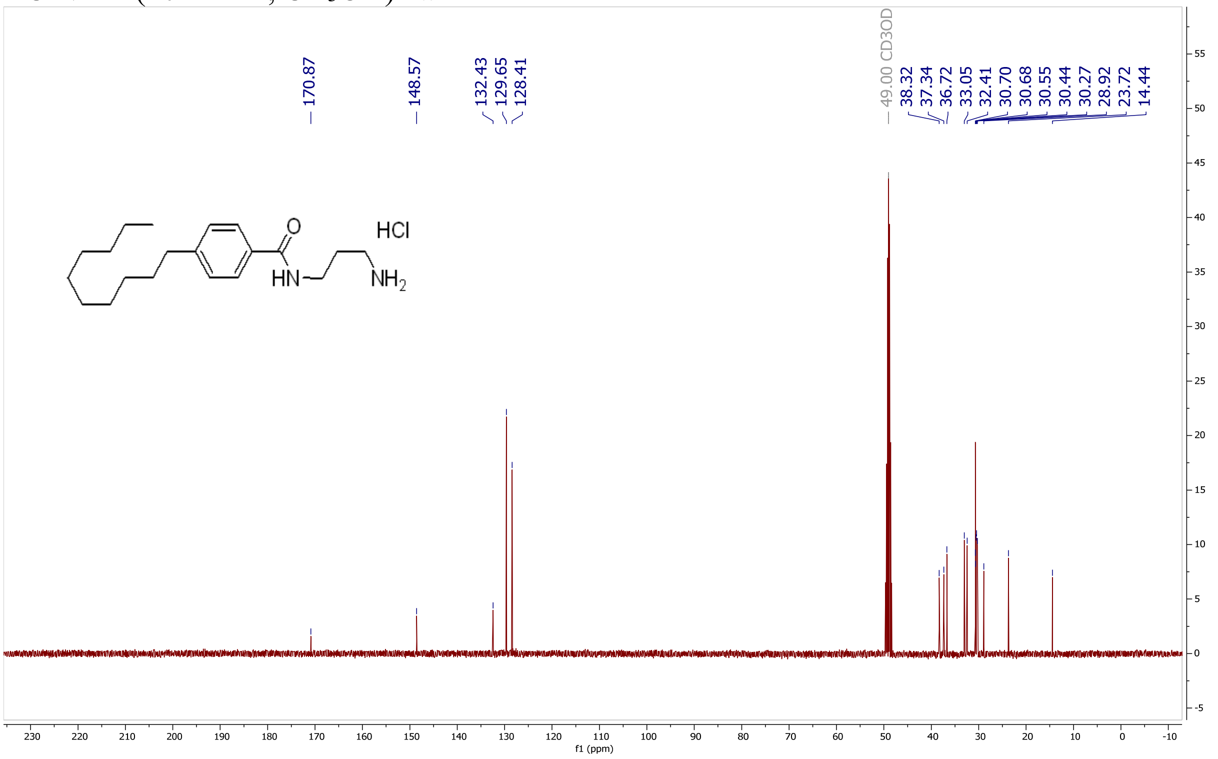

^{1}H NMR (400 MHz, CD_3OD) **4b**

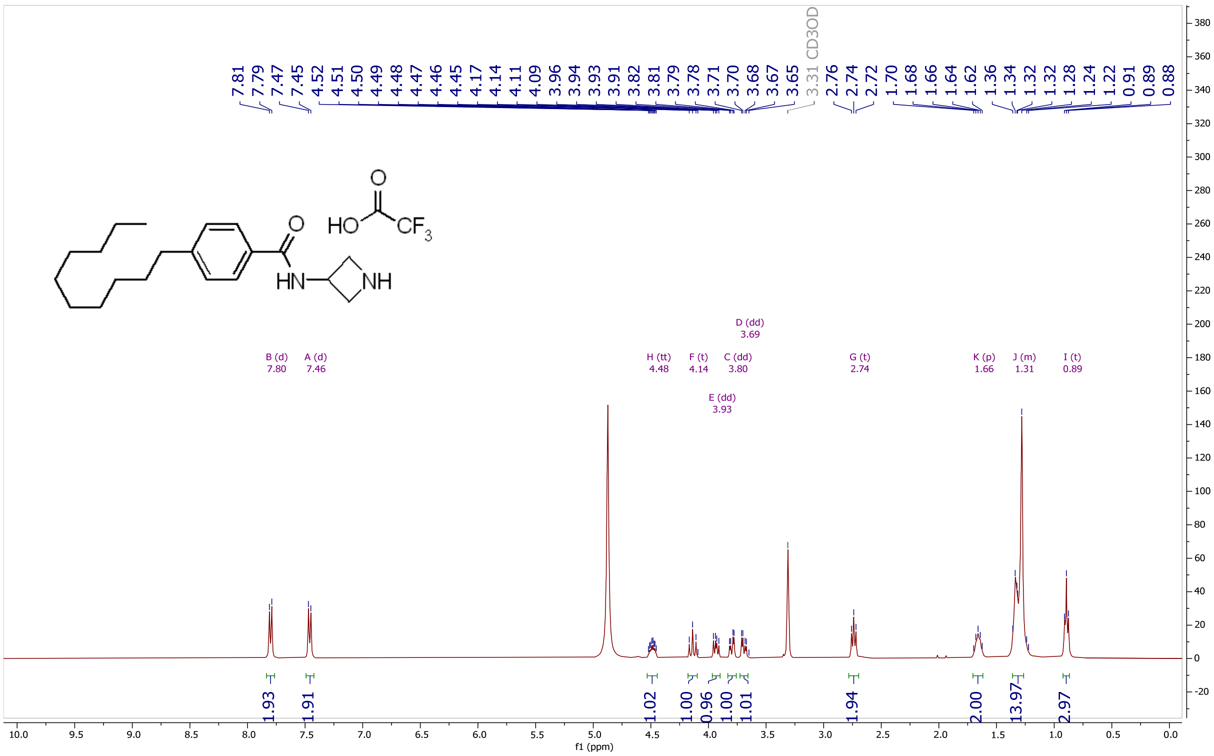

^{13}C NMR (101 MHz, CD_3OD) **4b**

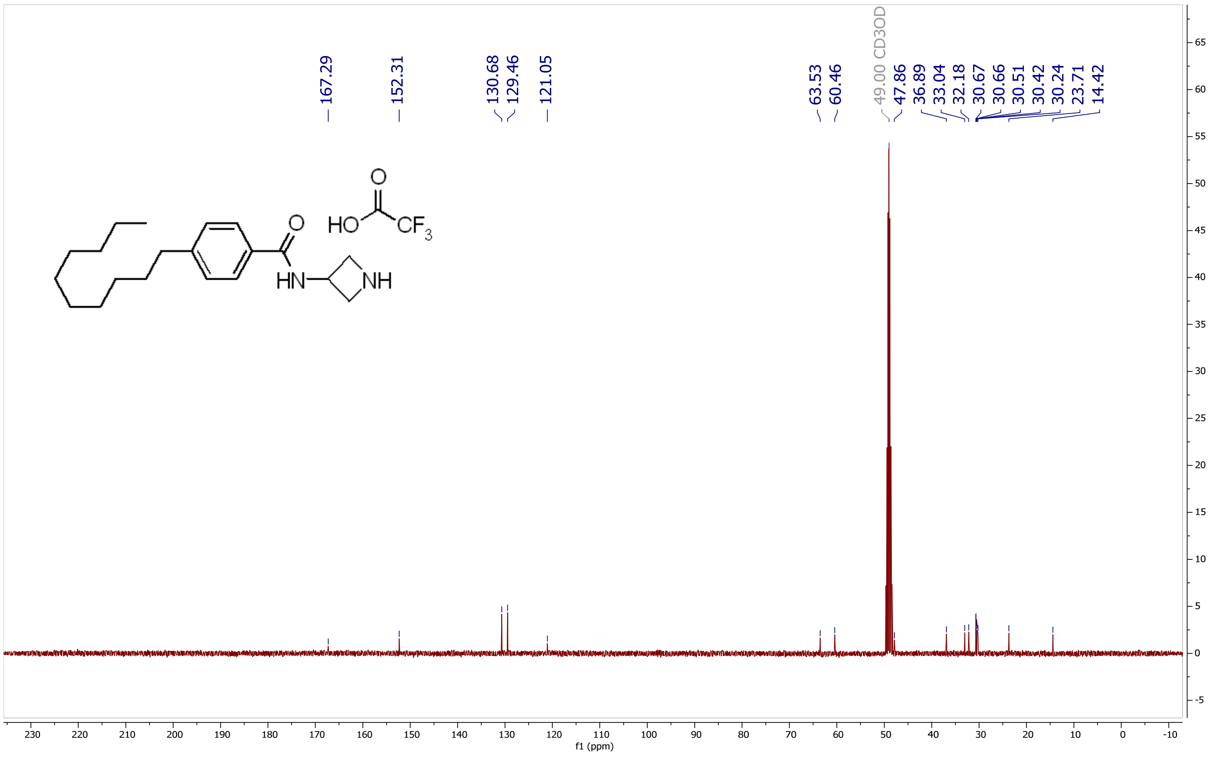

^{1}H NMR (400 MHz, CD_3OD) **4c**

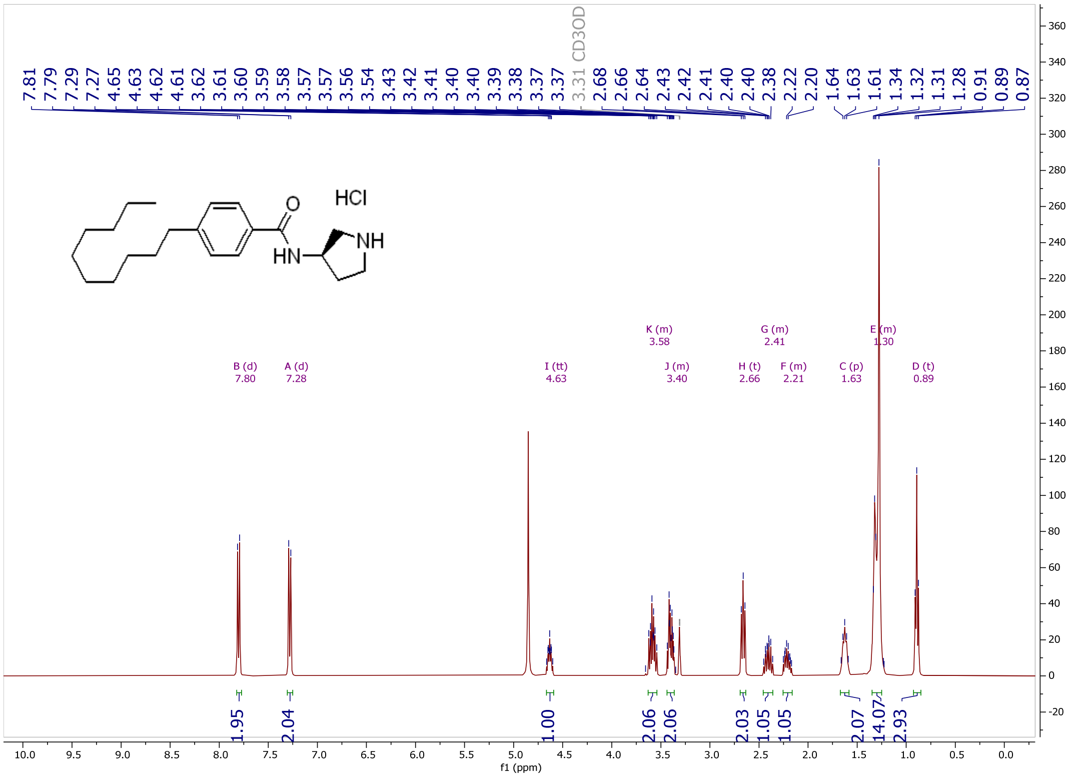

^{13}C NMR (101 MHz, CD_3OD) **4c**

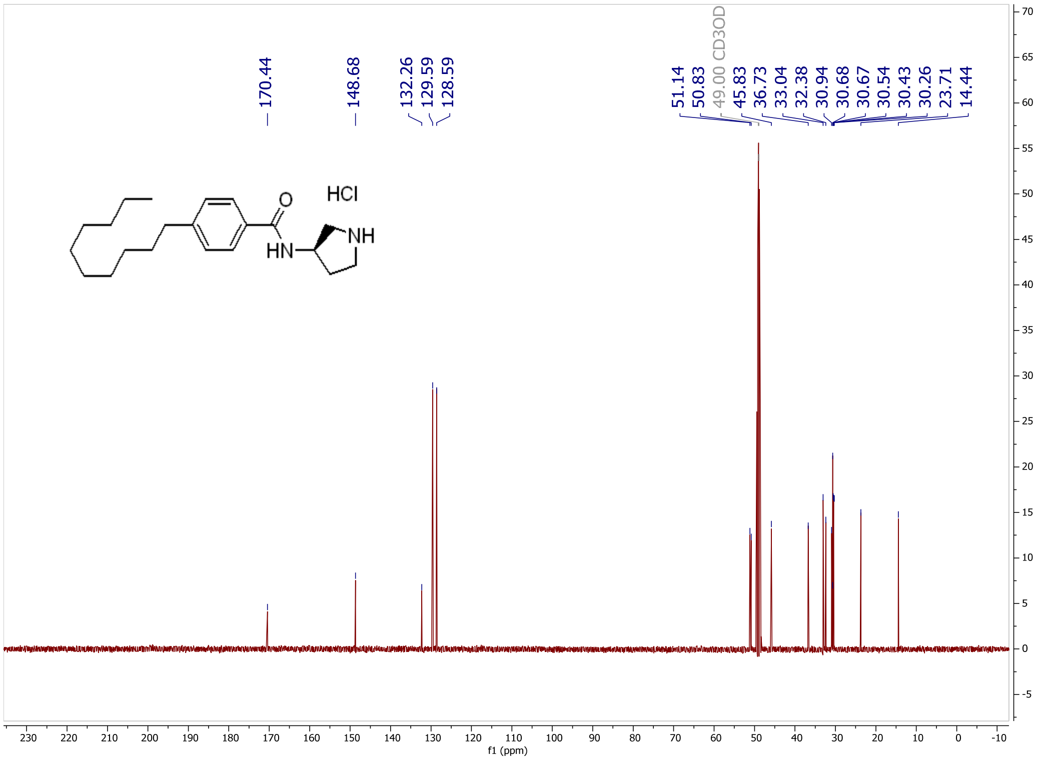

^{1}H NMR (400 MHz, CD_3OD) **4d**

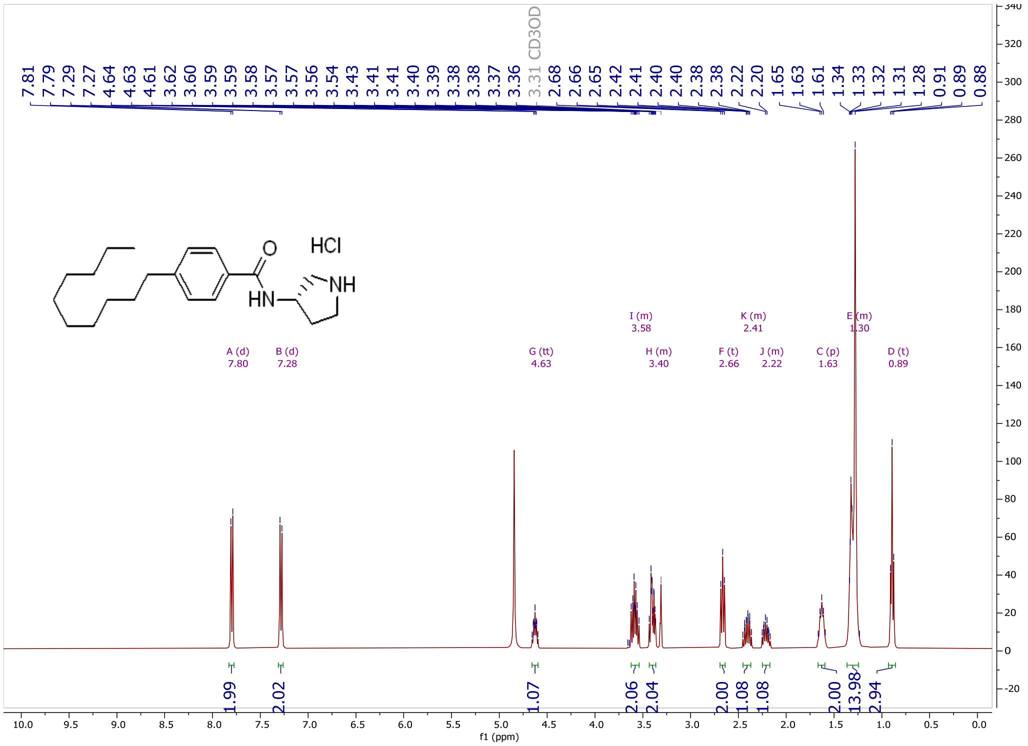

^{13}C NMR (101 MHz, CD_3OD) **4d**

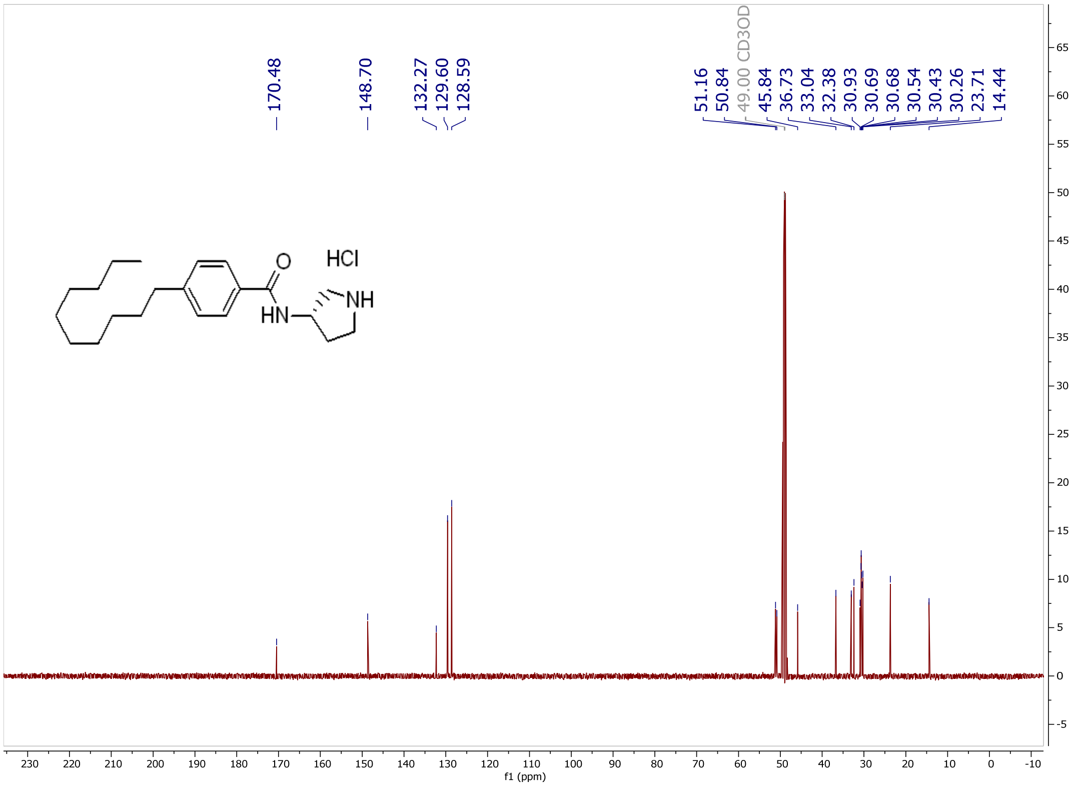

^{1}H NMR (400 MHz, CD_3OD) **4e**

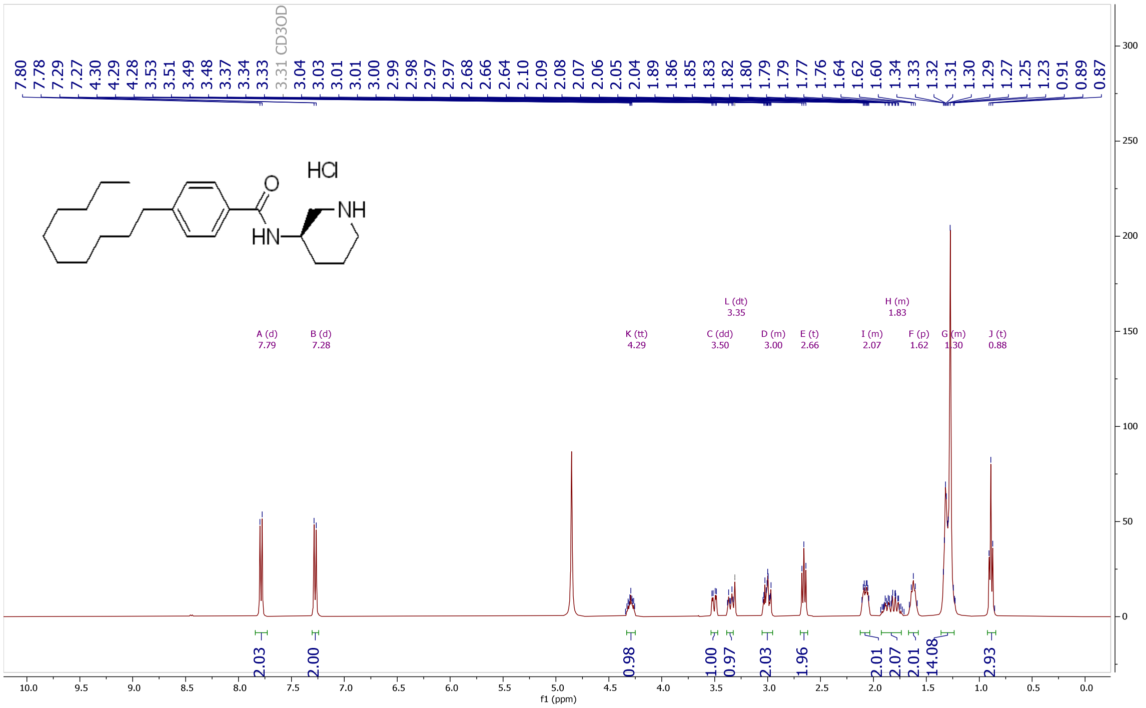

^{13}C NMR (101 MHz, CD_3OD) **4e**

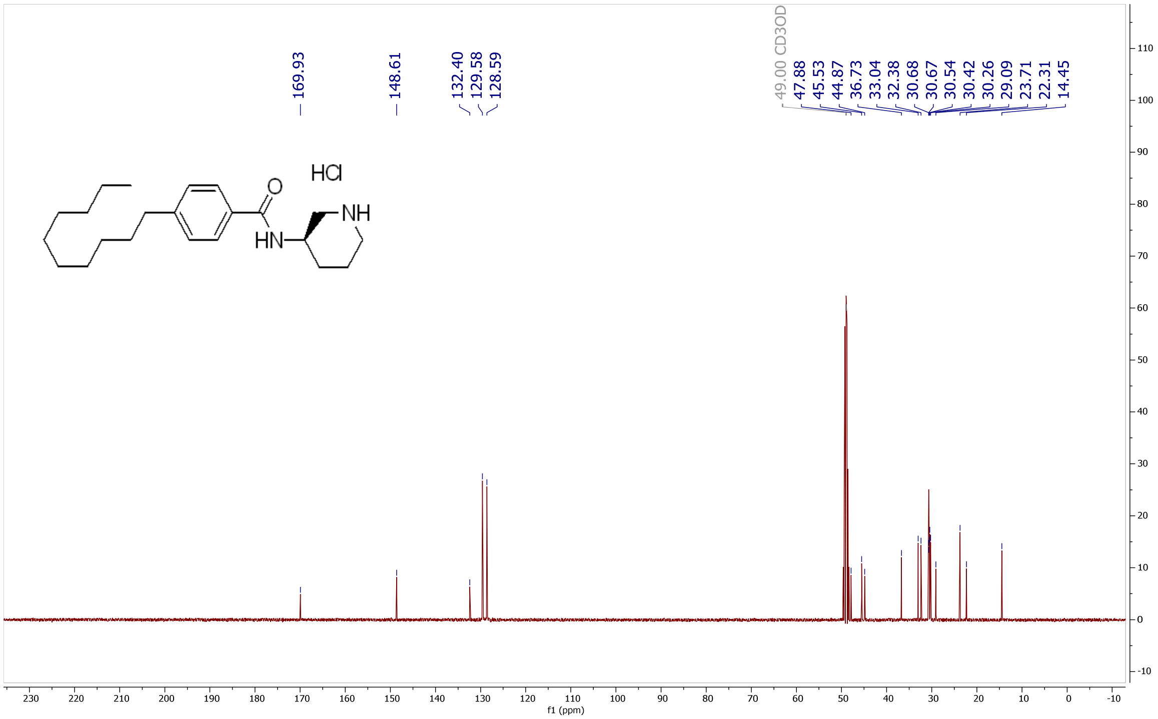

^{1}H NMR (400 MHz, CD_3OD) **4f**

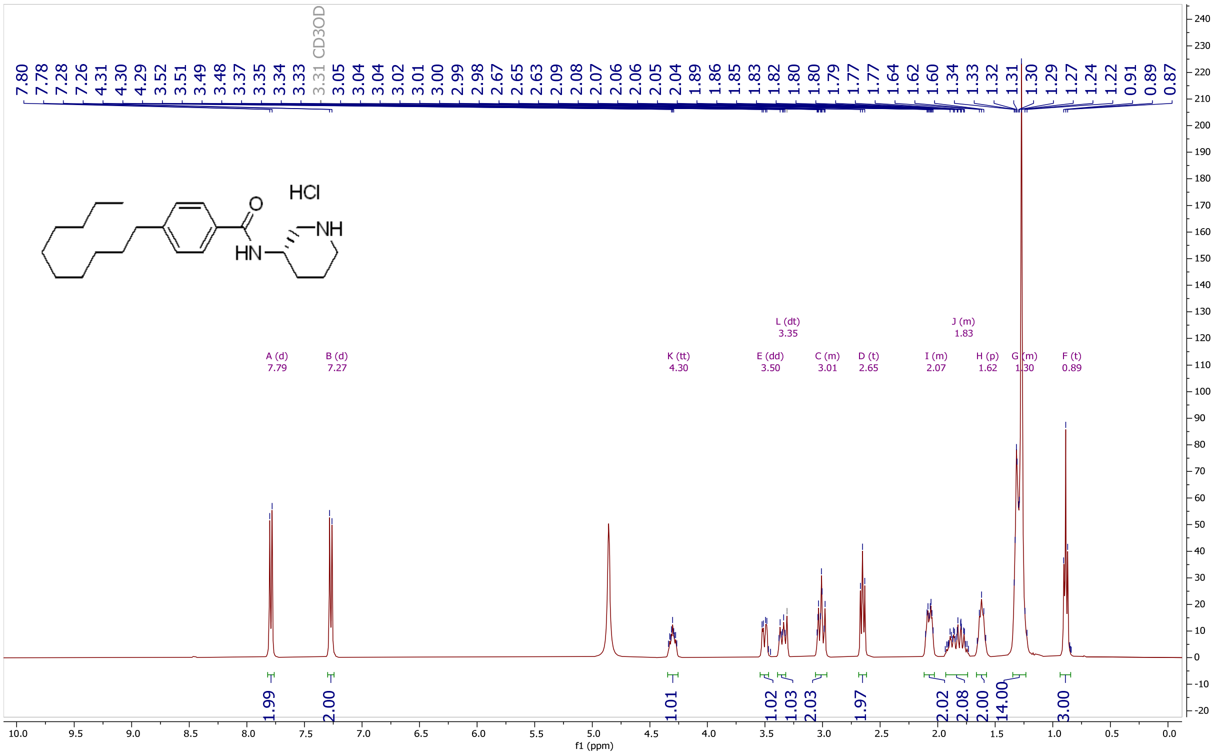

^{13}C NMR (101 MHz, CD_3OD) **4f**

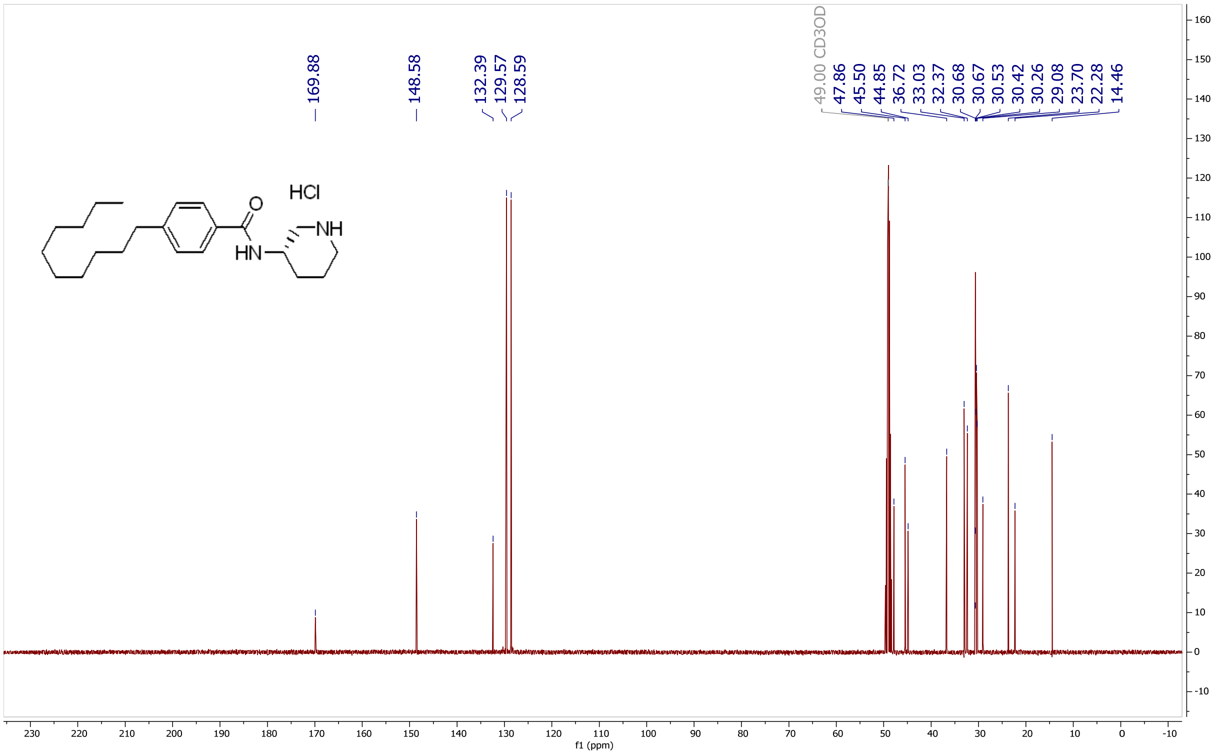

^{1}H NMR (400 MHz, CD_3OD) **4g**

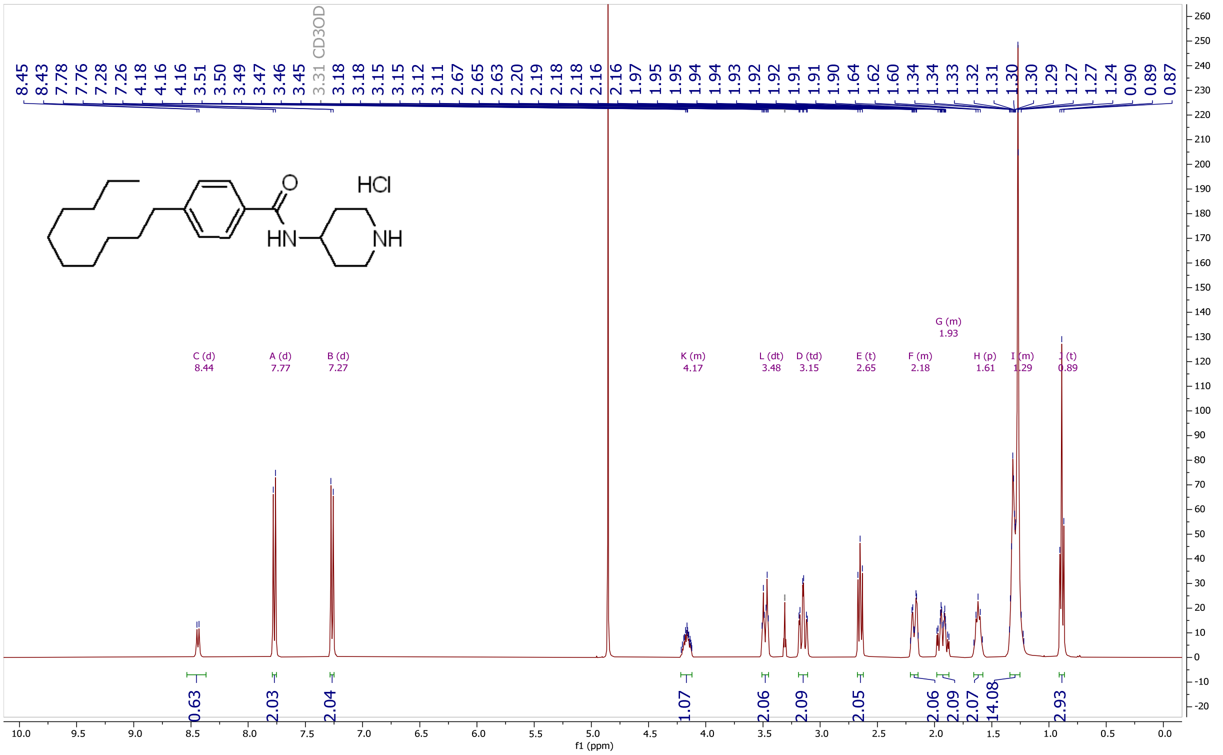

^{13}C NMR (101 MHz, CD_3OD) **4g**

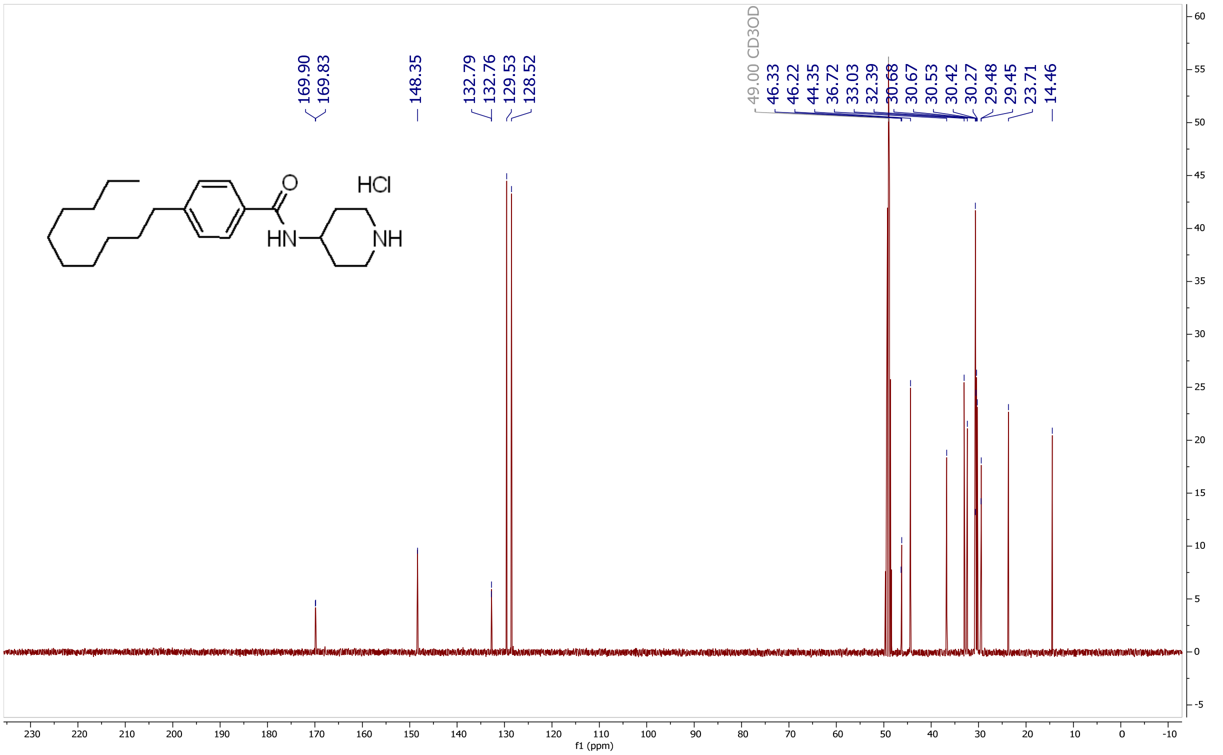

^{1}H NMR (400 MHz, CD_3OD) **4h**

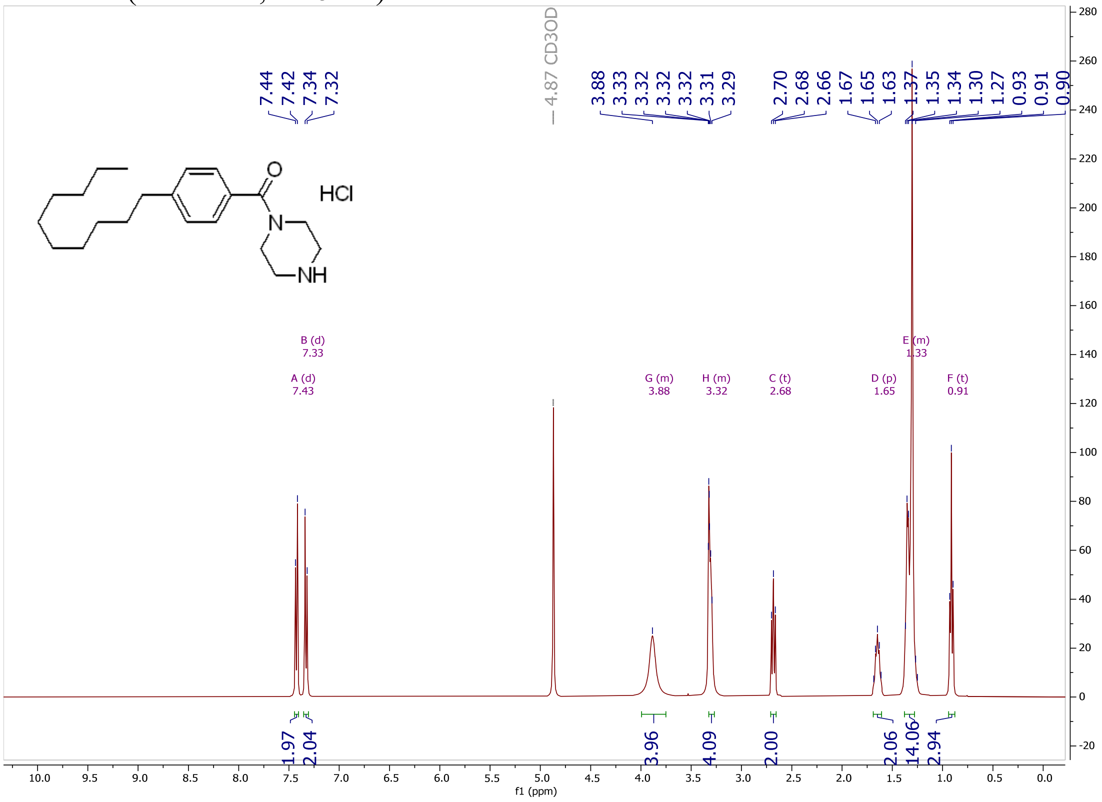

^{13}C NMR (101 MHz, CD_3OD) **4h**

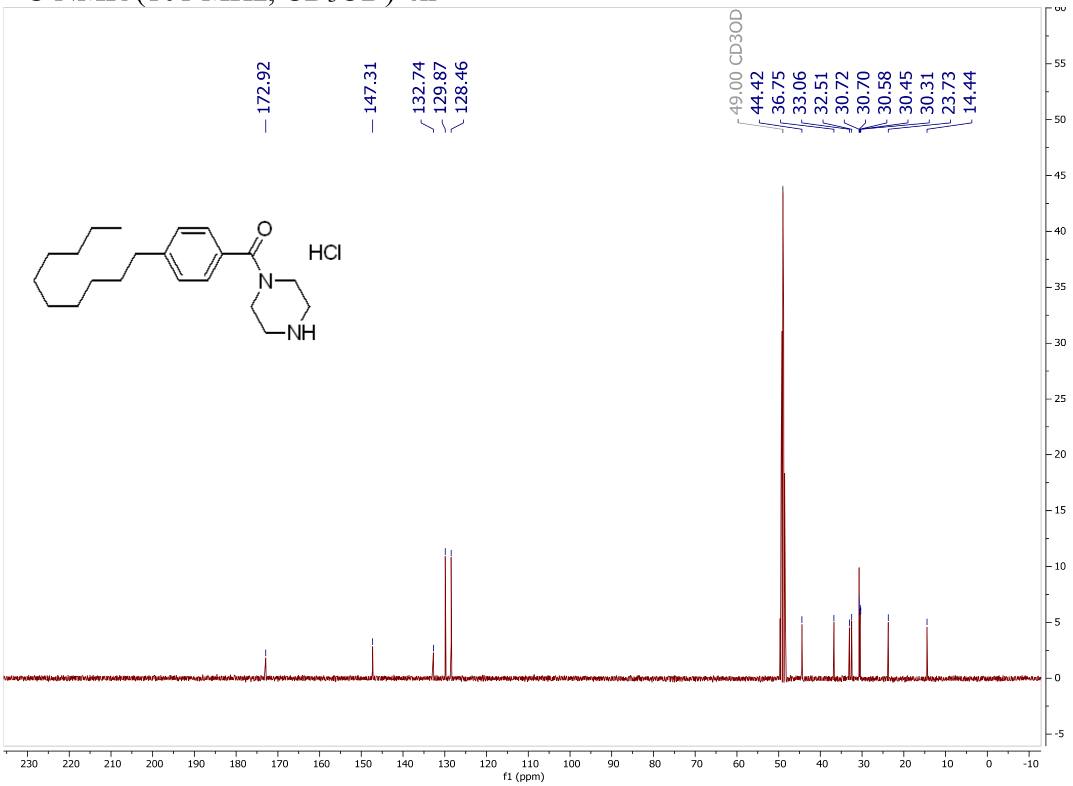

^{1}H NMR (500 MHz, CD_3OD) **4i**

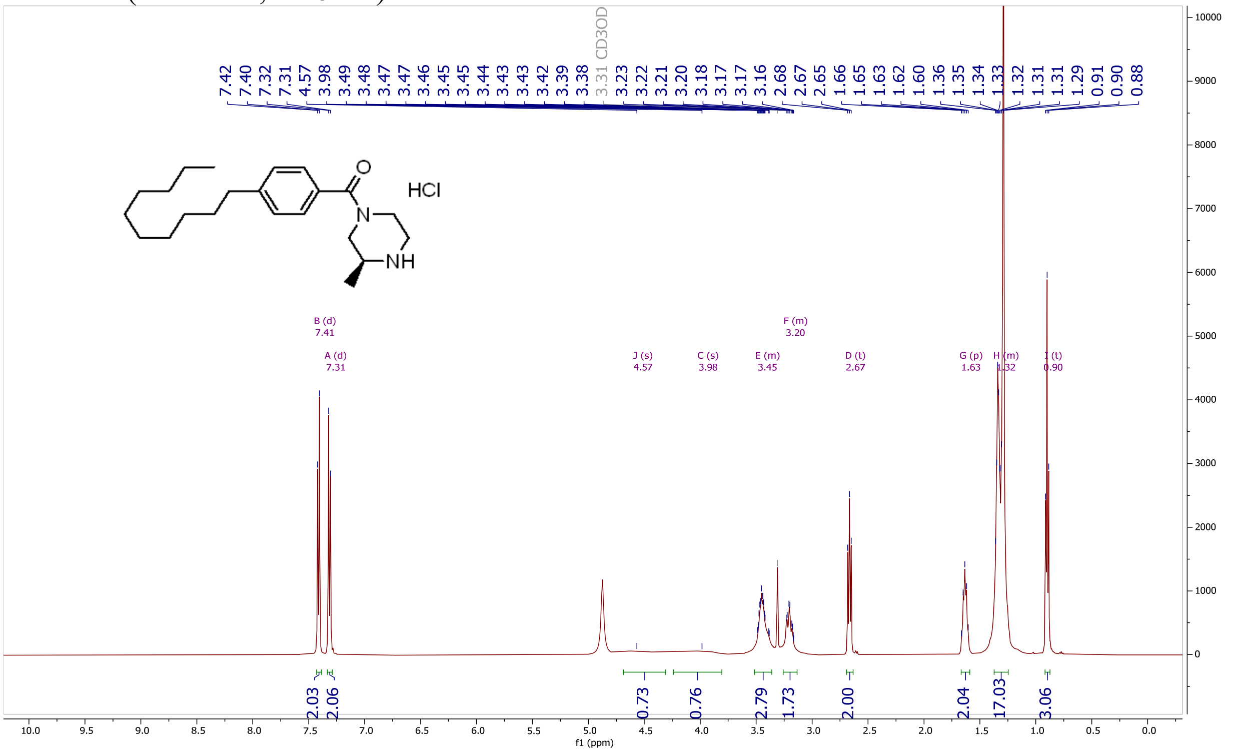

^{13}C NMR (126 MHz, CD_3OD) **4i**

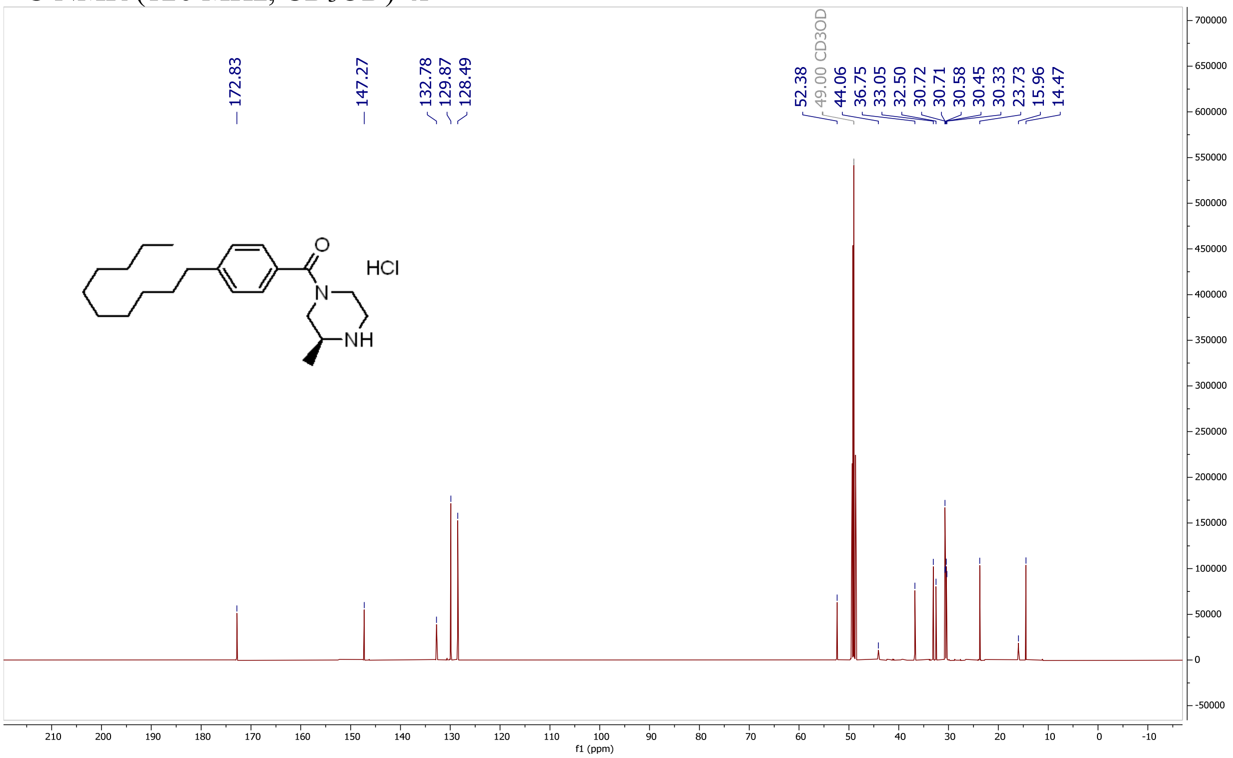

^{1}H NMR (500 MHz, CD_3OD) **4j**

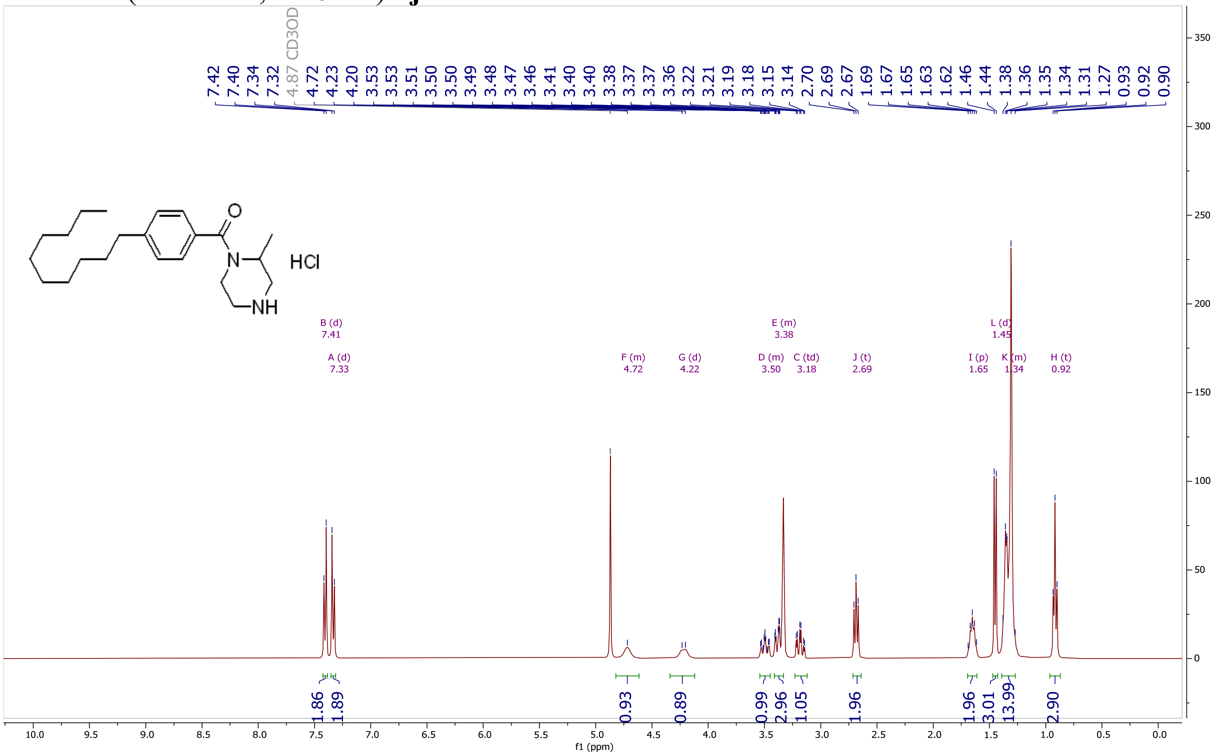

^{13}C NMR (126 MHz, CD_3OD) **4j**

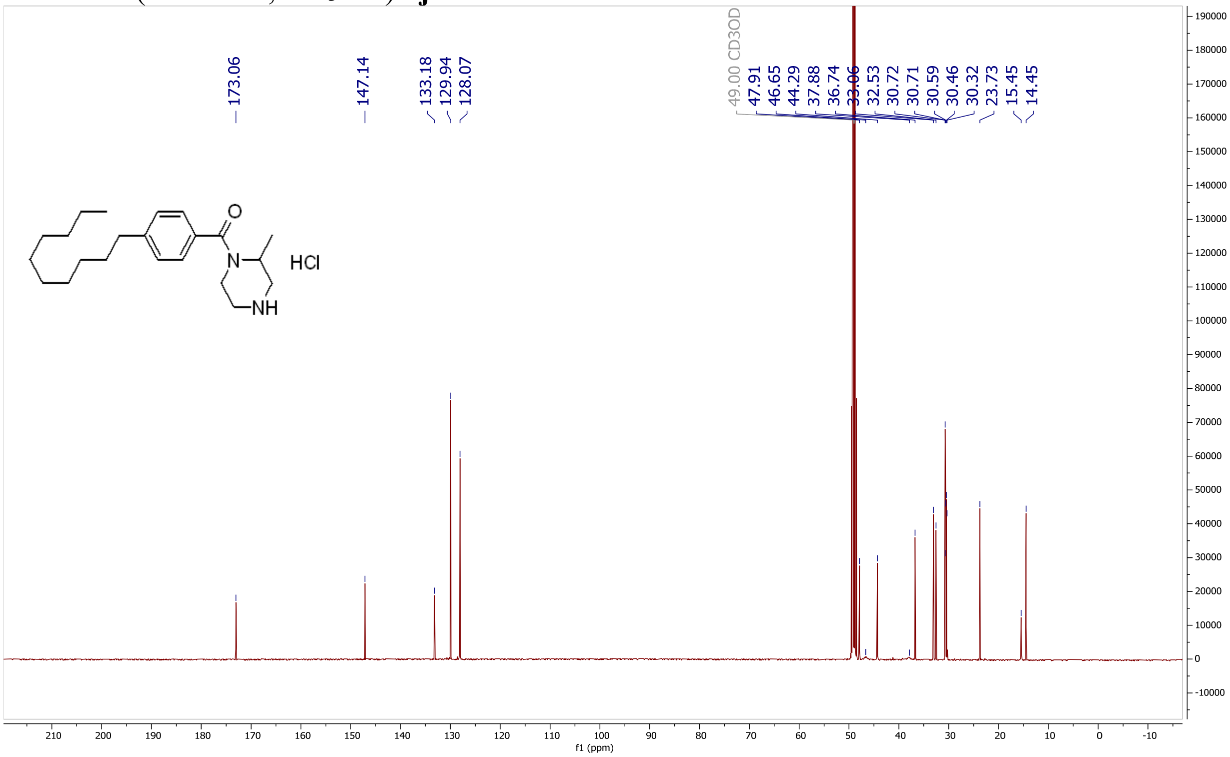

^{1}H NMR (400 MHz, CD_3OD) **4k**

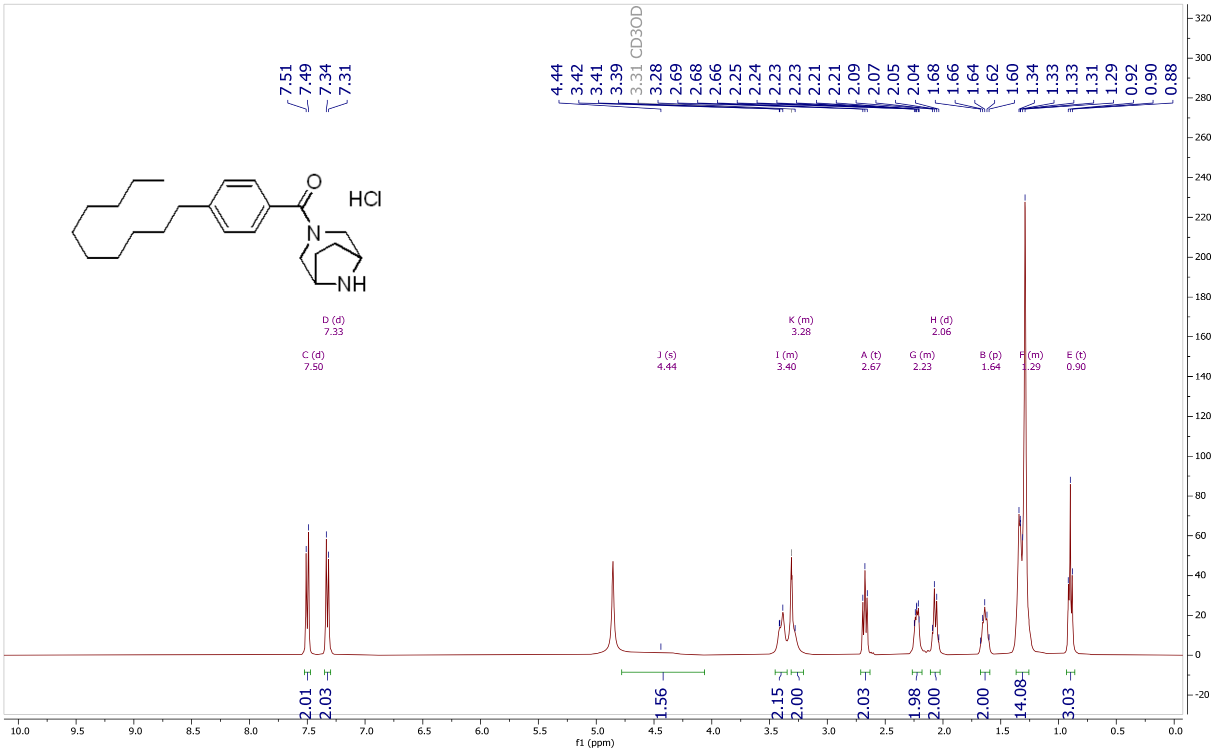

^{13}C NMR (126 MHz, CD_3OD) **4k**

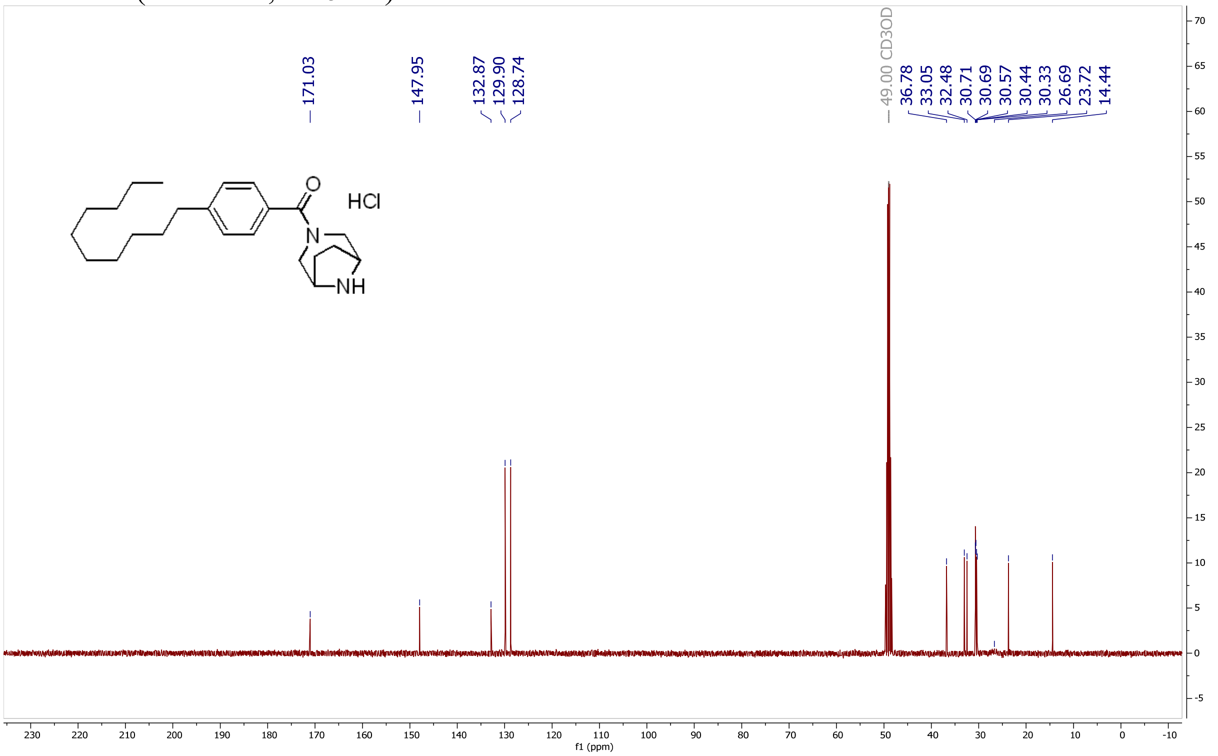

^{1}H NMR (400 MHz, CD_3OD) **4l**

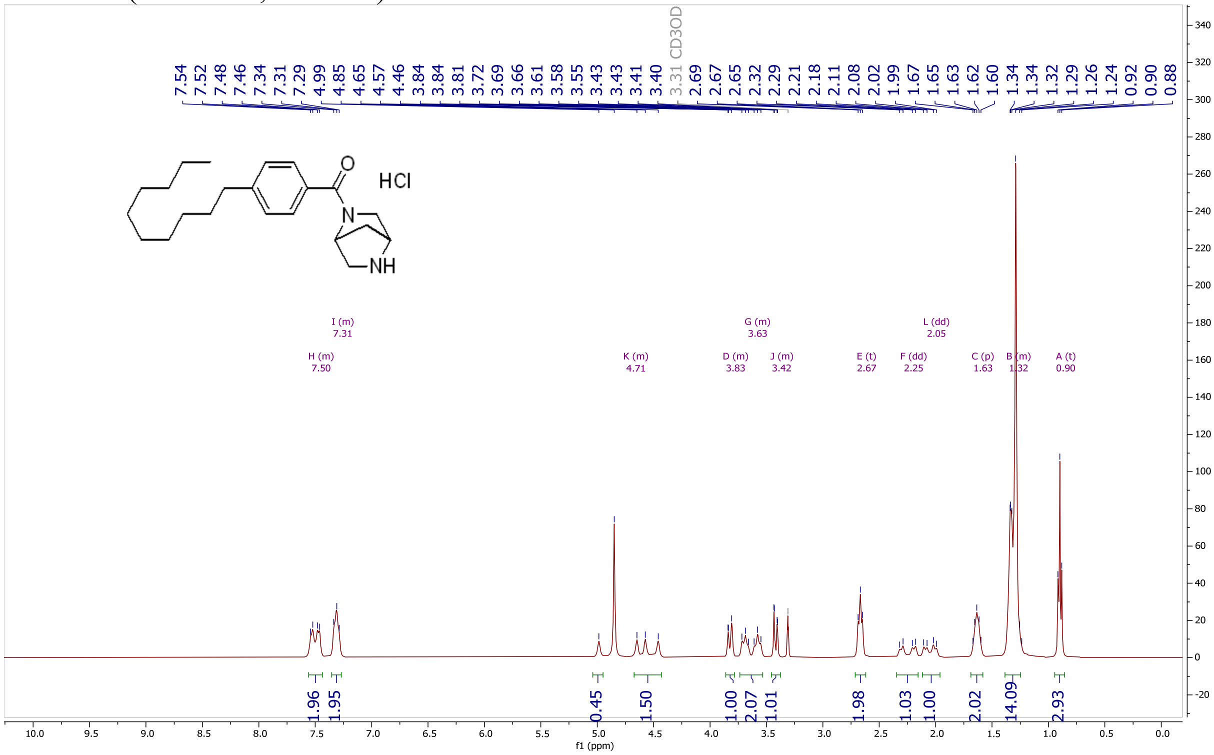

^{13}C NMR (101 MHz, CD_3OD) **4l**

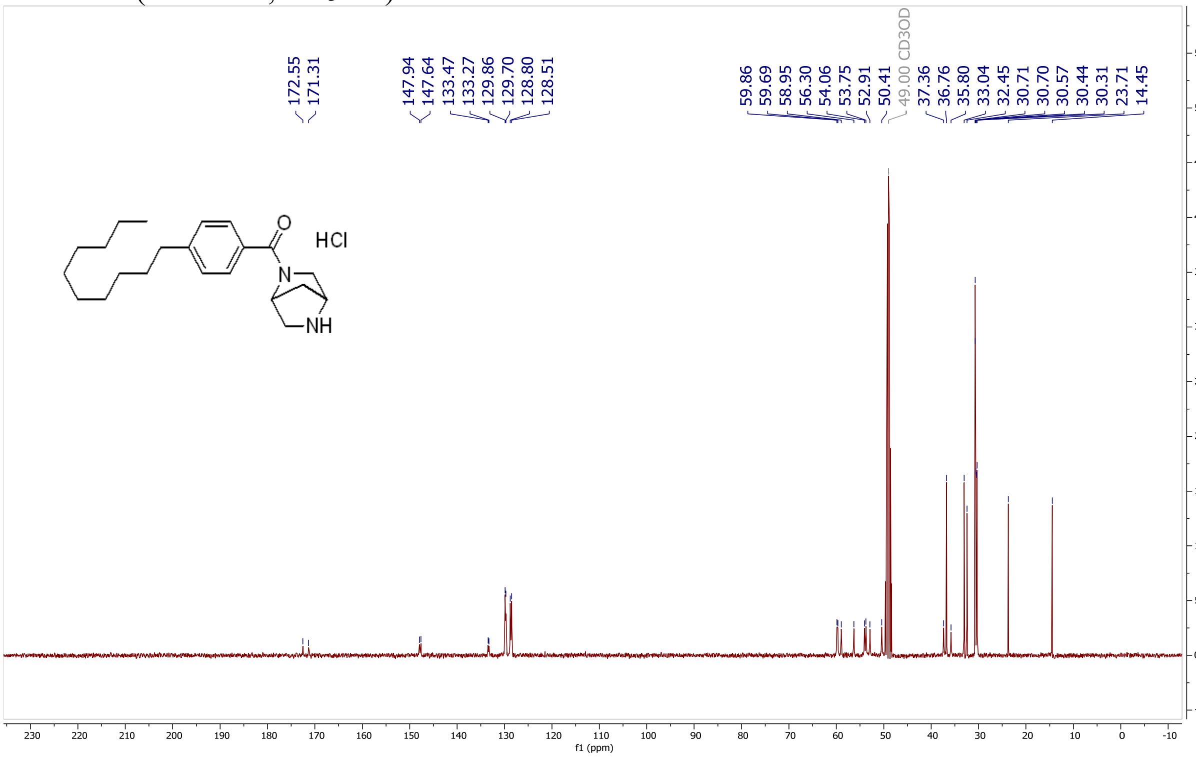

^{1}H NMR (400 MHz, CD_3OD) **8a**

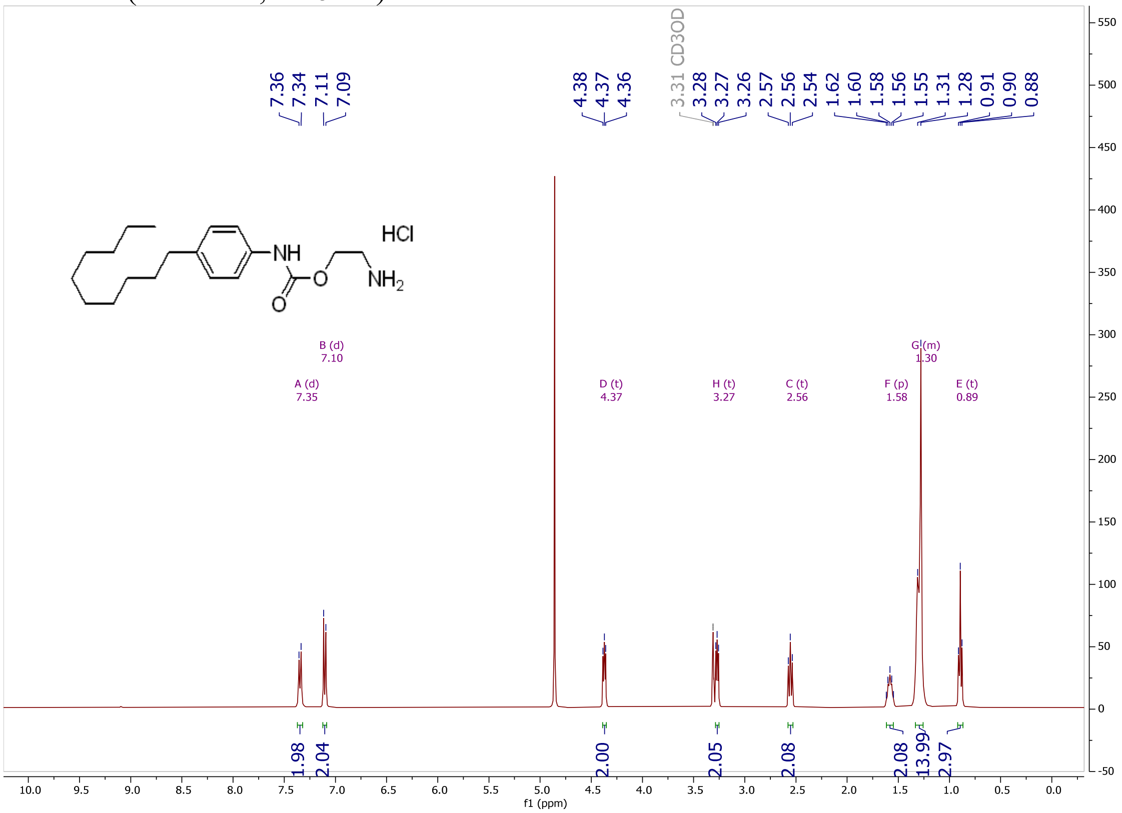

^{13}C NMR (101 MHz, CD_3OD) **8a**

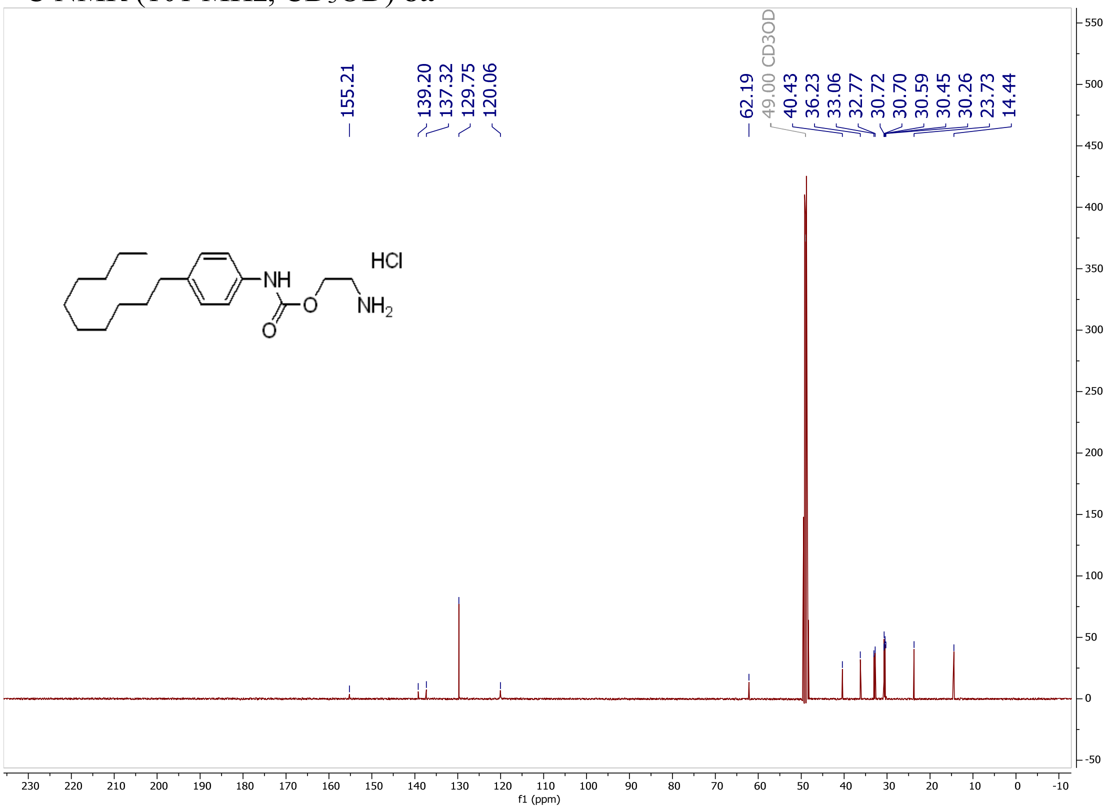

^{1}H NMR (400 MHz, CD_3OD) **8b**

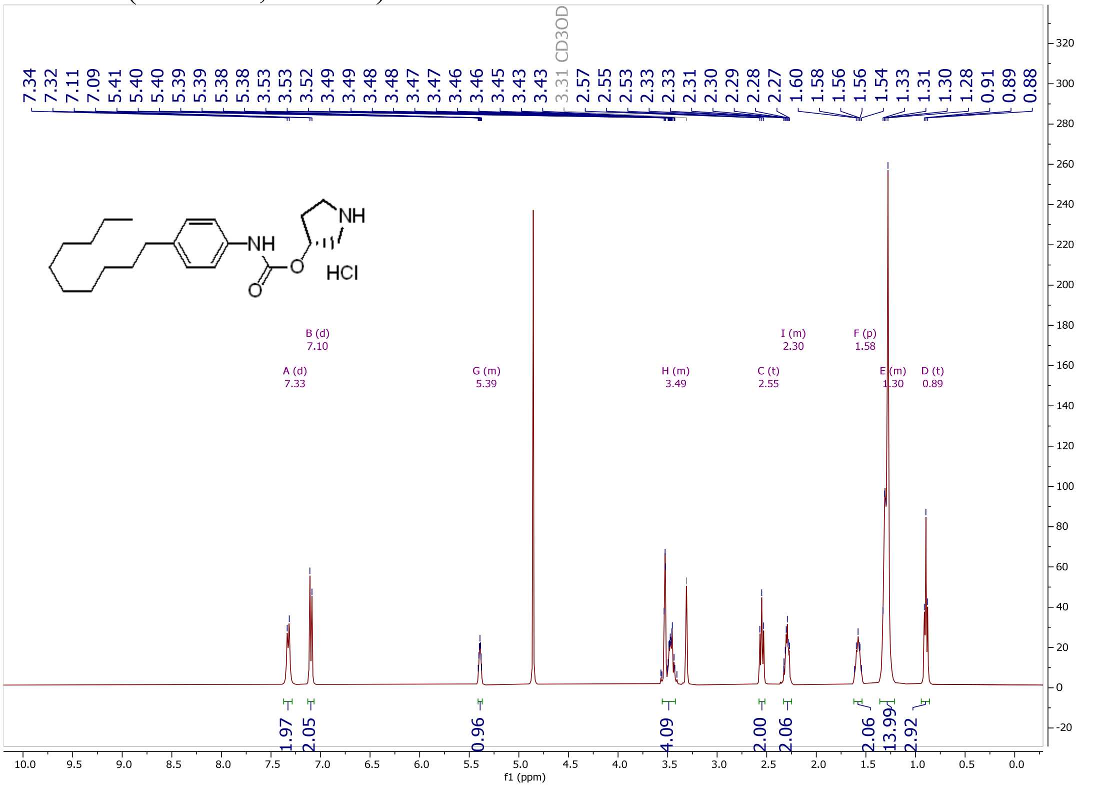

^{13}C NMR (101 MHz, CD_3OD) **8b**

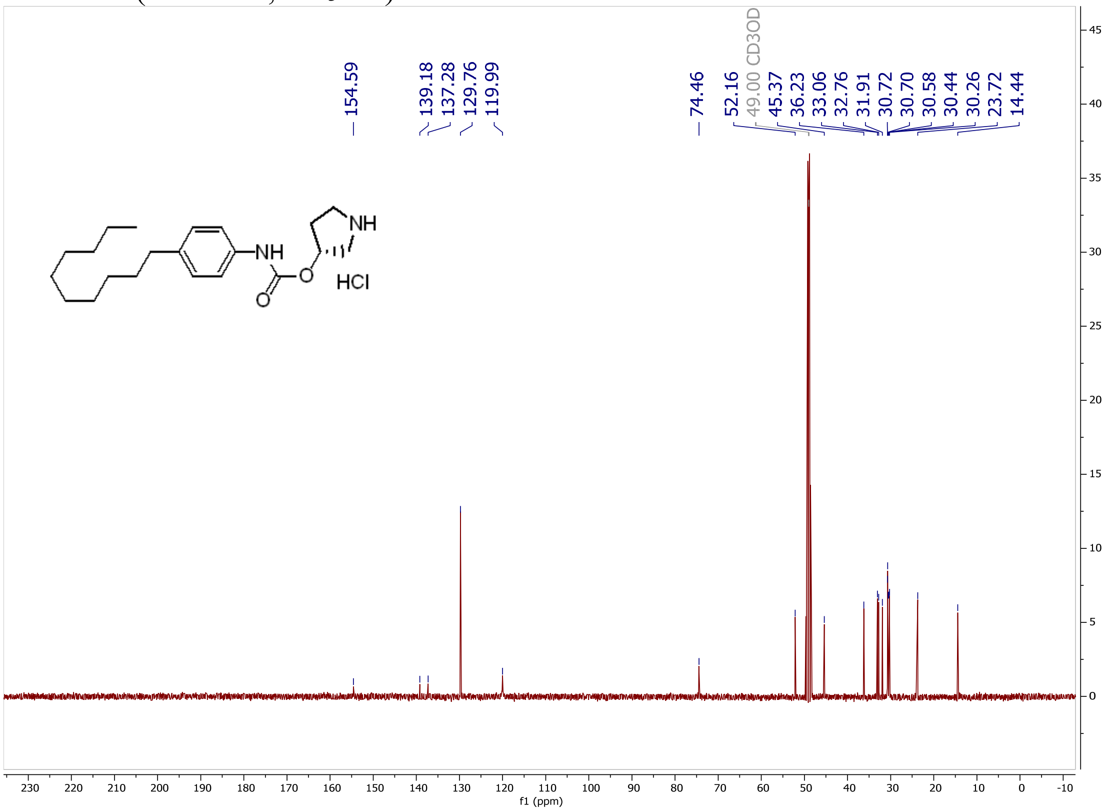

^{1}H NMR (400 MHz, CD_3OD) **8c**

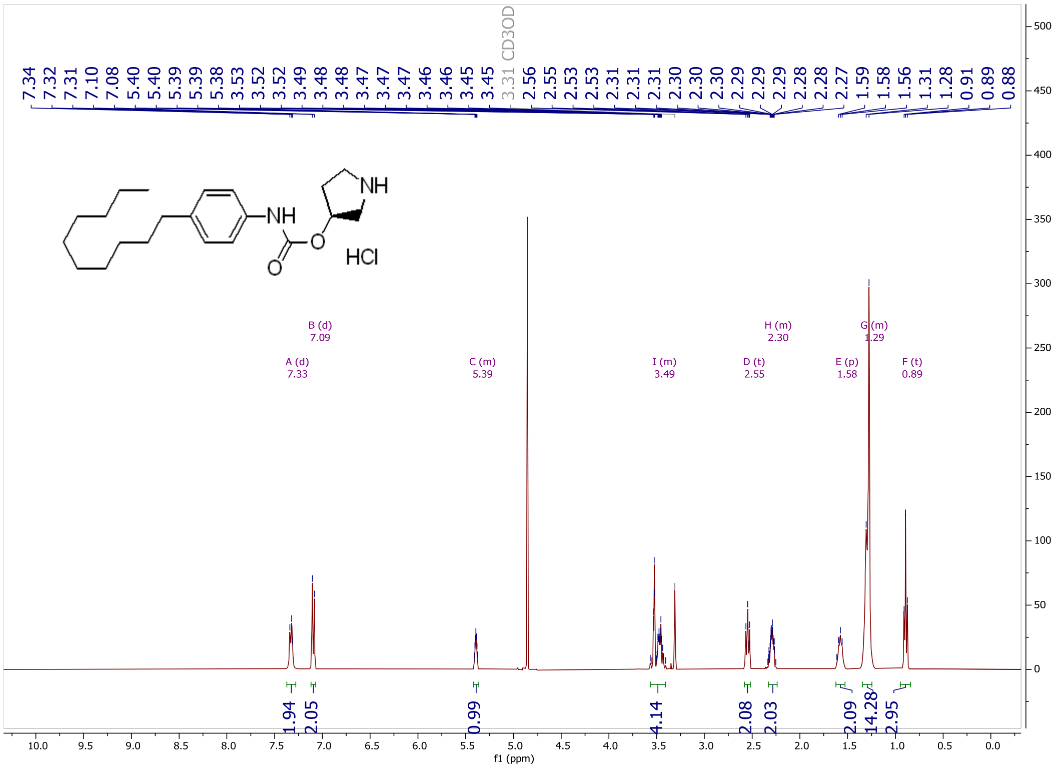

^{13}C NMR (101 MHz, CD_3OD) **8c**

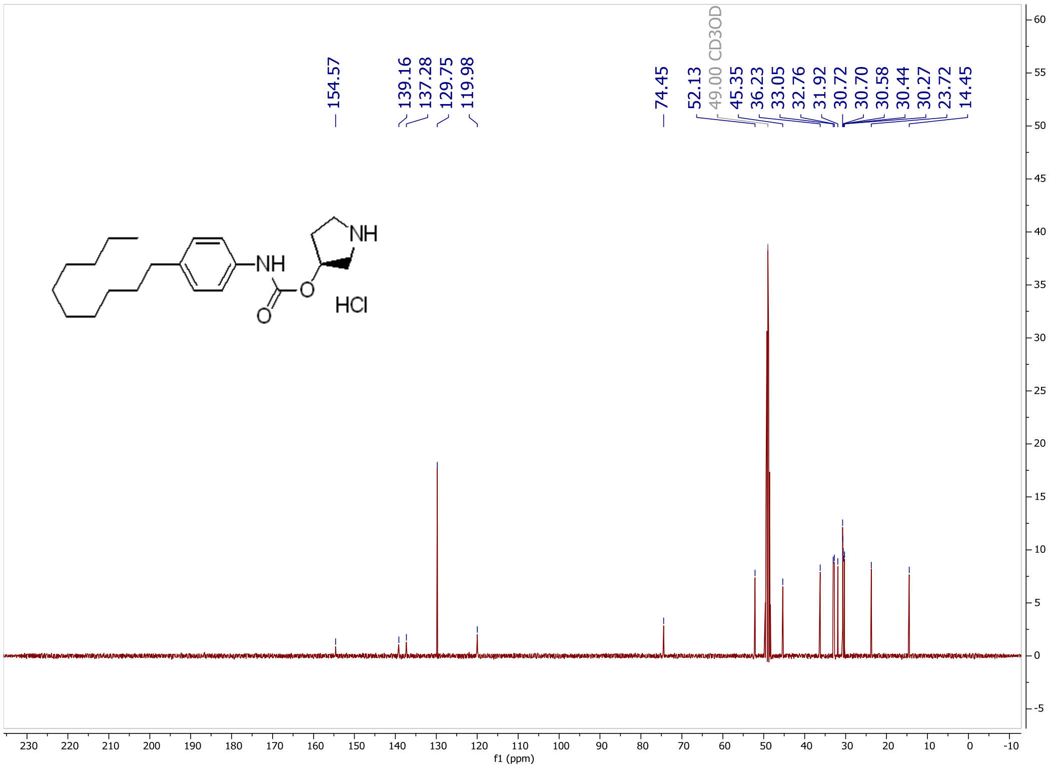

^{1}H NMR (400 MHz, CD_3OD) **8d**

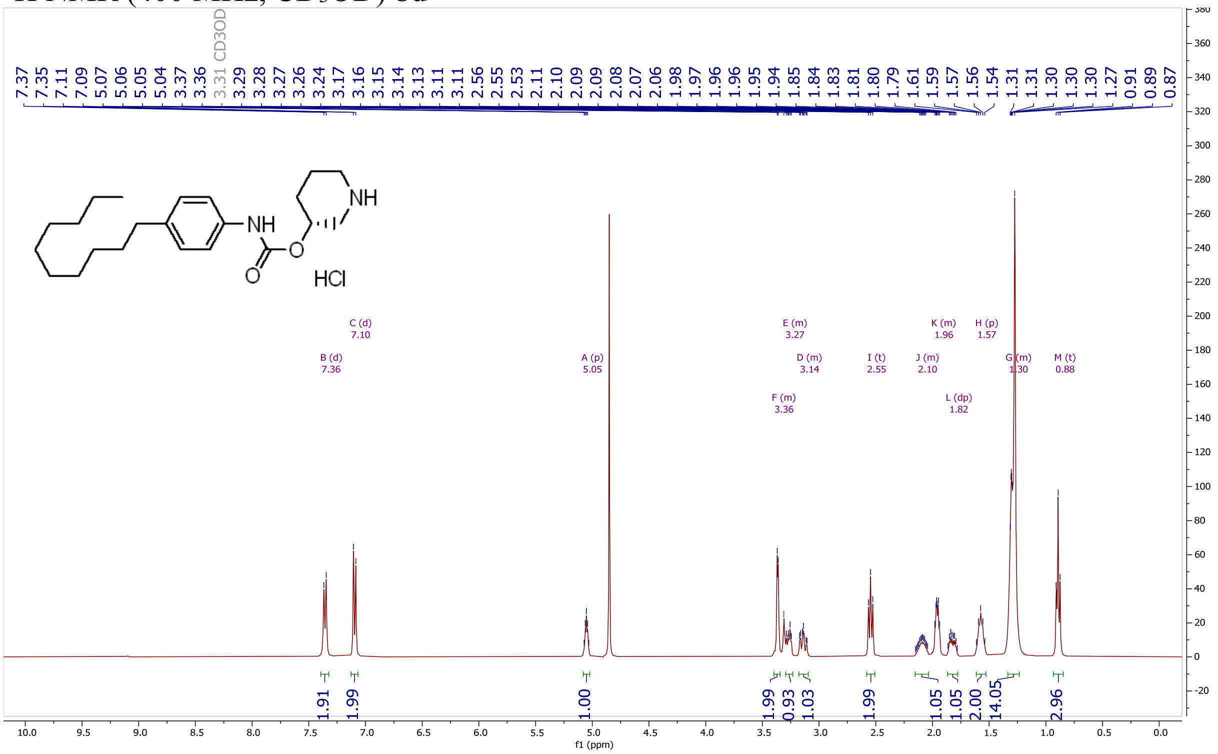

^{13}C NMR (101 MHz, CD_3OD) **8d**

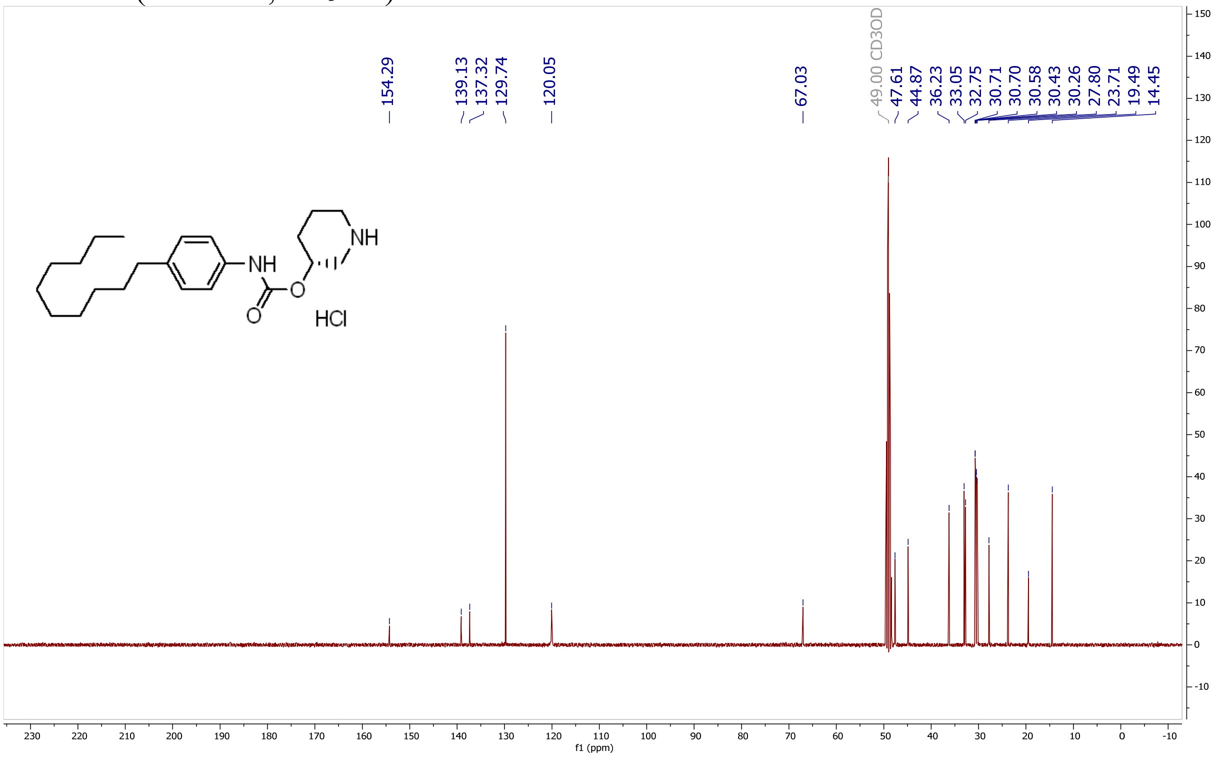

^{1}H NMR (400 MHz, CD_3OD) **8e**

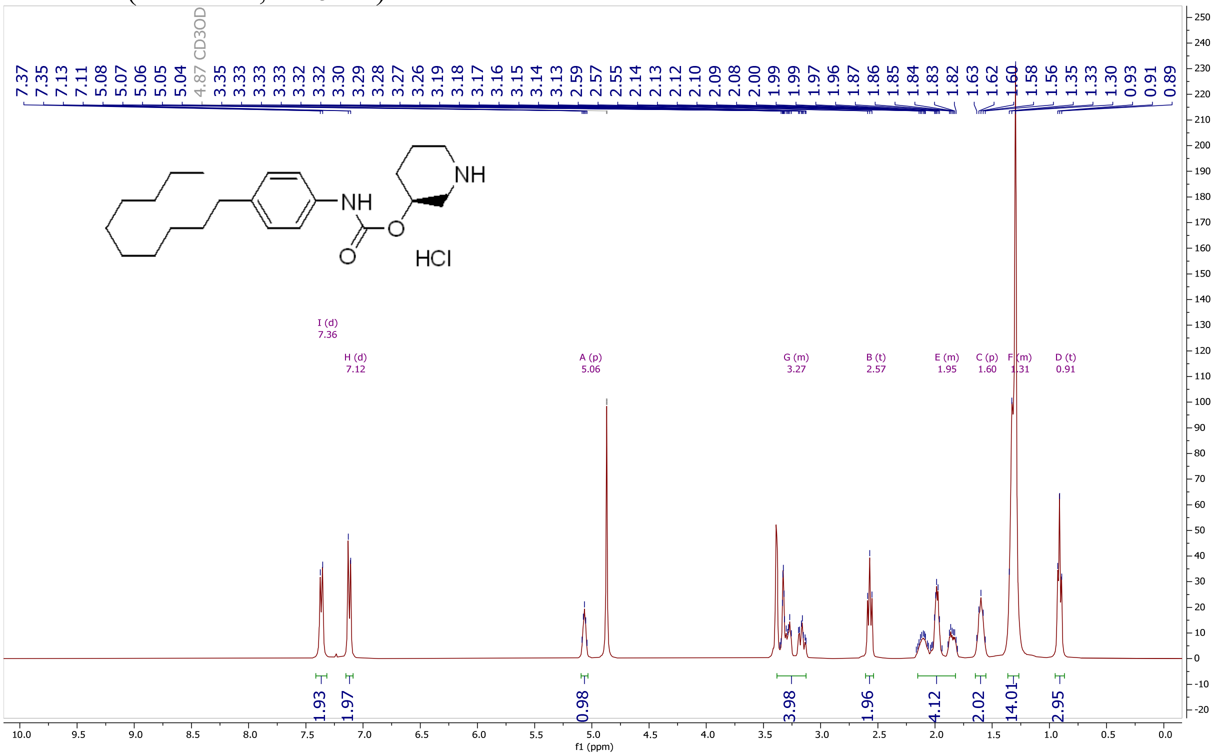

^{13}C NMR (101 MHz, CD_3OD) **8e**

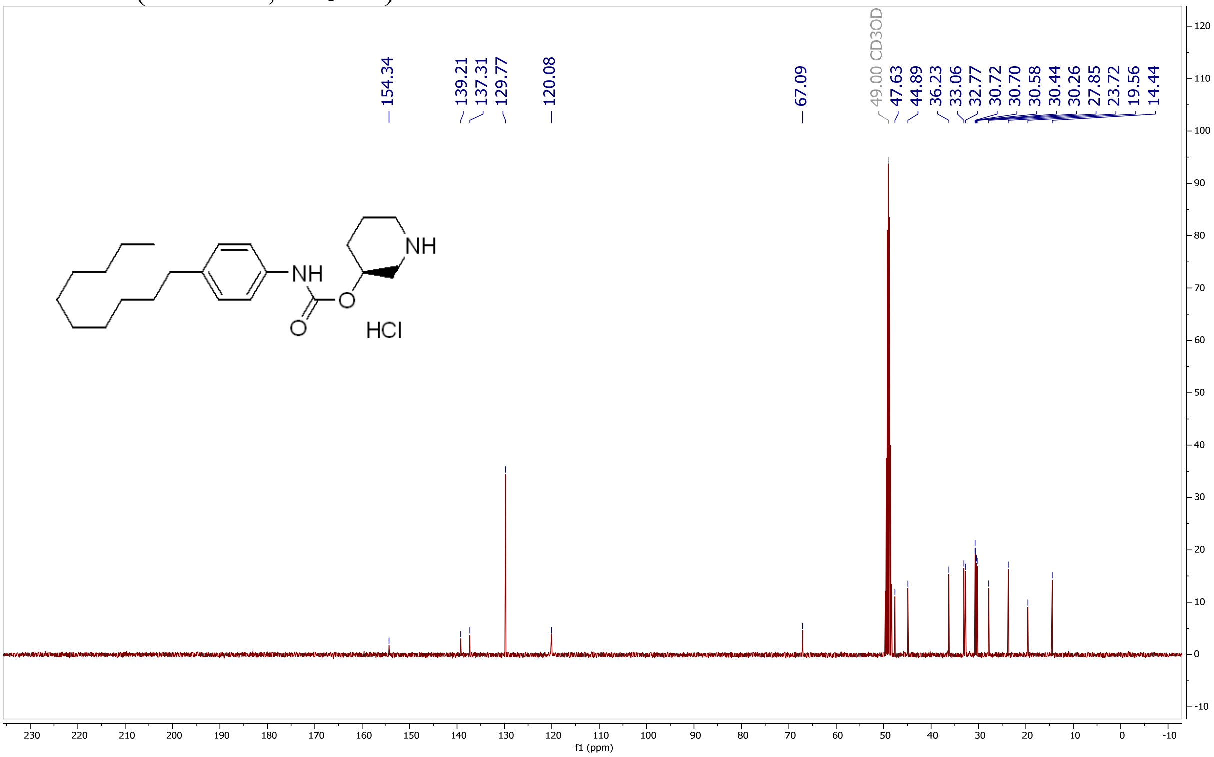

^{1}H NMR (400 MHz, CD_3OD) **8f**

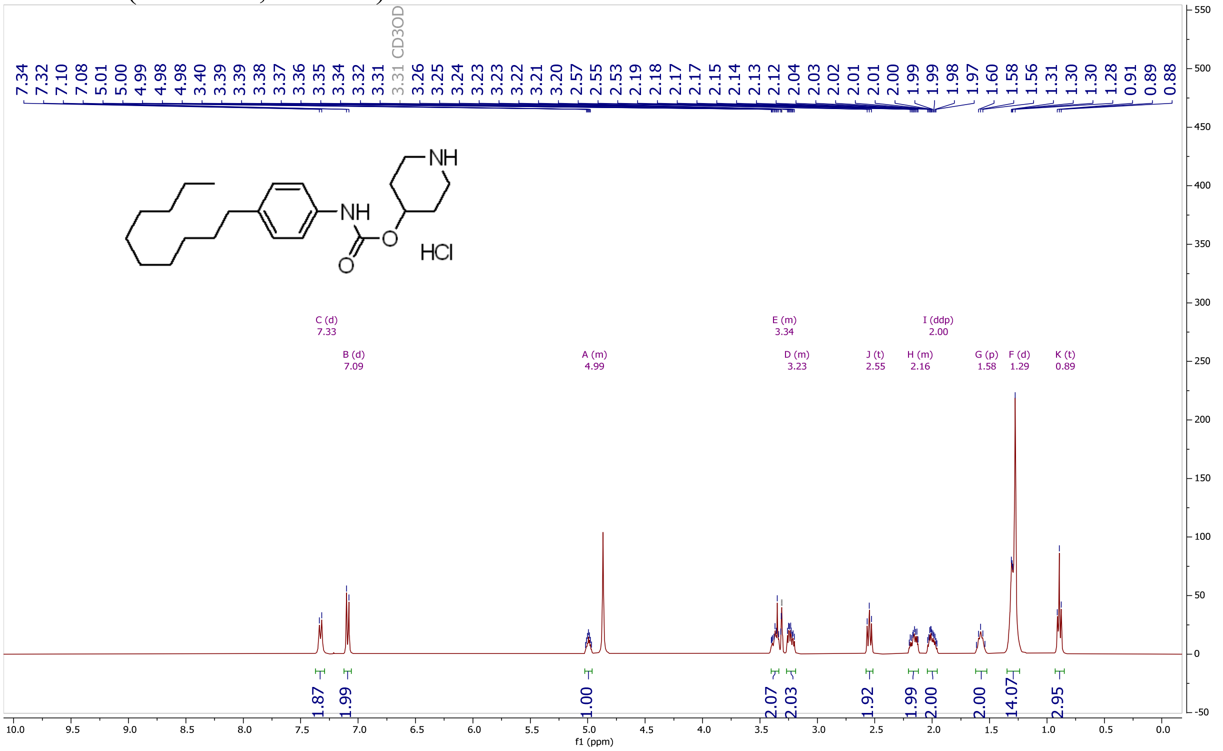

^{13}C NMR (101 MHz, CD_3OD) **8f**

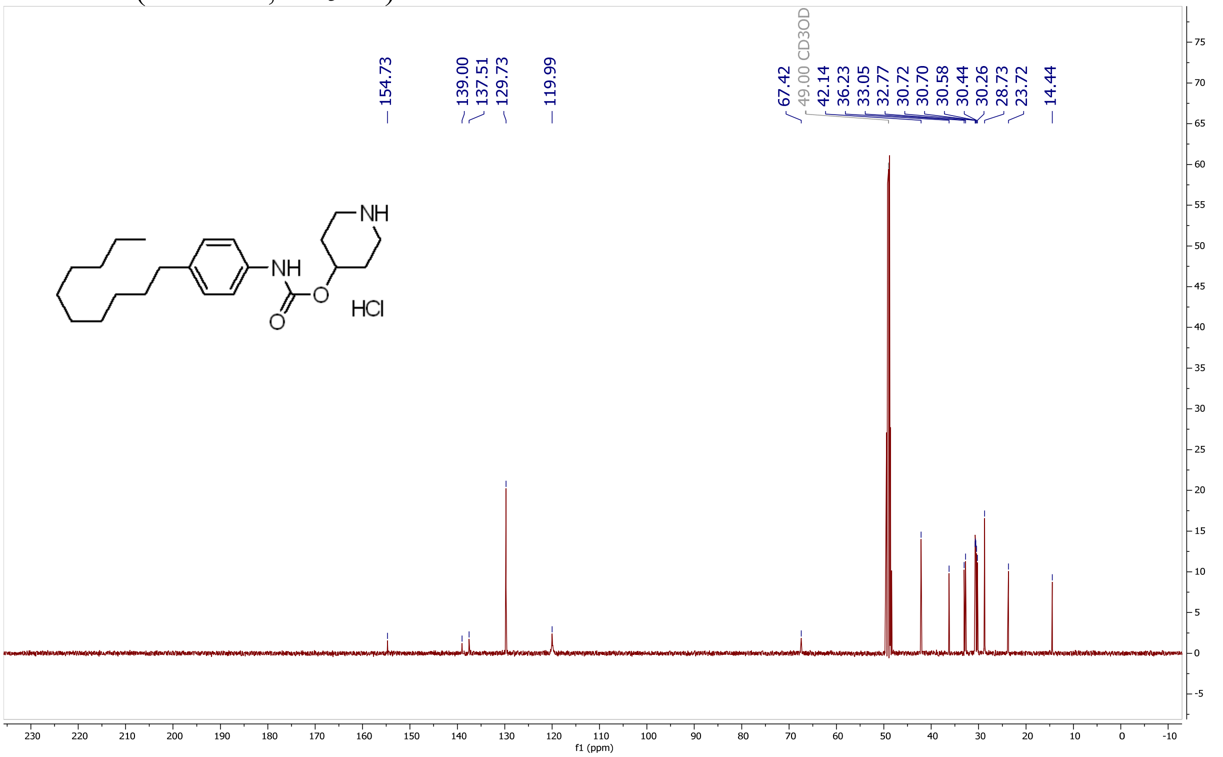

^{1}H NMR (400 MHz, CD_3OD) **11a**

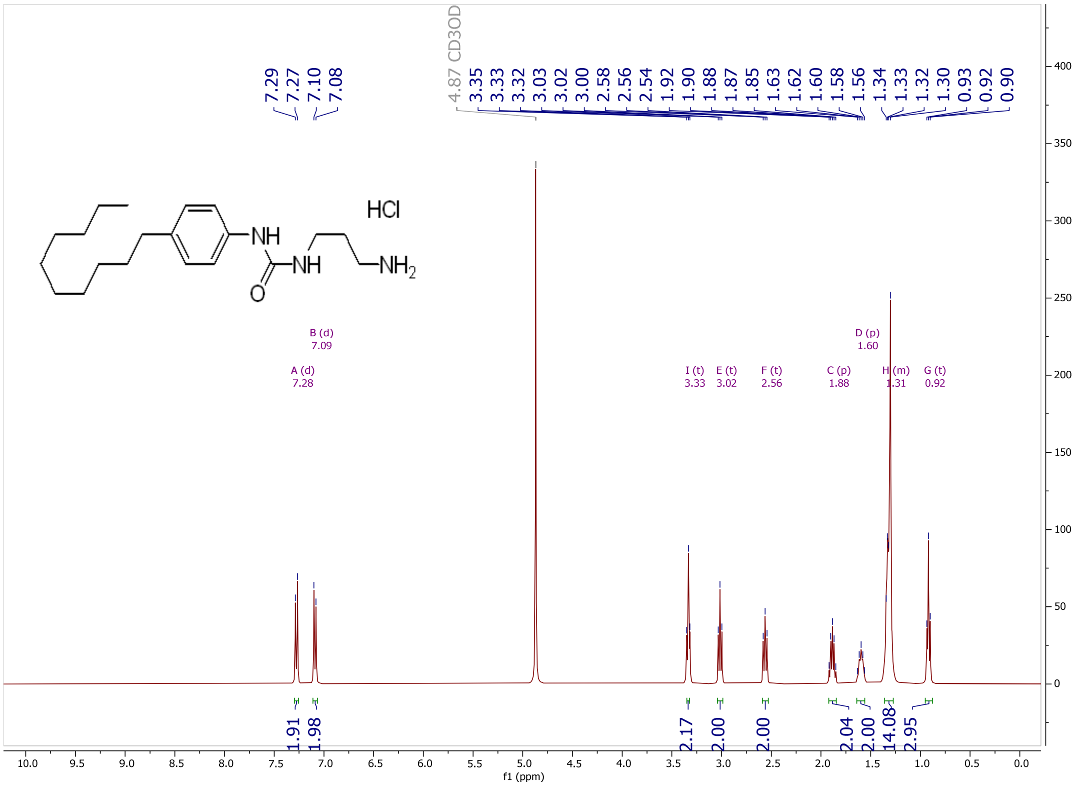

^{13}C NMR (101 MHz, CD_3OD) **11a**

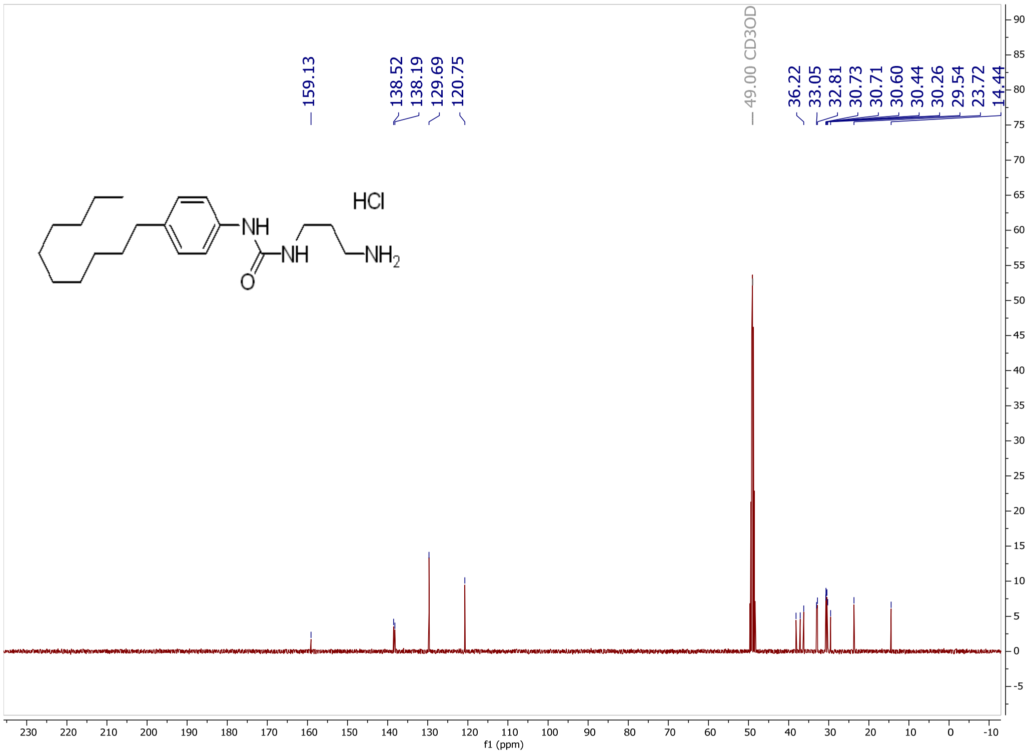

^{1}H NMR (400 MHz, CD_3OD) **11b**

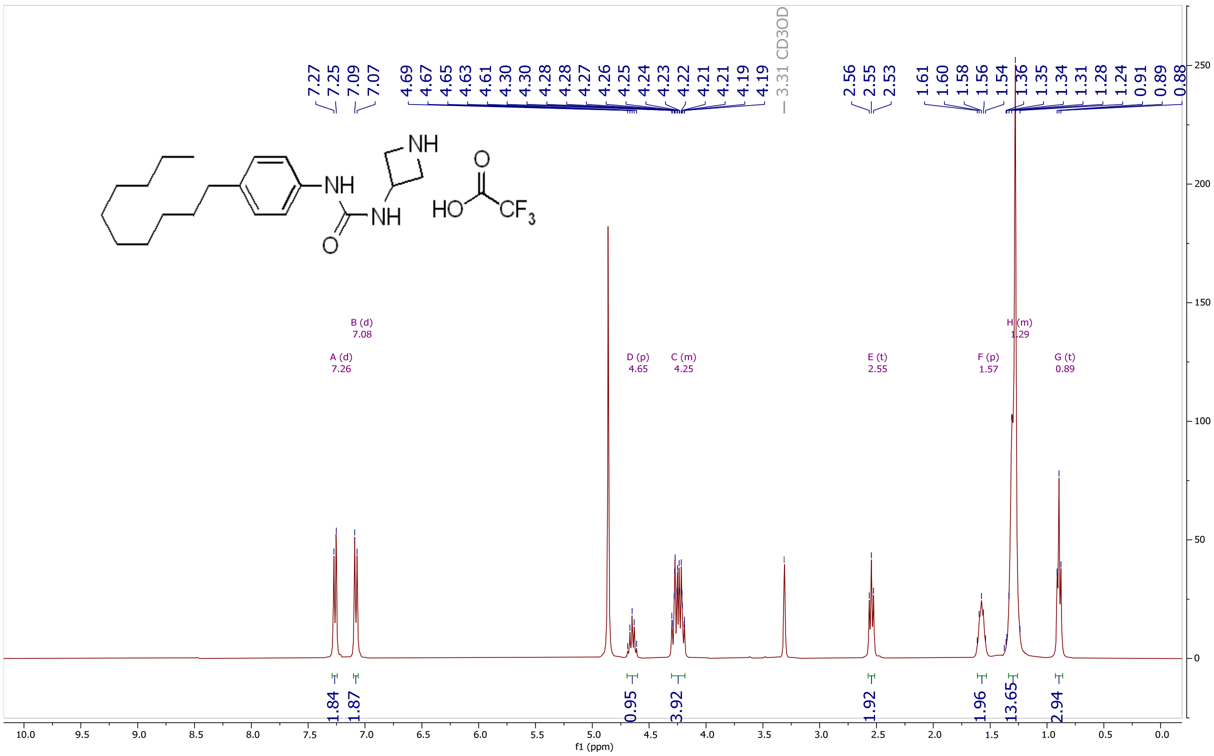

^{13}C NMR (101 MHz, CD_3OD) **11b**

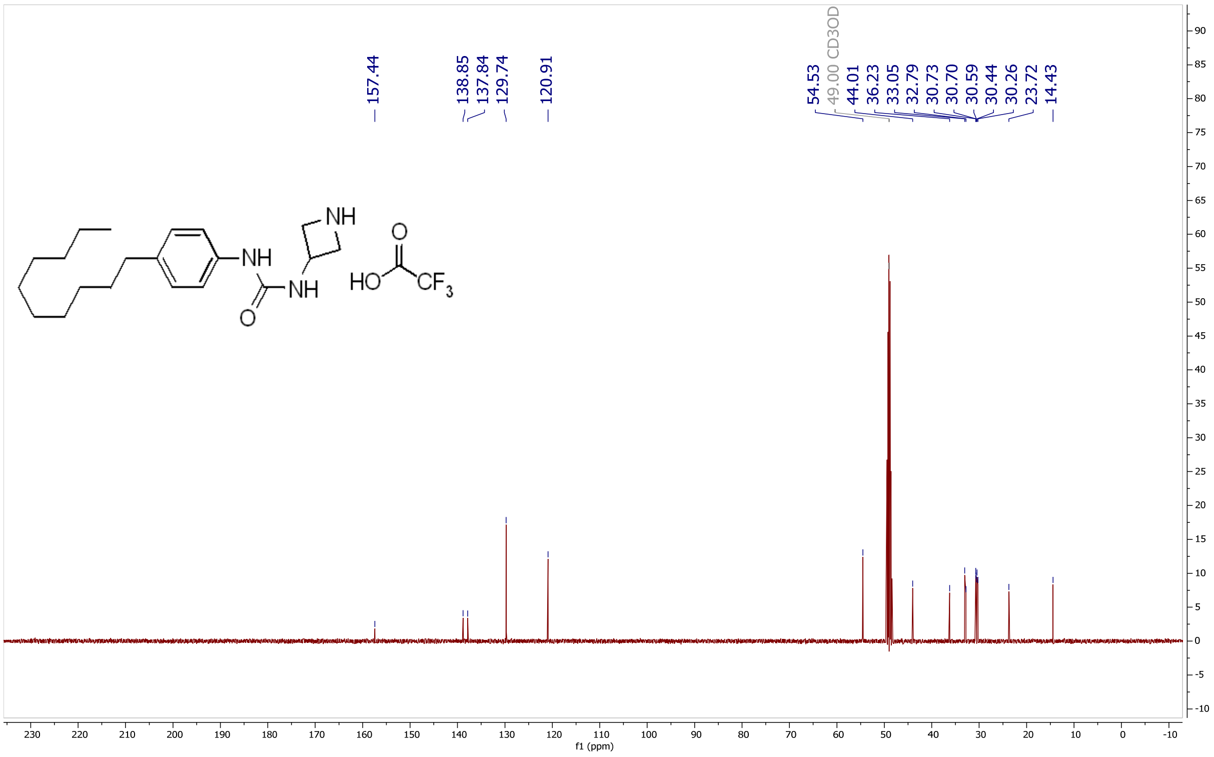

^{1}H NMR (400 MHz, CD_3OD) **11c**

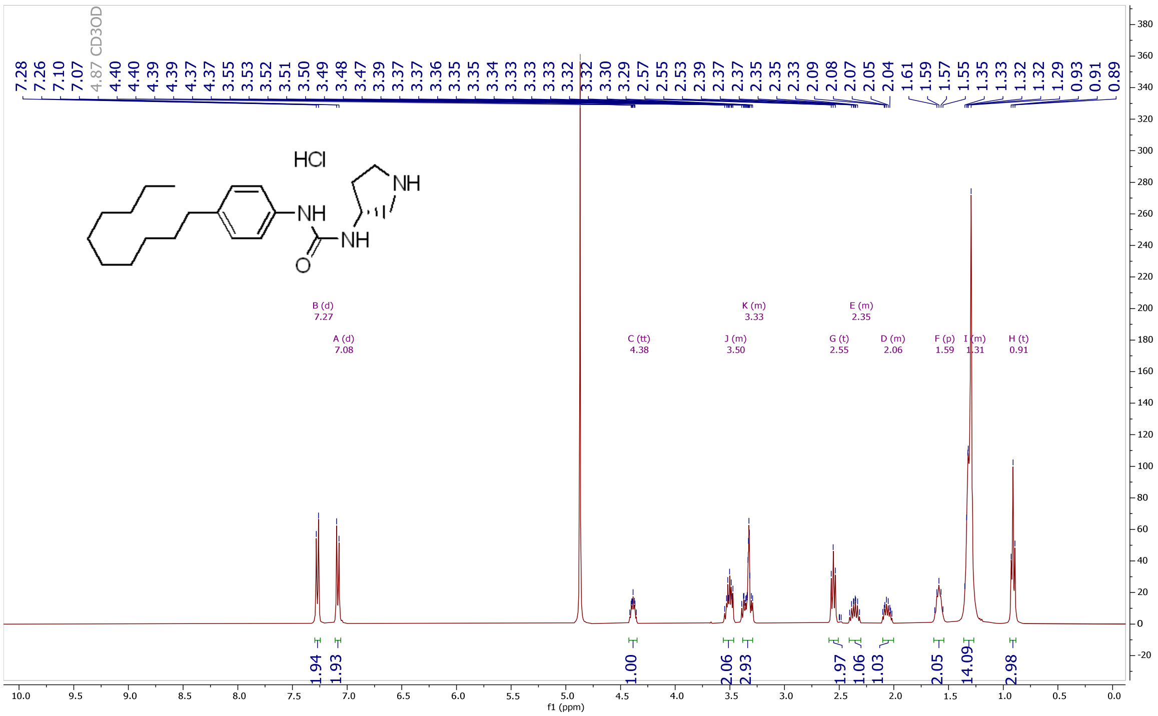

^{13}C NMR (101 MHz, CD_3OD) **11c**

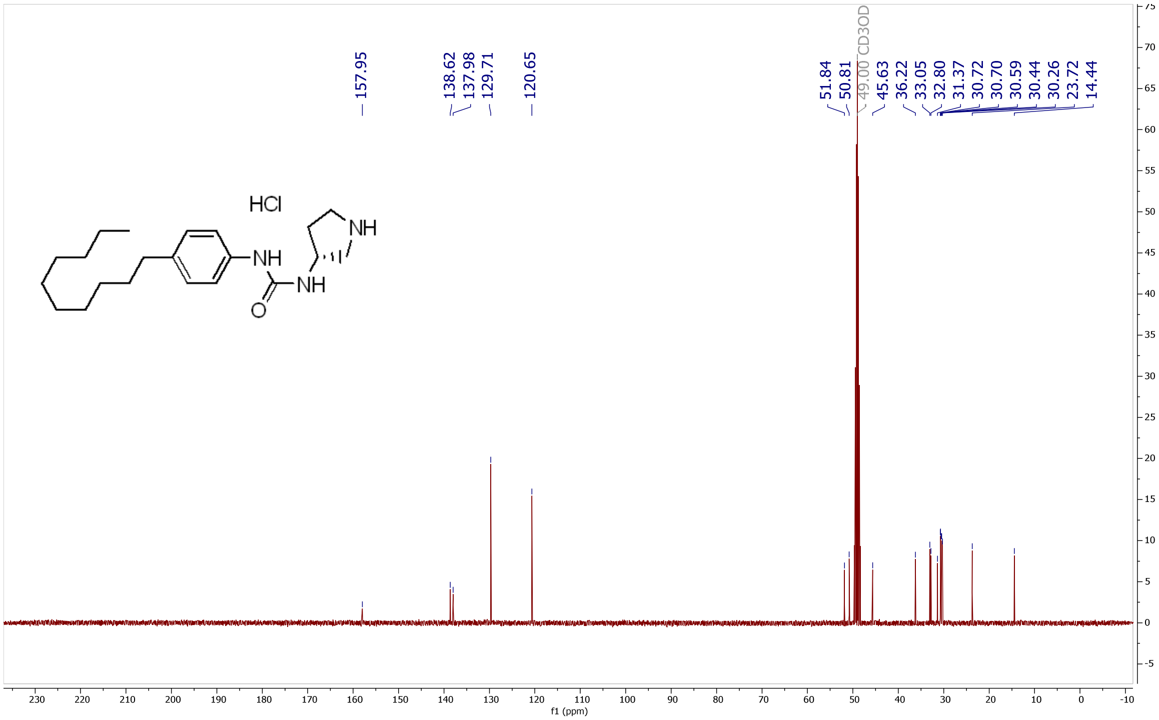

^{1}H NMR (400 MHz, CD_3OD) **11d**

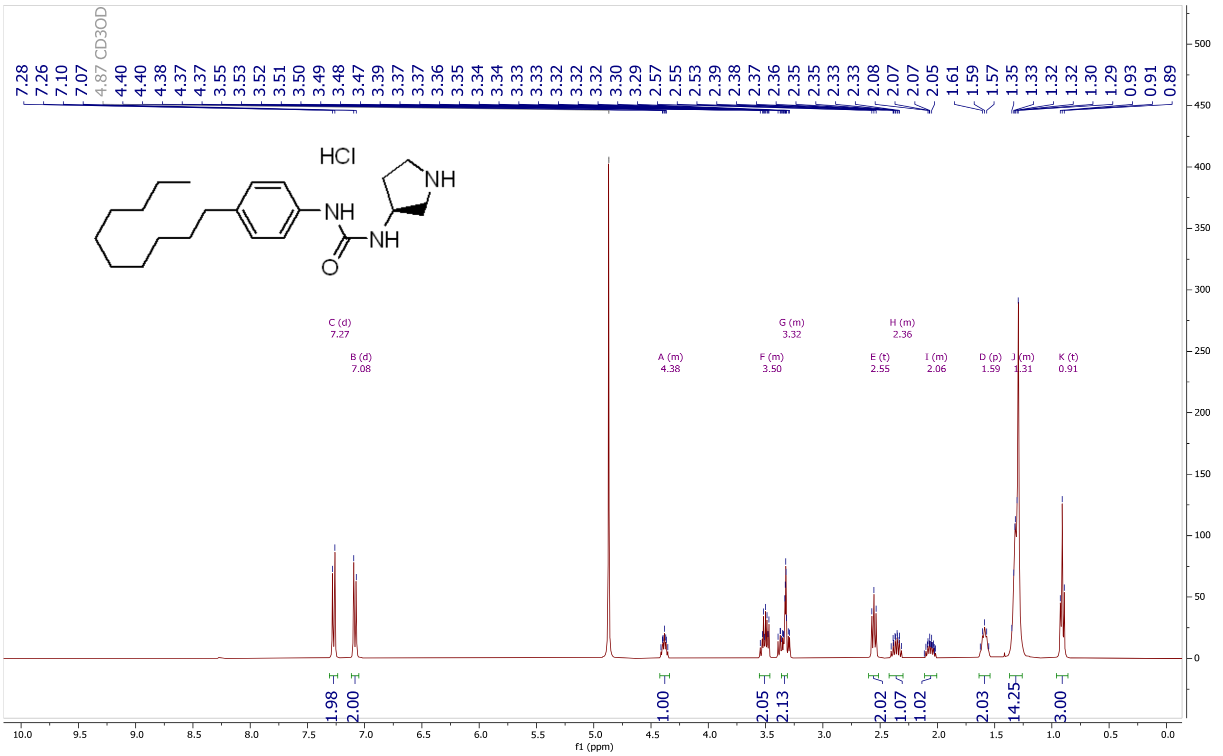

^{13}C NMR (101 MHz, CD_3OD) **11d**

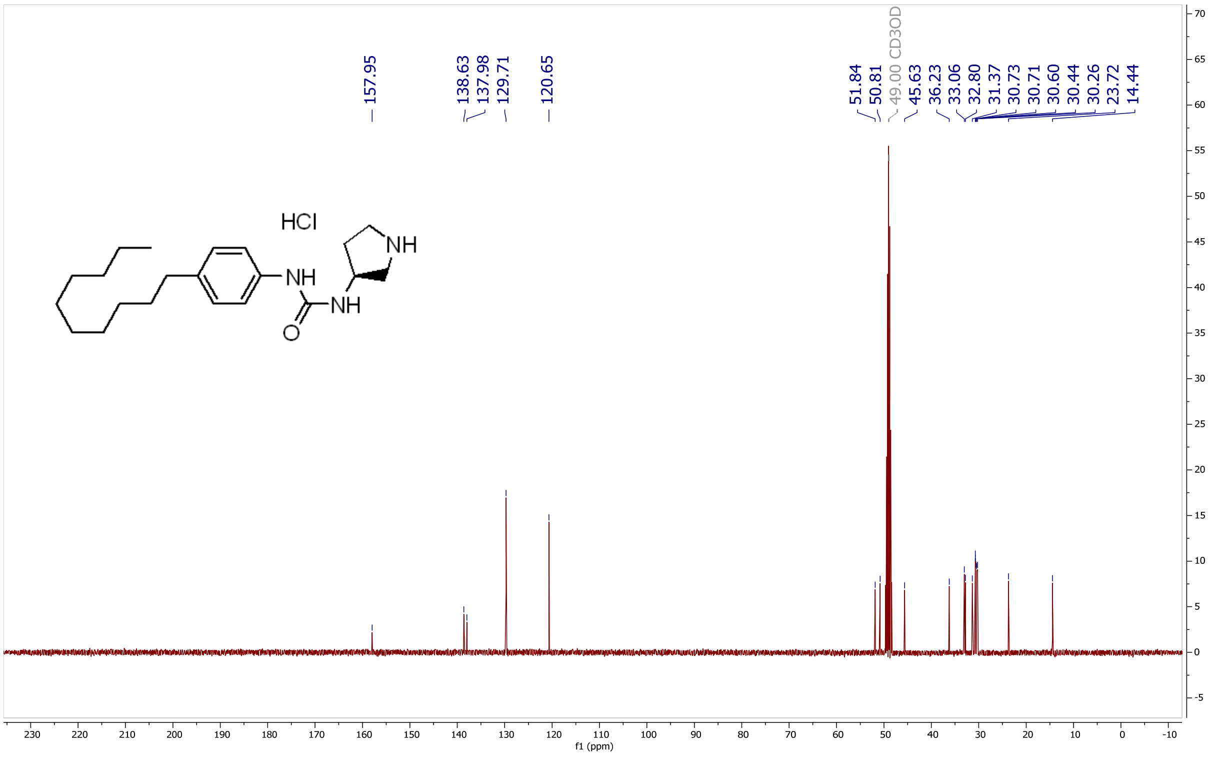

^{1}H NMR (400 MHz, CD_3OD) **11e**

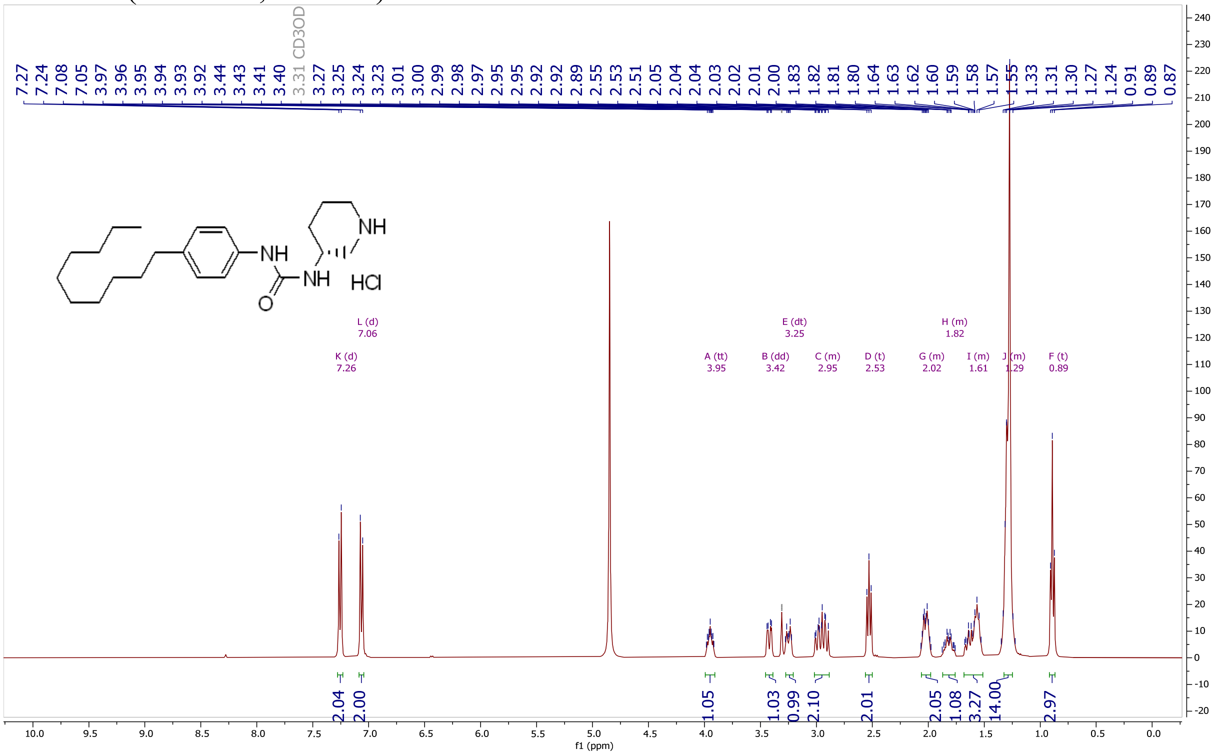

^{13}C NMR (101 MHz, CD_3OD) **11e**

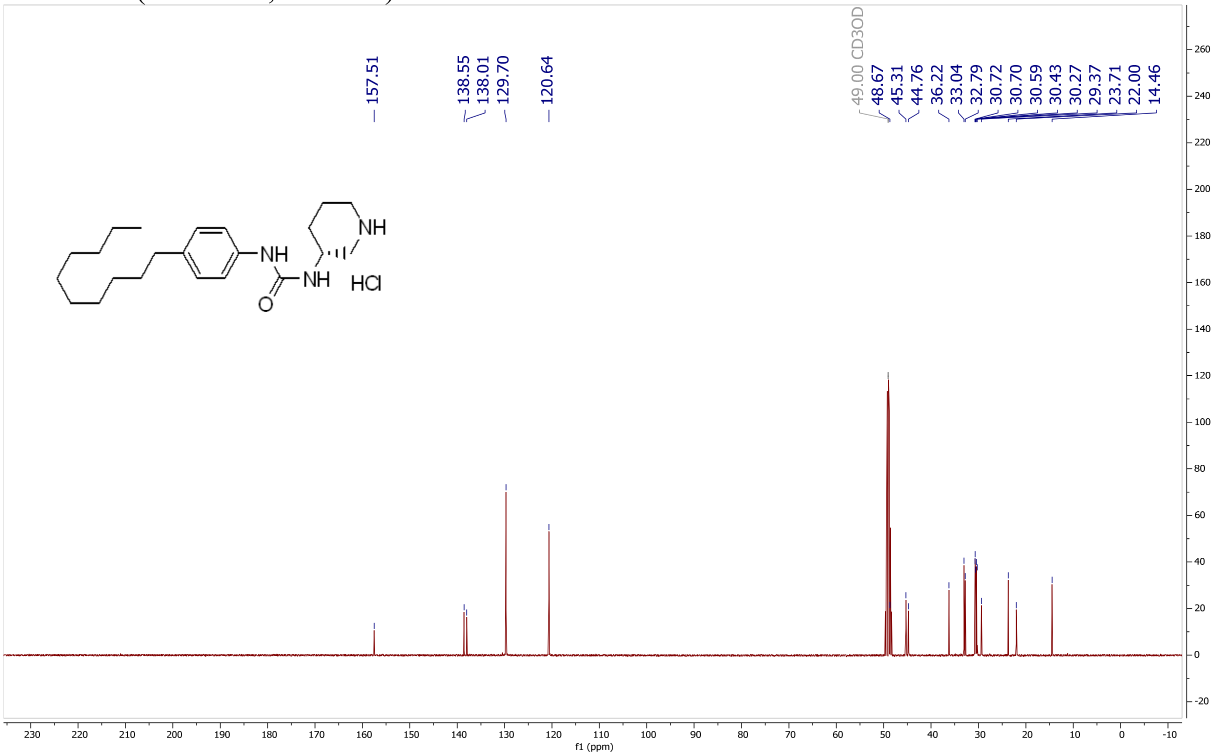

^{1}H NMR (400 MHz, CD_3OD) **11f**

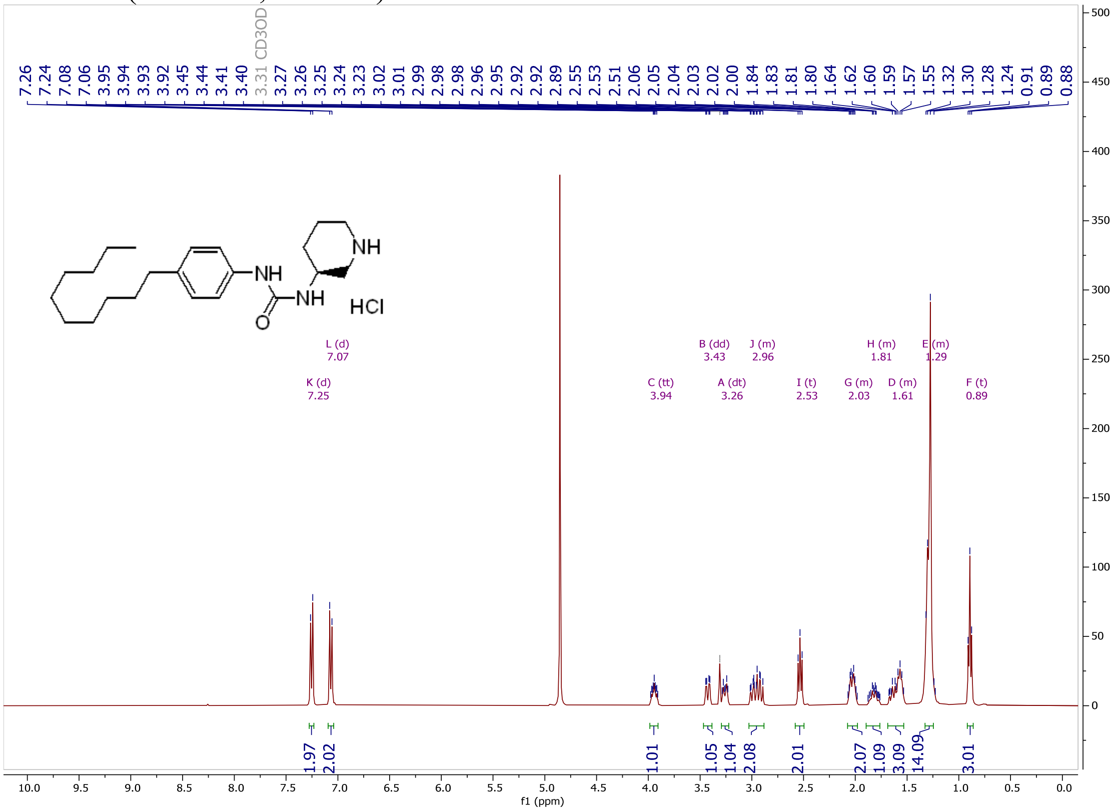

^{13}C NMR (101 MHz, CD_3OD) **11f**

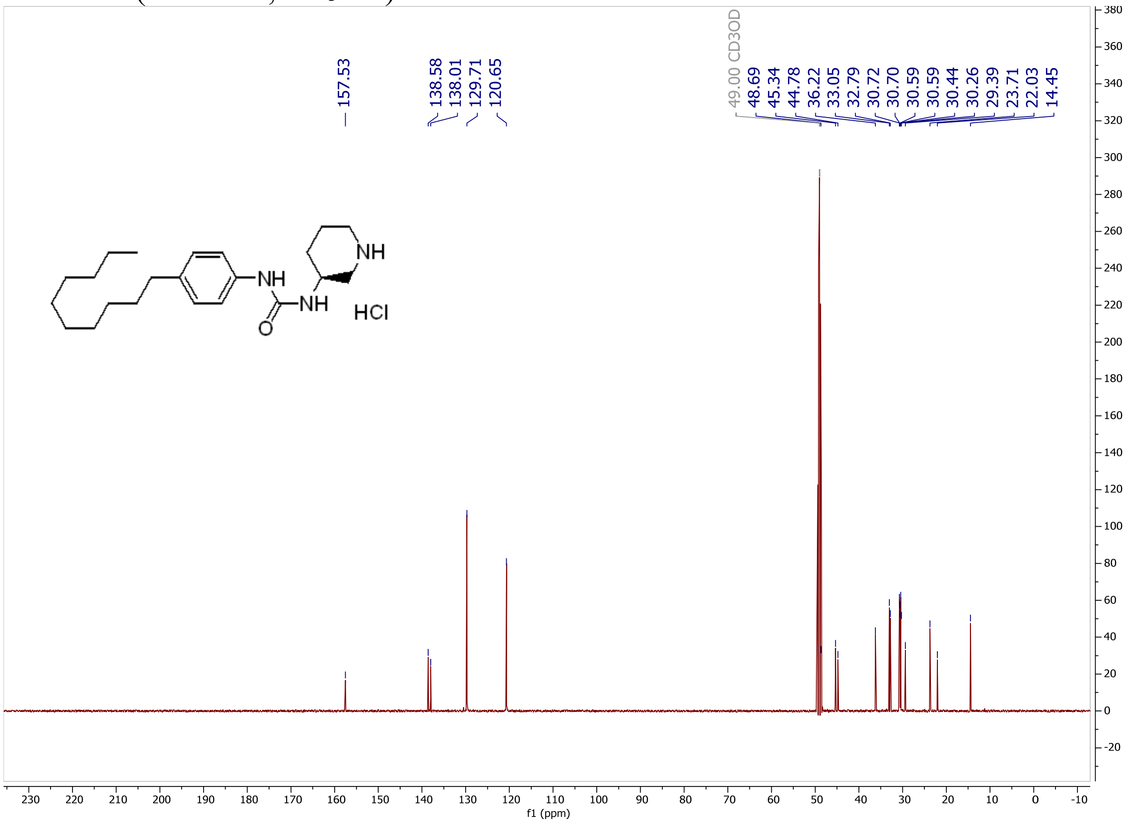

^{1}H NMR (400 MHz, CD_3OD) **11g**

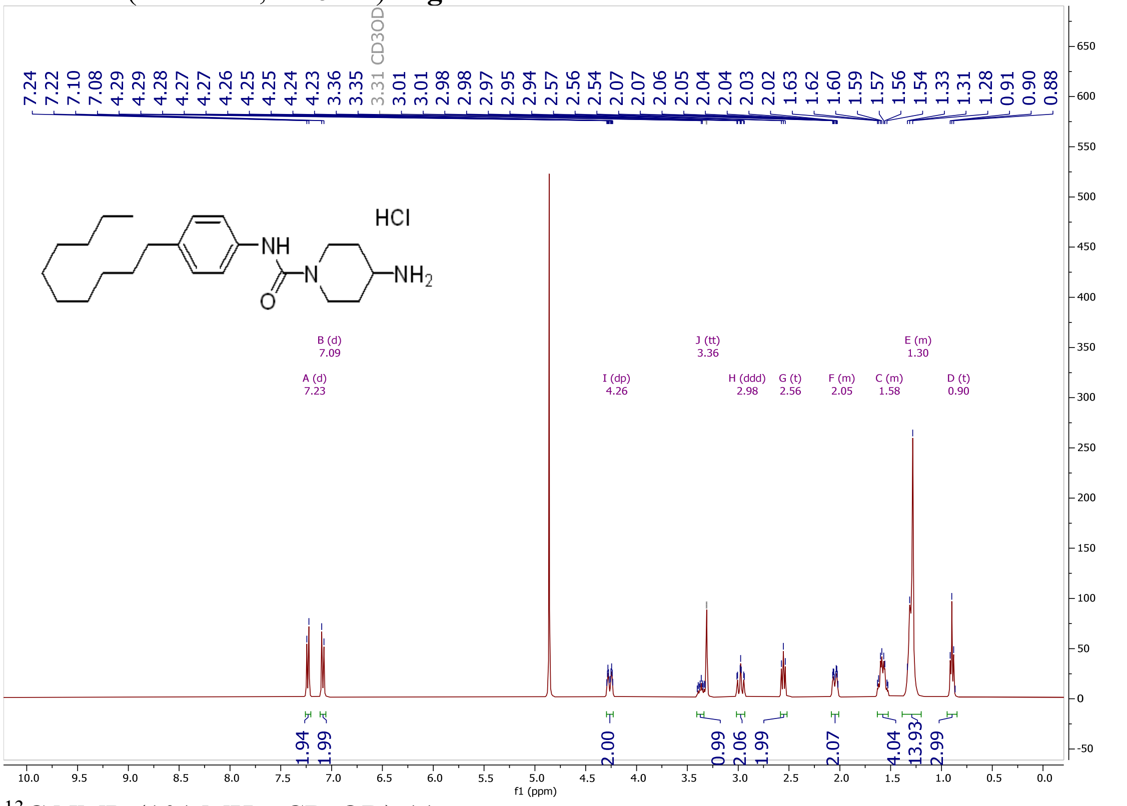

^{13}C NMR (101 MHz, CD_3OD) **11g**

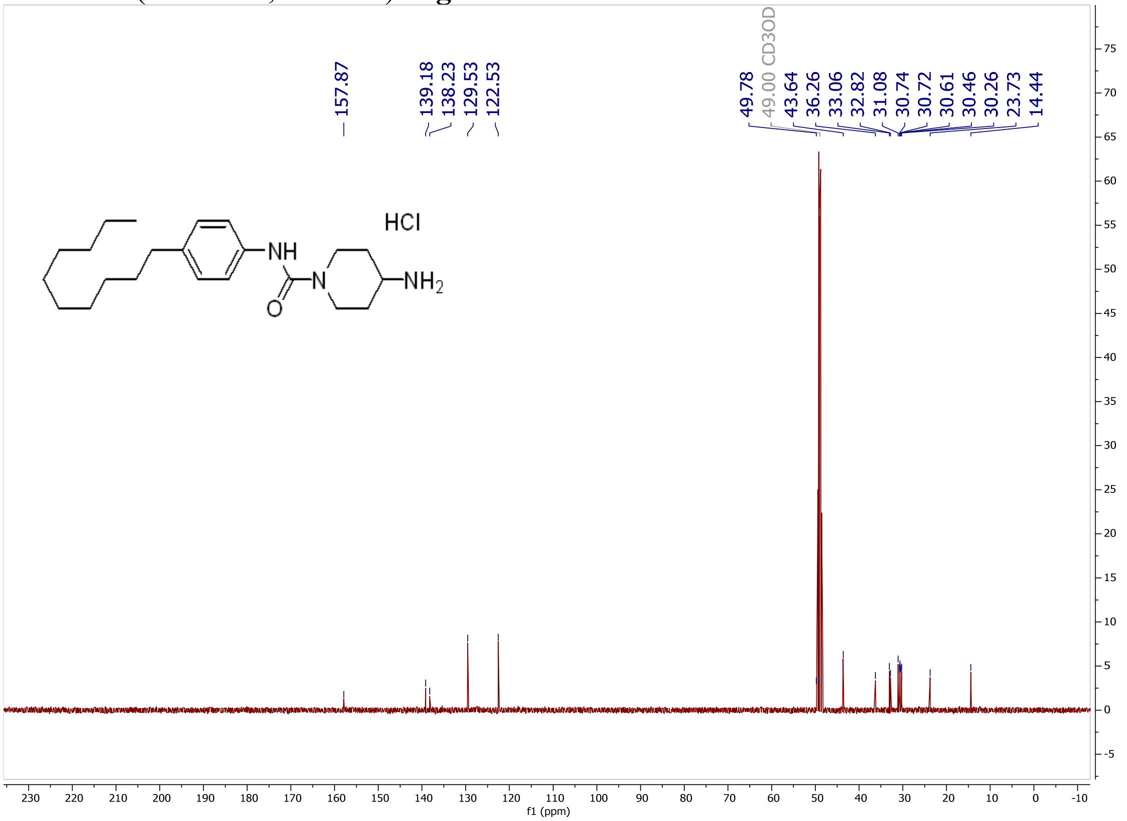

^{1}H NMR (400 MHz, CD_3OD) **11h**

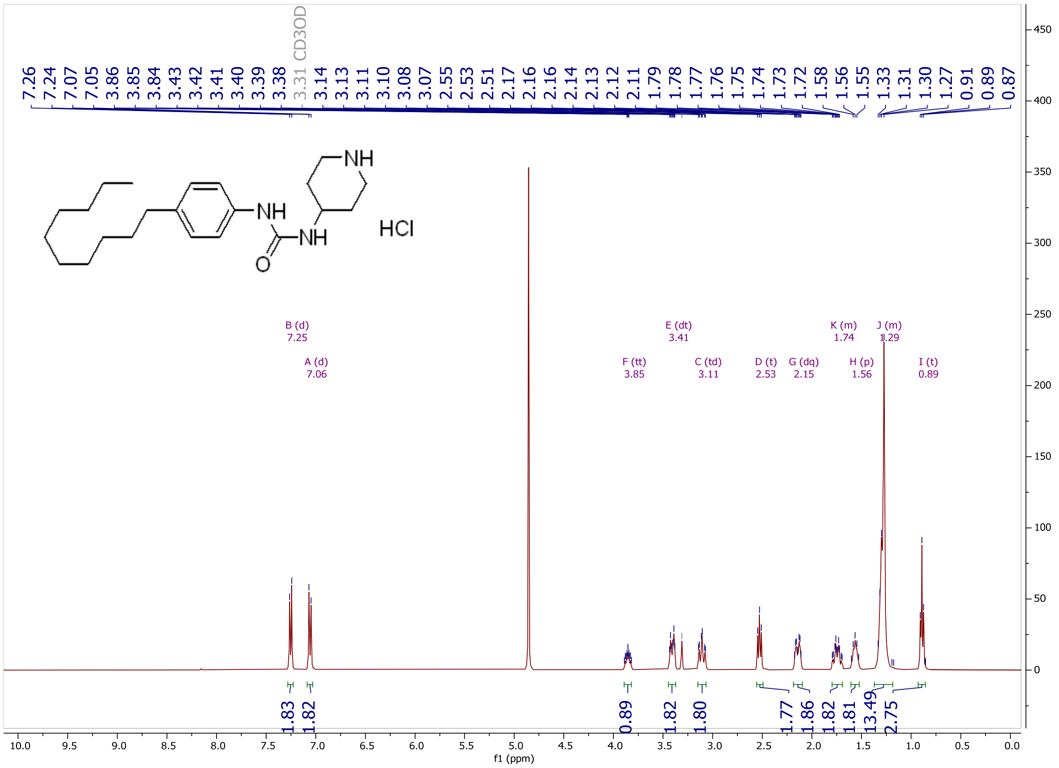

^{13}C NMR (101 MHz, CD_3OD) **11h**

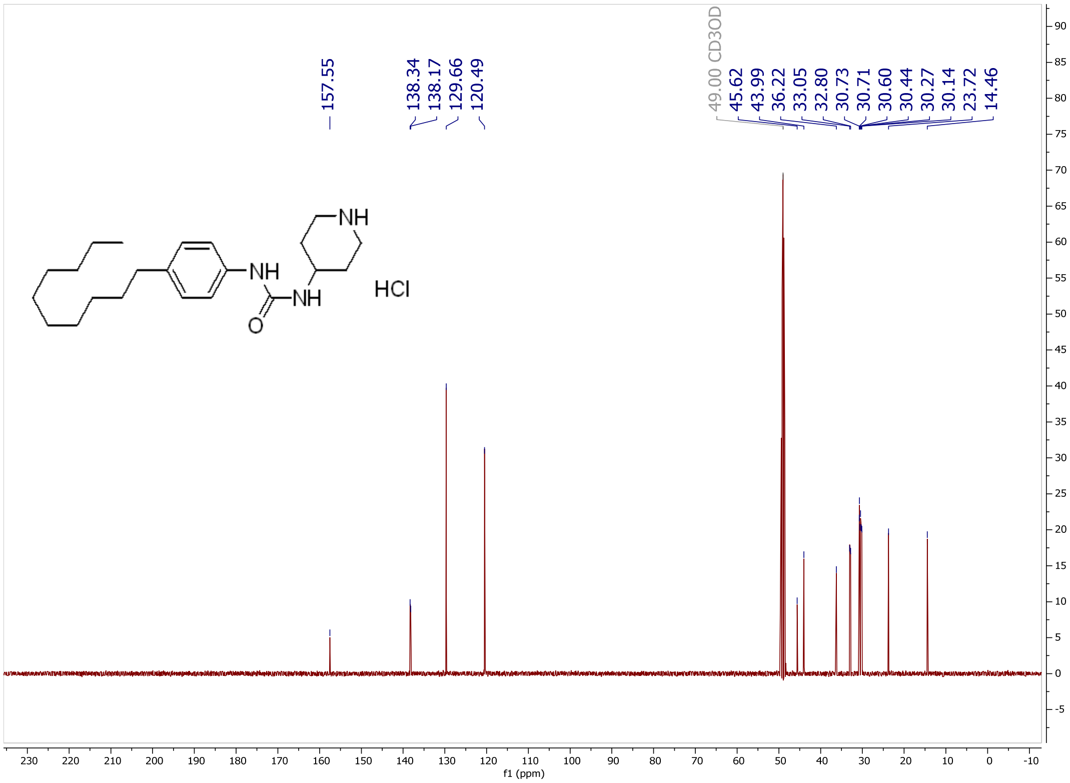

^{1}H NMR (400 MHz, CD_3OD) **11i**

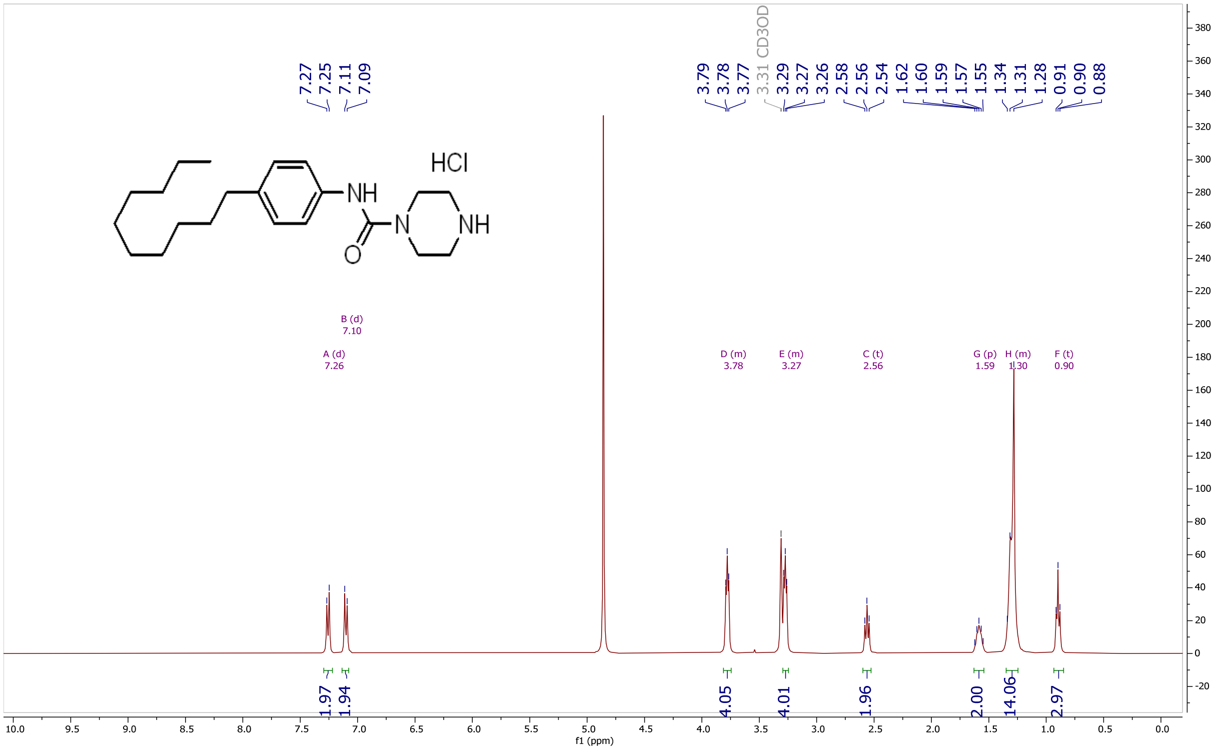

^{13}C NMR (101 MHz, CD_3OD) **11i**

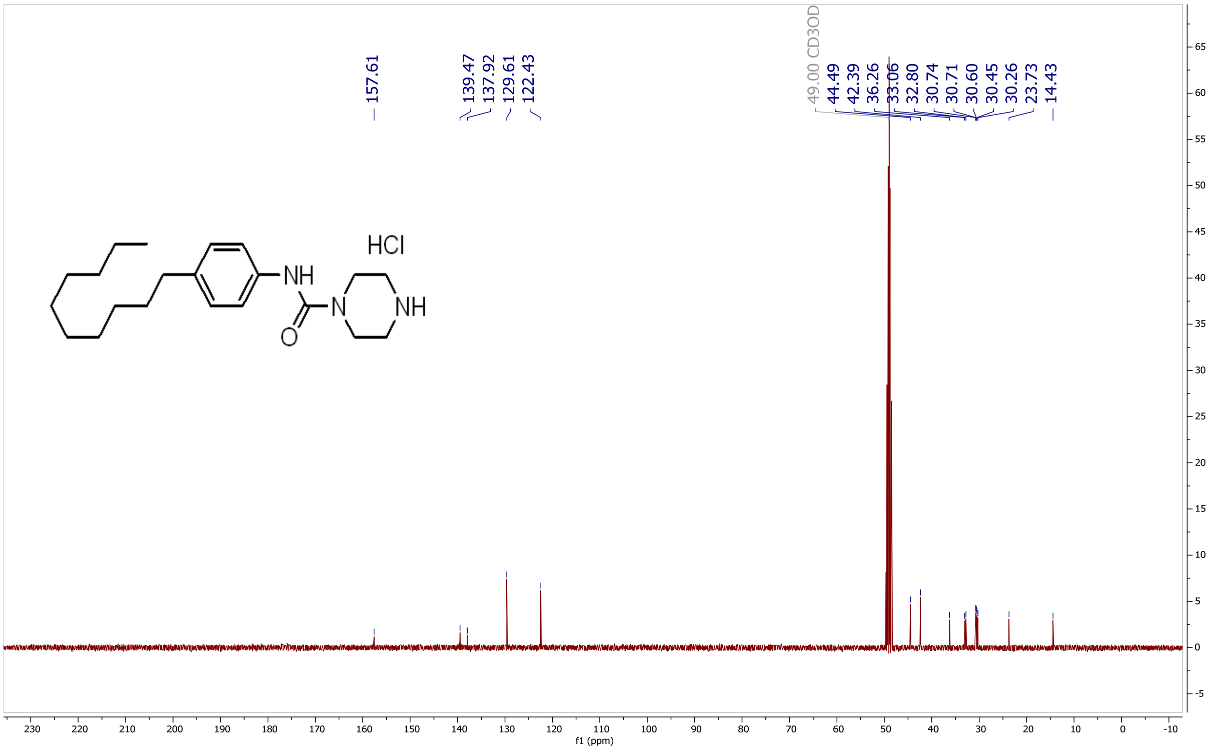

^{1}H NMR (400 MHz, CD_3OD) **11j**

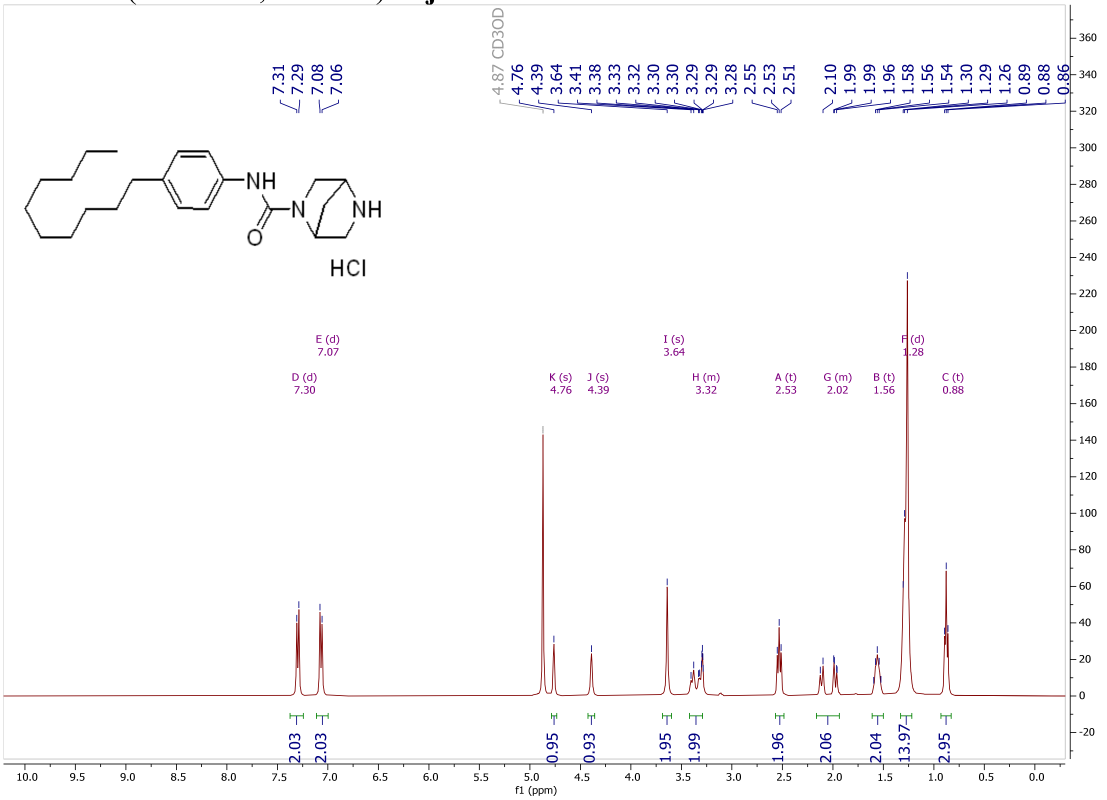

^{13}C NMR (101 MHz, CD_3OD) **11j**

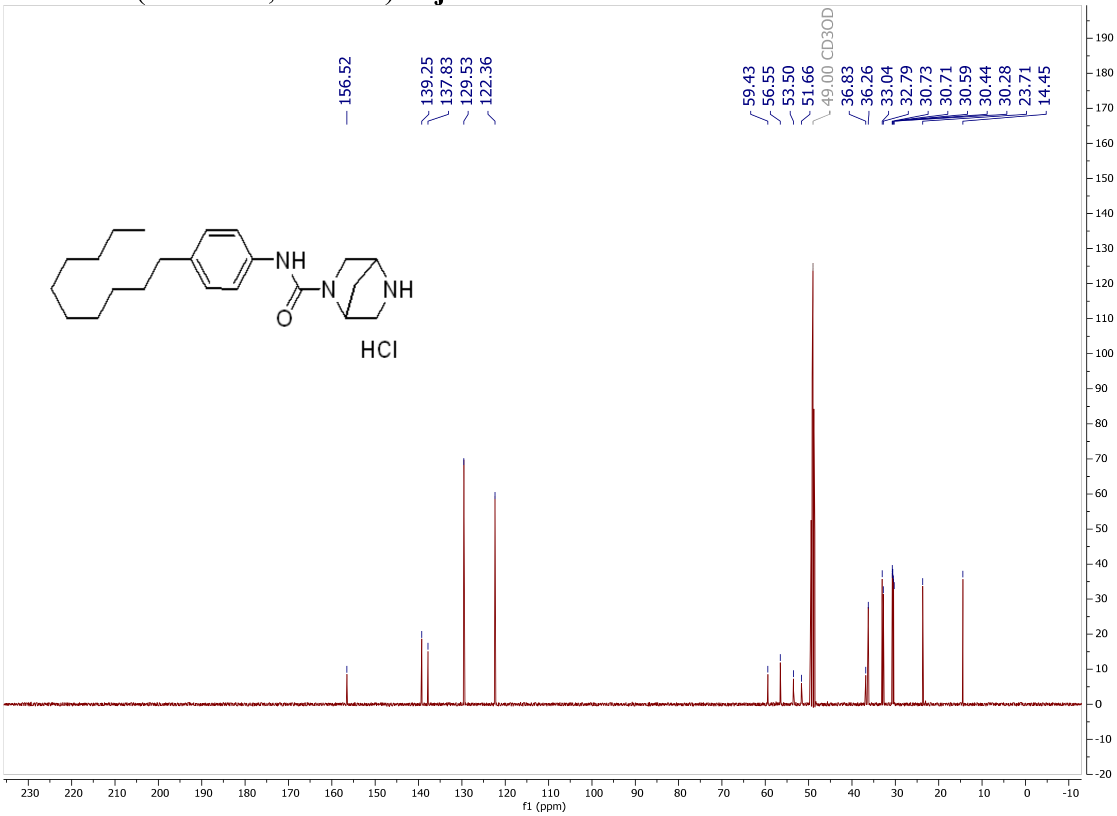

^{1}H NMR (400 MHz, CD_3OD) **11k**

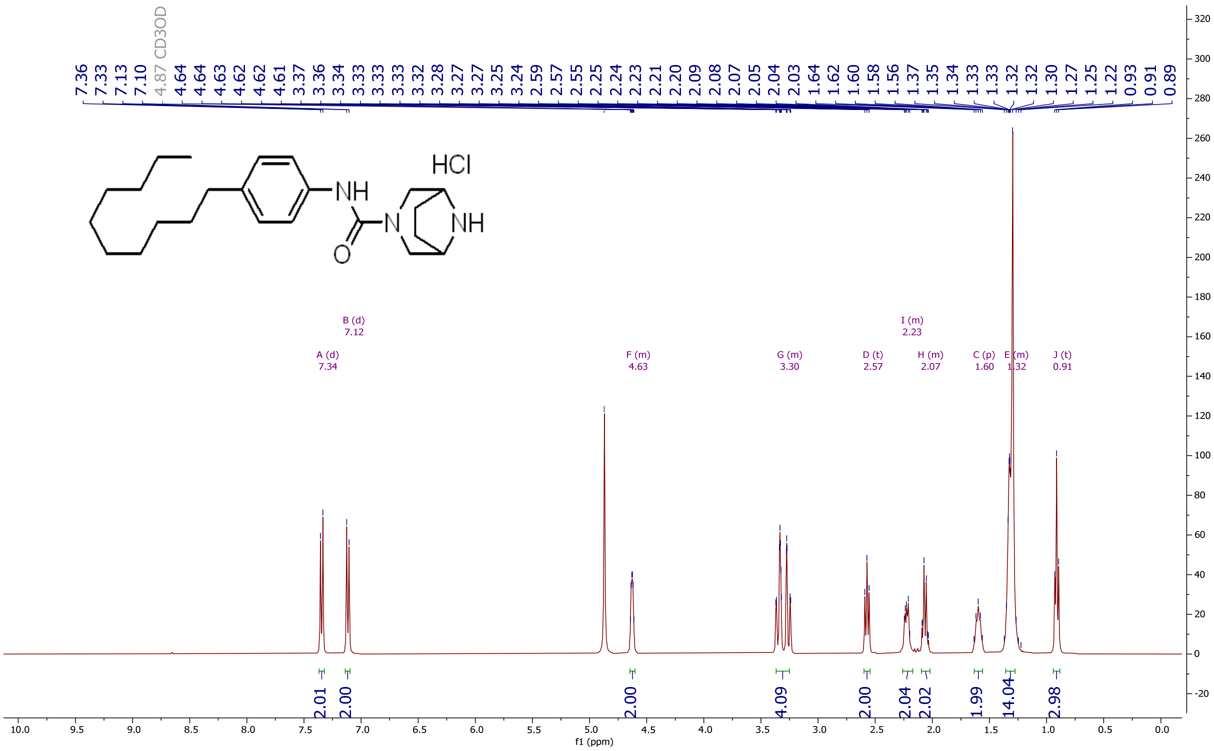

^{13}C NMR (101 MHz, CD_3OD) **11k**

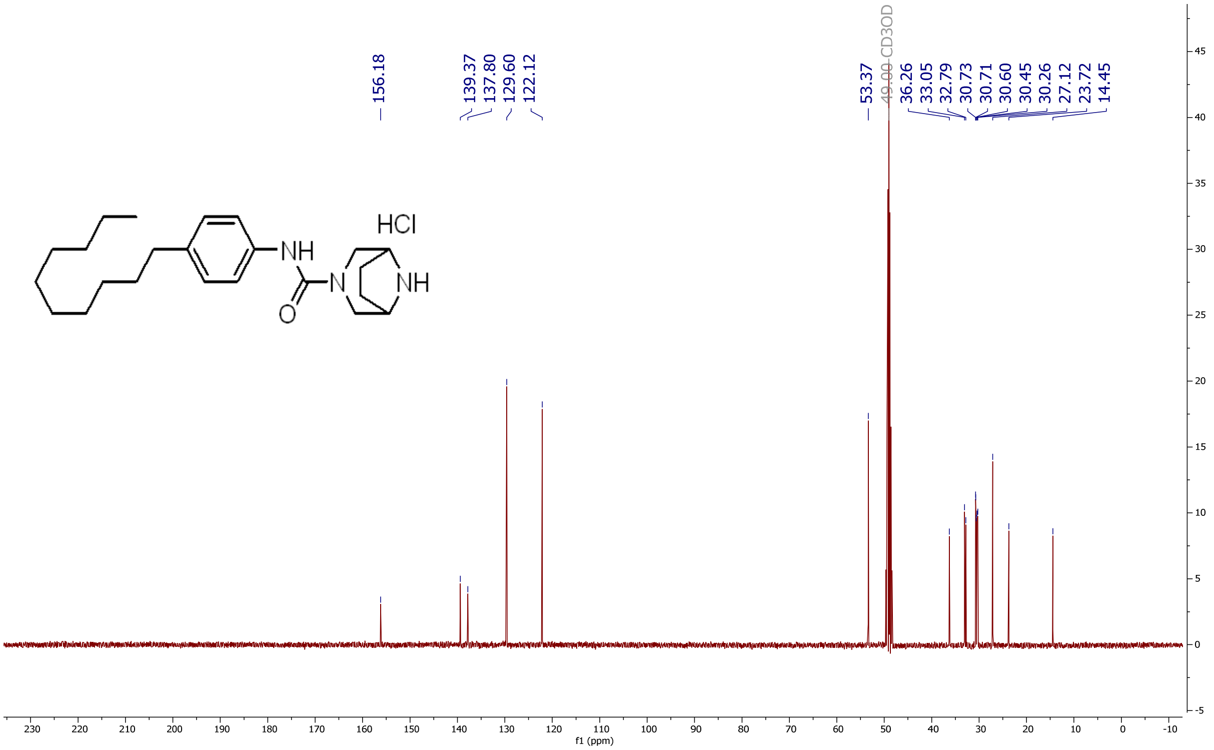

^{1}H NMR (400 MHz, CD_3OD) **11l**

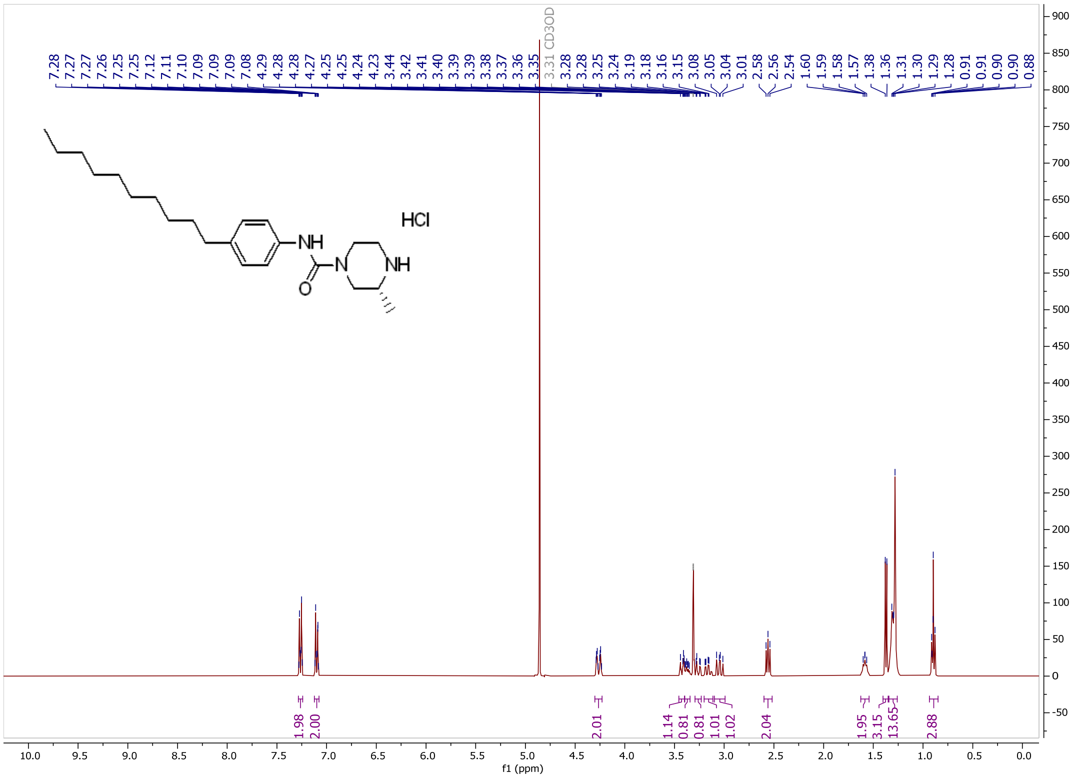

^{13}C NMR (151 MHz, CD_3OD) **11l**

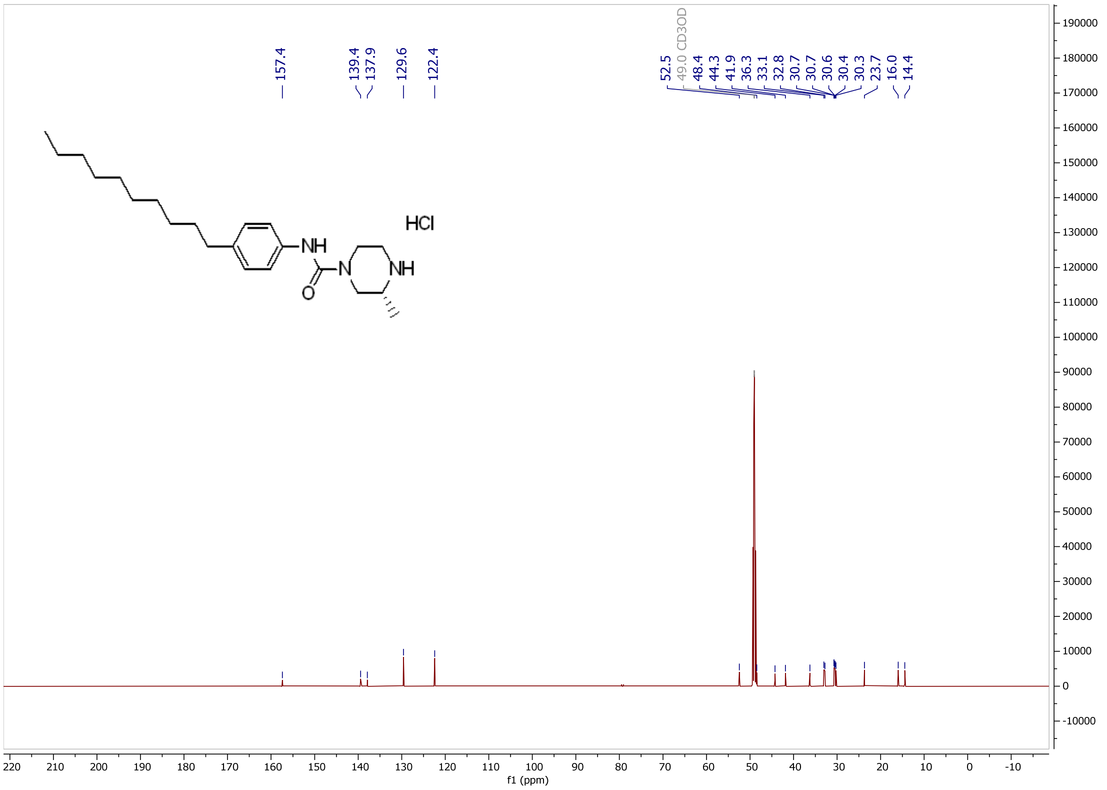

^{1}H NMR (600 MHz, CD_3OD) **11m**

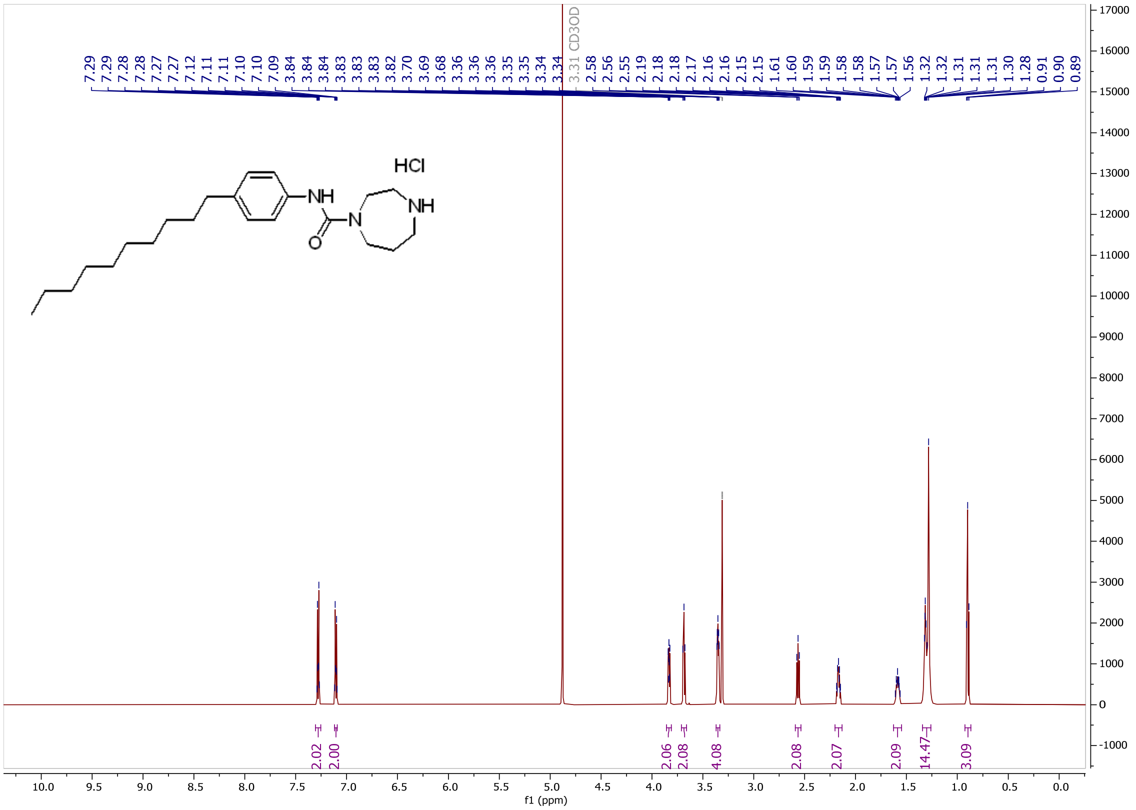

^{13}C NMR (151 MHz, CD_3OD) **11m**

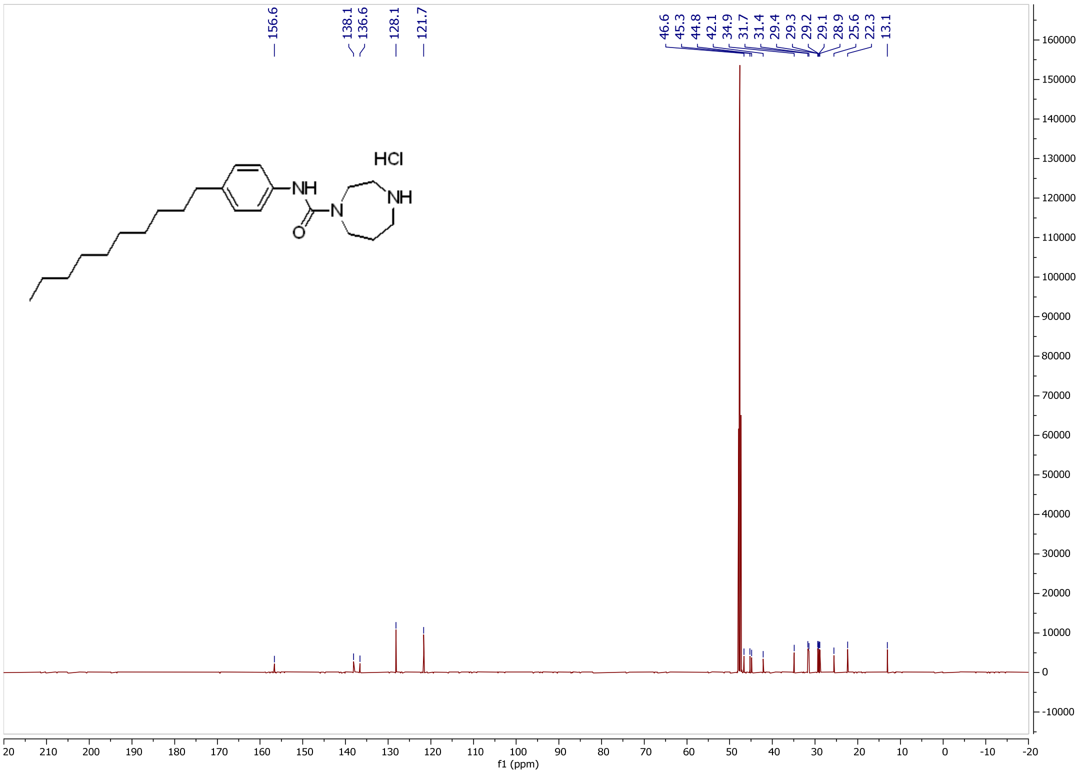

^{1}H NMR (400 MHz, CD_3OD) **13a**

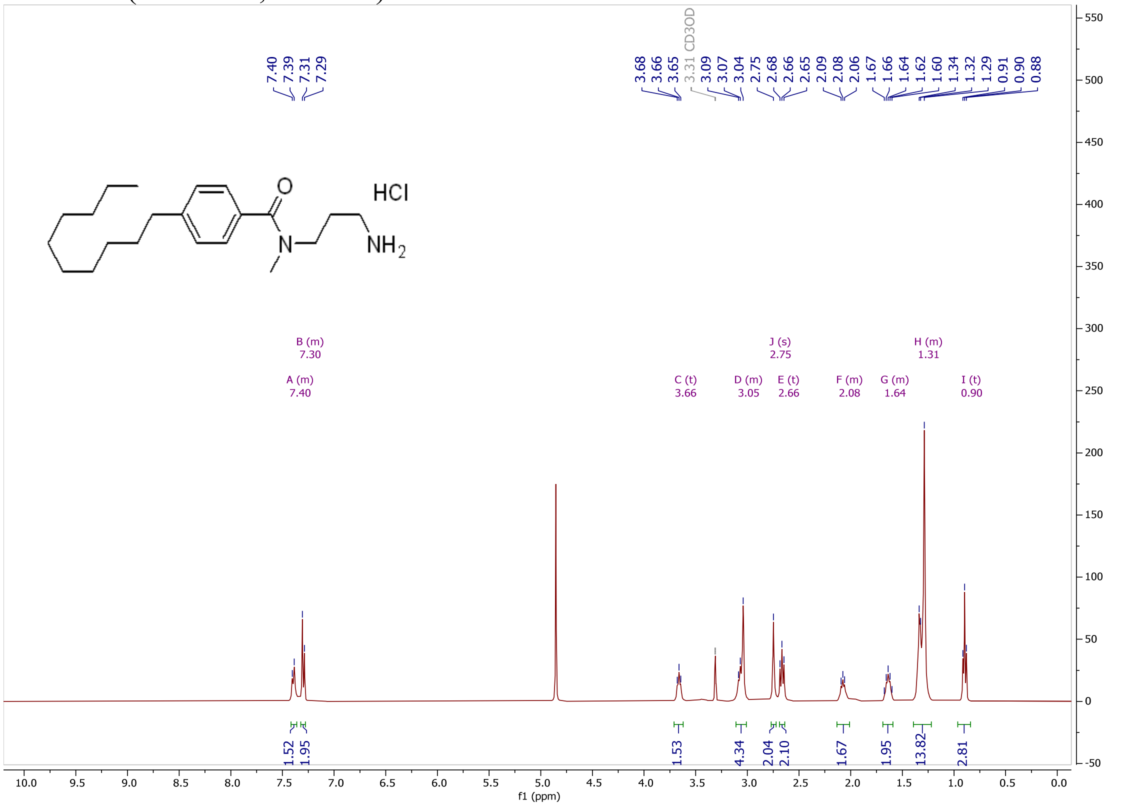

^{13}C NMR (101 MHz, CD_3OD) **13a**

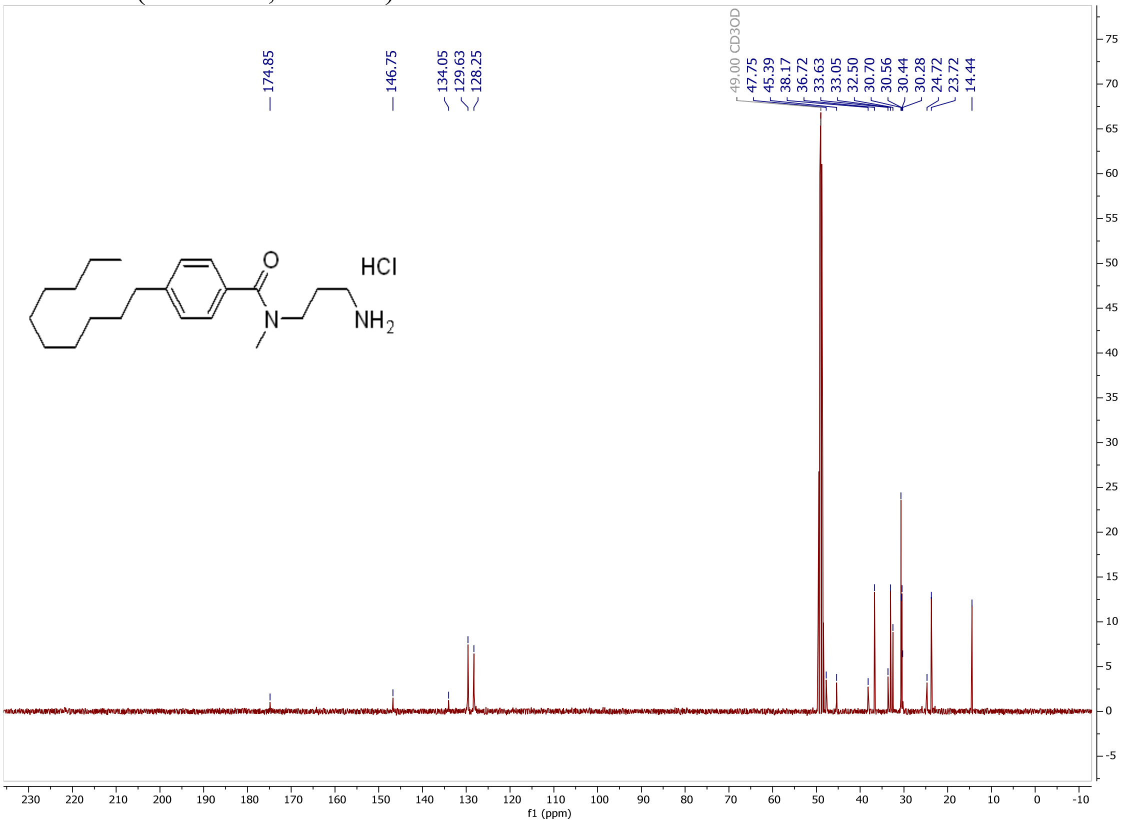

^{1}H NMR (400 MHz, CD_3OD) **13b**

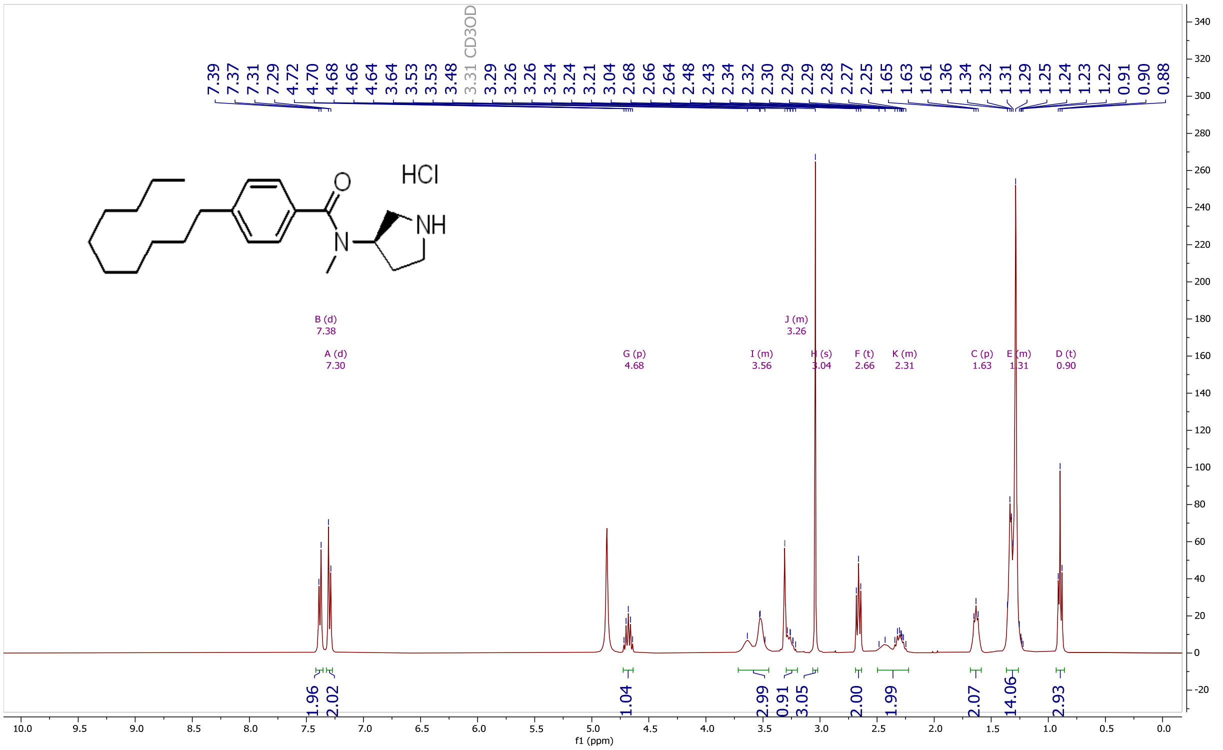

^{13}C NMR (101 MHz, CD_3OD) **13b**

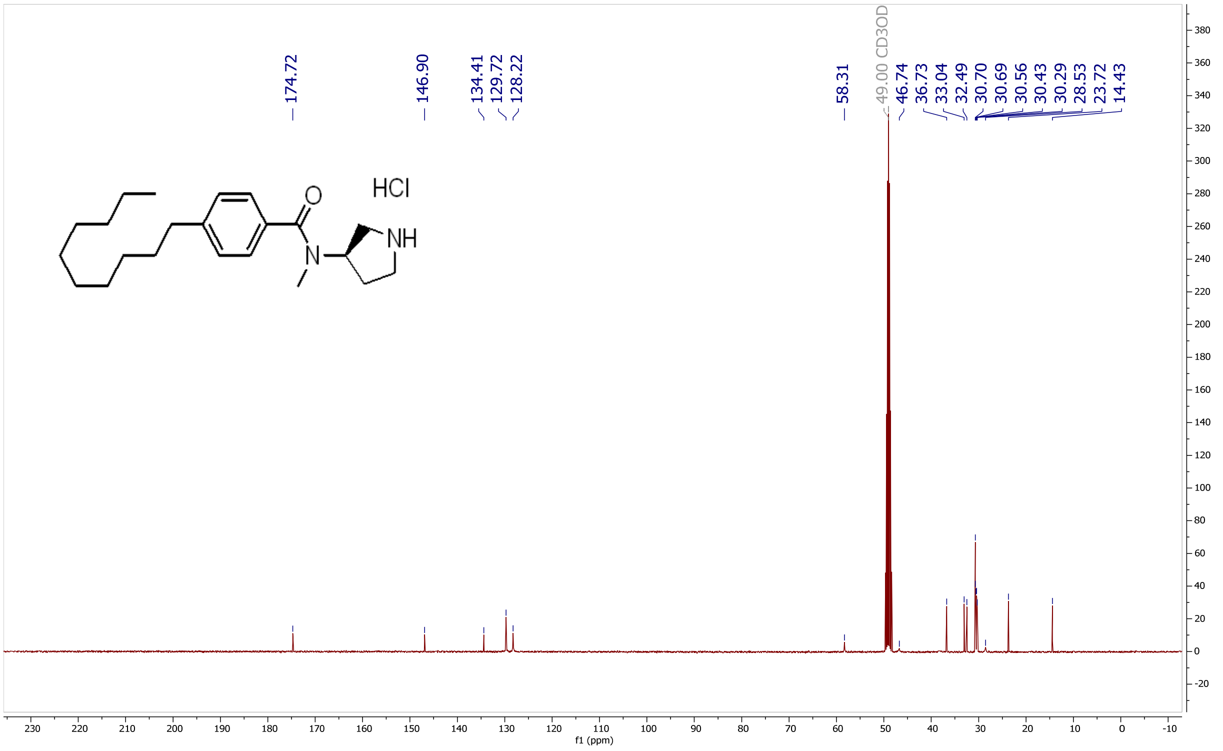

^{1}H NMR (400 MHz, CD_3OD) **13c**

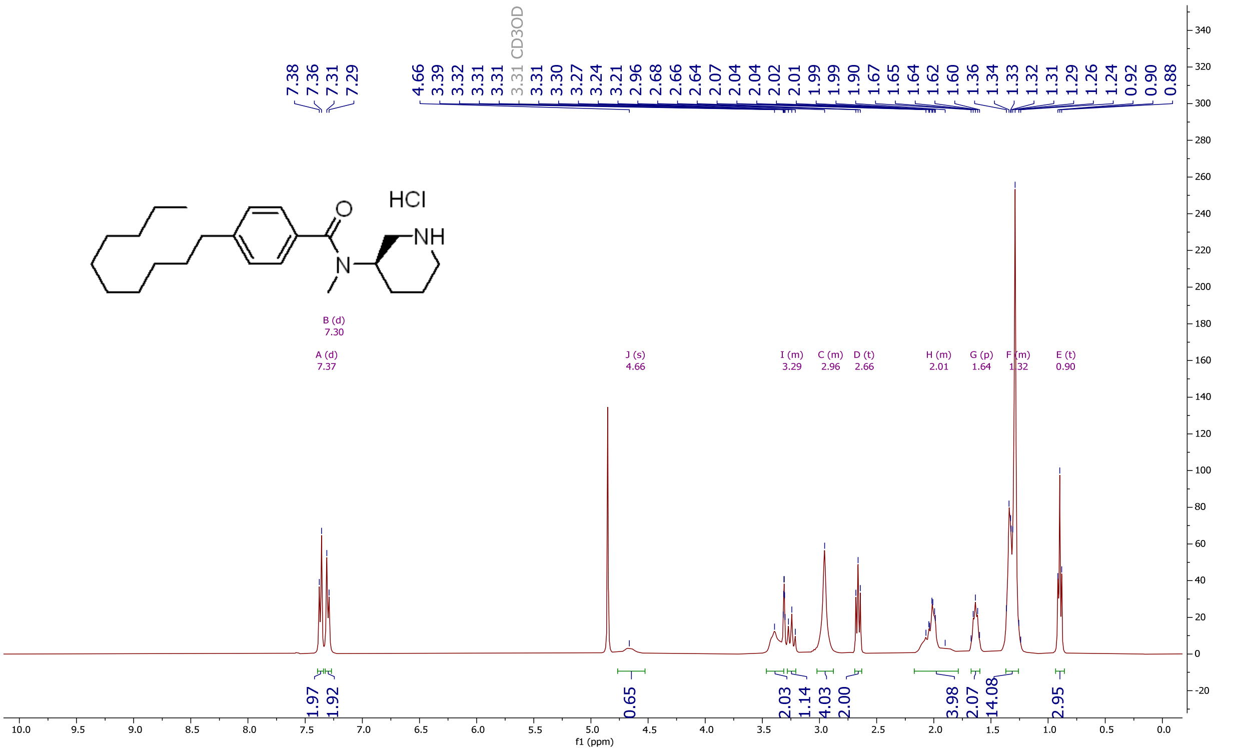

^{13}C NMR (126 MHz, CD_3OD) **13c**

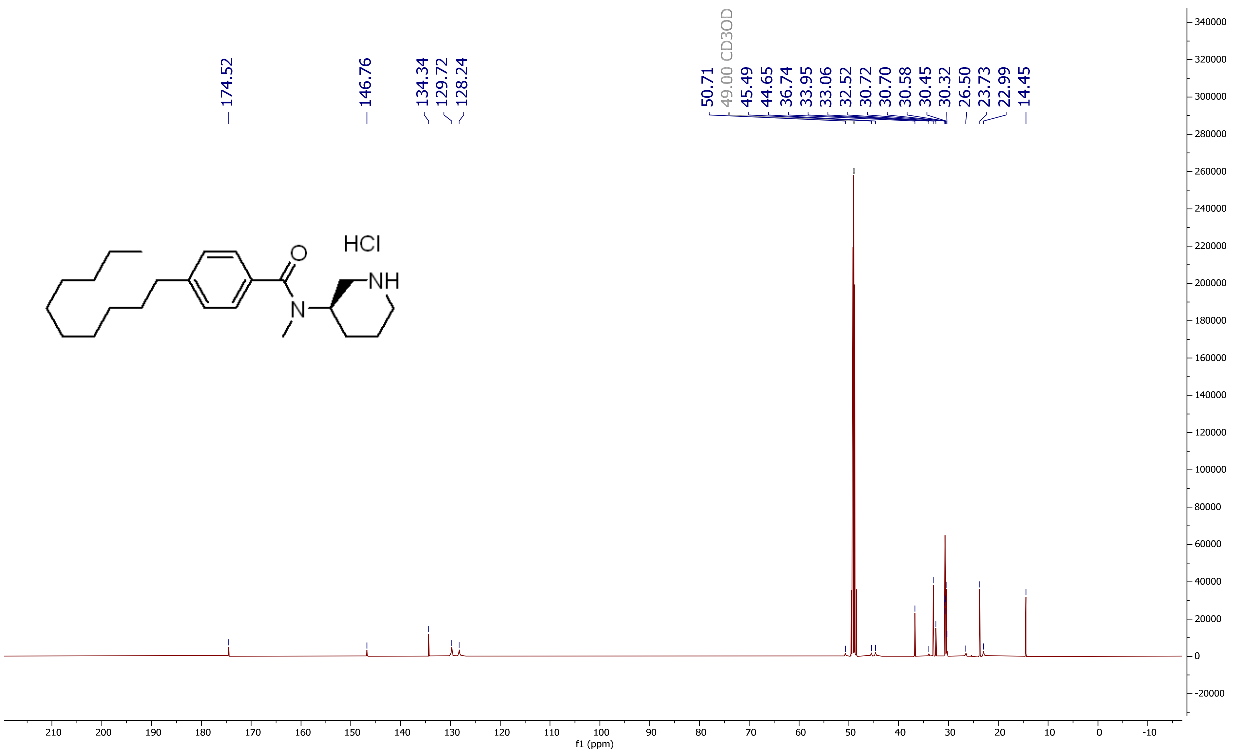

^{1}H NMR (400 MHz, CD_3OD) **15a**

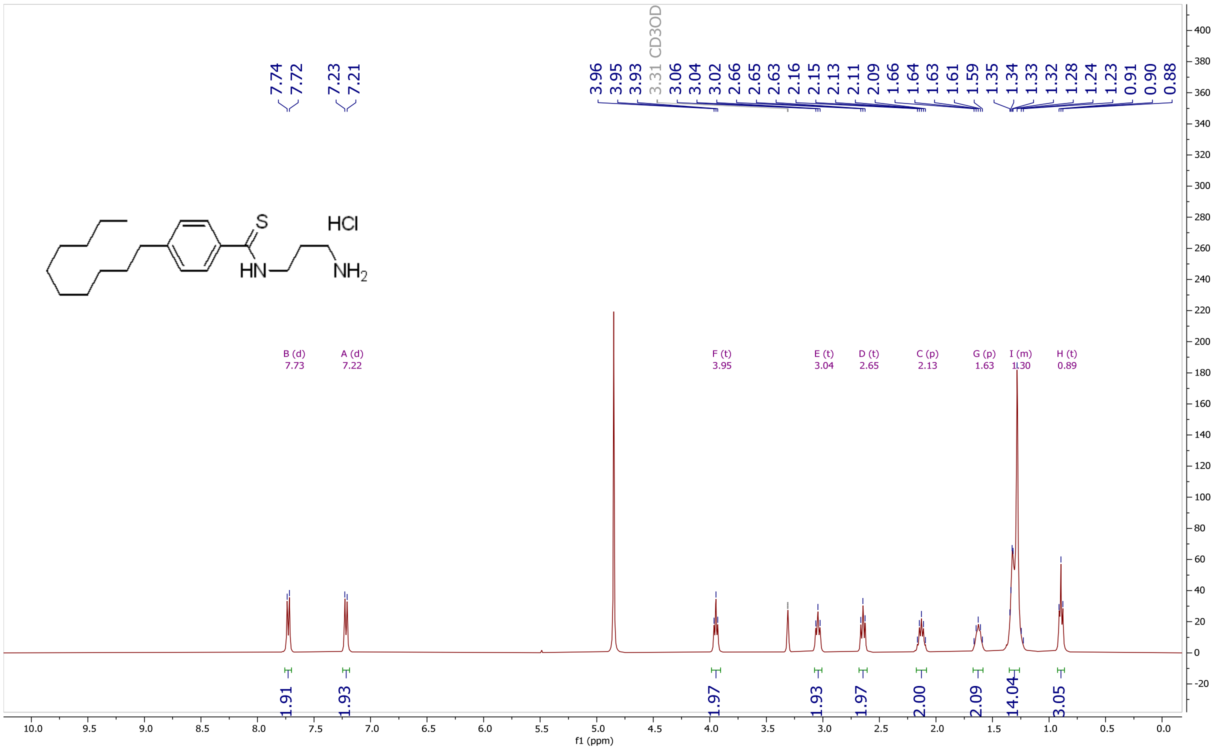

^{13}C NMR (101 MHz, CD_3OD) **15a**

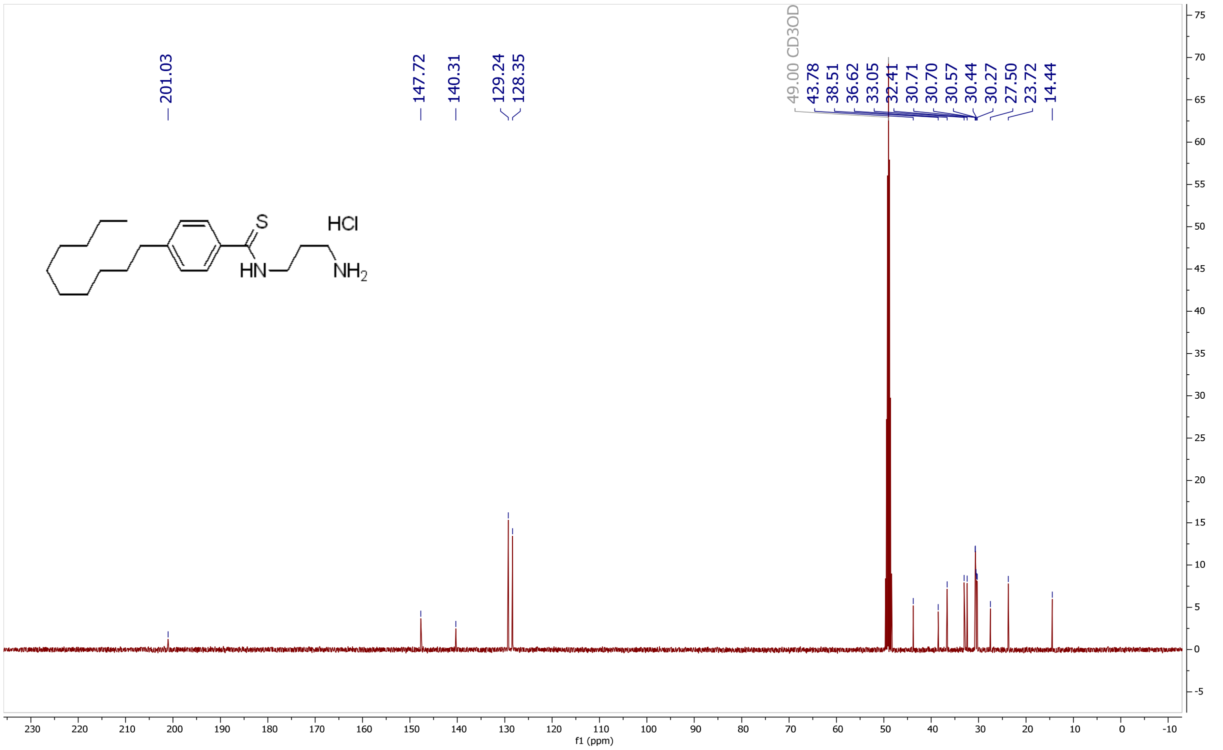

^{1}H NMR (400 MHz, CD_3OD) **15b**

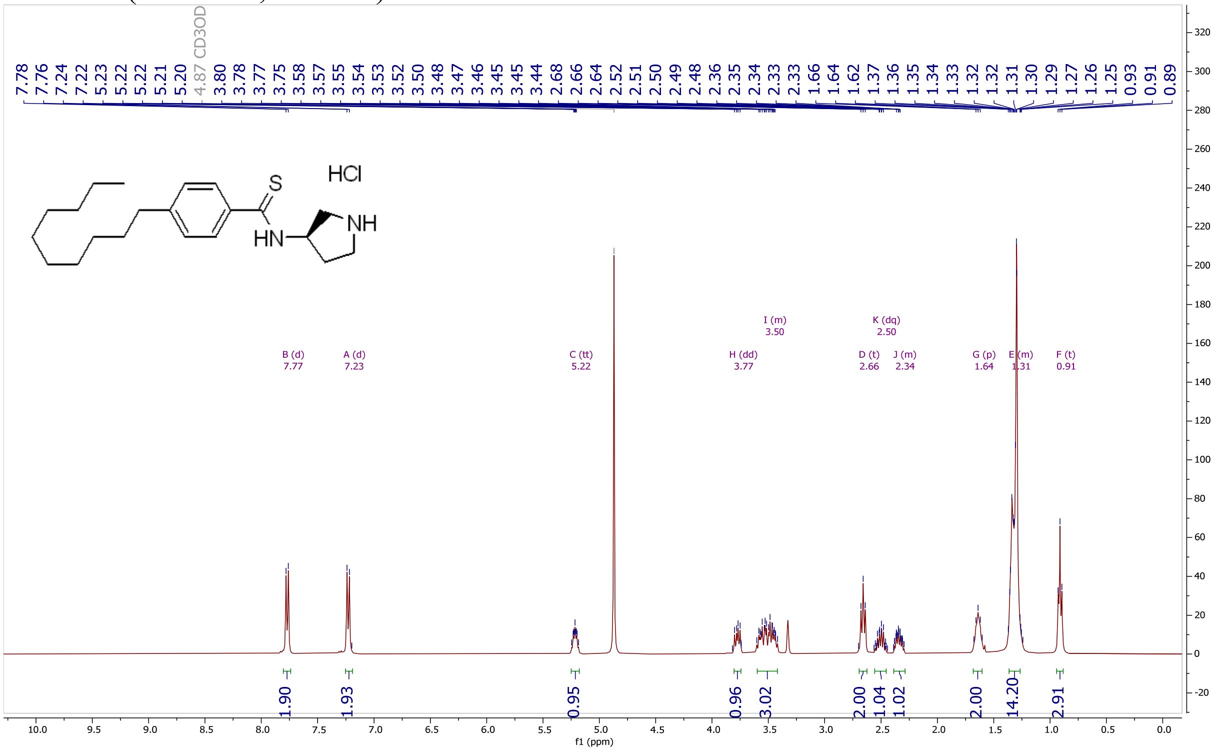

^{13}C NMR (101 MHz, CD_3OD) **15b**

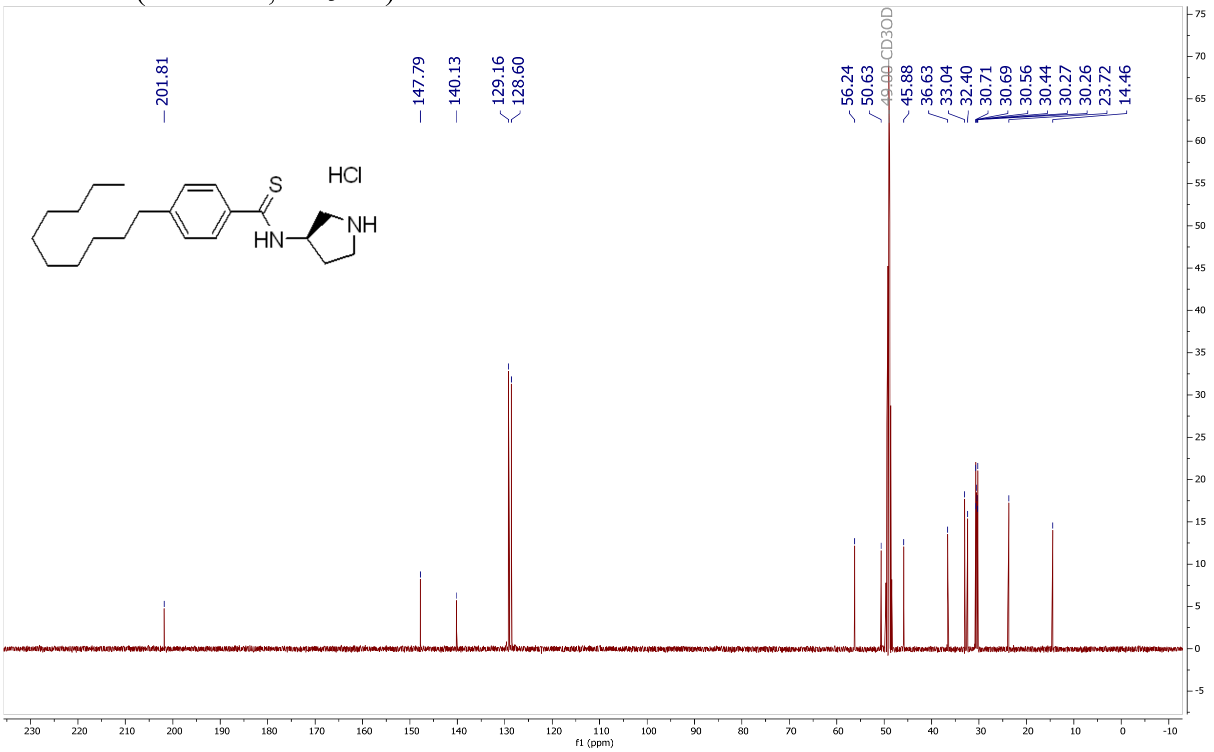

^{1}H NMR (400 MHz, CD_3OD) **15c**

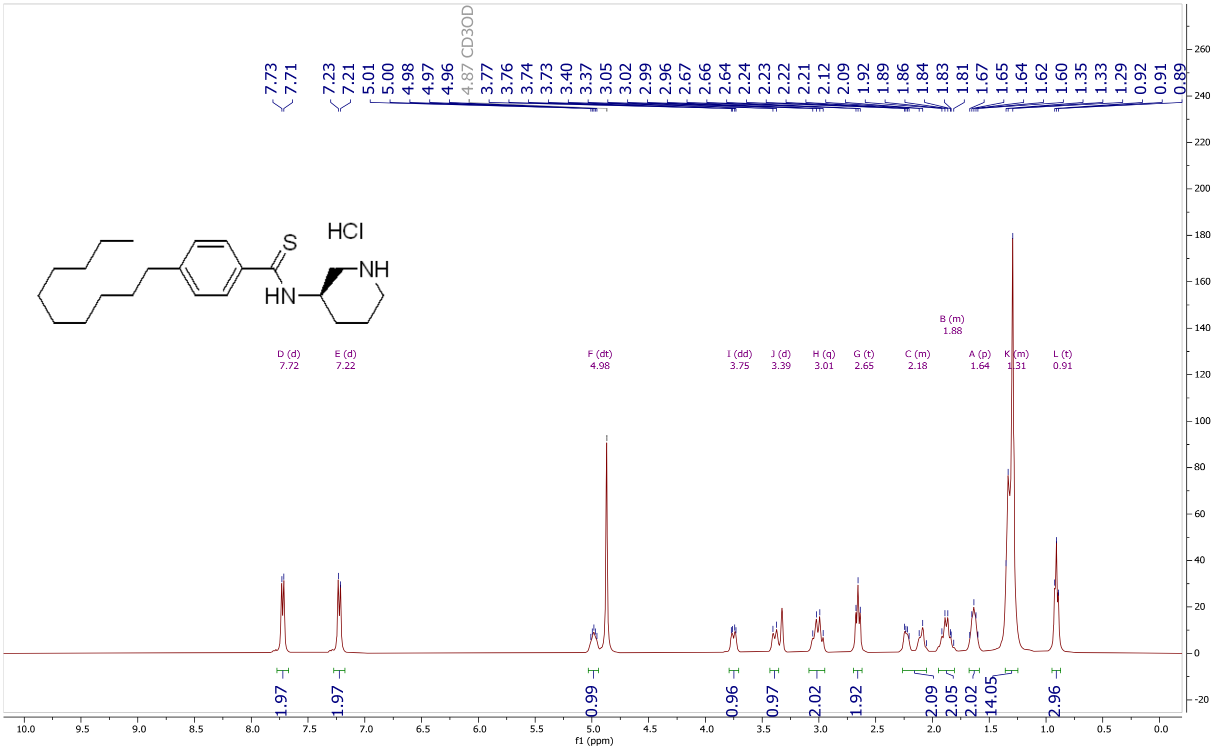

^{13}C NMR (101 MHz, CD_3OD) **15c**

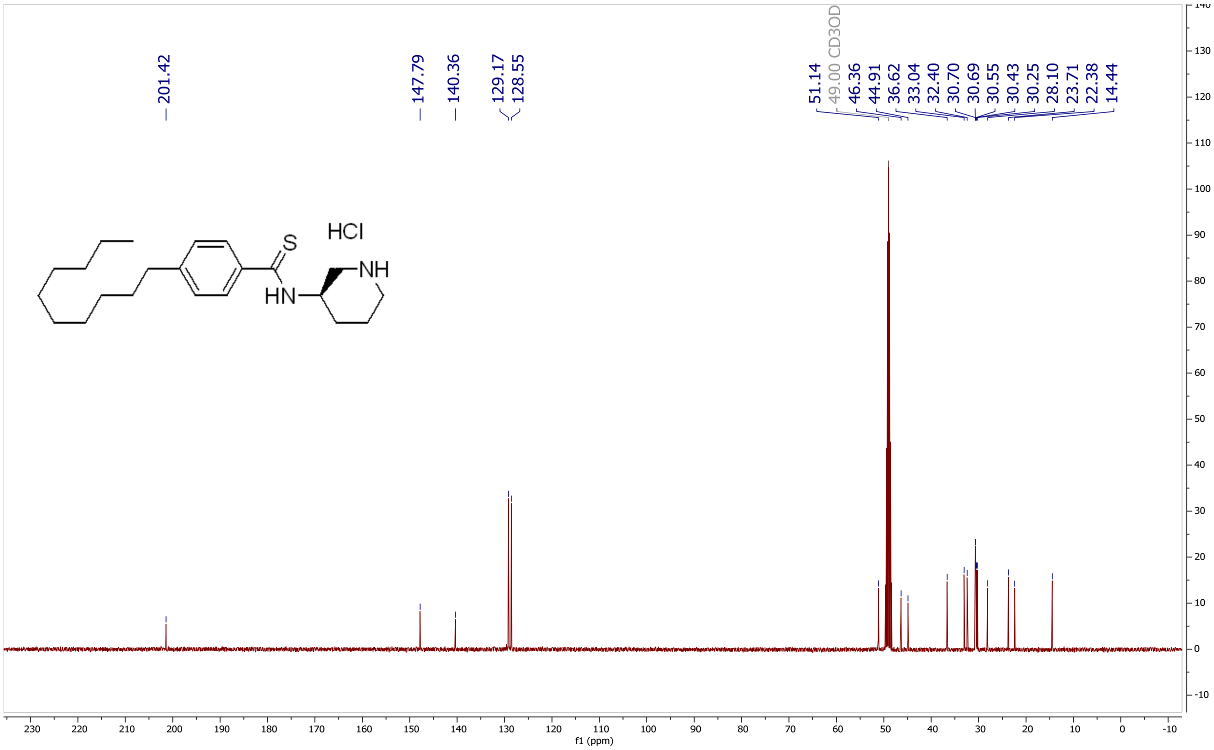

^{1}H NMR (400 MHz, CD_3OD) **17a**

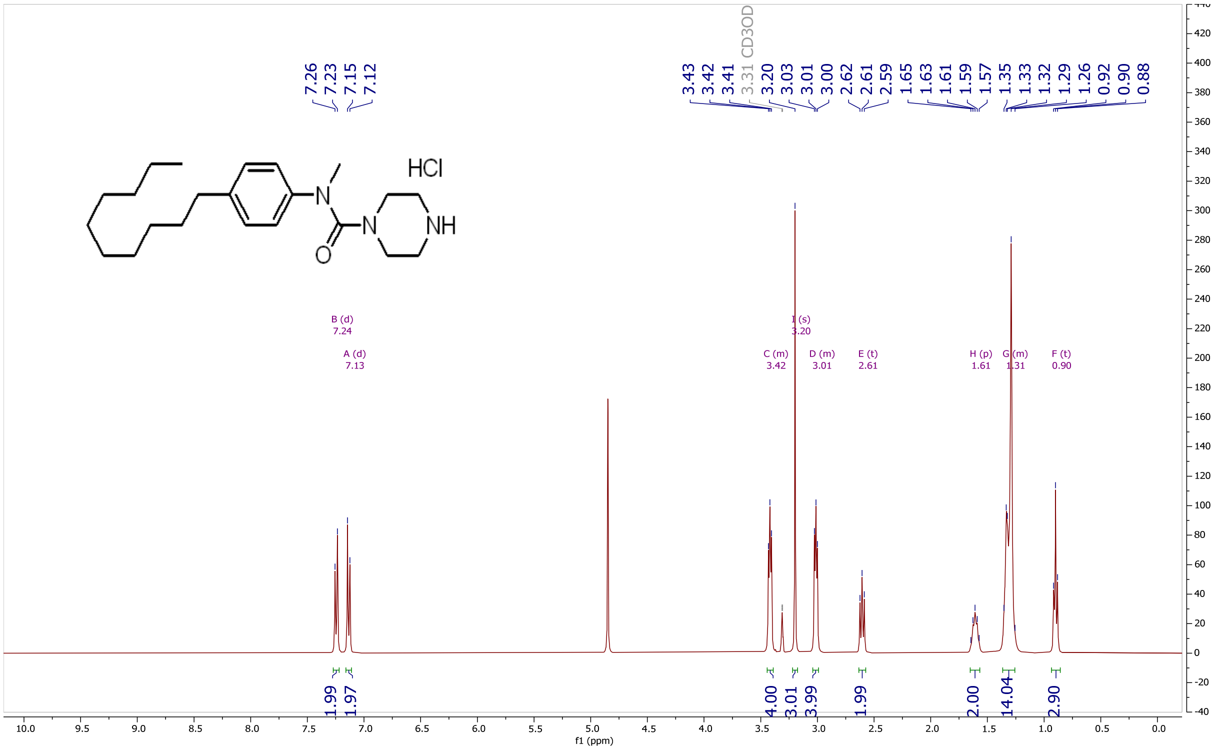

^{13}C NMR (101 MHz, CD_3OD) **17a**

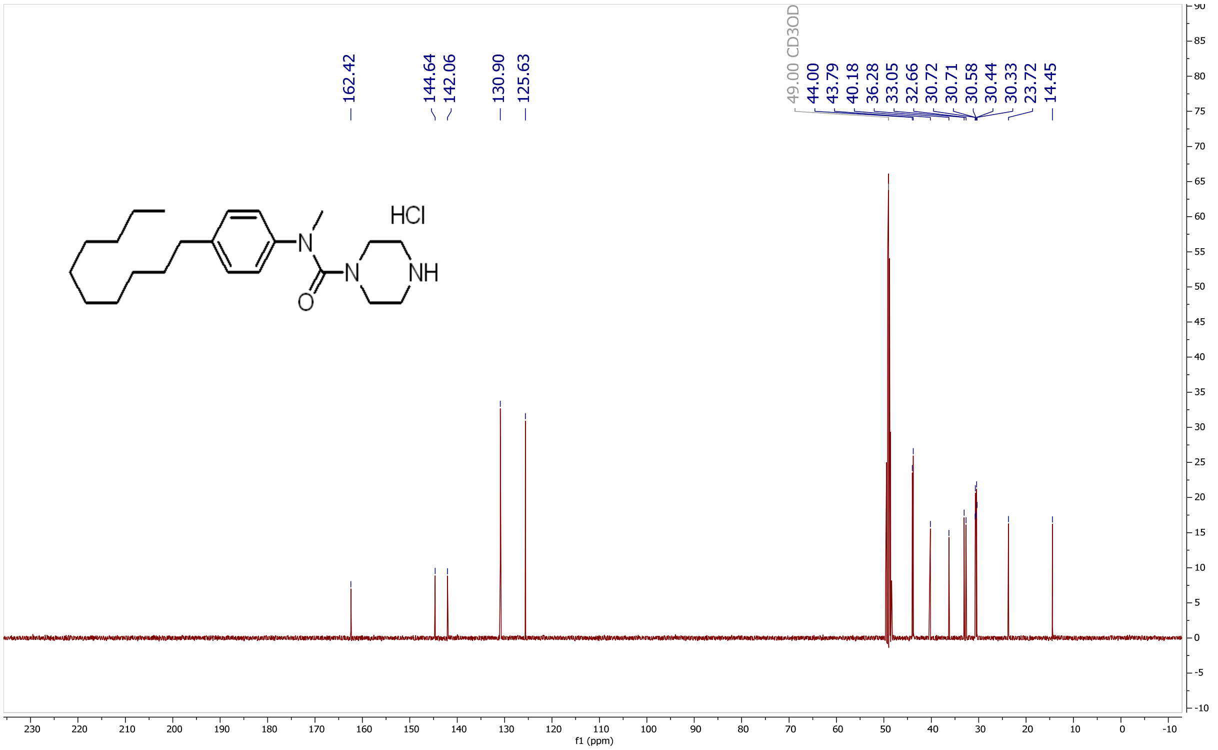

^{1}H NMR (400 MHz, CD_3OD) **17b**

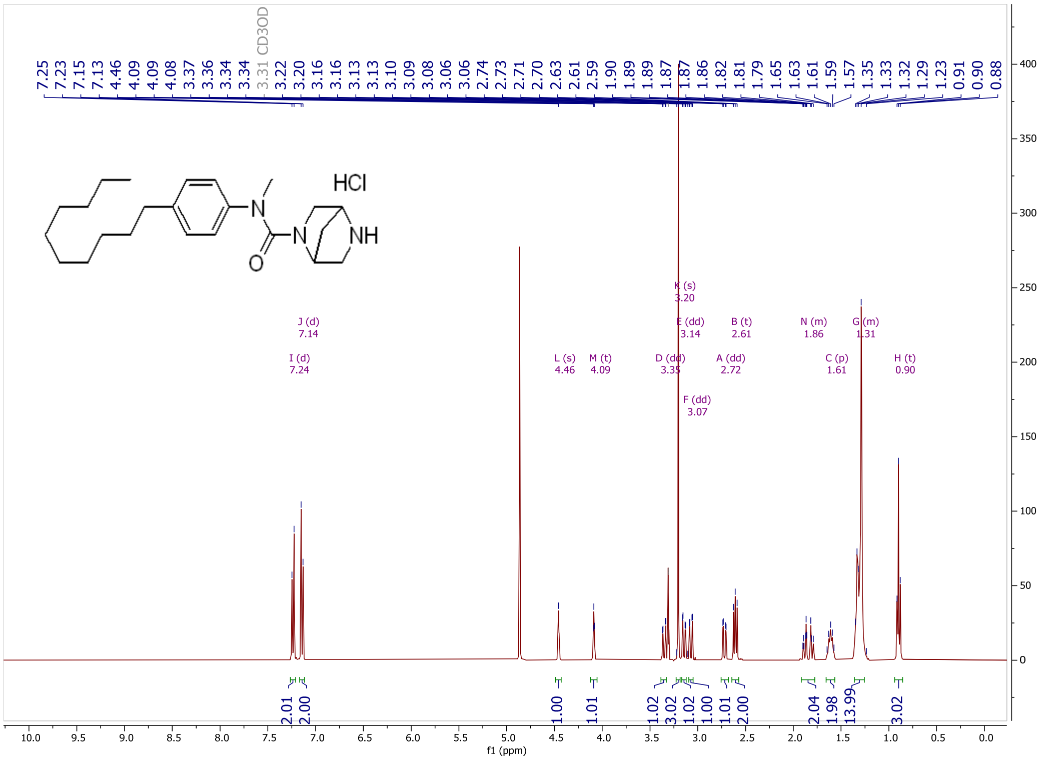

^{13}C NMR (126 MHz, CD_3OD) **17b**

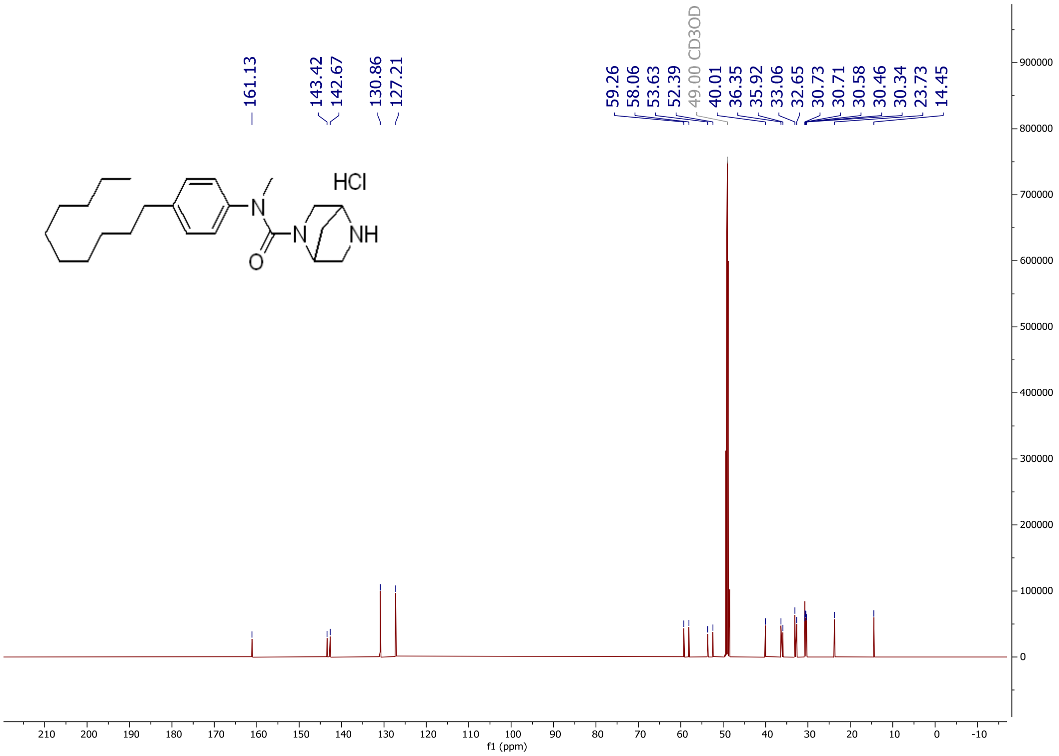

^{1}H NMR (400 MHz, CD_3OD) **17c**

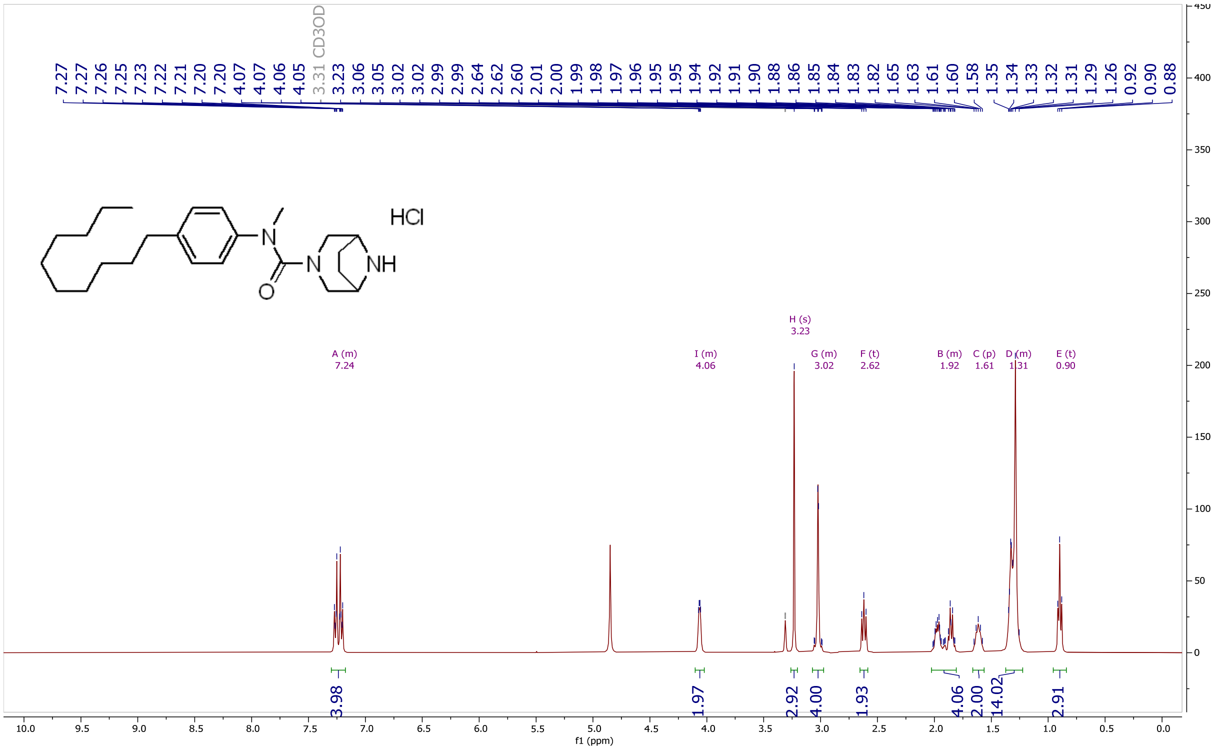

^{13}C NMR (101 MHz, CD_3OD) **17c**

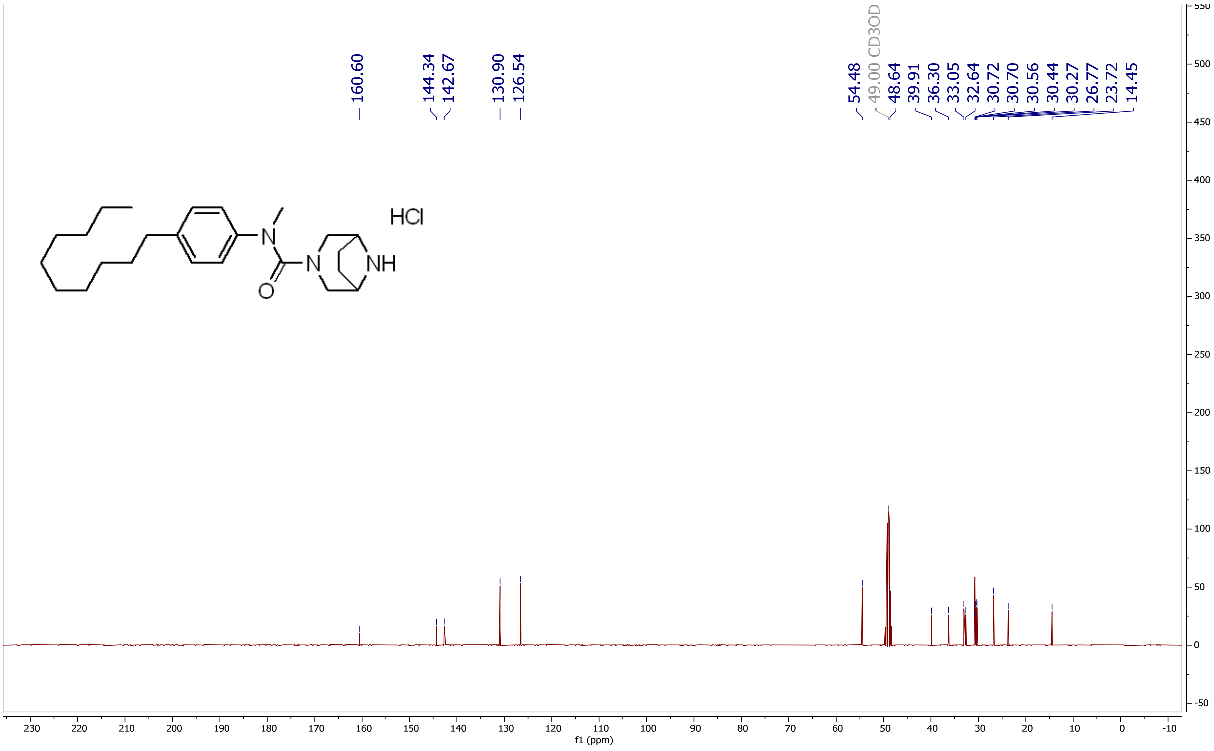

^{1}H NMR (400 MHz, CD_3OD) **18a**

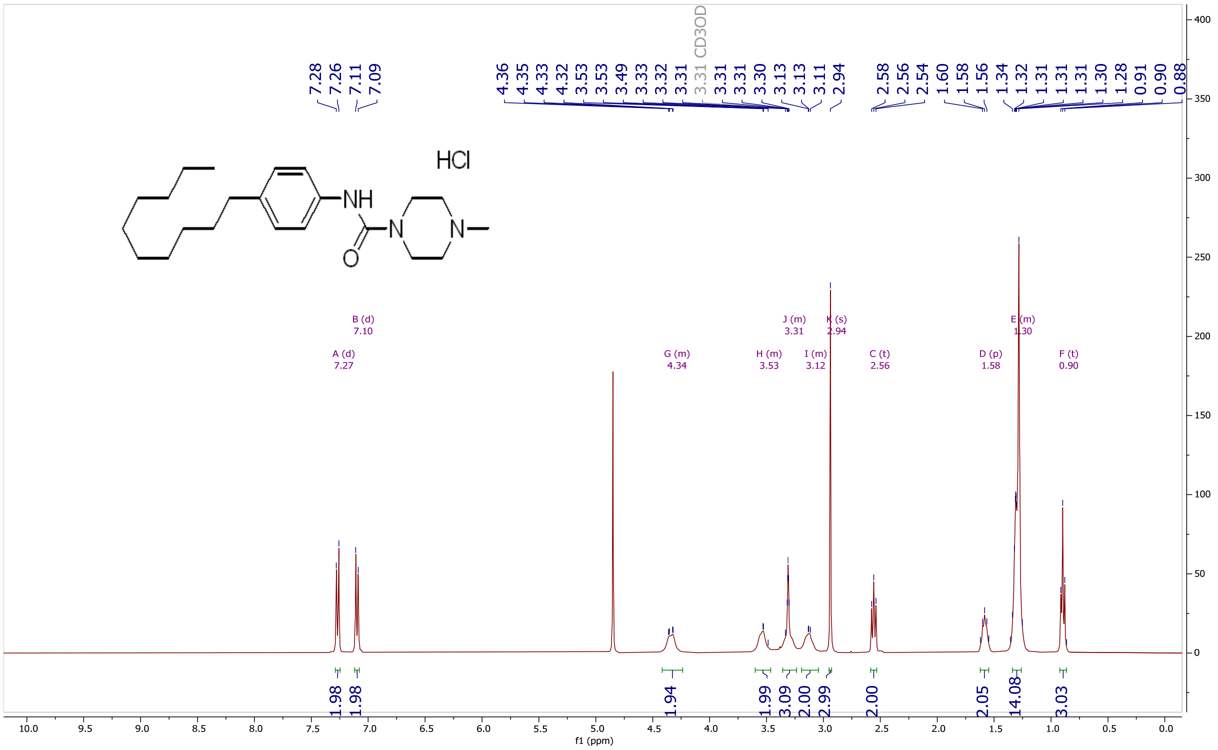

^{13}C NMR (101 MHz, CD_3OD) **18a**

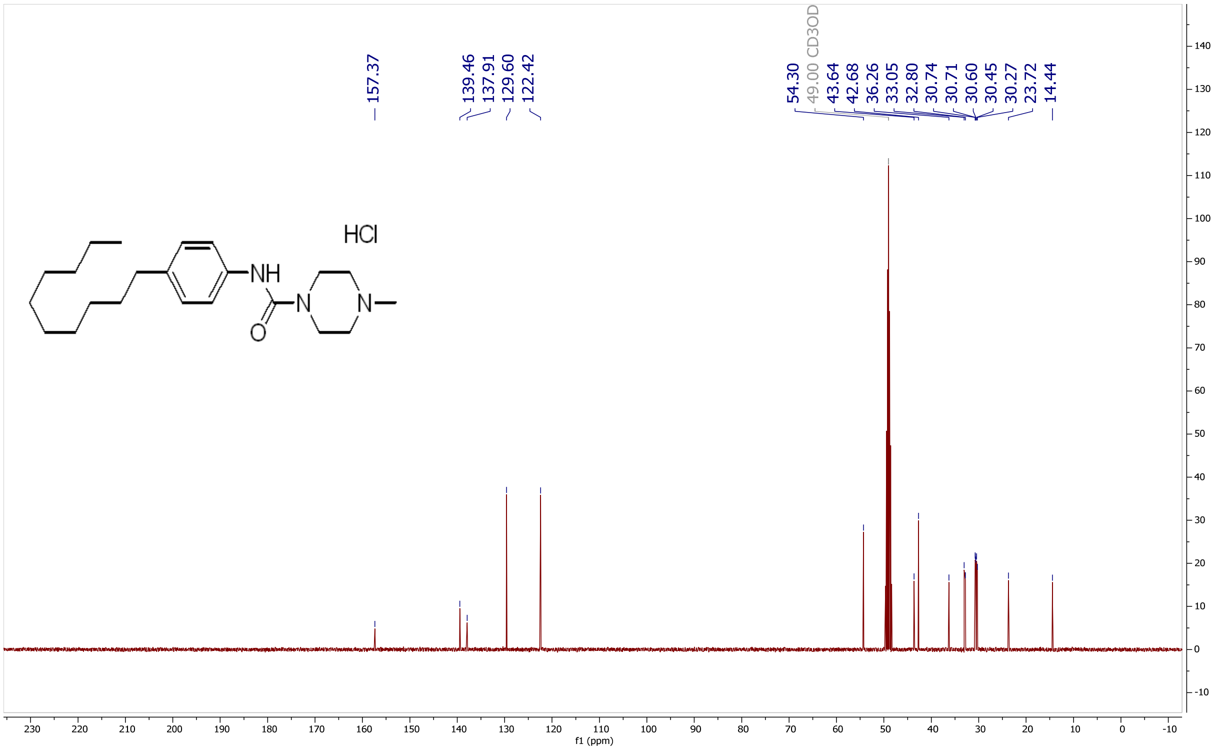

^{1}H NMR (400 MHz, CD_3OD) **18b**

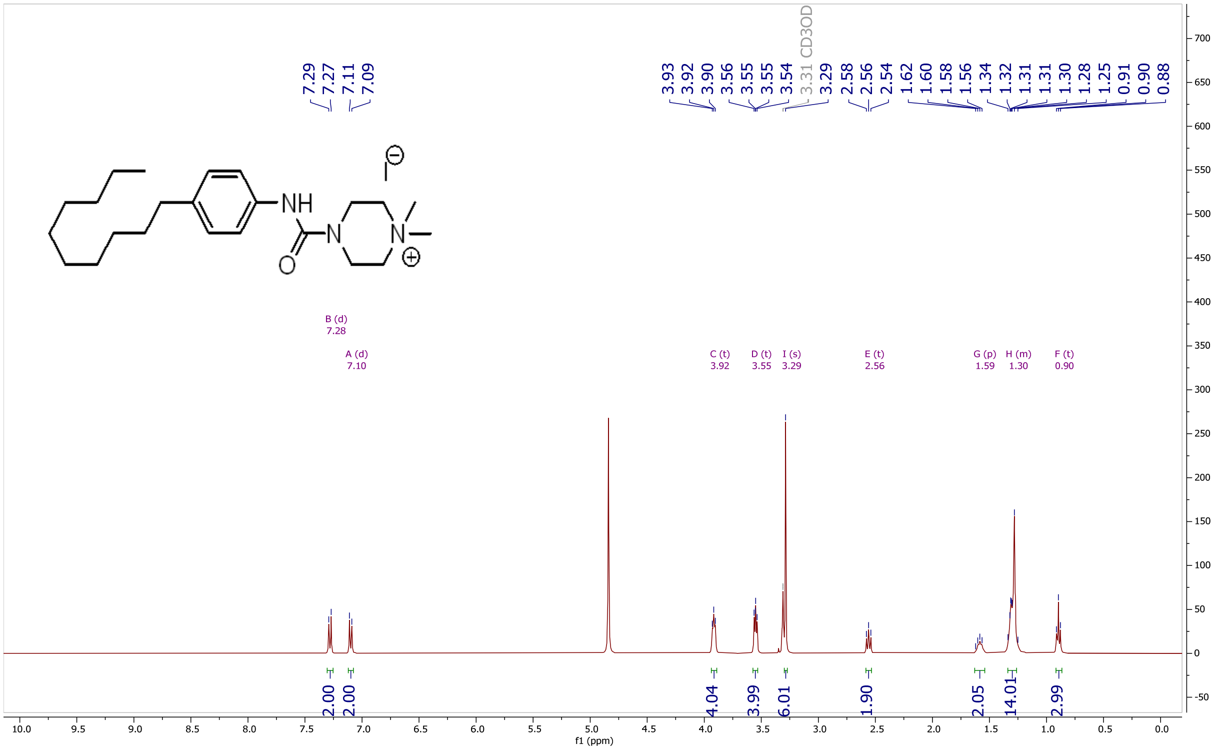

^{13}C NMR (101 MHz, CD_3OD) **18b**

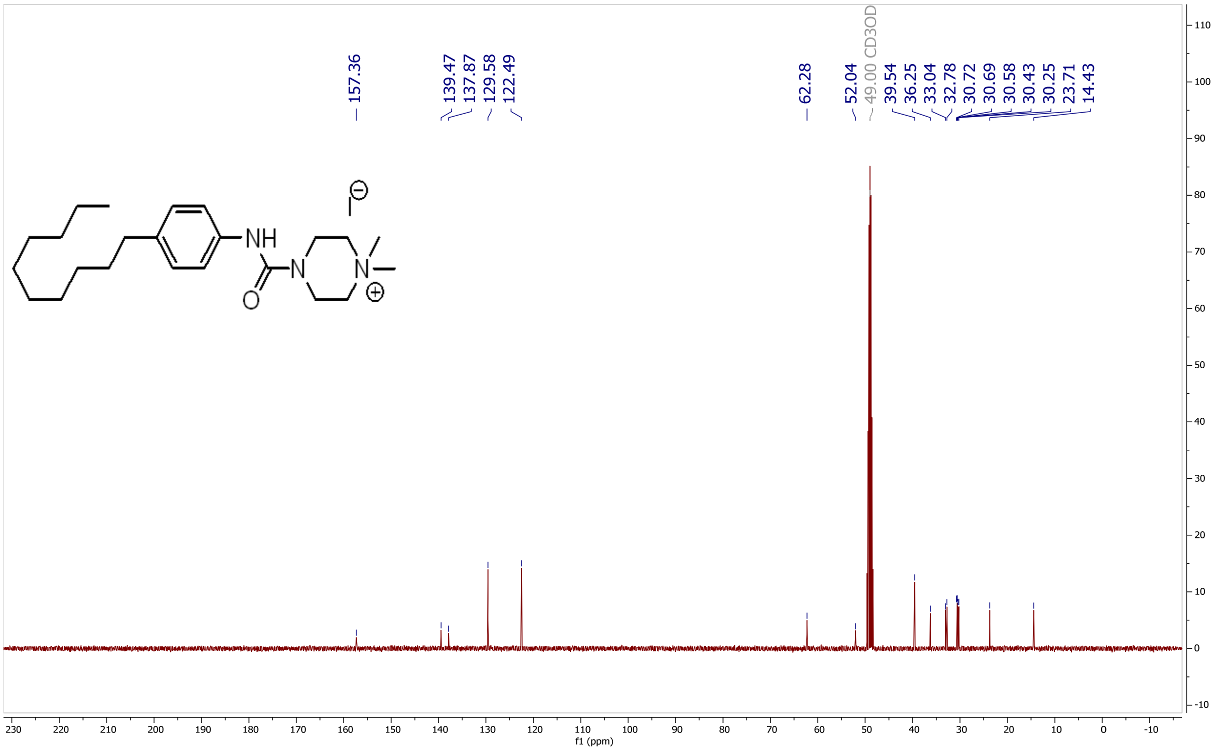

^{1}H NMR (500 MHz, CD_3OD) **20a**

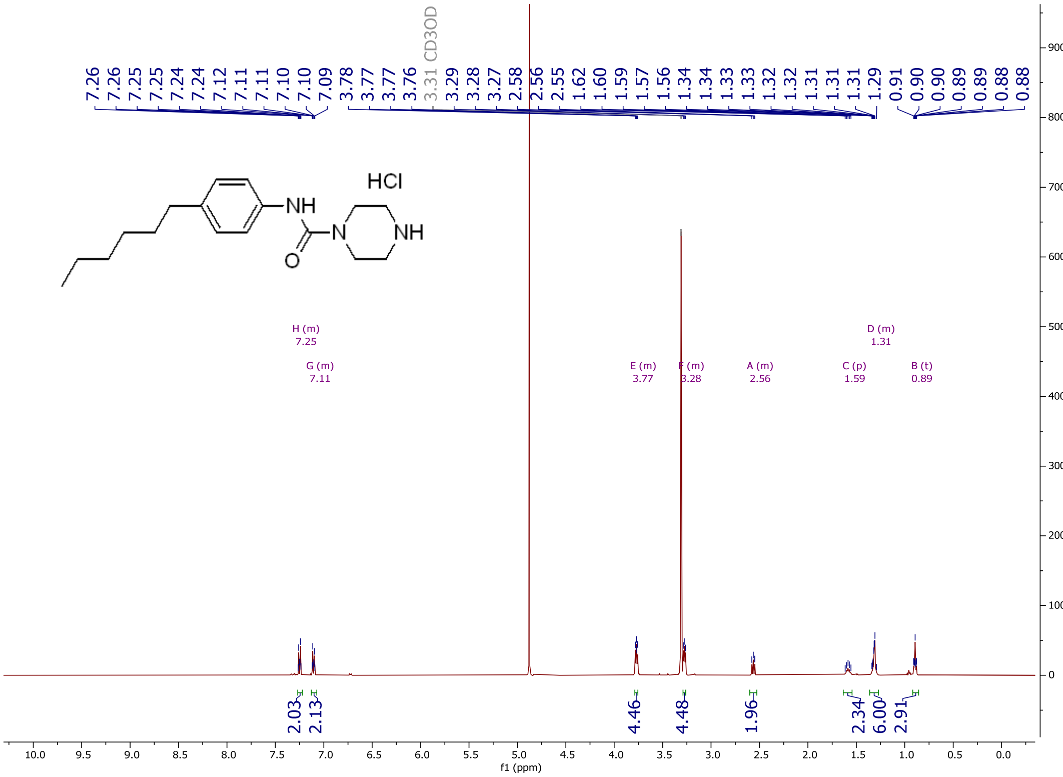

^{13}C NMR (126 MHz, CD_3OD) **20a**

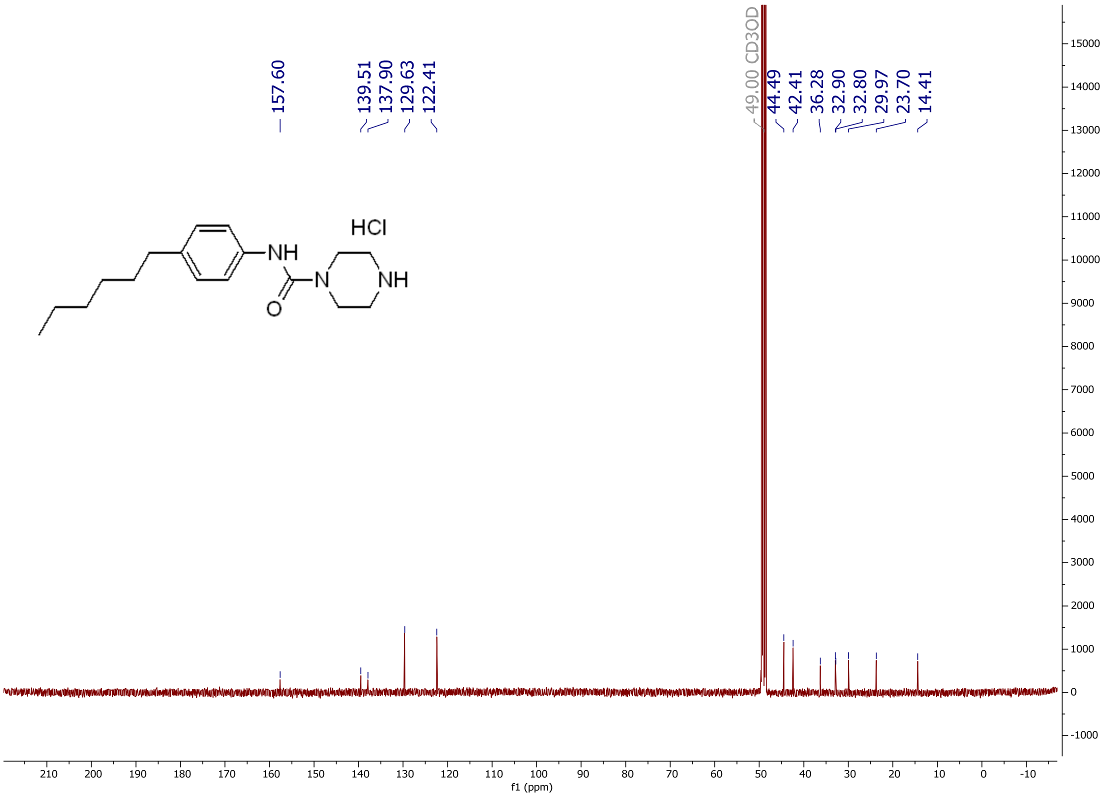

^{1}H NMR (500 MHz, CD_3OD) **20b**

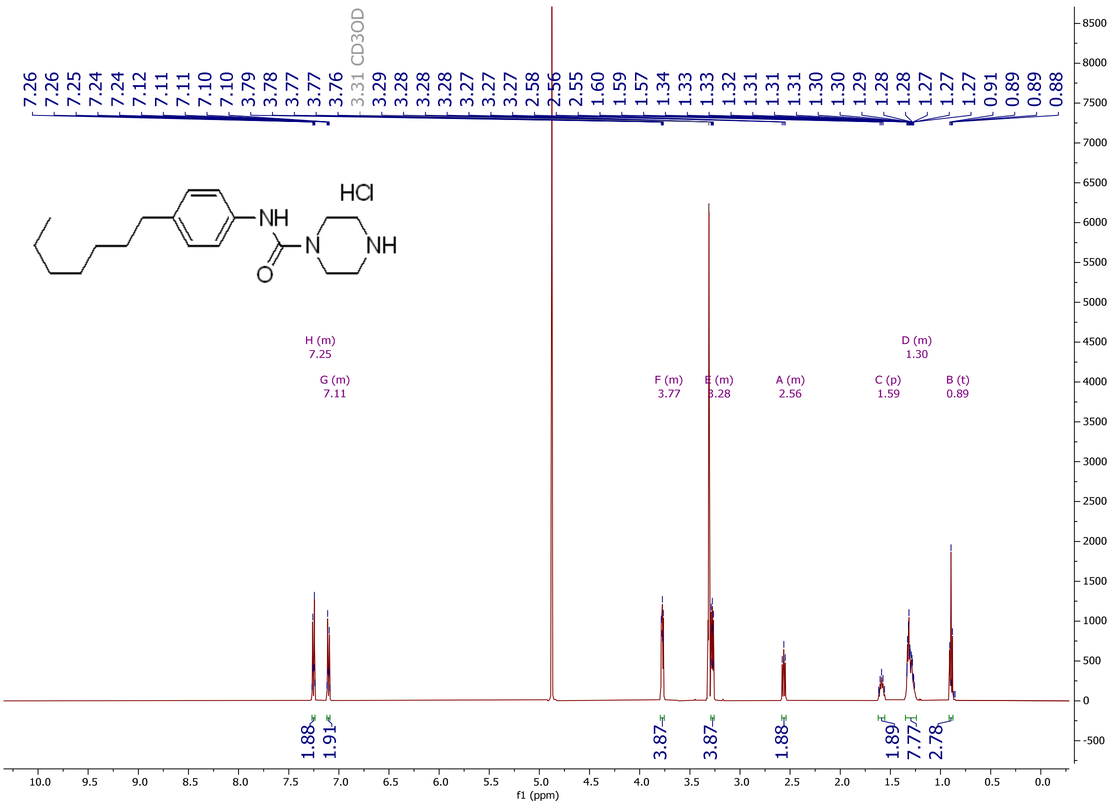

^{13}C NMR (126 MHz, CD_3OD) **20b**

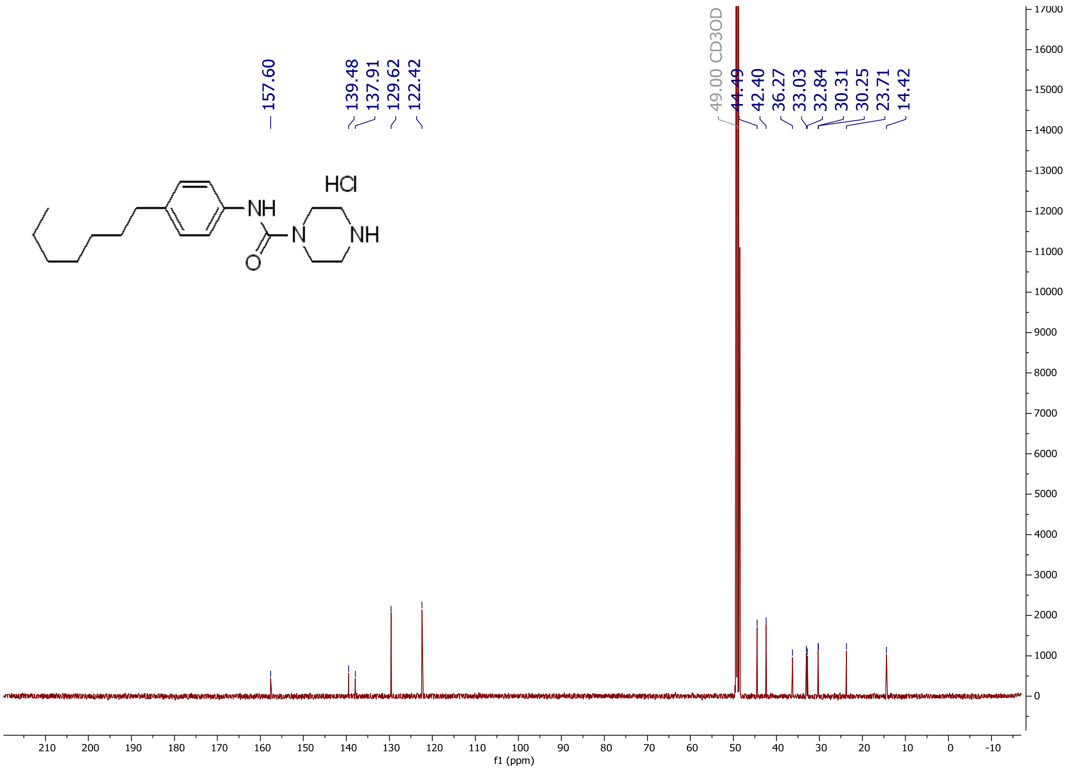

^{1}H NMR (500 MHz, CD_3OD) **20c**

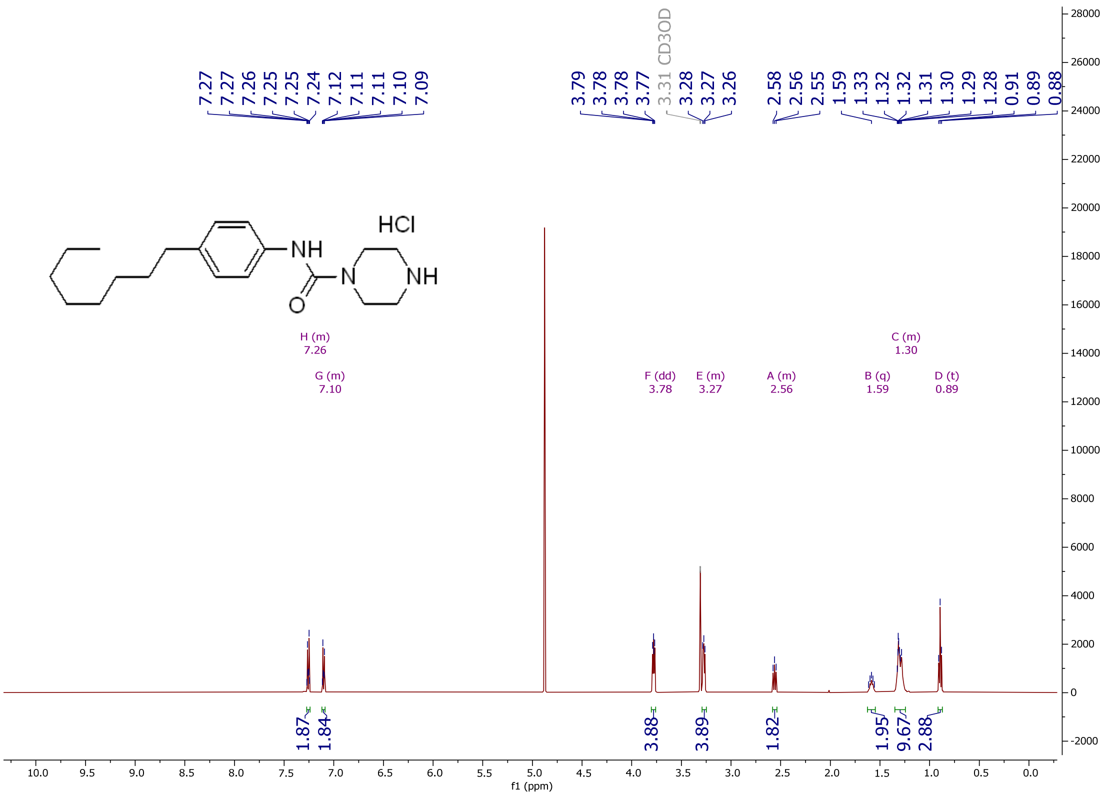

^{13}C NMR (126 MHz, CD_3OD) **20c**

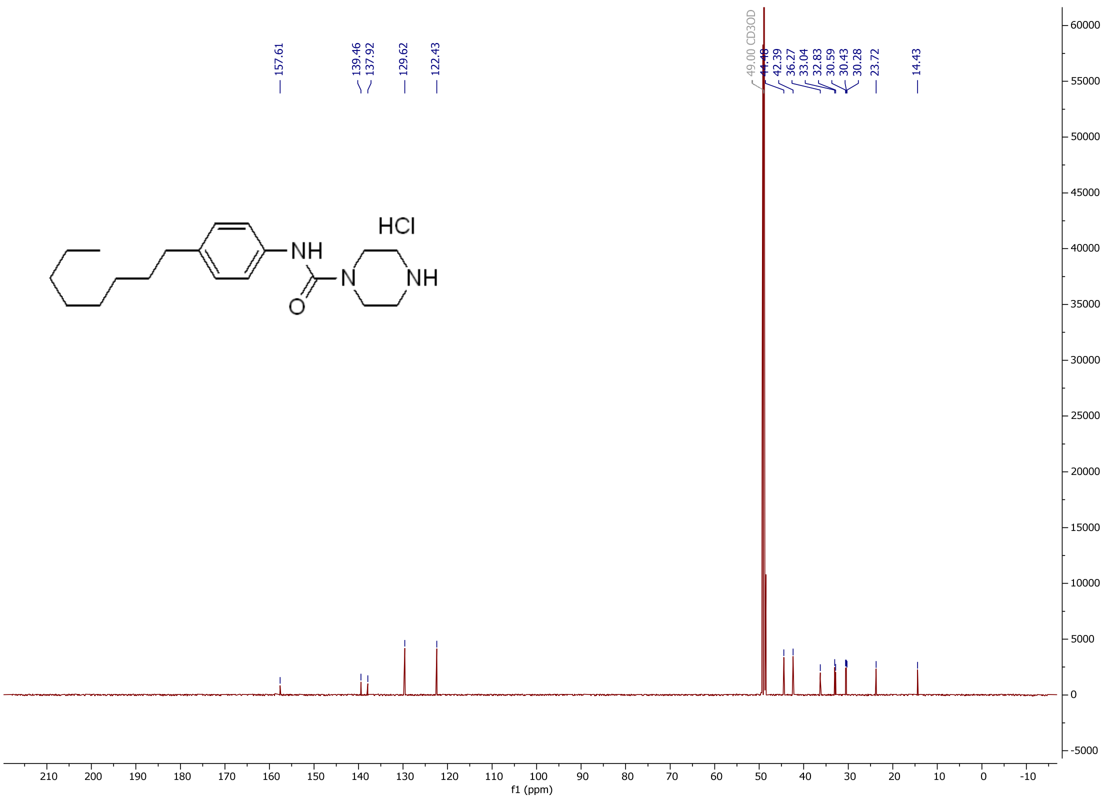

^{1}H NMR (500 MHz, CD_3OD) **20d**

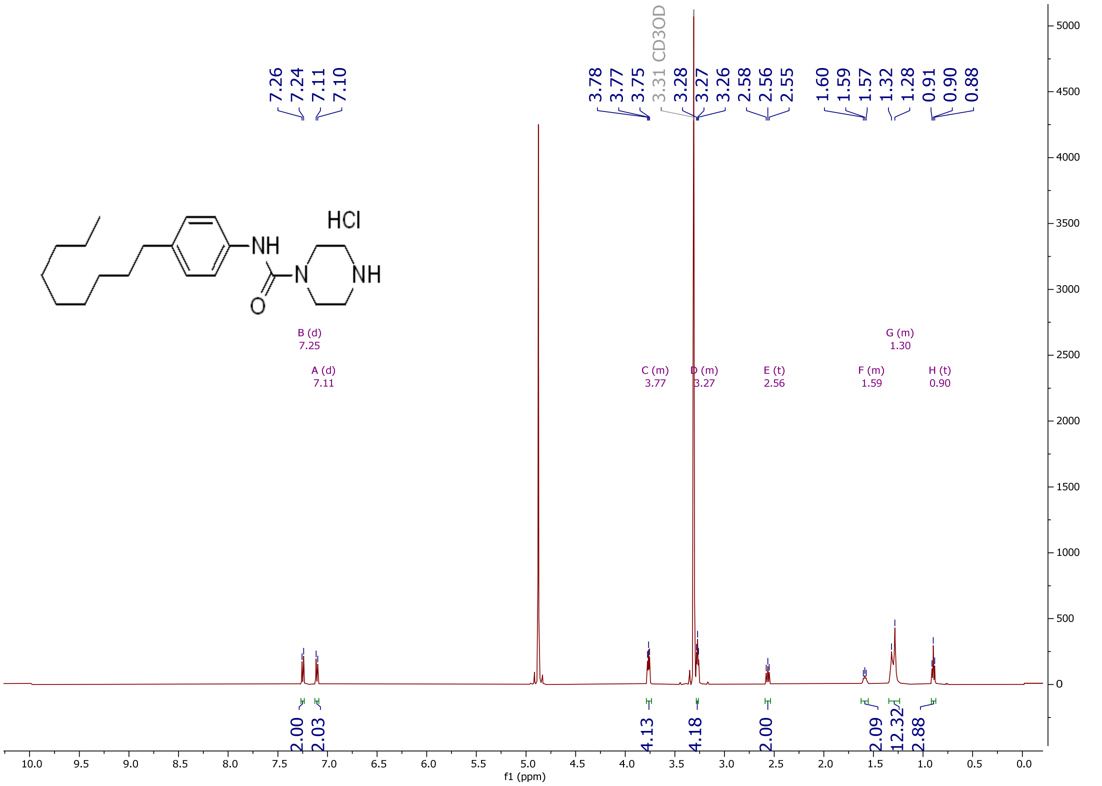

^{13}C NMR (126 MHz, CD_3OD) **20d**

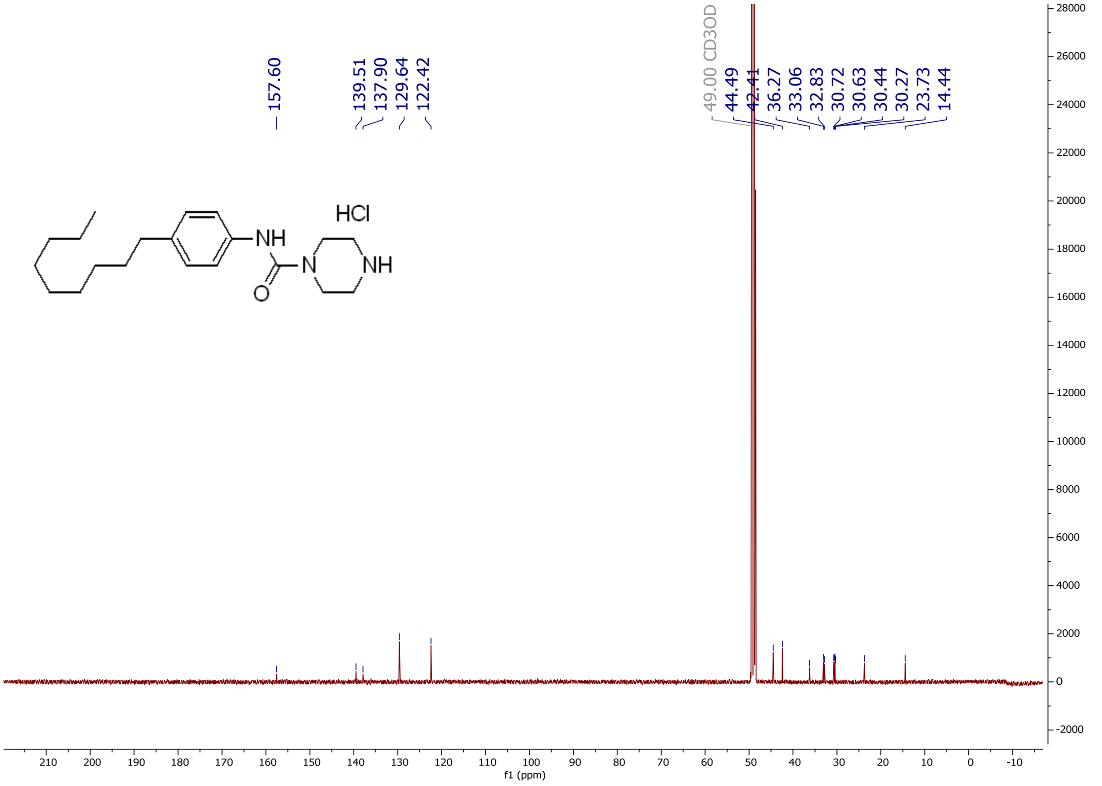

^{1}H NMR (500 MHz, CD_3OD) **20e**

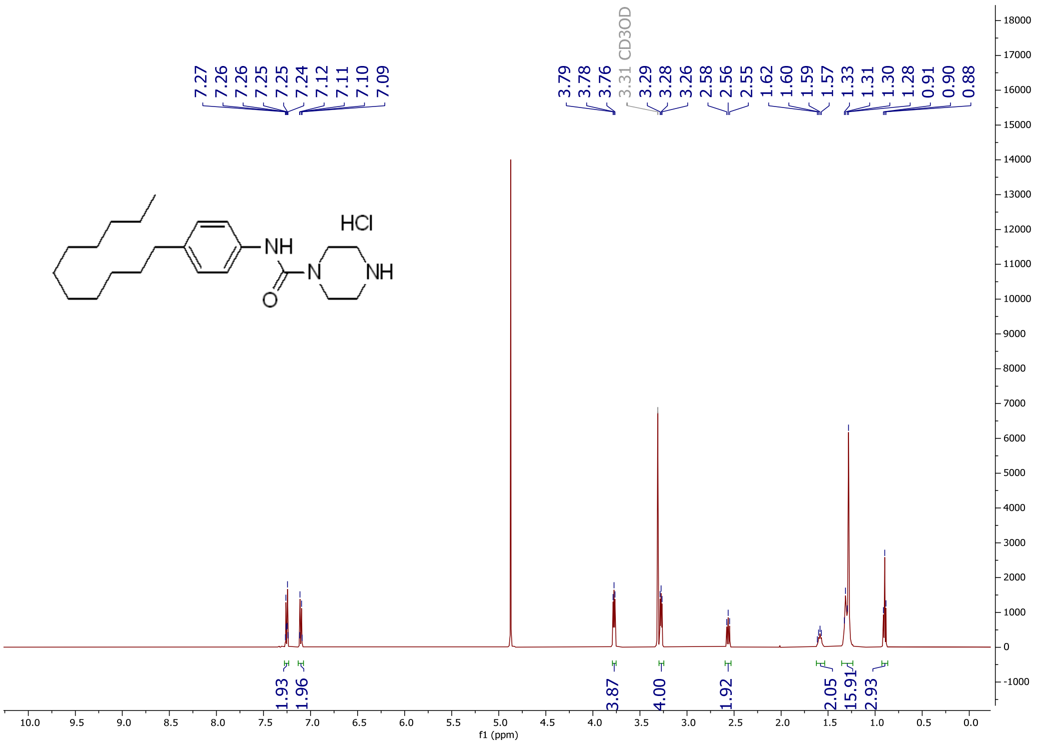

^{13}C NMR (126 MHz, CD_3OD) **20e**

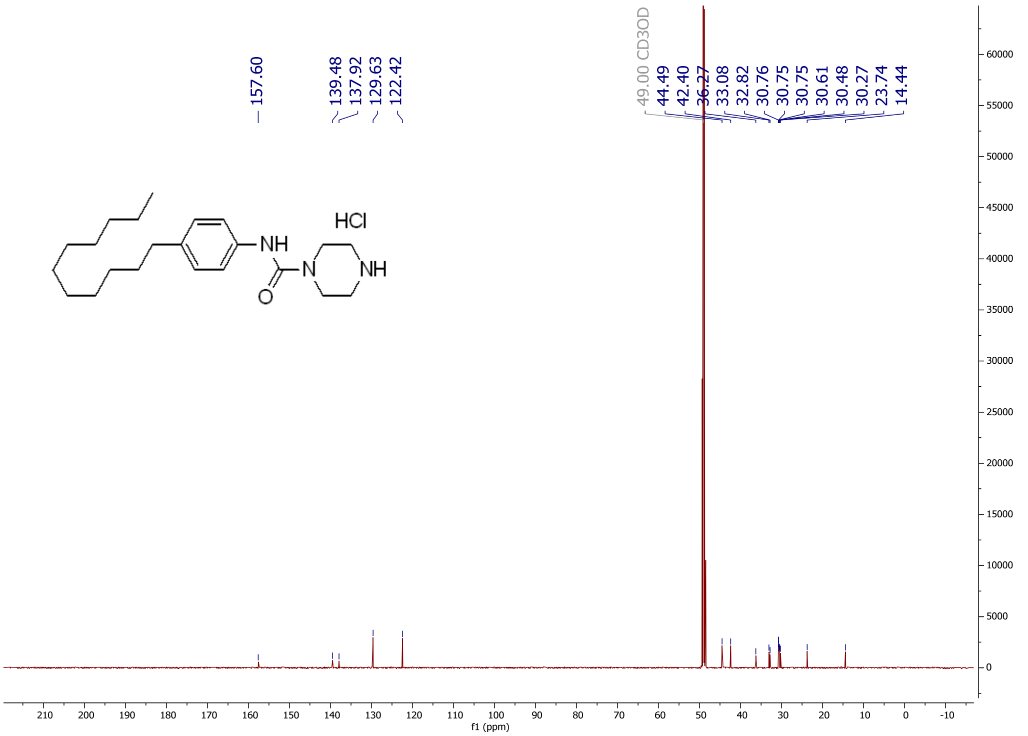

^{1}H NMR (600 MHz, CD_3OD) **20f**

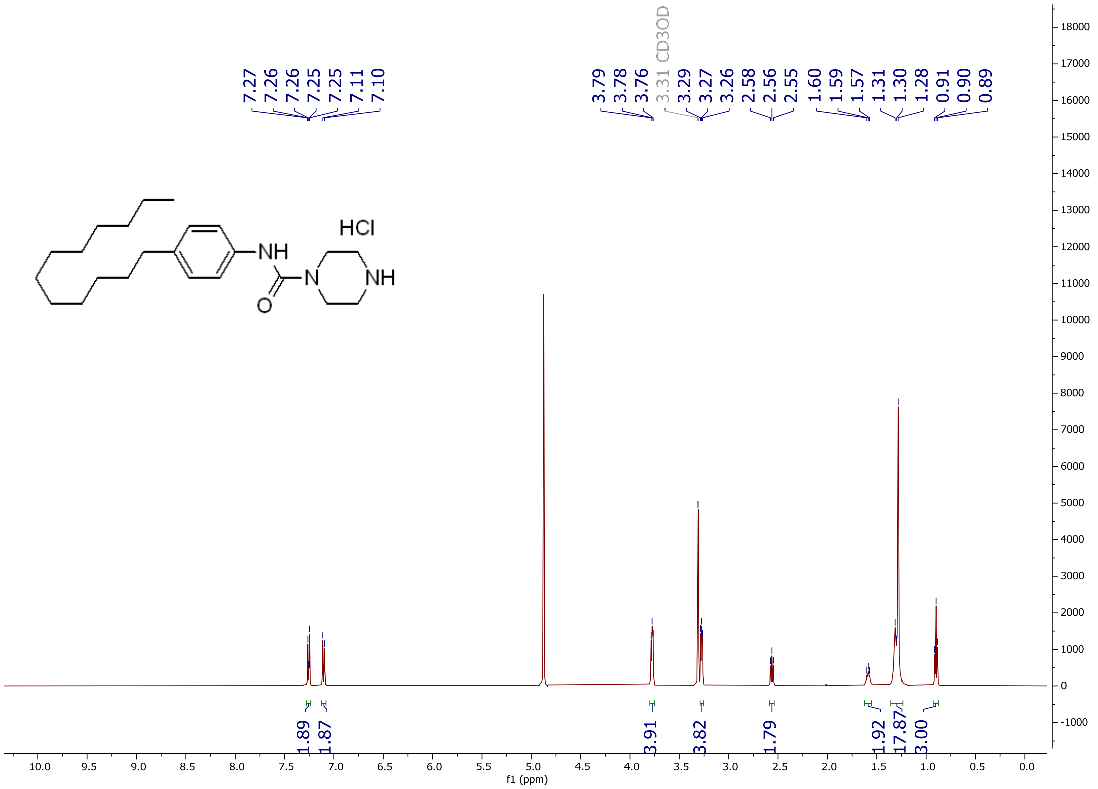

^{13}C NMR (151 MHz, CD_3OD) **20f**

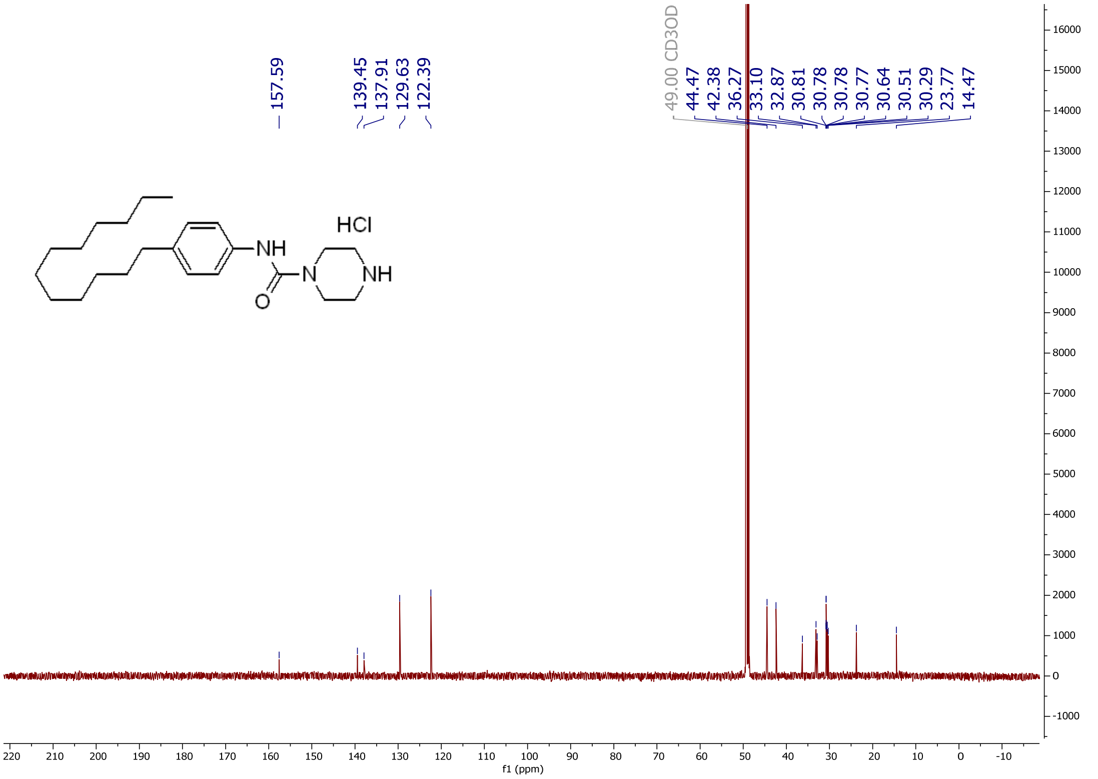

^{1}H NMR (600 MHz, DMSO) **20g**

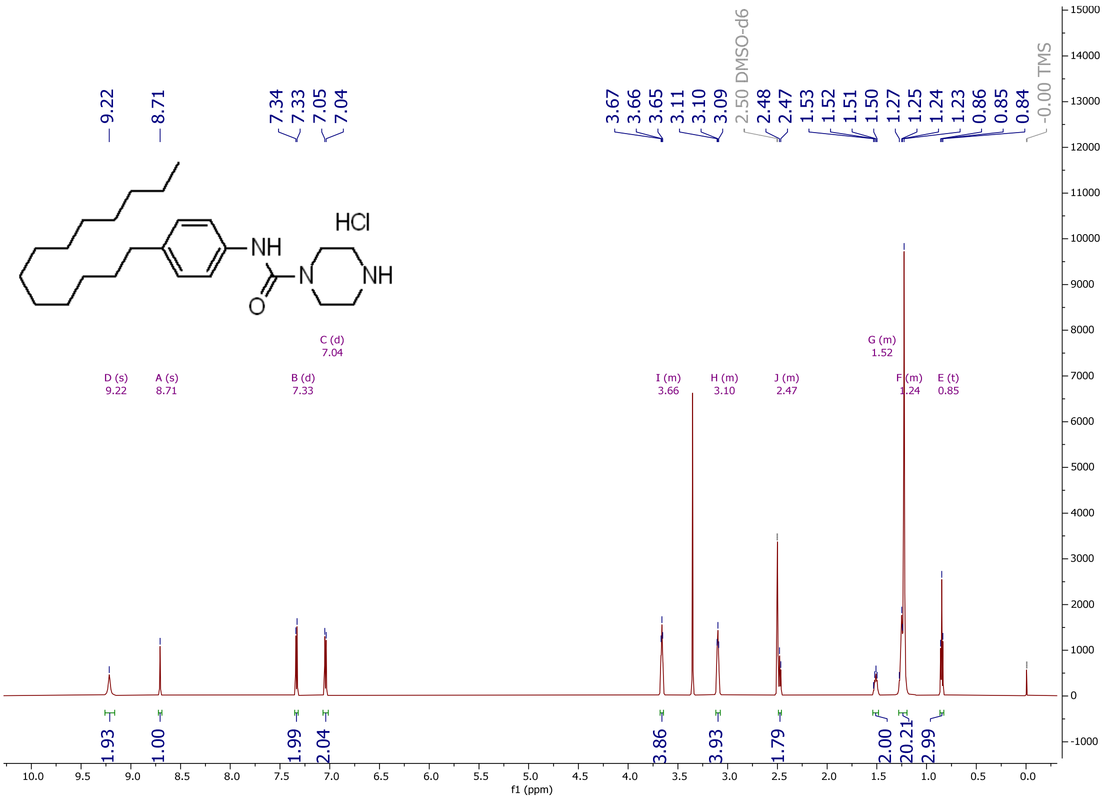

^{13}C NMR (151 MHz, DMSO) **20g**

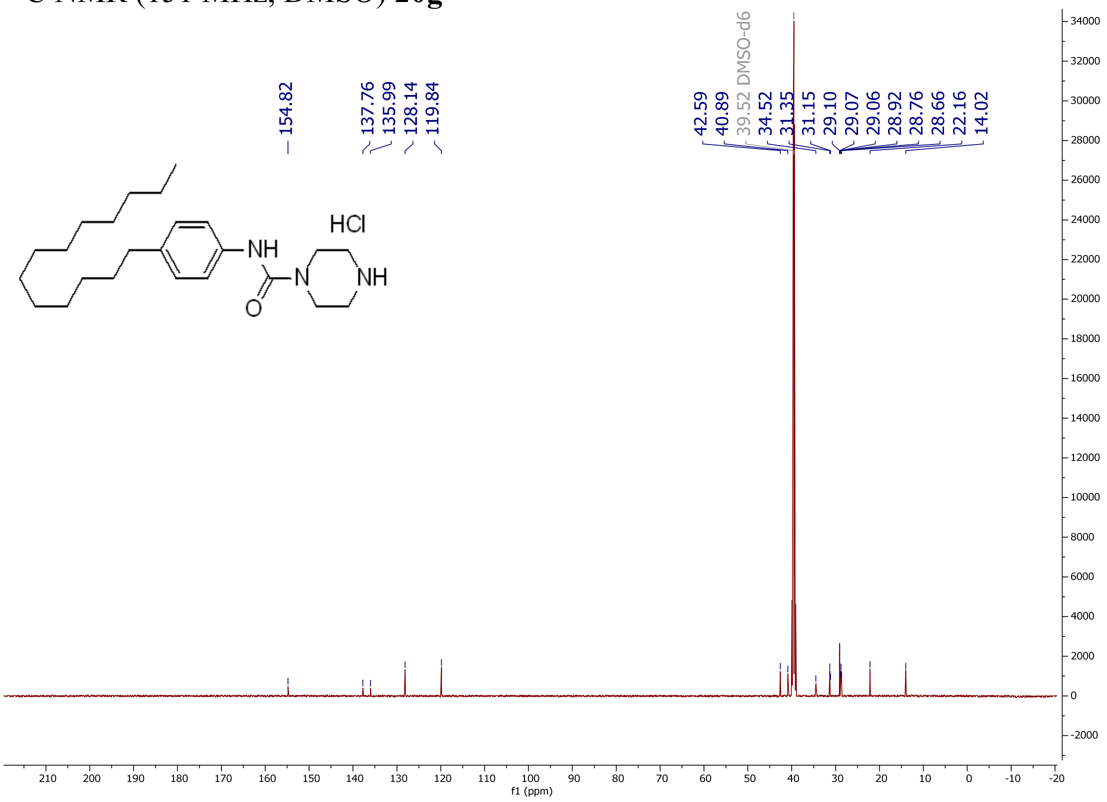

Supplement: Supplementary file 1 — jm4c00879_si_001.pdf [file jm4c00879_si_001.pdf]
